# Integrative Longitudinal Analysis of Metabolic Phenotype and Microbiota Changes During the Development of Obesity

**DOI:** 10.3389/fcimb.2021.671926

**Published:** 2021-08-03

**Authors:** Keah V. Higgins, Lauren N. Woodie, Haley Hallowell, Michael W. Greene, Elizabeth Hiltbold Schwartz

**Affiliations:** ^1^Department of Biological Sciences Auburn University, Auburn, AL, United States; ^2^Department of Nutrition, Dietetics and Hospitality Management, Auburn University, Auburn, AL, United States

**Keywords:** metabolic phenotype, microbiota, bacteriophage, obesity, bacteria-phage dynamics

## Abstract

Obesity has increased at an alarming rate over the past two decades in the United States. In addition to increased body mass, obesity is often accompanied by comorbidities such as Type II Diabetes Mellitus and metabolic dysfunction-associated fatty liver disease, with serious impacts on public health. Our understanding of the role the intestinal microbiota in obesity has rapidly advanced in recent years, especially with respect to the bacterial constituents. However, we know little of when changes in these microbial populations occur as obesity develops. Further, we know little about how other domains of the microbiota, namely bacteriophage populations, are affected during the progression of obesity. Our goal in this study was to monitor changes in the intestinal microbiome and metabolic phenotype following western diet feeding. We accomplished this by collecting metabolic data and fecal samples for shotgun metagenomic sequencing in a mouse model of diet-induced obesity. We found that after two weeks of consuming a western diet (WD), the animals weighed significantly more and were less metabolically stable than their chow fed counterparts. The western diet induced rapid changes in the intestinal microbiome with the most pronounced dissimilarity at 12 weeks. Our study highlights the dynamic nature of microbiota composition following WD feeding and puts these events in the context of the metabolic status of the mammalian host.

## Introduction

The obesity rate has almost tripled since 1975 in the United States (World Health Organization, 2016). Nearly 40% of the adult population was considered obese as recently as 2016 (Hales et al., 2015; World Health Organization, 2016). A contributing cause for this rapid rise is the prevalence and popularity of foods high in saturated fats and added sugars (Popkin and Gordon-Larsen, 2004; Popkin et al., 2012). Individuals consuming a high-fat western diet (WD) are prone to developing diseases comorbid with obesity such as Type-II Diabetes Mellitus, metabolic dysfunction-associated fatty liver disease, cardiovascular disease, irritable bowel disease and colon cancer  (Klein et al., 2004; Goodwin and Stambolic, 2015; Singh et al., 2017; Polyzos et al., 2020). The comorbidity of gastrointestinal (GI) disease and obesity have prompted investigations into potential links between the two disorders. Among the potential connections that have received a lot of attention are diet-induced alterations within the intestinal microbiota.

The intestinal microbiome is a diverse and dynamic microbial ecosystem residing within the GI tract. It consists primarily of organisms from the domain Bacteria, as well as populations of Archaea, Eukaryota, and numerous Viruses (Lewis et al., 2015; Hsu et al., 2019). Healthy diets, such as those high in plant polysaccharides, drive a colonic microbiota profile dominated by the phylum Bacteroidetes (Duncan et al., 2008). Members of the Firmicutes, Proteobacteria and Actinobacteria phyla are also common components of the intestinal microbiota (Human Microbiome Project Consortium, 2012). Resident commensal bacteria carry out important functions for the host such as synthesis of vitamins and short chain fatty acids, degradation of host dietary oxalates, and many other metabolic functions (Cantarel et al., 2012; Hollister et al., 2014). However, the microbiota can negatively impact the host when the balance of microbial populations becomes disrupted, a condition generally known as dysbiosis (Hollister et al., 2014). For example, the dysbiotic profile of the intestinal microbiota in obesity consistently reflects an increased abundance of Firmicutes, reversing the Bacteroidetes : Firmicutes ratio (Duncan et al., 2008; Howe et al., 2016). However, while consistent across numerous studies, this finding has been reported primarily at the endpoint of obesity. Thus, the kinetics with which microbial dysbiosis progresses during the development of obesity remains poorly defined.

Whereas much is known about the bacterial constituents within the microbiome (bacteriome), there is a substantial gap in our understanding of how other microbiome constituents, such as bacteriophage populations, change in abundance or composition during the development of obesity (Duncan et al., 2008; Sonnenburg et al., 2016). Bacteriophages are viruses that target bacteria and enter one of two lifecycles: lysogenic or lytic. Temperate bacteriophages may reside within the bacteria as a lysogen, or quickly replicate and lyse the host in a lytic cycle (Ul Haq et al., 2012). Virulent phages, however, do not possess the genes necessary to carry out lysogeny and are purely lytic (Hepworth et al., 2015). Given the predator:prey relationship of bacteriophage and their host bacteria, diet-induced changes in intestinal environment are likely to drastically alter bacteriophage populations, and vice versa. One study utilizing gnotobiotic mice demonstrated that introduction of specific bacteriophage can induce compositional changes, in both bacteria and bacteriophage populations, shaping the intestinal microbiome and metabolome (Reyes et al., 2013). Bacteriophage populations, collectively known as the phageome, have also displayed diet-induced disturbances (Minot et al., 2011; Howe et al., 2016). However, our understanding of diet-induced longitudinal changes in bacteria:bacteriophage dynamics remains incomplete. Bacteriophages represent an attractive agent for tailoring the gut bacteriome, targeting specific bacteria, leaving other beneficial commensals unaffected. Therefore, it is critical to understand their role in the development and progression of metabolic disease.

In the current study, we explore microbial population dynamics in a mouse model of diet-induced obesity. We collected fecal samples and metabolic readouts from animals across 12 weeks on a chow or high-fat Western Diet (WD) to determine how these populations changed as parameters of metabolic disease developed. Metabolic disruptions were apparent in animals after two weeks of WD feeding while microbial population shifts were detected rapidly with the most dissimilarity seen at 12 weeks. At 12 weeks, bacterial and bacteriophage communities were examined in the context of the metabolic data, revealing correlations between bacteria, phage, and metabolic profiles characteristic of obese or lean. Interestingly, the correlations between most bacteriophage populations and their putative hosts were similar, but this was not always evident. Our study highlights novel connections between diet-induced metabolic changes and constituents of the intestinal microbiome.

## Materials and Methods

### Animals and Diets

Male C57BL/6J mice (*n* = 8 per diet group) from Jackson Laboratories (Bar Harbor, ME) were singly housed in standard microisolator cages at the Veterinary Research Building, College of Veterinary Medicine, Auburn University (Protocol reference number 2014-2547). The room was maintained at an ambient temperature of 22°C ± 2°C on a 12:12 light:dark cycle with zeitgeber time (ZT) 0 representing lights on and ZT12 representing lights off. All experimental procedures were approved by the Auburn University Animal Care and Use Committee. Animals were fed standard rodent chow for 1 week during acclimation to the facility. After which, animals were split into groups receiving either the standard chow diet with tap water (Chow) or a High-Fat Western Diet with tap water (WD). The chow diet (Teklad Global Rodent Diet) contained 24% of calories from protein, 18% from fat, and 58% from carbohydrate. The WD diet was based on the AIN-93G diet and consisted of 44% carbohydrate, 16% protein, and 40% fat, 30% of which was provided from lard, 30% from butterfat, 30% from Crisco, 7% from soybean oil and 3% from corn oil (Reeves, 1997). All dietary groups were given food and water *ad libitum*.

### Metabolic Phenotyping

Promethion Metabolic Mouse Cages (Sable Systems, Las Vegas, NV) were used to house animals for metabolic screening and phenotyping. Animals were transferred from their home cages and singly housed in the metabolic cages at three time points (2, 4 and 12 weeks after diets began). The animals (*n*=8 per treatment group) were singly housed in the cages for 3 consecutive days with the 1^st^ day committed to environment acclimatization and the 2^nd^ and 3^rd^ days for data collection. All animals were returned to their home cage after completion of metabolic phenotyping. Animal activity was measured by the Promethion XYZ Beambreak Activity Monitor. Food, water, and body weight were measured by Promethion precision MM-1 Load Cell sensors. The amount food and water withdrawn from the hoppers was measured and analyzed. The body mass monitors were plastic tubes that also functioned as in-cage enrichment and nesting devices. Water vapor, CO_2_, and O_2_ were analyzed by the Promethion GA-3 gas-analyzer to provide detailed respirometry data. Energy expenditure (EE) was calculated in kilocalories (kcal) by utilizing the Weir equation: 60*(0.003941*VO_2_ (n) +0.001106*VCO_2_ (n)) in which VO_2_ is the oxygen uptake and VCO_2_ is the carbon dioxide output, both of which are measured in ml/min. Respiratory exchange ratio (RER) was determined by measuring gas exchange within the metabolic cages to identify the substrate being primarily utilized for energy within the body. Specifically, RER is the ratio of CO_2_ produced to the volume of O_2_ consumed (RER = VCO_2_/VO_2_) where a RER ~ 0.7 indicates lipid utilization and a RER ~ 1.0 indicates carbohydrate utilization. All metabolic phenotyping data were analyzed using ExpeData software (version 1.8.2; Sable Systems) with Universal Macro Collection (version 10.1.3; Sable Systems).

### Fecal Sample Collection

Collection of fecal matter was performed at the initiation of diet change (Day 0) as well at four other time points following WD feeding: 2 days, 2, 8, and 12 weeks. These timepoints were selected to evaluate microbiome composition prior to WD feeding and to allow for early (2 day and 2 weeks) and late (8 weeks and 12 weeks) profiling of the microbiome prior to the estimated timeline for diet induced obesity (16 weeks). Animals (*n=*8) were removed from their home cages and placed in sterile microisolator cages without bedding for 3 hours. Food and water were provided to the animals during this time. Mice were then returned to resident cages and feces were collected from the sterile cages for DNA extraction and shotgun metagenomic sequencing. Six random mice fecal samples from each treatment group were used to create the 3 samples for microbiome analysis. The comprehensive timeline of the experimental procedures and sampling periods are depicted in **Figure S6** (SMART - Servier Medical ART).

### Tissue Collection and Analysis

Upon completion of the study, all animals were fasted and then sacrificed via CO_2 _asphyxiation and quickly decapitated by guillotine to allow trunk blood collection. Tissues including the liver and visceral (epididymal and retroperitoneal) and subcutaneous (inguinal) white adipose depots were excised and weighed. Final blood glucose was measured by an Accu-Chek blood glucose meter. Serum insulin levels were determined by an insulin ELISA assay (Crystal Chem, Inc., Downers Grove, IL) and data were analyzed for insulin resistance using the HOMA-IR score (HOMA-IR = 26 * fasting serum insulin * fasting blood glucose)/405).

### Shotgun Metagenomic Sequencing-Based Microbiome Profiling

Immediately following collection, DNA was extracted from fecal samples using the Omega E.N.Z.A. Stool DNA kit according to manufacturer’s guidelines. Extracted DNA samples were pooled (2/sample) and shotgun metagenomic sequencing was performed by Hudson Alpha (Huntsville, AL). Whole genome sequencing was performed using an Illumina HiSeq v4 with a 2 x 125 paired-end sequencing 200 million reads. Metagenomic sequences were evaluated for quality using FastX toolkit (FASTX-Toolkit). Raw sequences can be found on the NCBI repository under the BioProject PRJNA730805 ^1^. Low quality sequences (Q-score less than 30) and sequencing adapters were removed using Trimmomatic (Bolger et al., 2014). Sequences were then uploaded to the Metagenomic Rapid Annotation Server (MG-RAST) version 4.0.3 for taxonomic and functional annotation (Meyer et al., 2008). Briefly, sequences were paired, filtered for quality, dereplicated, filtered for host-specific sequences (*Mus musculus*, UMD v3.0), and annotated. Annotated profiles of each sample are publicly available at the MG-RAST repository (MG-RAST Project ID: mgp81921) ^2^. Taxonomic classifications were annotated using the GenBank repository with the minimum cutoff parameters of 1x10^-5^ e-value and alignment length of 15. Density plots, calculated by total annotated hits, were used to set stringent uniform percent identity thresholds while maintaining an accurate measurement of the microbial populations. The percent identity was set to a minimum of 80% identity for bacterial and functional annotations and 70% identity for bacteriophage (Order Caudovirales) annotations. At these parameters, we were able to provide a conservative estimate of taxa while excluding ambiguous sequences. Current databases are not complete for viral genera classification due to variations in taxonomic classification strategies. Therefore, species within the order Caudovirales, as annotated by MG-RAST, were cross-referenced with the International Committee on Taxonomy of Viruses taxonomic database or other documentation detailing classification of the bacteriophage (International Committee on Taxonomy of Viruses. and King, 2011)^3^ (**Table S8**). From this, we were able to update viral species into current taxonomic genera in order to describe the types of bacteriophage present and how they fluctuate following dietary change. Descriptions of genera present in our samples are outlined in **Appendix 1**. Taxonomic classification hits were then normalized based on the total hit count. Rarefaction curves depicting alpha diversity were generated using the MG-RAST server.

### Statistical Analysis

Final body and tissue weights along with serum measures were analyzed using a one-way ANOVA with a Newman-Keuls* post-hoc* test. The percent body weight change and 24-hour cycle data were assessed by a repeated measures two-way ANOVA so that animals in one diet group could be compared with animals in ;another diet group across dietary weeks or circadian time points The above statistical analyses were performed using SigmaPlot with significance determined at p < 0.05. The National Mouse Metabolic Phenotyping Centers (MMPC) Energy Expenditure analysis page ^4^ was used for multiple linear regression analysis (ANCOVA) to assess body weight as a covariate on energy expenditure with significance determined at p < 0.05. Significance for all measures was determined at p < 0.05 and all data are presented as Mean ± SE.

### Microbiome Statistical Analysis

Three pooled samples for each diet group at each collection point were used to calculate changes of relative abundance in the microbiome. Relative abundance was used to calculate means and standard deviations of each treatment groups at each time point using the statistical program GraphPad Prism v4. Using the R studio statistical platform (R Core Team, 2014), t-tests were performed to identify significant difference in relative abundance of microbial taxa. Non-metric multidimensional scaling (n*MDS*) ordination was generated in R studio using the *vegan* package (Dixon, 2003). To generate the nMDS, raw bacterial hits were used to compute a sample dissimilarity matrix using the Bray-Curtis dissimilarity index. This matrix was then used to compute an ordination of the samples in two dimensions. The *vegan* package was also used to calculate Shannon’s Diversity Index scores. Then, the Pielou’s Evenness Index was calculated by dividing the Shannon’s Diversity Index score by the log of unique species amount. Mann-Kendall Trend tests were performed on diversity and evenness scores separately using the *randtest* package (Pohlert, 2018). Pearson correlation coefficients were calculated using relative abundance and metabolic readouts at 12 weeks following dietary exposure in R studio using the package *psych* (Revelle, 2017). Pearson correlation plots were generated in R studio using the package *ggcorplot* (Kassambara, 2015) using a correlation coefficient threshold of an absolute value of 0.6.

## Results

### Diet-Induced Obesity Pathophysiology

**Figure 1:**
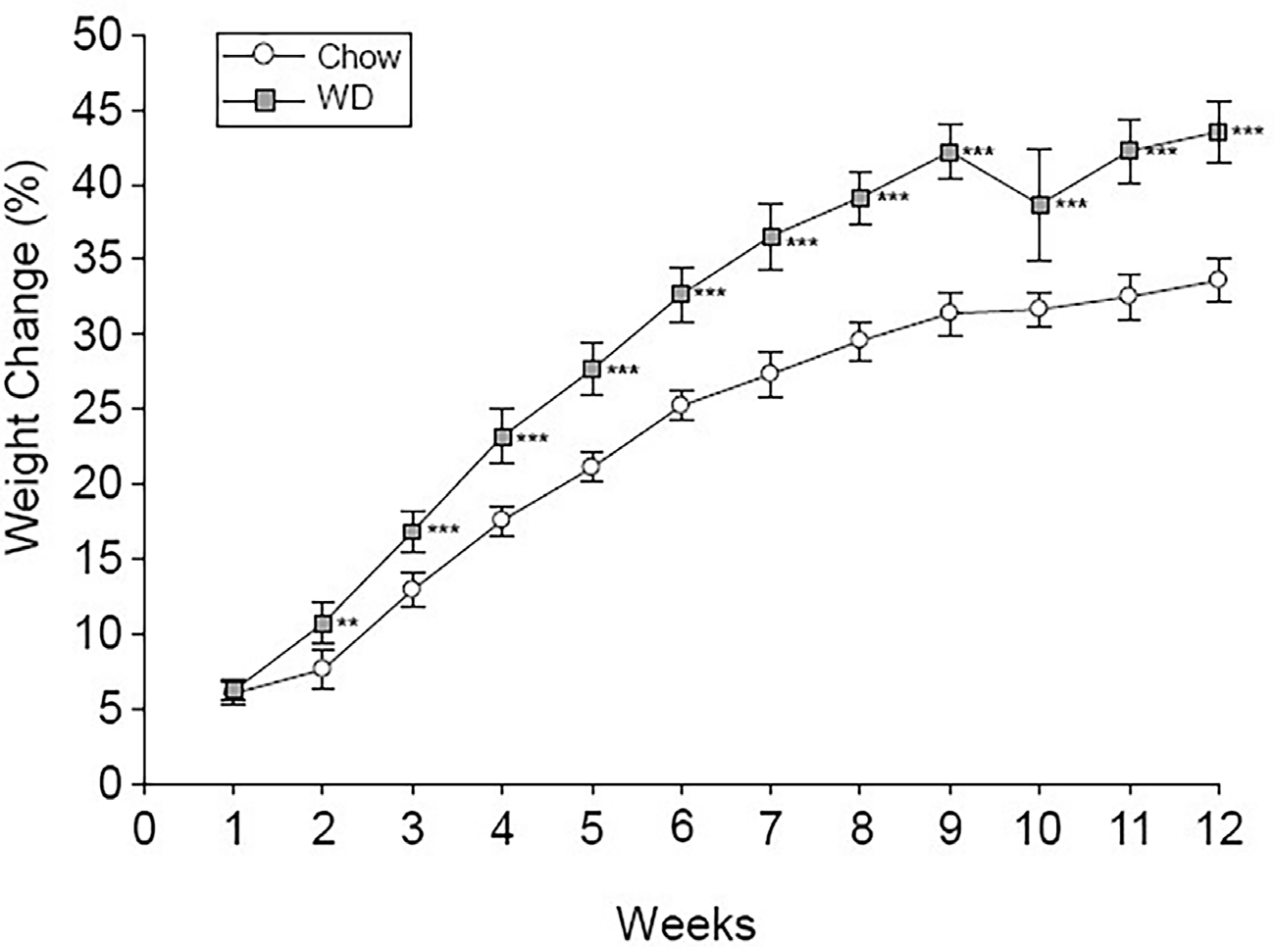
Percent body weight change. Weekly percent weight change over time is shown for the Chow and WD groups. Group differences over the course of dietary treatment were analyzed by ANOVA. All data points are shown as group mean ± SE. (**p < 0.01, ***p < 0.001 compared to Chow).

Body weight was tracked over the course of the 12 week experiment (**Figure 1**). Prior to the experiment, the average body mass of the chow group was 20.9 +/- 1.8 g and the WD group was 20.8 +/- 1.7 g. By week 2 on different diets, animals fed the WD had a significantly higher percent body weight change compared to chow (p < 0.01) and persisting throughout the duration of the 12 week experiment (p < 0.001). The change in body weight was mainly due to an increase in body fat as the WD-fed group had significantly heavier visceral and subcutaneous (Sub-Q) fat pad weights at the 12 week endpoint of the experiment (**Table 1**, visceral: p < 0.001; Sub-Q: p < 0.01). There was no effect of WD on fasting blood glucose by 12 weeks, yet serum insulin levels were significantly elevated in the WD-fed group (**Table 1**, p < 0.01). Insulin resistance, as calculated using the Homeostatic Model Assessment of Insulin Resistance (HOMA-IR) method, revealed insulin resistance in the WD-fed animals compared to the Chow-fed animals also at 12 weeks (**Table 1**, p < 0.01).

**Table 1:** Final body weight, tissue weights normalized to body weight and serum measures for the three dietary groups.

	Chow	WD
Final Body (g)	27.4 ± 0.426^a^	39.8 ± 1.58^b^
Normalized eWAT (g)	0.024 ± 0.002^a^	0.060 ± 0.004^b^
Normalized rWAT (g)	0.006 ± 0.001^a^	0.017 ± 0.003^b^
Normalized iWAT (g)	0.009 ± 0.0004^a^	0.026 ± 0.004^b^
Normalized Liver (g)	0.046 ± 0.003	0.048 ± 0.002
Insulin (ng/mL)	0.814 ± 0.246^a^	2.27 ± 0.367^a^
Glucose (mg/dL)	164 ± 11.4	171 ± 14.7
HOMA-IR	8.84 ± 2.71^a^	26.2 ± 4.82^a^

Data are presented as mean ± SE. Differing superscript letters indicate differences between dietary conditions P<0.05.

### Metabolic Rhythm and Flexibility

**Figure 2:**
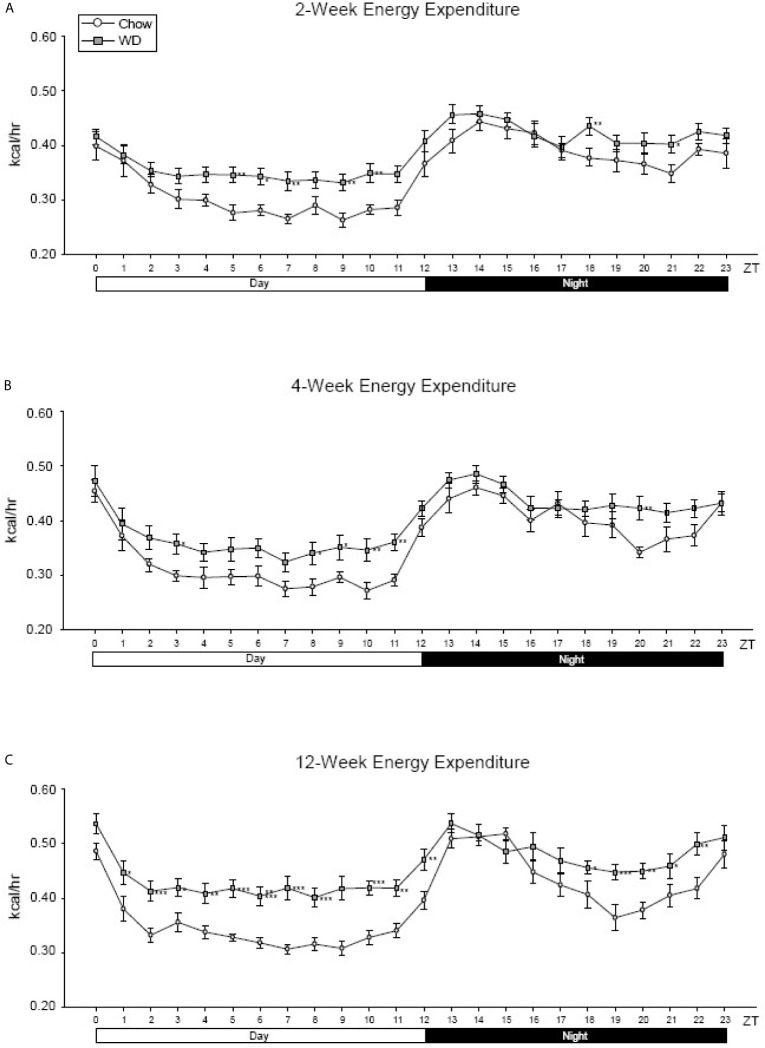
Energy expenditure at 2-, 4- and 12-weeks. **(A)** Mean circadian analysis of energy expenditure at each hour in the 24-hour cycle in the Chow and WD groups after 2-weeks of dietary exposure. **(B)** Mean circadian analysis of energy expenditure at each hour in the 24-hour cycle in the Chow and WD groups after 4-weeks of dietary exposure. **(C)** Mean circadian analysis of energy expenditure at each hour in the 24-hour cycle in the Chow and WD groups after 12-weeks of dietary exposure. All data points are shown as group mean ± SE. (*p < 0.05, **p < 0.01, ***p < 0.001 compared to Chow).

To examine how diet impacted average energy expenditure (EE) within the light and dark phases, we measured EE at each ZT over a 24-hour cycle. Although diurnal rhythmicity was observed in both dietary groups, the WD induced significant disruptions evident as early as 2 weeks. The WD-fed group exhibited elevated average EE when compared to Chow-fed group during the inactive (day) period from ZT4-ZT11 (**Figure 2A**, p < 0.01). At 4 weeks, the WD-fed group continued to demonstrate elevated average EE during the day, specifically at ZT3 and ZT8-11 (**Figure 2B**, p < 0.05), as well as ZT20 (**Figure 2B**, p < 0.01). By 12 weeks, the WD-fed group exhibited significantly elevated average EE for the entirety of the day cycle (**Figure 2C**, p < 0.01). Additionally, we observed a dip in the average EE in from ZT18-22, which was not as pronounced in the WD-fed group. Consequently, the EE of the WD-fed group was significantly elevated during that time (**Figure 2**, p < 0.05).

**Figure 3:**
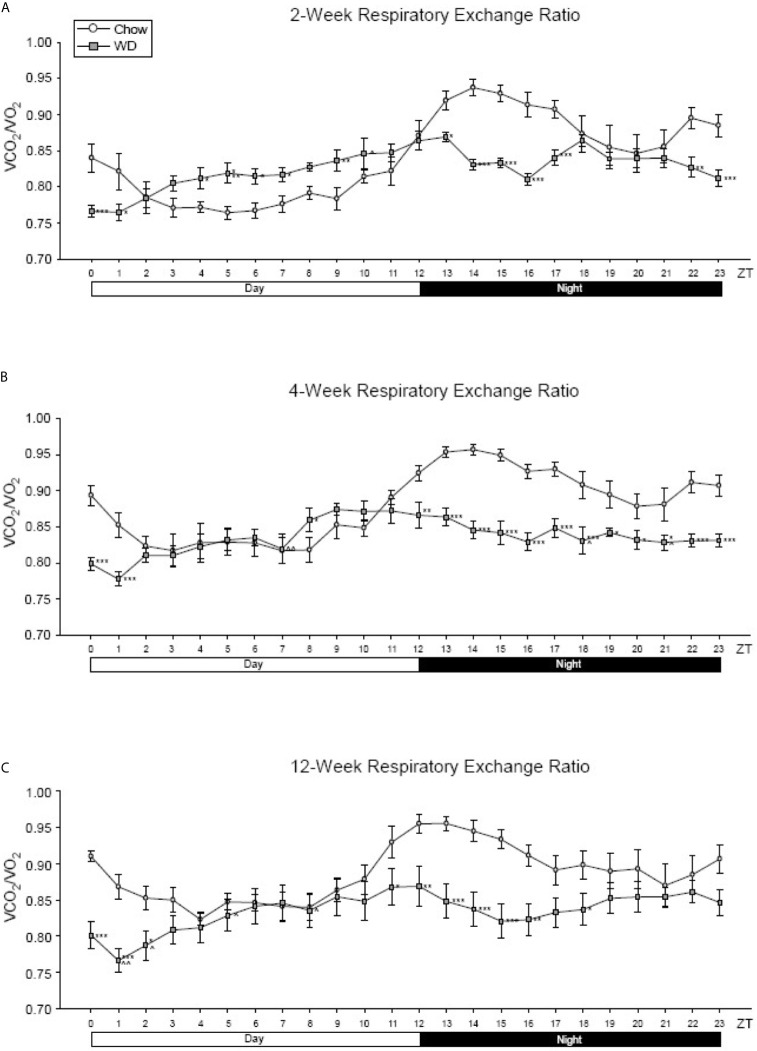
Respiratory exchange ratio at 2-, 4- and 12-weeks. **(A)** Mean circadian analysis of respiratory equivalent ratio at each hour in the 24-hour cycle in the Chow and WD groups after 2-weeks of dietary exposure. **(B)** Mean circadian analysis of respiratory equivalent ratio at each hour in the 24-hour cycle in the Chow and WD groups after 4-weeks of dietary exposure. **(C)** Mean circadian analysis of respiratory equivalent ratio at each hour in the 24-hour cycle in the Chow and WD groups after 12-weeks of dietary exposure. All data points are shown as group mean ± SE. (*p < 0.05, **p < 0.01, ***p < 0.001 compared to Chow).

Across all three experimental time points, a diurnal rhythm was observed in the respiratory exchange ratio (RER) of Chow-fed mice: greater metabolism of lipids during the inactive, day phase and more carbohydrate utilization during the active, night phase (**Figures 3A–C**). At week 2 and persisting through week 12, this rhythm of metabolic flexibility was significantly dampened in the WD-fed group with near constant lipid utilization across the time points. At 2 weeks, WD-fed animals demonstrated a significantly elevated RER from ZT4-ZT7 and a significantly decreased RER from ZT13-17 and ZT22-ZT24 (**Figure 3A**, p < 0.05). Four weeks after diets began, RER in the WD-fed group was significantly decreased from Chow-fed animals starting at ZT12 and continuing to ZT24 (**Figure 3B**, p < 0.05). Lastly, after 12 weeks of dietary exposure, we observed results similar to week 4 during ZT13-ZT18 with the WD-fed animals exhibiting a significantly decreased RER (**Figure 3C**, p <0.05). These data suggest that the WD significantly impacted metabolic diurnal rhythms as well as metabolic flexibility. These effects were observed as early as 2 weeks and persisted through 4 and 12 weeks of WD feeding.

### Diurnal Activity and Feeding Behavior

We observed a typical murine diurnal rhythm in our animals with elevated activity during the night phase and decreased activity during the day (**Figures S1A–C**). Diet did not appear to have an effect on activity at the 2 week time point (**Figure S1A**). Similarly, 4 weeks of WD consumption did not induce large-scale changes in diurnal activity (**Figure S1B**). However, at the 12 week experimental time point, we observed a significant reduction activity in the WD-fed group from ZT13-ZT15 (**Figure S1C**, p < 0.05). However, this did not result in a significant decrease in activity when averaged across the entire night cycle.

After 2 weeks on the diets, the WD-fed group consumed significantly more food and water by weight than the Chow-fed group during the day (**Figure S2A**, p < 0.05). Kilocalorie consumption was found to be significantly greater in the WD-fed group during all three time points (**Figure S2A**, p < 0.05). At the 4 week time point, the WD-fed group consumed more grams of food during the day, but less food during the night than the Chow-fed groups (**Figure S2B**, p < 0.01). This translated into greater kilocalorie consumption in the WD-fed animals during the day and total (**Figure S2B**, p < 0.01). Twelve weeks after diets began, the WD-fed group did not consume more food by weight than the Chow-fed animals. However, kcal consumption was significantly elevated in the WD-fed group compared to Chow-fed group for the day, night and 24-hour total data points due to caloric density of the food (**Figure S2C**, p < 0.05). There was not a significant difference in water consumption between the Chow-fed and WD-fed groups at 4 or 12 weeks.

### Diet-Induced Changes in the Enteric Microbiota

To identify WD-induced changes in the enteric microbiota, we used shotgun metagenomic sequencing of freshly isolated feces at each indicated timepoint. First, to ensure that we had adequate depth of sequencing, estimates of alpha diversity and depth of sampling were assessed using rarefaction curves (**Figures S3A–E**). Each sample at each time point reached a plateau, (indicating more sequences than OTUs) signifying adequate depth of sampling and alpha diversity.

**Figure 4:**
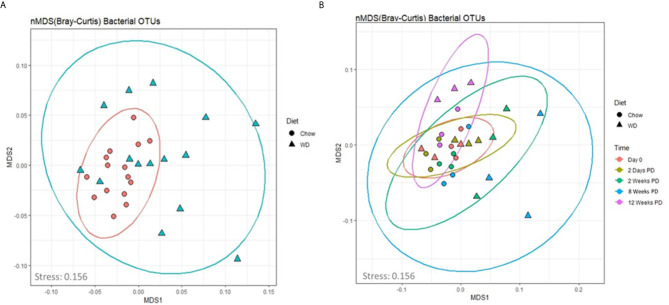
An nMDS ordination of the microbiota samples **(A)** by diet and **(B)** over time. The taxonomic profiles of the samples were used to compute the sample dissimilarity matrix using Bray-Curtis dissimilarity index. The matrix was used to compute an ordination of the samples in two dimensions (MDS1 and MDS2). The stress associated with this ordination is 0.156. The shapes in plot B denote the diet (Chow and WD), where the color denotes the time point (0 days, 2 days and 2, 8 and 12 weeks on the diets).

We next assessed global changes in the enteric microbiome in the WD-fed vs. Chow-fed mice. To achieve this, we analyzed the composition of the enteric microbiota over time by means of non-metric dimensional scaling (nMDS) ordination plots based on the Bray-Curtis dissimilarity index (**Figure 4**). This method provides a similar visualization of the data as a principle component analysis (PCA) without the biases associated with a PCA concerning low abundance organisms (Zhu and Yu, 2009). The fit or stress of the nMDS denotes how well the ordination consolidates the observed distances among the samples where a value less than 0.3 would indicate a good fit. The fit associated with our analysis was 0.156. In **Figure 4A**, the nMDS ordination plot factored by diet revealed one predominant central cluster (Chow diet) with the other diet (WD) scattered in a radiating pattern, indicating increasing dissimilarity. Thus, the composition of the microbiome was much more stable in the chow group over time, than in the WD group. To examine how dissimilarity progressed over time between the two diet groups, an nMDS ordination plot factored by diet and time was constructed (**Figure 4B**). This analysis highlighted the progression of increasing changes within the intestinal microbiome over time in those animals fed a WD (**Figure 4B**, **Figure S5**). This trend of increasing dissimilarity continued throughout the duration of the experiment in which greater body weight and metabolic changes overtime were observed between the WD- and Chow-fed mice. We also observed a significant decrease in bacterial diversity and evenness in the WD-fed group compared to Chow-fed group, primarily at 12 weeks (*p* = 0.006; *p* = 0.008) (**Figures S4A, B** and **Tables S6, S7**). Using Pearson’s correlation we also noted that bacterial diversity and evenness were inversely related to parameters that increased during the development of obesity including HOMA-IR, body weight, visceral fat weight, and Day EE (**Figure S4E** and **Table S10**). In summary, decreases in enteric bacterial diversity and evenness, indicators of dysbiosis, were observed in mice fed a WD and correlated strongly with the metabolic phenotype of obesity.

**Figure 5:**
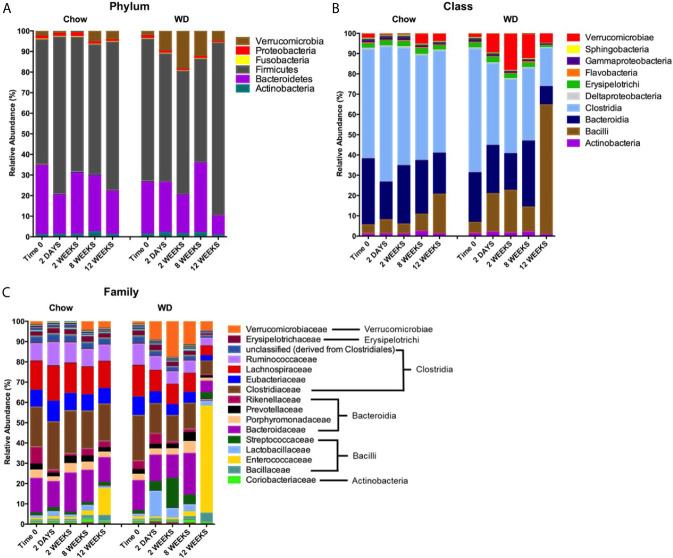
Changes in relative abundance of microbial composition after administration of the Western diet. **(A)** Phylum, **(B)** Class and **(C)** Family level bacterial composition in mice fed Chow or WD after 2 days, 2, 8 and 12 weeks of dietary exposure. The mean relative abundance (%) of bacterial phyla are shown. Statistical results outlined in **Tables S1**–**S3**.

Our next goal was to determine which microbial populations most significantly contributed to the dissimilarity seen in WD-associated microbiota. Relative abundance plots illustrate the composition of the microbiota in each group over time (**Figure 5A** and **Table S1**) (Shreiner et al., 2015). Prominent in the Chow-fed group were members of four well known bacterial phyla: Firmicutes (60 ± 8.5%), Bacteroidetes (33.460 ± 7.1%), Proteobacteria (1.675 ± 0.2%), and Actinobacteria (1.012 ± 0.2%), at day 0, consistent with previous reports (David et al., 2014; Ziętak et al., 2016). The microbial composition remained relatively stable throughout the 12 week experiment for mice fed a Chow diet. On the other hand, shifts in relative abundance profiles of WD-fed mice samples were detected as early as day 2 (**Figure 5A** and **Table S1**). For example, Proteobacteria showed a significant reduction in abundance in the WD-fed group vs. Chow-fed group (*p* = 0.02), while Verrucomicrobia began to increase after 2 days from 1.467 ± 2.4% to 9.182 ± 4.6% in the WD-fed group. The increase in Verrucomicrobia abundance was transient and returned to pre-diet abundance levels by 12 weeks. Notably, significant increases were observed in Firmicutes (*p =* 0.04) at 12 weeks in the WD group (**Figure 5A** and **Table S1**).

To more precisely resolve shifts in bacterial communities, we next evaluated class-level and family-level community profiles (**Figures 5B, C** and **Tables S2, S3**). The composition of the bacterial classes and families in the Chow-fed group were relatively stable throughout the experiment (**Figures 5B, C** and **Tables S2, S3**). In contrast, we observed significant changes in bacterial abundance/composition in the WD-fed group over the experimental timeline.

Most notable among the WD-induced changes was an increase in class Bacilli at 12 weeks (*p* = 0.04). Bacilli have been previously shown to be elevated in the intestinal microbiota in obese individuals (Ziętak et al., 2016). Enterococcaceae, specifically the Genera *Enterococcus*, appeared to be the major contributor to this increase at 12 weeks in the WD group (*p* = 0.04). Additionally, the increase seen in the class Bacilli was also due to a bloom in Streptococcaceae abundance at 2 weeks (*p* = 0.005), 8 weeks (*p* =0.01) and 12 weeks (*p* =0.02). Other notable increases in bacterial composition included Verrucomicrobiae. Starting as early as 2 days, we observed an increase in the class Verrucomicrobiae in the WD-fed group that peaked at 2 weeks and returned to roughly baseline levels by 12 weeks (**Figure 5B** and **Table S2**). This increase could be attributed to an increase in the family Verrucomicrobiaceae (**Figure 5C** and **Table S3**), primarily from the genera *Akkermansia* (data not shown).

We also observed decreases in class Clostridia starting at 2 weeks and becoming significant at 12 weeks (*p* = 0.01) (**Figure 5B** and **Table S2**). These decreases were accounted for primarily by members of the family Clostridiaceae beginning at 2 days on WD (*p* = 0.008) (**Figure 5C** and **Table S3**). Other significant decreases in the WD-fed group were observed in class Bacteroidia at 2 weeks (*p* = 0.04) and 12 weeks (*p* = 0.0007) and many members of the phylum Proteobacteria. Together, the reduction of overall diversity and evenness within the intestinal microbiota could be attributed to over-growth of Bacilli and Verrucomicrobiae family members along with reductions in Clostridiaceae and several other bacterial constituents on WD.

### Diet-Induced Changes in the Enteric Phageome

We next wanted to determine how other constituents of the microbiome, namely bacteriophage populations, were impacted by the WD. First, we assessed the degree of the dissimilarity by analyzing bacteriophage diversity and evenness (**Figures S4B, D** and **Table S6**). Similar to the trends observed in bacterial populations, we observed a reduction in the diversity and evenness of the enteric phageome over the course of the experiment in WD-fed mice. The Mann-Kendall trend analysis revealed a downward trend over the course of the experiment in both diversity (S = -65, p = 0.008) and evenness (S = -75, p = 0.003) in WD-fed groups (**Table S7**). Moreover, the reduction in the diversity and evenness of the enteric phageome preceded similar changes in bacteria following dietary exposure (**Figures S4A, B**). Bacteriophage diversity and evenness were also inversely related to metabolic parameters that increased during obesity development, specifically HOMA-IR, body weight, visceral fat weight, and Day EE (**Figure S4E** and **Table S11**). Thus, changes in bacteriophage populations preceded changes in bacterial composition, but both had similar overall trends of decreasing diversity on WD. Also, both changes correlated with changes in metabolic phenotype.

**Figure 6:**
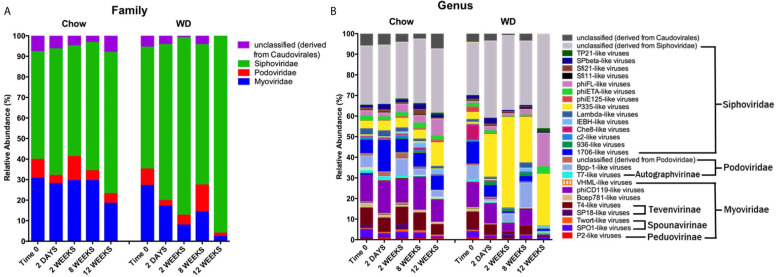
Changes in relative abundance of bacteriophage composition after administration of the Western diet. **(A)** Family and **(B)** Genus level viral composition derived from the order Caudovirales in mice fed Chow or WD at 2 days, 2, 8 and 12 weeks of dietary exposure. Data shown are based on those families belonging to the order Caudovirales. The mean relative abundances (%) of viral families are shown. Statistical results outlined in **Tables S4**, **S5**.

We next evaluated the relative abundances of bacteriophage at the family and genus levels. It is important to note here that phage taxonomy classifications are not as complete as those for bacteria, especially at the genus level (Kuhn, 2020). Thus, many of the bacteriophages in our analysis remain unclassified beyond the family annotation. The enteric phageome of Chow-fed mice remained relatively consistent throughout the experiment at the family and genus levels (**Figures 6A, B**). In contrast, mice fed a WD demonstrated an increase in Siphoviridae at 2 weeks (*p* = 0.0001) coupled with a reduction in Myoviridae at 2 weeks (*p* = 0.001) and 8 weeks (*p* = 0.05), and Podoviridae as early as day 2 (**Figure 6A** and **Table S4**). The increase in Siphoviridae in the WD-fed group could be largely attributed to increases in *P335-like viruses* starting at 2 days, which were maintained at elevated abundance through 12 weeks (**Figure 6B** and **Table S5**), with significant increases at 2 weeks (*p* = 1.78 x 10^-5^) and 8 weeks (*p* =0.0001). Additionally, we observed an increase in *phiFL-like viruses* at 12 weeks. *phiFL-like viruses* are primarily temperate bacteriophage that target members of the Enterococcaceae family (Adriaenssens et al., 2015), and this increase could be attributed to an increase in host species containing a prophage (**Table S8**).

Myoviridae family abundance decreased significantly as early as 2 weeks and continued to decline in WD-fed mice at 12 weeks (2.297 ± 0.5%) (**Figure 6A** and **Table S4**). This reduction was due in part to a significant depletion of the bacteriophage genus *phiCD119-like viruses* at 2 weeks (*p* = 0.004) in WD-fed mice (**Figure 6B** and **Table S5**). Though not significant, *phiCD119-like viruses* remained reduced in relative abundance in the WD-fed group at 8 weeks and 12 weeks. Taken together, WD feeding increased the abundance of the Siphoviridae family and reduced abundance in the Myoviridae family compared to Chow-fed mice, further, WD-induced alterations in the enteric phageome occurred as early as 2 days on diet (**Figure 6B** and **Table S5**), preceding changes in their bacterial host abundance (**Figure 5B**).

### Correlations Between Diet-Induced Changes in the Bacterial and Bacteriophage Consortia

**Figure 7:**
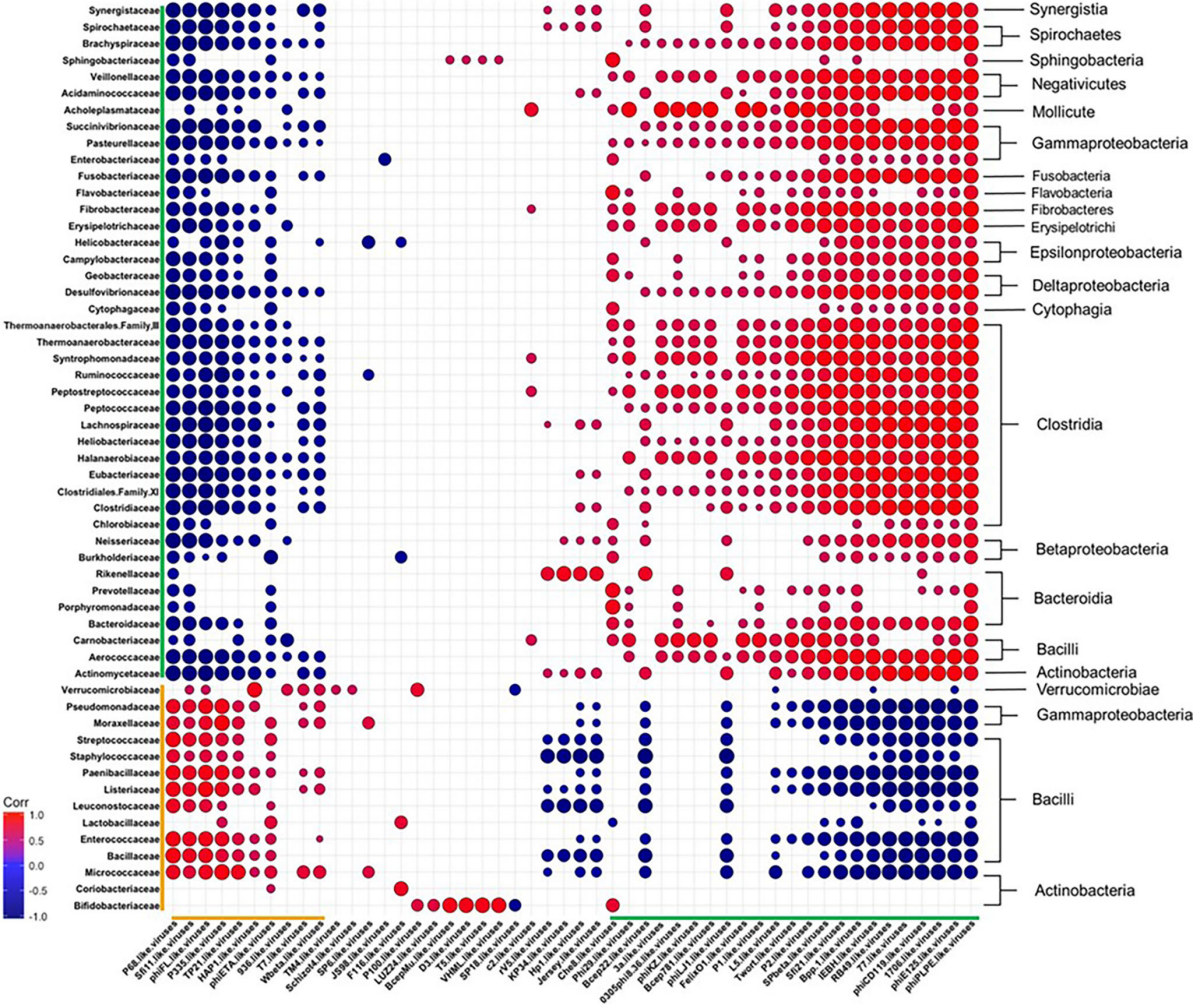
Diet Induced Bacteria-Bacteriophage Patterns in Obesity. Pearson’s correlation plot of the top 55 most abundant Bacterial Families and all Bacteriophage Genera (Viral OTUs within the order Caudovirales) for mice fed Chow or WD at 12 weeks. Positive values (red circles) indicate positive correlation coefficients above 0.6, and negative values (blue circles) indicate inverse correlation coefficients below -0.6. The size and shading of the circles indicate the magnitude of the correlation, where darker shades indicate a stronger correlation than lighter shades. Organization of the plot is based on the correlation coefficient values of Brachyspiraceae. Correlation coefficient values outside of the threshold of 0.6 are not included in this plot, but are outlined in **Table S9**. Putative host information and correlation coefficient values between bacteriophage genera and putative bacterial host family are listed in **Table S8**.

To obtain a more comprehensive picture of how diet affects the relationship between specific phage genera and bacterial families, we calculated Pearson correlation coefficients between the Caudovirales genera and the 55 most abundant bacterial families at the final time point of 12 weeks (**Figure 7** and **Table S9**). The correlation coefficients were sorted into negatively and positively correlated groups which resulted in: 1) a large group of bacteria that correlated positively with a large group of phages (both highlighted with green brackets) and 2) a smaller group of bacteria and bacteriophage that were positively correlated with each other (orange brackets). We observed members within the designated groups had the same trend in response to diet. For example, members within the green group were often reported as reduced in abundance following WD-feeding and the members of the orange group were often seen to bloom following WD-feeding. The two groups also had conversely negative correlations with members of the other (green vs. orange) group. This correlation grouping resulted in separation of bacterial families, most of which clustered with other families belonging to the same class. Given these findings, we next determined the frequency of putative phage-host relationships between the positively correlated groups.

Among the organisms within the green brackets, roughly 48% had positive correlations between bacteriophage genera and their putative bacterial hosts (**Figure 7** and **Tables S8, S9**). Six of these were very strong correlations of 0.6 or higher. These include bacteriophage genera who target bacterial families Pasteurellaceae (*phiPLPE-like viruses*) (Leblanc et al., 2009; Comeau et al., 2012), Burkholderiaceae (*Bpp-1-* and *phiE125-like viruses*) (Summer et al., 2007), Clostridiaceae (*phiCD119-like viruses*) (Revathi et al., 2011; Sekulovic et al., 2014; Hargreaves and Clokie, 2015) and Enterobacteriaceae (*Che8-* and *RB49-like viruses*) (International Committee on Taxonomy of Viruses (ICTV); Petrov et al., 2010; Hatfull, 2012). Many of the bacteriophage in these genera were temperate. *phiCD119-like viruses*, for example, are temperate bacteriophage that target *Clostridium* species as their host (International Committee on Taxonomy of Viruses (ICTV); Revathi et al., 2011; Sekulovic et al., 2014; Hargreaves and Clokie, 2015). Temperate bacteriophage, such as *phiCD119-like viruses*, would correlate directly with bacterial host abundance if they are present as integrated prophage. Also in the organisms within the green brackets were 7 bacteriophage genera that negatively correlated with their putative host (correlation coefficients between 0.4 and 0.6; **Table S8, S9**). Interestingly, bacteriophage genera with the strongest negative correlations in this grouping targeted either members of Bacillaceae (McLaughlin et al., 1986; Grose et al., 2014), Streptococcaceae (Garneau et al., 2008; Guglielmotti et al., 2009) or Staphylococcaceae (Goerke et al., 2009) (**Table S8**). For example, Streptococcaceae-targeting bacteriophage genera *1706-* and *Sfi21-like viruses* were strongly negatively correlated with their host, Streptococcaceae (International Committee on Taxonomy of Viruses (ICTV); Garneau et al., 2008; Guglielmotti et al., 2009).

While the orange group contained fewer bacterial families and bacteriophage genera than the green group (**Figure 7** and **Table S8, S9**), the majority of bacteriophage and putative hosts in this group showed strong positive correlations. These include Streptococcaceae-targeting *P68-* (Deghorain and Van Melderen, 2012)*, Sfi11-* (Deveau et al., 2008; Guglielmotti et al., 2009; Ali et al., 2014), and *P335-like viruses* (Labrie and Moineau, 2000; Samson and Moineau, 2010; International Committee on Taxonomy of Viruses and King, 2011), Staphylococcaceae-targeting *P68-* (Nelson et al., 2003) *and phiETA-like viruses* (Bae et al., 2006; Daniel et al., 2007; International Committee on Taxonomy of Viruses and King, 2011; Bueno et al., 2012), Bacillaceae-targeting *TP21-like viruses* (Klumpp et al., 2010) and Enterococcaceae-targeting *phiFL-like viruses* (Adriaenssens et al., 2015). For example, *P335-like viruses* were seen to strongly correlate with their bacterial host, Streptococcaceae. This genus is comprised of an equivalent amount of virulent and temperate bacteriophage species. Thus, the elevation in abundance of this bacteriophage genera cannot be solely due to replication of prophage *via* host replication. Taken together, phage abundance positively correlated with the abundance of their putative host bacteria in many cases, but not all.

### Correlations Between WD Induced Metabolic Phenotype and the Intestinal Bacteria and Bacteriophage Populations

**Figure 8:**
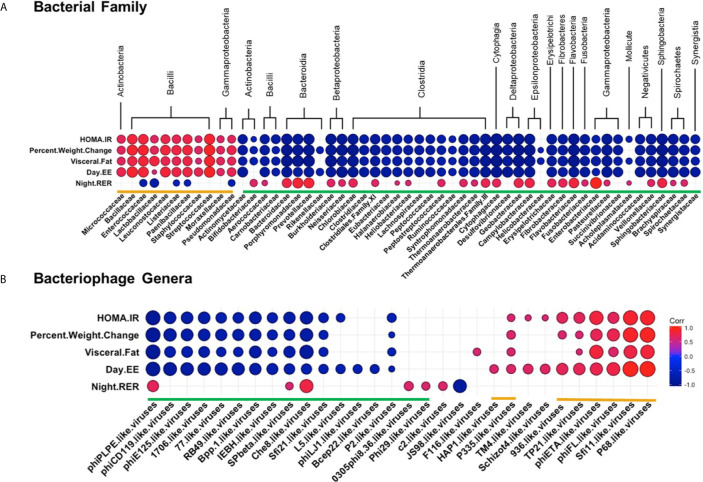
Diet Induced Bacteria/Bacteriophage – Metabolic Patterns in Obesity. Pearson’s correlation plot of **(A)** Bacterial Families (top 55 most abundant) or **(B)** Bacteriophage Genera (Viral OTUs within the order Caudovirales) and metabolic parameters for mice fed Chow or WD for data after 12 weeks of dietary exposure. Statistical significance was determined for all pairwise comparisons. Positive values (red circles) indicate positive correlation coefficients above 0.6, and negative values (blue circles) indicate inverse correlation coefficients below -0.6. The size and shading of the circles indicate the magnitude of the correlation, where darker shades indicate a stronger correlation than lighter shades. Correlation coefficient values outside of the threshold of 0.6 are not included in this plot (**Tables S10**, **S11**).

Next, we examined how specific bacterial families correlated with metabolic phenotypes within the WD-fed group. Interestingly, bacterial families clustered into similar groupings as those generated by correlations of bacteria and bacteriophage (again designated by orange or green brackets, **Figure 8A** and **Table S10**). Bacterial families grouped in the orange cluster positively correlated with metabolic parameters shown to be indicative of metabolic disease, namely HOMA-IR, Percent Body Weight Change, Visceral Fat Weight, and Day EE. Conversely, this orange group of bacterial families showed a strong inverse relationship with Night RER. As mentioned previously, a lower night RER (close to 0.7) during the active cycle would indicate primarily lipid utilization, characteristic of metabolic inflexibility. These results indicate that bacterial families within the orange group positively correlate with key pathophysiological metabolic parameters associated with obesity. Thus, our findings suggest that bacterial families within the orange group represent an obesity-responsive group of bacteria.

Correlation profiles showing a negative relationship with metabolic parameters associated with obesity were apparent for the bacterial families within the green grouping. Bacterial families within the green grouping negatively correlated with HOMA-IR, Percent Body Weight Change, Visceral fat weight, and Day EE and were most often positively correlated with Night RER. Hence, bacterial families grouped in green negatively correlate with key pathophysiological metabolic parameters associated with obesity.

Because strong relationships were identified between bacterial families and metabolic phenotypes, we sought to examine the relationships between bacteriophage populations in the intestinal microbiome and the metabolic phenotype observed in the WD-fed animals. Many of the bacteriophage genera that showed strong correlations to metabolic readouts correlated similarly in terms of green and orange grouped bacteriophage genera (**Figure 8B** and **Table S11**). Orange labeled bacteriophage genera positively correlated with HOMA-IR, Percent Body Weight Change, Visceral Fat Weight, and Day EE. As these particular bacteriophage genera increased in relative abundance, phenotypic markers for body weight gain increased following dietary exposure. In contrast, green labeled bacteriophage genera had a negative relationship with the metabolic parameters mentioned above, revealing that as these metabolic parameters increased, there was a decrease in the relative abundance of the bacteriophage genera in the green group (**Figure 8B** and **Table S11**). Because of the linked relationship between the bacteriophage genera and their host, correlations between bacteriophage and metabolic parameters must be considered within the framework of bacterial host dynamics.

## Discussion

In this study, we utilized an integrative approach of metabolic readouts coupled with microbiome analysis to comprehensively explore the relationship between diet, host metabolic changes and constituents of the gut microbiota. Extending the scope of previous reports (Turnbaugh et al., 2006; Turnbaugh et al., 2009; David et al., 2014; Hakkak et al., 2017), we confirm that WD-feeding adversely effects a wide range of communities within the intestinal microbiome using shotgun metagenomic sequencing. More importantly, we now describe novel relationships between specific microbiome constituents and pathophysiological metabolic parameters associated with obesity. We report dynamic alterations to the composition of the intestinal microbiota as early as 2 days following WD feeding (**Figures 5** and **6**). Over time, consortia of bacteria and bacteriophages became progressively less diverse and even (**Figure S4**). Furthermore, fluctuations in the phageome were quite rapid, often preceding changes in abundance of their bacterial host. After 12 weeks of WD-feeding, constituents of the enteric microbiome, namely bacteria and bacteriophages, showed distinct correlation patterns between bacteria and bacteriophage with the metabolic phenotype, which allowed us to distinguish between a healthy microbiome and one more typical of obesity (**Figures 7** and **8**).

To characterize metabolic changes over the course of the induction of obesity we examined energy expenditure, substrate utilization (RER), food intake, and activity parameters. The metabolic and rhythmic disruptions in EE and RER were observed throughout the time course of the experiment (**Figures 2** and **S1**). In contrast, we observed a rhythmic disruption in food uptake (greater food intake in WD-fed mice in the day) early in the time course of the experiment while rhythmic disruption in activity (a reduction in WD-fed mice at night) was observed late in the time course of the experiment. These findings are consistent with our prior findings and those from others (Kohsaka et al., 2007; Hatori et al., 2012; Eckel-Mahan et al., 2013; Luo et al., 2016; Woodie et al., 2018). Thus, our WD feeding paradigm resulted in metabolic and rhythmic disruptions similar to those previously published in diet-induced obese mice.

One prominent example of a rapid microbial change in WD-fed animals was the transient rise in Verrucomicrobia which peaked at 2 weeks and declined to levels comparable to Chow-fed counterparts by 12 weeks (**Figure 5**). This spike in Verrucomicrobia was mainly attributed to the genera *Akkermansia*, which has been shown to play a protective role during obesity (Everard et al., 2013; Hakkak et al., 2017). In fact, administration of *Akkermansia muciniphila* during diet-induced obesity has been shown to improve the metabolic profile and colonic mucus layer thickness (Everard et al., 2013). Perhaps these organisms aid in short-term protection from intestinal microbiota dysbiosis or bloom simply due to nutrient availability following dietary exposure (Cani, 2018). Nevertheless, *Akkermansia* spp. were unable to maintain increased abundance after 8 weeks PD in our study (**Figure 5**).

At 12 weeks PD, we observed a depletion of Bacteroidetes and domination by Firmicutes in WD-fed mice (**Figure 5**). The disproportionality of Bacteroidetes : Firmicutes has been previously associated with the obese phenotype of the intestinal microbiota (Duncan et al., 2008; Howe et al., 2016). Although changes in the microbiota occurred rapidly, the shift in Bacteriodetes : Firmicutes ratio did not happen until later in our study, at 12 weeks. Instead, we observed diet-induced fluctuations in several other phyla that preceded the well-documented B:F ratio (Duncan et al., 2008; Howe et al., 2016; Hakkak et al., 2017). Further, while small changes were observed in bacterial populations at phylum level, more dynamic shifts were detectable at the class and family levels as early as 2 days PD in WD-fed mice. The WD-fed group demonstrated a shift in bacterial profile to one dominated by Bacilli in contrast to the Chow-fed group which was dominated by Clostridia and Bacteroidia. Not only have we observed changes that are consistent with existing studies of the bacteriome in obesity (Backhed et al., 2004; Hakkak et al., 2017), but we also bring into context changes in the phageome that occur in tandem with mammalian host metabolic changes.

The rapid changes that occurred on the WD-fed animals led to an increased abundance in bacteriophage belonging to the Siphoviridae family, and a decreased abundance in the Myoviridae family (**Figure 6**). Similar changes have been observed in studies characterizing changes in intestinal bacteriophage populations during inflammation (Norman et al., 2015; Gogokhia et al., 2019). Norman et al. reported increased abundance of Siphoviridae, and specific bacteria:bacteriophage correlations in a broad study of clinical samples from human inflammatory bowel diseases. For example, *Enterococcus bacteriophage phiFL1A*, a member of *phiFL-like viruses*, were positively correlated with Bacteroidaceae and Pasteurellaceae in Crohn’s disease while also being negatively correlated with Prevotellaceae (Norman et al., 2015). In our study, we observed a negative relationship between temperate *phiFL-like viruses* and members of Bacteroidaceae, Paseurellaceae and Prevotellaceae in line with the relative abundance changes of its host, Enterococcaceae, following WD-feeding. Members of Bacteroidaceae and Prevotellaceae are known to be reduced where Enterococcaceae and Pasturellaceae are known to be increased in abundance in the context of inflammatory bowel diseases (Gevers et al., 2014; Lewis et al., 2015; Alam et al., 2020; Horwat et al., 2020). The differences in our studies may reflect the relative degrees of inflammation in each system. Intestinal inflammation may drive disease-specific alterations in the intestinal microbiome while bacteriophage abundance under different diets may change more directly with the bacterial host’s response to the diet. Studies of longer term WD-induced obesity and the low grade inflammation associated with it would be likely to reveal results more similar to IBD.

Previous studies have also reported effects of diet on composition of the virome. Howe, *et al.* demonstrated distinct viral communities in individuals on a low fat vs. high-refined sugar, milkfat diet (Howe et al., 2016). One explanation for elevated bacteriophage abundance following diet change could be that viruses replicate at a much faster rate than bacteria. Bacteria typically produce one daughter cell per replication cycle where one bacteriophage can give rise to around 100 new virions within one host cell per replication cycle (Orlova, 2009). Additionally, bacteriophage require less resources for production of progeny than bacteria (Orlova, 2009). When the opportunity arises, such as a bloom in target bacteria, bacteriophage could benefit from the increase in viable host bacteria. Thus, a bloom of bacteria could give rise to a rapid bloom of bacteriophage that target this host. Bacterial abundance levels could appear reduced or stagnant as bacteriophage progeny infect new daughter bacterial cells (Manrique et al., 2016; Scanlan, 2017; Hsu et al., 2019). A plateau may also be reached such that bacteriophage co-exist with host as a result of co-evolution (Manrique et al., 2016; Scanlan, 2017; Hsu et al., 2019).

Many of the bacteriophage genera exhibited abundance patterns that correlated positively with their host, indicating that their relative abundance changed in parallel to their host (**Figure 7**). We further demonstrated several trends in bacteriophage abundance dynamics that did not follow that of their host, and this pattern was exacerbated in WD-fed mice. For example, members of the Streptococcaceae-targeting bacteriophage genera showed a wide range of responses to WD-feeding. *P335-like* and *936-like viruses* showed similar responses to diet change, directly corresponding to that of their host. While all members of *936-like viruses* are classified as virulent, members of *P335-like viruses* are composed of equal portions of virulent and temperate. Therefore, a bloom of *P335-like viruses* could arise as a result of a combination of interactions, such as prophage integration (temperate) in the host and kill-the-winner dynamics (virulent). In the “kill-the-winner” scenario, host bacteria increase in abundance, followed by a bloom in virulent bacteriophage who target that particular host (Maslov and Sneppen, 2017; Sutton and Hill, 2019). Consistent with this scenario we observed that *936-like viruses* increased following a bloom of their host Streptococcaceae (Mahony et al., 2006; Michelsen et al., 2007). As these studies have increased our understanding of phage-host dynamics, we must also acknowledge that such dynamics change very rapidly. Even though we sampled across numerous timepoints, these observations cannot fully account for changes that happened between timepoints. Future studies should therefore include more timepoints, and perhaps more targeted measurements, focusing only on specific phage and host bacteria.

The prevailing theory of bacteriophage ecology and abundance predicts that bacteriophage populations follow that of their putative host - either due to lysogeny or kill-the-winner dynamics - which much of our findings support (Knowles et al., 2016; Silveira and Rohwer, 2016; Maslov and Sneppen, 2017; Sutton and Hill, 2019). However, we also noted bacteriophage genera whose abundance inversely correlated with the abundance of their bacterial host. This was exemplified by *1706-like viruses*, which are virulent bacteriophage who target Streptococcaceae (Garneau et al., 2008), similar to *936-like* viruses. They exhibited a reduction in abundance as compared to its bacterial host in WD-fed animals, but not in Chow-fed animals. Potential explanations for this inverse correlation are: 1) it could be due to a decrease in as yet-unidentified bacterial host populations; 2) competition of different phages for the same host, perhaps some phages lose out (Trinh et al., 2017); 3) development of host resistance to the bacteriophage which would reportedly reduce the number of said bacteriophage in the environment (Hall et al., 2011; Sutton and Hill, 2019); or 4) as a recent publication reported, phage dynamics can change in response to non-host bacteria (Hsu et al., 2019). To our knowledge, *936-like* and *1706-like viruses* both target *Lactococcus* within Streptococcaceae. Bacteriophage persistence could also be based on sensitivities of these bacteriophage external triggers, such as metabolites or inflammatory products, within the microbiome following dietary exposure. Ongoing studies are aimed at more closely evaluating the specific bacteria:bacteriophage dynamics following dietary exposure.

Here we show that compositional shifts in the microbiota occur prior to the onset of pathophysiological metabolic parameters associated with obesity. Families within the class Bacilli, namely Enterococcaceae, Streptococcaceae, Bacillaceae and Staphylococcaceae, showed strong positive relationships with metabolic parameters elevated in WD-fed mice (**Figure 8**). Interestingly, these families have been previously implicated in the literature as increasing the energy-harvesting capacity of the microbiota, and thus contributing to the pathophysiology of obesity (Turnbaugh et al., 2006; Kobyliak et al., 2016). Therefore, the elevation in body weight, visceral fat, and metabolic inflexibility observed in the WD-fed mice could be directly related to the elevation in these bacterial components in the intestinal microbiota. The same study noted an inverse relationship between these obese-associated bacterial families and members of the bacterial families Clostridiaceae, Rumminococcaceae, Bacteroidaceae and Prevotellaceae (Turnbaugh et al., 2006). Similarly, we found that these families were depleted in WD-fed animals and had strong negative relationships with the metabolic parameters elevated in the WD-fed mice. Our findings support previous studies and implicate the above listed bacteria as those associated with obesity, and those representative of a leaner phenotype, respectively (Turnbaugh et al., 2006; Kobyliak et al., 2016).

With the surge of antibiotic resistance within recent years, there is renewed interest in using bacteriophage as therapy for bacterial infections (Ul Haq et al., 2012; Serwer et al., 2014; Danis-Wlodarczyk et al., 2016). One noted advantage of bacteriophage therapy to traditional antibiotics is the reduced impact on the organism’s commensal microbiome due to bacteriophage host specificity. In a similar fashion, bacteriophage could be utilized to deplete specific blooms of bacteria in the intestinal microbiome in order to induce a return to homeostasis which has been disrupted by colitis or obesity (Ul Haq et al., 2012; Serwer et al., 2014). The intestinal microbiota has been previously implicated in having a causal role in the development of obesity (Ridaura et al., 2013). Considering this study and other previous reports (Mai et al., 2015; Sybesma et al., 2016; Hsu et al., 2019), bacteriophage therapy could be used to deplete obese-associated bacteria. Thus, an important goal for the field will be further examination of bacteria-bacteriophage dynamics and the impacts of this on the surrounding microbiome community. By evaluating the dynamic relationship between bacteriophage and their bacterial hosts following WD-feeding, our work provides an overview of the timeline of disruption of both constituents and how this relates to the pathophysiology of obesity.

## Data Availability Statement

The datasets presented in this study can be found in online repositories. The names of the repository/repositories and accession number(s) can be found below: NCBI PRJNA730805.

## Ethics Statement

The animal study was reviewed and approved by Auburn University Institutional Animal Care and Use Committee.

## Author Contributions

KH carried out experiments and microbiome analysis and wrote manuscript. LW carried out experiments, analyzed metabolic data, and wrote some of the manuscript. HH assisted with experiments and microbiome analysis and figure generation. MG oversaw completion of metabolic experiments and wrote some of the manuscript. EH oversaw experiments and data analysis and edited manuscript. All authors contributed to the article and approved the submitted version.

## Funding

These studies were funded by a grant from the Alabama Experiment Station in the form of an Auburn University HATCH award (ALA021-1-14015).

## Conflict of Interest

The authors declare that the research was conducted in the absence of any commercial or financial relationships that could be construed as a potential conflict of interest.

## Publisher’s Note

All claims expressed in this article are solely those of the authors and do not necessarily represent those of their affiliated organizations, or those of the publisher, the editors and the reviewers. Any product that may be evaluated in this article, or claim that may be made by its manufacturer, is not guaranteed or endorsed by the publisher.

## Footnotes

1
https://www.ncbi.nlm.nih.gov/bioproject/PRJNA730805

2
https://www.mg-rast.org/mgmain.html?mgpage=project&project=mgp81921

3
https://talk.ictvonline.org/

4
https://www.mmpc.org/shared/regression.aspx
