# Supplementary material for: Integrative Longitudinal Analysis of Metabolic Phenotype and Microbiota Changes During the Development of Obesity
Source: Front Cell Infect Microbiol. 2021 Aug 3;11:671926. doi: 10.3389/fcimb.2021.671926 (PMC8370388; doi:10.3389/fcimb.2021.671926)
Supplement: Supplementary file 1 [file DataSheet_1.docx]

**Supplementary Figure Legends**

**Supplemental Figure 1. Activity at 2-, 4- and 12-weeks** A. Mean circadian analysis of activity at each hour in the 24-hour cycle in the Chow and WD groups after 2-weeks of dietary exposure. B. Mean circadian analysis of activity at each hour in the 24-hour cycle in the Chow and WD groups after 4-weeks of dietary exposure. C. Mean circadian analysis of activity at each hour in the 24-hour cycle in the Chow and WD groups after 12-weeks of dietary exposure. All data points are shown as group mean ± SE. (* p < 0.05, ** p < 0.01, *** p <0.001 compared to Chow)

**Supplemental Figure 2. Food and water uptake at 2-, 4- and 12-weeks** A. Circadian analysis of food uptake (*left panel*), water uptake (*middle panel*), and kcal consumed (*right panel*) for the day and night phases and total amount over a 24-hours in the Chow and WD groups after 2-weeks of dietary exposure. B. Circadian analysis of food uptake (*left panel*), water uptake (*middle panel*), and kcal consumed (*right panel*) for the day and night phases and total amount over a 24-hours in the Chow and WD groups after 4-weeks of dietary exposure. C. Circadian analysis of food uptake (*left panel*), water uptake (*middle panel*), and kcal consumed (*right panel*) for the day and night phases and total amount over a 24-hours in the Chow and WD groups after 12-weeks of dietary exposure. All data points are shown as group mean + SE. (* p < 0.05, ** p < 0.01, *** p <0.001 compared to Chow)

**Supplemental Figure 3. Alpha diversity rarefaction curves of microbiota over time** Rarefaction curves comparing the number of reads with the number of species found in the DNA from feces in mice in the Chow and WD groups over time. Total species count from all domains and total number of reads were used to plot alpha diversity of all samples at (A) time 0, (B) 2 days, (C) 2 weeks, (D) 8 weeks, and (E) 12 weeks of dietary exposure.

**Supplemental Figure 4.** **Diversity and evenness analysis of microbial assemblages over time** Shannon diversity was used to estimate changes in diversity for A) Bacterial OTUs and B) Bacteriophage OTUs (Viral OTUs within the order Caudovirales) and Pielou’s Evenness index was used to estimate changes in evenness for C) Bacterial OTUs and D) Bacteriophage OTUs within the fecal microbiota of mice in the Chow and WD groups at time 0, 2 days, 2, 8, and 12 weeks after diets began. *P < 0.05; **P < 0.01; ***P < 0.001. Statistical results outlined in Table S6. Pearson’s correlation plot of E) Diversity and Evenness with Metabolic parameters. Statistical results outlined in Table S10 and S11).

**Supplemental Figure 5.** **nMDS ordination of the microbiota samples separated by time** The taxonomic profiles of the samples were used to compute the sample dissimilarity matrix using Bray-Curtis dissimilarity index. The matrix was used to compute an ordination of the samples in two dimensions (MDS1 and MDS2). The stress associated with this ordination is 0.168. The shapes in the plot denote the diet (Chow or WD). Plots are separated by time point (0 days, 2 days and 2, 8, 12 weeks).

**Supplemental Figure 6.** **Timeline of the experimental procedures and sampling periods** Art sourced from SMART - Servier Medical ART.
