# Supplementary material for: Integrative Longitudinal Analysis of Metabolic Phenotype and Microbiota Changes During the Development of Obesity
Source: Front Cell Infect Microbiol. 2021 Aug 3;11:671926. doi: 10.3389/fcimb.2021.671926 (PMC8370388; doi:10.3389/fcimb.2021.671926)
Supplement: Supplementary file 2 [file Table_1.docx]

**Supplemental Table 1: *t-test* for Equality of Means of Bacterial Phylum**

Dependent Variable: Diet

|  | **Day 0** | | | | | | **2 Day PD** | | | | | | **2 Weeks PD** | | | | | |
| --- | --- | --- | --- | --- | --- | --- | --- | --- | --- | --- | --- | --- | --- | --- | --- | --- | --- | --- |
|  | **Chow** | | **WD** | | **F** | **P value** | **Chow** | | **WD** | | **F** | **P value** | **Chow** | | **WD** | | **F** | **P value** |
|  | **Mean** | **SD** | **Mean** | **SD** |  |  | **Mean** | **SD** | **Mean** | **SD** |  |  | **Mean** | **SD** | **Mean** | **SD** |  |  |
| **Acidobacteria** | 0.0169 | 0.0027 | 0.0212 | 0.0011 | -2.5619 | 0.0925 | 0.0164 | 0.0013 | 0.0199 | 0.0036 | -1.5923 | 0.2261 | 0.0185 | 0.0049 | 0.0222 | 0.0114 | -0.5194 | 0.6429 |
| **Actinobacteria** | 1.0115 | 0.2340 | 1.2461 | 0.1589 | -1.4363 | 0.2334 | 1.1595 | 0.1370 | 1.9674 | 1.1842 | -1.1738 | 0.3586 | 1.1076 | 0.0091 | 1.5182 | 0.3677 | -1.9335 | 0.1927 |
| **Aquificae** | 0.0158 | 0.0040 | 0.0182 | 0.0019 | -0.9166 | 0.4298 | 0.0155 | 0.0050 | 0.0149 | 0.0065 | 0.1086 | 0.9191 | 0.0258 | 0.0028 | 0.0107 | 0.0020 | 7.5807 | 0.0024 |
| **Bacteroidetes** | 33.4599 | 7.1430 | 25.1429 | 12.5966 | 0.9948 | 0.3897 | 19.0213 | 2.4043 | 24.3785 | 10.3077 | -0.8767 | 0.4652 | 29.7453 | 1.1519 | 18.6671 | 4.4650 | 4.1612 | 0.0427 |
| **Candidatus Poribacteria** | 0.0004 | 0.0002 | 0.0004 | 0.0003 | 0.0464 | 0.9655 | 0.0002 | 0.0004 | 0.0001 | 0.0001 | 0.7261 | 0.5358 | 0.0002 | 0.0001 | 0.0002 | 0.0002 | -0.2718 | 0.7997 |
| **Chlamydiae** | 0.0065 | 0.0026 | 0.0051 | 0.0016 | 0.8193 | 0.4676 | 0.0050 | 0.0005 | 0.0085 | 0.0031 | -1.9016 | 0.1916 | 0.0043 | 0.0026 | 0.0107 | 0.0095 | -1.1064 | 0.3708 |
| **Chlorobi** | 0.0929 | 0.0216 | 0.0645 | 0.0240 | 1.5242 | 0.2029 | 0.0588 | 0.0047 | 0.0619 | 0.0181 | -0.2846 | 0.7999 | 0.0914 | 0.0188 | 0.0591 | 0.0161 | 2.2574 | 0.0885 |
| **Chloroflexi** | 0.1576 | 0.0380 | 0.2045 | 0.0230 | -1.8268 | 0.1569 | 0.1828 | 0.0575 | 0.1192 | 0.0194 | 1.8155 | 0.1873 | 0.2289 | 0.1055 | 0.0949 | 0.0185 | 2.1663 | 0.1552 |
| **Chrysiogenetes** | 0.0029 | 0.0017 | 0.0033 | 0.0014 | -0.3529 | 0.7423 | 0.0019 | 0.0001 | 0.0025 | 0.0007 | -1.4973 | 0.2659 | 0.0047 | 0.0012 | 0.0024 | 0.0014 | 2.1958 | 0.0947 |
| **Cyanobacteria** | 0.0786 | 0.0079 | 0.0829 | 0.0029 | -0.8739 | 0.4568 | 0.0666 | 0.0066 | 0.0516 | 0.0009 | 3.9136 | 0.0564 | 0.0796 | 0.0027 | 0.0597 | 0.0029 | 8.7899 | 0.0009 |
| **Deferribacteres** | 0.0137 | 0.0012 | 0.0085 | 0.0019 | 3.9640 | 0.0221 | 0.0106 | 0.0028 | 0.0075 | 0.0014 | 1.6825 | 0.1926 | 0.0137 | 0.0051 | 0.0058 | 0.0003 | 2.6773 | 0.1147 |
| **Deinococcus Thermus** | 0.0219 | 0.0080 | 0.0288 | 0.0064 | -1.1554 | 0.3151 | 0.0199 | 0.0029 | 0.0174 | 0.0028 | 1.0693 | 0.3452 | 0.0233 | 0.0032 | 0.0179 | 0.0063 | 1.3272 | 0.2772 |
| **Dictyoglomi** | 0.0136 | 0.0046 | 0.0093 | 0.0029 | 1.3844 | 0.2505 | 0.0100 | 0.0028 | 0.0058 | 0.0027 | 1.8800 | 0.1334 | 0.0097 | 0.0030 | 0.0041 | 0.0006 | 3.1220 | 0.0798 |
| **Elusimicrobia** | 0.0187 | 0.0059 | 0.0206 | 0.0046 | -0.4256 | 0.6935 | 0.0161 | 0.0031 | 0.0070 | 0.0017 | 4.4525 | 0.0196 | 0.0260 | 0.0083 | 0.0052 | 0.0026 | 4.1360 | 0.0393 |
| **Fibrobacteres** | 0.1480 | 0.0285 | 0.1458 | 0.0034 | 0.1318 | 0.9069 | 0.1342 | 0.0008 | 0.1023 | 0.0188 | 2.9370 | 0.0986 | 0.1565 | 0.0216 | 0.0929 | 0.0254 | 3.3010 | 0.0311 |
| **Firmicutes** | 60.6344 | 8.5264 | 69.0064 | 14.6555 | -0.8552 | 0.4514 | 76.2758 | 2.4178 | 62.0968 | 5.0612 | 4.3784 | 0.0242 | 65.3037 | 1.0628 | 59.8249 | 12.9156 | 0.7323 | 0.5393 |
| **Fusobacteria** | 0.2480 | 0.0201 | 0.2431 | 0.0300 | 0.2380 | 0.8252 | 0.2670 | 0.0260 | 0.2111 | 0.0170 | 3.1163 | 0.0435 | 0.2711 | 0.0370 | 0.1650 | 0.0522 | 2.8690 | 0.0515 |
| **Gemmatimonadetes** | 0.0000 | 0.0000 | 0.0001 | 0.0001 | -1.0000 | 0.4226 | 0.0000 | 0.0000 | 0.0000 | 0.0000 | NA | NA | 0.0000 | 0.0000 | 0.0000 | 0.0000 | NA | NA |
| **Lentisphaerae** | 0.0245 | 0.0068 | 0.0251 | 0.0063 | -0.1012 | 0.9243 | 0.0230 | 0.0064 | 0.0231 | 0.0057 | -0.0184 | 0.9862 | 0.0365 | 0.0017 | 0.0227 | 0.0044 | 5.0927 | 0.0205 |
| **Nitrospirae** | 0.0069 | 0.0030 | 0.0037 | 0.0013 | 1.6438 | 0.2086 | 0.0083 | 0.0027 | 0.0027 | 0.0006 | 3.5159 | 0.0641 | 0.0080 | 0.0038 | 0.0075 | 0.0068 | 0.1219 | 0.9104 |
| **Planctomycetes** | 0.0116 | 0.0058 | 0.0153 | 0.0027 | -0.9908 | 0.3991 | 0.0096 | 0.0023 | 0.0100 | 0.0032 | -0.1650 | 0.8777 | 0.0161 | 0.0066 | 0.0071 | 0.0045 | 1.9605 | 0.1310 |
| **Proteobacteria** | 1.6753 | 0.2227 | 1.5759 | 0.1263 | 0.6723 | 0.5472 | 1.6449 | 0.1060 | 1.2158 | 0.1593 | 3.8826 | 0.0231 | 1.9402 | 0.0781 | 1.0776 | 0.1043 | 11.4665 | 0.0005 |
| **Spirochaetes** | 0.2081 | 0.0467 | 0.2147 | 0.0396 | -0.1843 | 0.8629 | 0.2195 | 0.0192 | 0.1788 | 0.0146 | 2.9230 | 0.0469 | 0.2266 | 0.0197 | 0.1461 | 0.0487 | 2.6550 | 0.0880 |
| **Synergistetes** | 0.0700 | 0.0143 | 0.0977 | 0.0260 | -1.6187 | 0.2007 | 0.0907 | 0.0199 | 0.0631 | 0.0121 | 2.0488 | 0.1246 | 0.0987 | 0.0182 | 0.0500 | 0.0191 | 3.1951 | 0.0332 |
| **Tenericutes** | 0.0416 | 0.0008 | 0.0600 | 0.0175 | -1.8130 | 0.2110 | 0.0502 | 0.0210 | 0.0286 | 0.0104 | 1.5965 | 0.2111 | 0.0515 | 0.0071 | 0.0187 | 0.0044 | 6.8145 | 0.0044 |
| **Thermotogae** | 0.0362 | 0.0050 | 0.0357 | 0.0045 | 0.1138 | 0.9150 | 0.0368 | 0.0008 | 0.0247 | 0.0012 | 14.7262 | 0.0003 | 0.0433 | 0.0062 | 0.0199 | 0.0032 | 5.7821 | 0.0104 |
| **Verrucomicrobia** | 1.7157 | 1.8932 | 1.4609 | 2.3527 | 0.1461 | 0.8912 | 0.3970 | 0.5314 | 9.1822 | 4.5616 | -3.3133 | 0.0774 | 0.1221 | 0.0216 | 17.8570 | 17.1073 | -1.7956 | 0.2144 |
| **Unclassified** | 0.2685 | 0.0783 | 0.2594 | 0.0166 | 0.1973 | 0.8605 | 0.2585 | 0.0097 | 0.1985 | 0.0772 | 1.3353 | 0.3101 | 0.3467 | 0.0229 | 0.2322 | 0.0894 | 2.1484 | 0.1499 |
|  | **8 Weeks PD** | | | | | | **12 Weeks PD** | | | | | |  |  |  |  |  |  |
|  | **Chow** | | **WD** | | **F** | **P value** | **Chow** | | **WD** | | **F** | **P value** |  |  |  |  |  |  |
|  | **Mean** | **SD** | **Mean** | **SD** |  |  | **Mean** | **SD** | **Mean** | **SD** |  |  |  |  |  |  |  |  |
| **Acidobacteria** | 0.0266 | 0.0025 | 0.0289 | 0.0094 | -0.4194 | 0.7111 | 0.0192 | 0.0012 | 0.0121 | 0.0028 | 4.1126 | 0.0317 |  |  |  |  |  |  |
| **Actinobacteria** | 2.2840 | 0.7685 | 1.9732 | 0.5100 | 0.5838 | 0.5951 | 1.0792 | 0.0578 | 0.8049 | 0.6903 | 0.6858 | 0.5628 |  |  |  |  |  |  |
| **Aquificae** | 0.0170 | 0.0010 | 0.0184 | 0.0029 | -0.7708 | 0.5084 | 0.0199 | 0.0069 | 0.0092 | 0.0010 | 2.6647 | 0.1122 |  |  |  |  |  |  |
| **Bacteroidetes** | 27.3240 | 4.0921 | 33.7088 | 11.2064 | -0.9270 | 0.4338 | 20.9146 | 1.3653 | 9.3150 | 1.0016 | 11.8652 | 0.0005 |  |  |  |  |  |  |
| **Candidatus Poribacteria** | 0.0003 | 0.0003 | 0.0001 | 0.0002 | 1.0273 | 0.3717 | 0.0002 | 0.0002 | 0.0001 | 0.0001 | 0.5826 | 0.6071 |  |  |  |  |  |  |
| **Chlamydiae** | 0.0099 | 0.0018 | 0.0128 | 0.0105 | -0.4821 | 0.6750 | 0.0063 | 0.0025 | 0.0045 | 0.0030 | 0.8008 | 0.4697 |  |  |  |  |  |  |
| **Chlorobi** | 0.0808 | 0.0081 | 0.0894 | 0.0096 | -1.1882 | 0.3022 | 0.0700 | 0.0066 | 0.0324 | 0.0048 | 7.9632 | 0.0019 |  |  |  |  |  |  |
| **Chloroflexi** | 0.1798 | 0.0527 | 0.1286 | 0.0138 | 1.6280 | 0.2300 | 0.1579 | 0.0076 | 0.0469 | 0.0138 | 12.2052 | 0.0010 |  |  |  |  |  |  |
| **Chrysiogenetes** | 0.0049 | 0.0028 | 0.0038 | 0.0025 | 0.5423 | 0.6169 | 0.0041 | 0.0012 | 0.0014 | 0.0005 | 3.6275 | 0.0398 |  |  |  |  |  |  |
| **Cyanobacteria** | 0.0788 | 0.0030 | 0.0718 | 0.0029 | 2.9210 | 0.0432 | 0.0639 | 0.0205 | 0.0289 | 0.0025 | 2.9416 | 0.0954 |  |  |  |  |  |  |
| **Deferribacteres** | 0.0100 | 0.0011 | 0.0083 | 0.0026 | 0.9954 | 0.3990 | 0.0095 | 0.0027 | 0.0056 | 0.0016 | 2.1513 | 0.1120 |  |  |  |  |  |  |
| **Deinococcus Thermus** | 0.0243 | 0.0040 | 0.0201 | 0.0006 | 1.8064 | 0.2074 | 0.0216 | 0.0015 | 0.0117 | 0.0017 | 7.7508 | 0.0016 |  |  |  |  |  |  |
| **Dictyoglomi** | 0.0086 | 0.0007 | 0.0057 | 0.0032 | 1.5548 | 0.2491 | 0.0076 | 0.0011 | 0.0029 | 0.0002 | 7.2448 | 0.0151 |  |  |  |  |  |  |
| **Elusimicrobia** | 0.0148 | 0.0014 | 0.0039 | 0.0019 | 8.0589 | 0.0017 | 0.0096 | 0.0018 | 0.0026 | 0.0003 | 6.7384 | 0.0177 |  |  |  |  |  |  |
| **Fibrobacteres** | 0.1209 | 0.0103 | 0.1097 | 0.0410 | 0.4587 | 0.6870 | 0.1070 | 0.0347 | 0.0426 | 0.0089 | 3.1104 | 0.0768 |  |  |  |  |  |  |
| **Firmicutes** | 63.0616 | 11.5859 | 50.1269 | 10.3486 | 1.4422 | 0.2236 | 72.0128 | 4.0232 | 83.8270 | 5.4487 | -3.0212 | 0.0436 |  |  |  |  |  |  |
| **Fusobacteria** | 0.2279 | 0.0356 | 0.2037 | 0.0511 | 0.6724 | 0.5424 | 0.2284 | 0.0685 | 0.0979 | 0.0128 | 3.2440 | 0.0761 |  |  |  |  |  |  |
| **Gemmatimonadetes** | 0.0000 | 0.0000 | 0.0000 | 0.0000 | NA | NA | 0.0000 | 0.0001 | 0.0000 | 0.0000 | 1.0000 | 0.4226 |  |  |  |  |  |  |
| **Lentisphaerae** | 0.0315 | 0.0056 | 0.0388 | 0.0036 | -1.9000 | 0.1428 | 0.0209 | 0.0055 | 0.0123 | 0.0034 | 2.3150 | 0.0945 |  |  |  |  |  |  |
| **Nitrospirae** | 0.0070 | 0.0007 | 0.0033 | 0.0018 | 3.2807 | 0.0550 | 0.0059 | 0.0040 | 0.0029 | 0.0018 | 1.1742 | 0.3321 |  |  |  |  |  |  |
| **Planctomycetes** | 0.0100 | 0.0022 | 0.0104 | 0.0027 | -0.1691 | 0.8742 | 0.0115 | 0.0020 | 0.0070 | 0.0004 | 3.8256 | 0.0538 |  |  |  |  |  |  |
| **Proteobacteria** | 1.2083 | 0.0807 | 1.1996 | 0.1550 | 0.0861 | 0.9368 | 1.1432 | 0.0532 | 0.7664 | 0.0233 | 11.2376 | 0.0023 |  |  |  |  |  |  |
| **Spirochaetes** | 0.1817 | 0.0195 | 0.1387 | 0.0279 | 2.1834 | 0.1024 | 0.1943 | 0.0610 | 0.0663 | 0.0103 | 3.5820 | 0.0644 |  |  |  |  |  |  |
| **Synergistetes** | 0.0733 | 0.0139 | 0.0582 | 0.0154 | 1.2650 | 0.2752 | 0.0607 | 0.0220 | 0.0316 | 0.0021 | 2.2811 | 0.1478 |  |  |  |  |  |  |
| **Tenericutes** | 0.0897 | 0.0435 | 0.0281 | 0.0112 | 2.3762 | 0.1259 | 0.0573 | 0.0456 | 0.0121 | 0.0046 | 1.7101 | 0.2268 |  |  |  |  |  |  |
| **Thermotogae** | 0.0326 | 0.0009 | 0.0255 | 0.0073 | 1.6724 | 0.2328 | 0.0331 | 0.0046 | 0.0148 | 0.0012 | 6.7247 | 0.0154 |  |  |  |  |  |  |
| **Verrucomicrobia** | 4.5844 | 7.7586 | 11.6976 | 19.8855 | -0.5772 | 0.6100 | 3.5131 | 4.8459 | 4.7196 | 4.9197 | -0.3026 | 0.7773 |  |  |  |  |  |  |
| **Unclassified** | 0.3073 | 0.0263 | 0.2856 | 0.0694 | 0.5065 | 0.6528 | 0.2281 | 0.0600 | 0.1212 | 0.0260 | 2.8320 | 0.0738 |  |  |  |  |  |  |
