# Supplementary material for: Integrative Longitudinal Analysis of Metabolic Phenotype and Microbiota Changes During the Development of Obesity
Source: Front Cell Infect Microbiol. 2021 Aug 3;11:671926. doi: 10.3389/fcimb.2021.671926 (PMC8370388; doi:10.3389/fcimb.2021.671926)
Supplement: Supplementary file 3 [file Table_2.docx]

**Supplemental Table 2: *t-test* for Equality of Means of Bacterial Class**

Dependent Variable: Diet

|  | **Day 0** | | | | | | **2 Day PD** | | | | | | **2 Weeks PD** | | | | | |
| --- | --- | --- | --- | --- | --- | --- | --- | --- | --- | --- | --- | --- | --- | --- | --- | --- | --- | --- |
|  | **Chow** | | **WD** | | **F** | **P value** | **Chow** | | **WD** | | **F** | **P value** | **Chow** | | **WD** | | **F** | **P value** |
|  | **Mean** | **SD** | **Mean** | **SD** |  |  | **Mean** | **SD** | **Mean** | **SD** |  |  | **Mean** | **SD** | **Mean** | **SD** |  |  |
| **Acidobacteria** | 0.0046 | 0.0004 | 0.0048 | 0.0018 | -0.2056 | 0.8545 | 0.0064 | 0.0026 | 0.0043 | 0.0013 | 1.2663 | 0.2987 | 0.0060 | 0.0019 | 0.0069 | 0.0018 | -0.5618 | 0.6043 |
| **Actinobacteria** | 1.0115 | 0.2340 | 1.2461 | 0.1589 | -1.4363 | 0.2334 | 1.1595 | 0.1370 | 1.9674 | 1.1842 | -1.1738 | 0.3586 | 1.1076 | 0.0091 | 1.5182 | 0.3677 | -1.9335 | 0.1927 |
| **Alphaproteobacteria** | 0.1916 | 0.0140 | 0.1904 | 0.0213 | 0.0812 | 0.9398 | 0.1916 | 0.0274 | 0.1564 | 0.0085 | 2.1271 | 0.1463 | 0.2003 | 0.0108 | 0.1392 | 0.0162 | 5.4280 | 0.0083 |
| **Aquificae** | 0.0158 | 0.0040 | 0.0182 | 0.0019 | -0.9166 | 0.4298 | 0.0155 | 0.0050 | 0.0149 | 0.0065 | 0.1086 | 0.9191 | 0.0258 | 0.0028 | 0.0107 | 0.0020 | 7.5807 | 0.0024 |
| **Bacilli** | 4.3385 | 1.0310 | 5.3006 | 1.4395 | -0.9412 | 0.4050 | 6.7097 | 0.4031 | 18.7973 | 10.9472 | -1.9112 | 0.1958 | 4.5825 | 0.3947 | 20.8941 | 0.8892 | -29.0423 | 0.0002 |
| **Bacteroidia** | 32.2789 | 7.0163 | 24.2589 | 12.1273 | 0.9915 | 0.3903 | 18.3455 | 2.3031 | 23.5626 | 10.1106 | -0.8714 | 0.4679 | 28.5402 | 1.1458 | 17.9140 | 4.3112 | 4.1259 | 0.0430 |
| **Betaproteobacteria** | 0.2160 | 0.0144 | 0.2027 | 0.0442 | 0.4953 | 0.6618 | 0.1826 | 0.0077 | 0.1756 | 0.0213 | 0.5406 | 0.6329 | 0.2348 | 0.0061 | 0.1422 | 0.0141 | 10.4265 | 0.0029 |
| **Chlamydiae** | 0.0065 | 0.0026 | 0.0051 | 0.0016 | 0.8193 | 0.4676 | 0.0050 | 0.0005 | 0.0085 | 0.0031 | -1.9016 | 0.1916 | 0.0043 | 0.0026 | 0.0107 | 0.0095 | -1.1064 | 0.3708 |
| **Chlorobia** | 0.0929 | 0.0216 | 0.0645 | 0.0240 | 1.5242 | 0.2029 | 0.0588 | 0.0047 | 0.0619 | 0.0181 | -0.2846 | 0.7999 | 0.0914 | 0.0188 | 0.0591 | 0.0161 | 2.2574 | 0.0885 |
| **Chloroflexi** | 0.0402 | 0.0059 | 0.0392 | 0.0107 | 0.1357 | 0.9003 | 0.0332 | 0.0058 | 0.0295 | 0.0055 | 0.8043 | 0.4664 | 0.0407 | 0.0015 | 0.0271 | 0.0101 | 2.3149 | 0.1410 |
| **Chrysiogenetes** | 0.0029 | 0.0017 | 0.0033 | 0.0014 | -0.3529 | 0.7423 | 0.0019 | 0.0001 | 0.0025 | 0.0007 | -1.4973 | 0.2659 | 0.0047 | 0.0012 | 0.0024 | 0.0014 | 2.1958 | 0.0947 |
| **Clostridia** | 53.8404 | 7.2323 | 60.8438 | 12.9274 | -0.8189 | 0.4704 | 66.5744 | 2.1208 | 40.2170 | 6.9430 | 6.2885 | 0.0158 | 57.8370 | 1.3140 | 36.4697 | 12.0200 | 3.0607 | 0.0896 |
| **Cytophagia** | 0.2265 | 0.0354 | 0.1649 | 0.0849 | 1.1600 | 0.3390 | 0.1173 | 0.0145 | 0.1464 | 0.0415 | -1.1494 | 0.3491 | 0.2131 | 0.0248 | 0.1254 | 0.0368 | 3.4220 | 0.0328 |
| **Deferribacteres** | 0.0137 | 0.0012 | 0.0085 | 0.0019 | 3.9640 | 0.0221 | 0.0106 | 0.0028 | 0.0075 | 0.0014 | 1.6825 | 0.1926 | 0.0137 | 0.0051 | 0.0058 | 0.0003 | 2.6773 | 0.1147 |
| **Dehalococcoidetes** | 0.1096 | 0.0310 | 0.1554 | 0.0221 | -2.0840 | 0.1129 | 0.1412 | 0.0543 | 0.0844 | 0.0131 | 1.7637 | 0.2067 | 0.1788 | 0.1074 | 0.0653 | 0.0106 | 1.8212 | 0.2078 |
| **Deinococci** | 0.0219 | 0.0080 | 0.0288 | 0.0064 | -1.1554 | 0.3151 | 0.0199 | 0.0029 | 0.0174 | 0.0028 | 1.0693 | 0.3452 | 0.0233 | 0.0032 | 0.0179 | 0.0063 | 1.3272 | 0.2772 |
| **Deltaproteobacteria** | 0.2280 | 0.0165 | 0.2327 | 0.0071 | -0.4522 | 0.6847 | 0.2153 | 0.0261 | 0.1897 | 0.0274 | 1.1721 | 0.3064 | 0.2378 | 0.0116 | 0.1734 | 0.0080 | 7.9099 | 0.0023 |
| **Dictyoglomia** | 0.0136 | 0.0046 | 0.0093 | 0.0029 | 1.3844 | 0.2505 | 0.0100 | 0.0028 | 0.0058 | 0.0027 | 1.8800 | 0.1334 | 0.0097 | 0.0030 | 0.0041 | 0.0006 | 3.1220 | 0.0798 |
| **Elusimicrobia** | 0.0187 | 0.0059 | 0.0206 | 0.0046 | -0.4256 | 0.6935 | 0.0161 | 0.0031 | 0.0070 | 0.0017 | 4.4525 | 0.0196 | 0.0260 | 0.0083 | 0.0052 | 0.0026 | 4.1360 | 0.0393 |
| **Epsilonproteobacteria** | 0.0843 | 0.0053 | 0.1006 | 0.0141 | -1.8673 | 0.1744 | 0.0942 | 0.0153 | 0.0701 | 0.0059 | 2.5451 | 0.0980 | 0.1089 | 0.0140 | 0.0524 | 0.0075 | 6.1776 | 0.0081 |
| **Erysipelotrichi** | 1.9170 | 0.3814 | 2.2195 | 0.2105 | -1.2030 | 0.3124 | 2.3229 | 0.2195 | 2.6881 | 1.1166 | -0.5559 | 0.6306 | 2.2906 | 0.1197 | 2.1707 | 0.7594 | 0.2701 | 0.8113 |
| **Fibrobacteres** | 0.1480 | 0.0285 | 0.1458 | 0.0034 | 0.1318 | 0.9069 | 0.1342 | 0.0008 | 0.1023 | 0.0188 | 2.9370 | 0.0986 | 0.1565 | 0.0216 | 0.0929 | 0.0254 | 3.3010 | 0.0311 |
| **Flavobacteria** | 0.5078 | 0.0695 | 0.3594 | 0.1964 | 1.2338 | 0.3209 | 0.2871 | 0.0327 | 0.3441 | 0.0928 | -1.0036 | 0.4030 | 0.5240 | 0.0230 | 0.3428 | 0.0794 | 3.7990 | 0.0491 |
| **Fusobacteria** | 0.2480 | 0.0201 | 0.2431 | 0.0300 | 0.2380 | 0.8252 | 0.2670 | 0.0260 | 0.2111 | 0.0170 | 3.1163 | 0.0435 | 0.2711 | 0.0370 | 0.1650 | 0.0522 | 2.8690 | 0.0515 |
| **Gammaproteobacteria** | 0.9489 | 0.1931 | 0.8416 | 0.1195 | 0.8185 | 0.4675 | 0.9558 | 0.0969 | 0.6190 | 0.0974 | 4.2446 | 0.0132 | 1.1509 | 0.0813 | 0.5652 | 0.0783 | 8.9873 | 0.0009 |
| **Gemmatimonadetes** | 0.0000 | 0.0000 | 0.0001 | 0.0001 | -1.0000 | 0.4226 | 0.0000 | 0.0000 | 0.0000 | 0.0000 | NA | NA | 0.0000 | 0.0000 | 0.0000 | 0.0000 | NA | NA |
| **Ktedonobacteria** | 0.0026 | 0.0012 | 0.0041 | 0.0026 | -0.8675 | 0.4526 | 0.0030 | 0.0010 | 0.0014 | 0.0003 | 2.5927 | 0.1031 | 0.0028 | 0.0012 | 0.0008 | 0.0006 | 2.5467 | 0.0865 |
| **Mollicutes** | 0.0416 | 0.0008 | 0.0600 | 0.0175 | -1.8130 | 0.2110 | 0.0502 | 0.0210 | 0.0286 | 0.0104 | 1.5965 | 0.2111 | 0.0515 | 0.0071 | 0.0187 | 0.0044 | 6.8145 | 0.0044 |
| **Negativicutes** | 0.5385 | 0.0825 | 0.6425 | 0.1160 | -1.2642 | 0.2816 | 0.6689 | 0.0442 | 0.3944 | 0.0850 | 4.9606 | 0.0156 | 0.5936 | 0.0712 | 0.2905 | 0.0748 | 5.0853 | 0.0071 |
| **Nitrospira** | 0.0068 | 0.0030 | 0.0037 | 0.0013 | 1.6560 | 0.2048 | 0.0083 | 0.0027 | 0.0026 | 0.0007 | 3.5291 | 0.0584 | 0.0080 | 0.0039 | 0.0075 | 0.0068 | 0.1053 | 0.9225 |
| **Opitutae** | 0.0140 | 0.0040 | 0.0206 | 0.0106 | -1.0149 | 0.3966 | 0.0124 | 0.0026 | 0.0210 | 0.0020 | -4.5492 | 0.0122 | 0.0161 | 0.0033 | 0.0246 | 0.0124 | -1.1454 | 0.3579 |
| **Planctomycetacia** | 0.0116 | 0.0058 | 0.0153 | 0.0027 | -0.9908 | 0.3991 | 0.0096 | 0.0023 | 0.0100 | 0.0032 | -0.1650 | 0.8777 | 0.0161 | 0.0066 | 0.0071 | 0.0045 | 1.9605 | 0.1310 |
| **Solibacteres** | 0.0037 | 0.0010 | 0.0095 | 0.0028 | -3.3834 | 0.0558 | 0.0053 | 0.0008 | 0.0071 | 0.0028 | -1.0620 | 0.3860 | 0.0065 | 0.0008 | 0.0077 | 0.0070 | -0.2936 | 0.7960 |
| **Spartobacteria** | 0.0038 | 0.0039 | 0.0038 | 0.0037 | 0.0173 | 0.9870 | 0.0016 | 0.0008 | 0.0151 | 0.0086 | -2.7000 | 0.1120 | 0.0011 | 0.0005 | 0.0309 | 0.0284 | -1.8183 | 0.2106 |
| **Sphingobacteria** | 0.2253 | 0.0498 | 0.1524 | 0.0841 | 1.2925 | 0.2804 | 0.1217 | 0.0180 | 0.1555 | 0.0624 | -0.9021 | 0.4504 | 0.2074 | 0.0107 | 0.1194 | 0.0238 | 5.8497 | 0.0123 |
| **Spirochaetes** | 0.2081 | 0.0467 | 0.2147 | 0.0396 | -0.1843 | 0.8629 | 0.2195 | 0.0192 | 0.1788 | 0.0146 | 2.9230 | 0.0469 | 0.2266 | 0.0197 | 0.1461 | 0.0487 | 2.6550 | 0.0880 |
| **Synergistia** | 0.0700 | 0.0143 | 0.0977 | 0.0260 | -1.6187 | 0.2007 | 0.0907 | 0.0199 | 0.0631 | 0.0121 | 2.0488 | 0.1246 | 0.0987 | 0.0182 | 0.0500 | 0.0191 | 3.1951 | 0.0332 |
| **Thermomicrobia** | 0.0052 | 0.0023 | 0.0058 | 0.0025 | -0.2899 | 0.7864 | 0.0052 | 0.0005 | 0.0038 | 0.0013 | 1.7782 | 0.1896 | 0.0064 | 0.0015 | 0.0018 | 0.0004 | 4.9651 | 0.0286 |
| **Thermotogae** | 0.0362 | 0.0050 | 0.0357 | 0.0045 | 0.1138 | 0.9150 | 0.0368 | 0.0008 | 0.0247 | 0.0012 | 14.7262 | 0.0003 | 0.0433 | 0.0062 | 0.0199 | 0.0032 | 5.7821 | 0.0104 |
| **Verrucomicrobiae** | 1.6937 | 1.8840 | 1.4355 | 2.3379 | 0.1489 | 0.8891 | 0.3817 | 0.5293 | 9.1423 | 4.5505 | -3.3122 | 0.0775 | 0.1038 | 0.0184 | 17.7995 | 17.0677 | -1.7958 | 0.2144 |
| **Zetaproteobacteria** | 0.0017 | 0.0010 | 0.0009 | 0.0001 | 1.3066 | 0.3168 | 0.0014 | 0.0009 | 0.0007 | 0.0003 | 1.2084 | 0.3269 | 0.0026 | 0.0027 | 0.0010 | 0.0004 | 1.0838 | 0.3868 |

**Supplemental Table 2: *t-test* for Equality of Means of Bacterial Class Continued**

Dependent Variable: Diet

|  | **8 Weeks PD** | | | | | | | | | **12 Weeks PD** | | | | | | | | | |  |
| --- | --- | --- | --- | --- | --- | --- | --- | --- | --- | --- | --- | --- | --- | --- | --- | --- | --- | --- | --- | --- |
|  | **Chow** | | | **WD** | | | **F** | | **P value** | | **Chow** | | | **WD** | | | **F** | | **P value** | |
|  | **Mean** | **SD** | **Mean** | | **SD** |  | |  | | **Mean** | | **SD** | **Mean** | | **SD** |  | |  | |  |
| Acidobacteria | 0.0087 | 0.0027 | 0.0112 | | 0.0071 | -0.5544 | | 0.6239 | | 0.0073 | | 0.0021 | 0.0039 | | 0.0001 | 2.7458 | | 0.1102 | |  |
| Actinobacteria | 2.2840 | 0.7685 | 1.9732 | | 0.5100 | 0.5838 | | 0.5951 | | 1.0792 | | 0.0578 | 0.8049 | | 0.6903 | 0.6858 | | 0.5628 | |  |
| Alphaproteobacteria | 0.1764 | 0.0189 | 0.1966 | | 0.0284 | -1.0270 | | 0.3705 | | 0.1774 | | 0.0239 | 0.0816 | | 0.0079 | 6.6032 | | 0.0132 | |  |
| Aquificae | 0.0170 | 0.0010 | 0.0184 | | 0.0029 | -0.7708 | | 0.5084 | | 0.0199 | | 0.0069 | 0.0092 | | 0.0010 | 2.6647 | | 0.1122 | |  |
| Bacilli | 8.2869 | 2.7337 | 12.1133 | | 3.6861 | -1.4442 | | 0.2279 | | 19.3473 | | 18.1593 | 63.8379 | | 5.8326 | -4.0403 | | 0.0408 | |  |
| Bacteroidia | 26.2543 | 3.9684 | 32.2797 | | 10.7306 | -0.9122 | | 0.4399 | | 20.0728 | | 1.4229 | 8.9048 | | 0.9720 | 11.2252 | | 0.0007 | |  |
| Betaproteobacteria | 0.1832 | 0.0276 | 0.2334 | | 0.0652 | -1.2266 | | 0.3163 | | 0.1554 | | 0.0083 | 0.0793 | | 0.0057 | 13.0686 | | 0.0004 | |  |
| Chlamydiae | 0.0099 | 0.0018 | 0.0128 | | 0.0105 | -0.4821 | | 0.6750 | | 0.0063 | | 0.0025 | 0.0045 | | 0.0030 | 0.8008 | | 0.4697 | |  |
| Chlorobia | 0.0808 | 0.0081 | 0.0894 | | 0.0096 | -1.1882 | | 0.3022 | | 0.0700 | | 0.0066 | 0.0324 | | 0.0048 | 7.9632 | | 0.0019 | |  |
| Chloroflexi | 0.0331 | 0.0071 | 0.0202 | | 0.0036 | 2.8206 | | 0.0675 | | 0.0286 | | 0.0045 | 0.0140 | | 0.0044 | 4.0007 | | 0.0161 | |  |
| Chrysiogenetes | 0.0049 | 0.0028 | 0.0038 | | 0.0025 | 0.5423 | | 0.6169 | | 0.0041 | | 0.0012 | 0.0014 | | 0.0005 | 3.6275 | | 0.0398 | |  |
| Clostridia | 51.7009 | 8.4848 | 35.6281 | | 8.3864 | 2.3335 | | 0.0800 | | 50.2054 | | 21.2627 | 18.9502 | | 2.0179 | 2.5346 | | 0.1245 | |  |
| Cytophagia | 0.1911 | 0.0275 | 0.2218 | | 0.0653 | -0.7521 | | 0.5124 | | 0.1547 | | 0.0052 | 0.0720 | | 0.0068 | 16.6650 | | 0.0001 | |  |
| Deferribacteres | 0.0100 | 0.0011 | 0.0083 | | 0.0026 | 0.9954 | | 0.3990 | | 0.0095 | | 0.0027 | 0.0056 | | 0.0016 | 2.1513 | | 0.1120 | |  |
| Dehalococcoidetes | 0.1389 | 0.0469 | 0.1040 | | 0.0109 | 1.2574 | | 0.3248 | | 0.1217 | | 0.0088 | 0.0292 | | 0.0103 | 11.8525 | | 0.0003 | |  |
| Deinococci | 0.0243 | 0.0040 | 0.0201 | | 0.0006 | 1.8064 | | 0.2074 | | 0.0216 | | 0.0015 | 0.0117 | | 0.0017 | 7.7508 | | 0.0016 | |  |
| Deltaproteobacteria | 0.2116 | 0.0075 | 0.1976 | | 0.0161 | 1.3590 | | 0.2723 | | 0.2108 | | 0.0328 | 0.0968 | | 0.0095 | 5.7884 | | 0.0198 | |  |
| Dictyoglomia | 0.0086 | 0.0007 | 0.0057 | | 0.0032 | 1.5548 | | 0.2491 | | 0.0076 | | 0.0011 | 0.0029 | | 0.0002 | 7.2448 | | 0.0151 | |  |
| Elusimicrobia | 0.0148 | 0.0014 | 0.0039 | | 0.0019 | 8.0589 | | 0.0017 | | 0.0096 | | 0.0018 | 0.0026 | | 0.0003 | 6.7384 | | 0.0177 | |  |
| Epsilonproteobacteria | 0.0797 | 0.0062 | 0.0601 | | 0.0099 | 2.9044 | | 0.0545 | | 0.0842 | | 0.0161 | 0.0465 | | 0.0109 | 3.3659 | | 0.0343 | |  |
| Erysipelotrichi | 2.6134 | 0.3463 | 2.0048 | | 0.4775 | 1.7873 | | 0.1553 | | 1.9712 | | 0.5337 | 0.8522 | | 0.2085 | 3.3828 | | 0.0533 | |  |
| Fibrobacteres | 0.1209 | 0.0103 | 0.1097 | | 0.0410 | 0.4587 | | 0.6870 | | 0.1070 | | 0.0347 | 0.0426 | | 0.0089 | 3.1104 | | 0.0768 | |  |
| Flavobacteria | 0.4448 | 0.0807 | 0.6375 | | 0.2355 | -1.3409 | | 0.2902 | | 0.3616 | | 0.0376 | 0.1826 | | 0.0212 | 7.1823 | | 0.0047 | |  |
| Fusobacteria | 0.2279 | 0.0356 | 0.2037 | | 0.0511 | 0.6724 | | 0.5424 | | 0.2284 | | 0.0685 | 0.0979 | | 0.0128 | 3.2440 | | 0.0761 | |  |
| Gammaproteobacteria | 0.5526 | 0.1011 | 0.5073 | | 0.0934 | 0.5702 | | 0.5992 | | 0.5085 | | 0.0397 | 0.4598 | | 0.0205 | 1.8879 | | 0.1556 | |  |
| Gemmatimonadetes | 0.0000 | 0.0000 | 0.0000 | | 0.0000 | NA | | NA | | 0.0000 | | 0.0001 | 0.0000 | | 0.0000 | 1.0000 | | 0.4226 | |  |
| Ktedonobacteria | 0.0019 | 0.0006 | 0.0008 | | 0.0006 | 2.3441 | | 0.0791 | | 0.0021 | | 0.0005 | 0.0008 | | 0.0000 | 4.9209 | | 0.0376 | |  |
| Mollicutes | 0.0897 | 0.0435 | 0.0281 | | 0.0112 | 2.3762 | | 0.1259 | | 0.0573 | | 0.0456 | 0.0121 | | 0.0046 | 1.7101 | | 0.2268 | |  |
| Negativicutes | 0.4604 | 0.0373 | 0.3806 | | 0.1370 | 0.9732 | | 0.4216 | | 0.4889 | | 0.1581 | 0.1867 | | 0.0096 | 3.3052 | | 0.0798 | |  |
| Nitrospira | 0.0070 | 0.0007 | 0.0033 | | 0.0018 | 3.2807 | | 0.0550 | | 0.0059 | | 0.0040 | 0.0027 | | 0.0017 | 1.2674 | | 0.3025 | |  |
| Opitutae | 0.0169 | 0.0098 | 0.0252 | | 0.0114 | -0.9587 | | 0.3931 | | 0.0180 | | 0.0058 | 0.0090 | | 0.0036 | 2.2981 | | 0.0962 | |  |
| Planctomycetacia | 0.0100 | 0.0022 | 0.0104 | | 0.0027 | -0.1691 | | 0.8742 | | 0.0115 | | 0.0020 | 0.0070 | | 0.0004 | 3.8256 | | 0.0538 | |  |
| Solibacteres | 0.0094 | 0.0016 | 0.0087 | | 0.0023 | 0.4206 | | 0.6981 | | 0.0056 | | 0.0004 | 0.0050 | | 0.0007 | 1.2937 | | 0.2809 | |  |
| Spartobacteria | 0.0077 | 0.0115 | 0.0162 | | 0.0267 | -0.5046 | | 0.6520 | | 0.0053 | | 0.0048 | 0.0060 | | 0.0059 | -0.1555 | | 0.8843 | |  |
| Sphingobacteria | 0.1980 | 0.0129 | 0.2243 | | 0.0690 | -0.6485 | | 0.5793 | | 0.1682 | | 0.0166 | 0.0679 | | 0.0037 | 10.1970 | | 0.0068 | |  |
| Spirochaetes | 0.1817 | 0.0195 | 0.1387 | | 0.0279 | 2.1834 | | 0.1024 | | 0.1943 | | 0.0610 | 0.0663 | | 0.0103 | 3.5820 | | 0.0644 | |  |
| Synergistia | 0.0733 | 0.0139 | 0.0582 | | 0.0154 | 1.2650 | | 0.2752 | | 0.0607 | | 0.0220 | 0.0316 | | 0.0021 | 2.2811 | | 0.1478 | |  |
| Thermomicrobia | 0.0059 | 0.0021 | 0.0036 | | 0.0012 | 1.6065 | | 0.2011 | | 0.0054 | | 0.0014 | 0.0029 | | 0.0011 | 2.4503 | | 0.0728 | |  |
| Thermotogae | 0.0326 | 0.0009 | 0.0255 | | 0.0073 | 1.6724 | | 0.2328 | | 0.0331 | | 0.0046 | 0.0148 | | 0.0012 | 6.7247 | | 0.0154 | |  |
| Verrucomicrobiae | 4.5543 | 7.7315 | 11.6543 | | 19.8470 | -0.5774 | | 0.6100 | | 3.4844 | | 4.8305 | 4.7022 | | 4.9096 | -0.3062 | | 0.7747 | |  |
| Zetaproteobacteria | 0.0012 | 0.0006 | 0.0006 | | 0.0004 | 1.4228 | | 0.2308 | | 0.0018 | | 0.0009 | 0.0003 | | 0.0001 | 2.8045 | | 0.1019 | |  |
