# Supplementary material for: Integrative Longitudinal Analysis of Metabolic Phenotype and Microbiota Changes During the Development of Obesity
Source: Front Cell Infect Microbiol. 2021 Aug 3;11:671926. doi: 10.3389/fcimb.2021.671926 (PMC8370388; doi:10.3389/fcimb.2021.671926)
Supplement: Supplementary file 4 [file Table_3.docx]

**Supplemental Table 3: *t-test* for Equality of Means of Bacterial Family**

Dependent Variable: Diet

|  | **Day 0** | | | | | | **2 Days PD** | | | | | | **2 Weeks PD** | | | | | |
| --- | --- | --- | --- | --- | --- | --- | --- | --- | --- | --- | --- | --- | --- | --- | --- | --- | --- | --- |
|  | **Chow** | | **WD** | | **F** | **P value** | **Chow** | | **WD** | | **F** | **P value** | **Chow** | | **WD** | | **F** | **P value** |
|  | **Mean** | **SD** | **Mean** | **SD** |  |  | **Mean** | **SD** | **Mean** | **SD** |  |  | **Mean** | **SD** | **Mean** | **SD** |  |  |
| **Acetobacteraceae** | 0.0079 | 0.0013 | 0.0105 | 0.0028 | -1.4529 | 0.2494 | 0.0117 | 0.0035 | 0.0071 | 0.0019 | 1.9888 | 0.1395 | 0.0106 | 0.0022 | 0.0060 | 0.0028 | 2.2031 | 0.0957 |
| **Acholeplasmataceae** | 0.0217 | 0.0013 | 0.0326 | 0.0163 | -1.1498 | 0.3678 | 0.0291 | 0.0161 | 0.0110 | 0.0046 | 1.8817 | 0.1824 | 0.0260 | 0.0035 | 0.0064 | 0.0031 | 7.3557 | 0.0019 |
| **Acidaminococcaceae** | 0.2349 | 0.0341 | 0.2810 | 0.0424 | -1.4676 | 0.2192 | 0.2992 | 0.0309 | 0.1731 | 0.0545 | 3.4864 | 0.0367 | 0.2545 | 0.0275 | 0.1176 | 0.0343 | 5.3917 | 0.0065 |
| **Acidimicrobiaceae** | 0.0004 | 0.0002 | 0.0005 | 0.0001 | -0.4345 | 0.6940 | 0.0011 | 0.0015 | 0.0005 | 0.0003 | 0.6694 | 0.5674 | 0.0006 | 0.0001 | 0.0005 | 0.0003 | 0.0347 | 0.9752 |
| **Acidithiobacillaceae** | 0.0011 | 0.0003 | 0.0071 | 0.0045 | -2.2823 | 0.1490 | 0.0013 | 0.0011 | 0.0006 | 0.0004 | 1.0747 | 0.3786 | 0.0025 | 0.0012 | 0.0026 | 0.0014 | -0.0592 | 0.9557 |
| **Acidobacteriaceae** | 0.0046 | 0.0004 | 0.0048 | 0.0018 | -0.2056 | 0.8545 | 0.0064 | 0.0026 | 0.0043 | 0.0013 | 1.2663 | 0.2987 | 0.0060 | 0.0019 | 0.0069 | 0.0018 | -0.5618 | 0.6043 |
| **Acidothermaceae** | 0.0010 | 0.0001 | 0.0027 | 0.0014 | -2.1077 | 0.1673 | 0.0031 | 0.0019 | 0.0015 | 0.0006 | 1.4159 | 0.2752 | 0.0009 | 0.0004 | 0.0012 | 0.0004 | -0.8573 | 0.4396 |
| **Actinomycetaceae** | 0.0569 | 0.0087 | 0.0711 | 0.0156 | -1.3776 | 0.2584 | 0.0676 | 0.0062 | 0.0454 | 0.0086 | 3.6195 | 0.0263 | 0.0746 | 0.0053 | 0.0374 | 0.0054 | 8.5099 | 0.0010 |
| **Actinosynnemataceae** | 0.0015 | 0.0007 | 0.0012 | 0.0005 | 0.6136 | 0.5751 | 0.0022 | 0.0009 | 0.0015 | 0.0011 | 0.7817 | 0.4792 | 0.0036 | 0.0021 | 0.0006 | 0.0006 | 2.4608 | 0.1158 |
| **Aerococcaceae** | 0.2825 | 0.0557 | 0.3571 | 0.1007 | -1.1222 | 0.3407 | 0.4013 | 0.0299 | 0.2156 | 0.0449 | 5.9650 | 0.0061 | 0.3106 | 0.0423 | 0.1617 | 0.0633 | 3.3861 | 0.0341 |
| **Aeromonadaceae** | 0.0152 | 0.0010 | 0.0184 | 0.0082 | -0.6687 | 0.5708 | 0.0128 | 0.0025 | 0.0098 | 0.0015 | 1.7418 | 0.1728 | 0.0214 | 0.0035 | 0.0064 | 0.0009 | 7.1496 | 0.0139 |
| **Alcaligenaceae** | 0.0183 | 0.0067 | 0.0171 | 0.0034 | 0.2725 | 0.8031 | 0.0160 | 0.0038 | 0.0119 | 0.0034 | 1.3919 | 0.2372 | 0.0233 | 0.0008 | 0.0162 | 0.0062 | 1.9551 | 0.1854 |
| **Alcanivoracaceae** | 0.0016 | 0.0004 | 0.0021 | 0.0009 | -0.8929 | 0.4405 | 0.0013 | 0.0012 | 0.0017 | 0.0011 | -0.4461 | 0.6787 | 0.0015 | 0.0008 | 0.0027 | 0.0016 | -1.1593 | 0.3314 |
| **Alicyclobacillaceae** | 0.0284 | 0.0072 | 0.0313 | 0.0096 | -0.4175 | 0.6993 | 0.0285 | 0.0043 | 0.0202 | 0.0009 | 3.3214 | 0.0712 | 0.0300 | 0.0095 | 0.0137 | 0.0036 | 2.7904 | 0.0824 |
| **Alteromonadaceae** | 0.0162 | 0.0107 | 0.0116 | 0.0003 | 0.7409 | 0.5358 | 0.0098 | 0.0052 | 0.0126 | 0.0056 | -0.6472 | 0.5529 | 0.0162 | 0.0011 | 0.0133 | 0.0045 | 1.0925 | 0.3777 |
| **Anaeroplasmataceae** | 0.0008 | 0.0004 | 0.0026 | 0.0012 | -2.5289 | 0.1055 | 0.0018 | 0.0013 | 0.0000 | 0.0000 | 2.3838 | 0.1400 | 0.0023 | 0.0011 | 0.0000 | 0.0000 | 3.4190 | 0.0759 |
| **Anaplasmataceae** | 0.0042 | 0.0024 | 0.0054 | 0.0012 | -0.7955 | 0.4860 | 0.0062 | 0.0008 | 0.0027 | 0.0006 | 5.8590 | 0.0051 | 0.0067 | 0.0020 | 0.0037 | 0.0038 | 1.2200 | 0.3087 |
| **Aquificaceae** | 0.0074 | 0.0040 | 0.0090 | 0.0014 | -0.6840 | 0.5525 | 0.0089 | 0.0042 | 0.0074 | 0.0028 | 0.5104 | 0.6405 | 0.0096 | 0.0028 | 0.0045 | 0.0004 | 3.1700 | 0.0814 |
| **Aurantimonadaceae** | 0.0038 | 0.0035 | 0.0020 | 0.0015 | 0.8555 | 0.4612 | 0.0030 | 0.0011 | 0.0022 | 0.0010 | 0.9596 | 0.3919 | 0.0015 | 0.0004 | 0.0005 | 0.0002 | 3.4974 | 0.0357 |
| **Bacillaceae** | 0.4745 | 0.0539 | 0.4722 | 0.0407 | 0.0586 | 0.9563 | 0.5166 | 0.0279 | 0.3619 | 0.0085 | 9.1685 | 0.0067 | 0.5744 | 0.0204 | 0.3376 | 0.0664 | 5.8992 | 0.0181 |
| **Bacteriovoracaceae** | 0.0000 | 0.0001 | 0.0002 | 0.0003 | -0.7298 | 0.5341 | 0.0000 | 0.0000 | 0.0000 | 0.0000 | NA | NA | 0.0006 | 0.0001 | 0.0000 | 0.0000 | 10.8407 | 0.0084 |
| **Bacteroidaceae** | 16.9668 | 2.8893 | 14.6955 | 3.8636 | 0.8154 | 0.4640 | 12.8647 | 1.7347 | 13.0650 | 2.9102 | -0.1024 | 0.9244 | 19.2531 | 0.5765 | 11.4497 | 2.7660 | 4.7836 | 0.0348 |
| **Bartonellaceae** | 0.0009 | 0.0007 | 0.0016 | 0.0005 | -1.4353 | 0.2322 | 0.0010 | 0.0004 | 0.0010 | 0.0006 | -0.0424 | 0.9685 | 0.0010 | 0.0003 | 0.0007 | 0.0004 | 1.3624 | 0.2497 |
| **Bdellovibrionaceae** | 0.0000 | 0.0000 | 0.0000 | 0.0000 | NA | NA | 0.0000 | 0.0000 | 0.0001 | 0.0001 | -1.0000 | 0.4226 | 0.0000 | 0.0000 | 0.0000 | 0.0000 | NA | NA |
| **Beijerinckiaceae** | 0.0025 | 0.0012 | 0.0038 | 0.0004 | -1.8946 | 0.1773 | 0.0030 | 0.0007 | 0.0025 | 0.0012 | 0.5969 | 0.5904 | 0.0028 | 0.0006 | 0.0010 | 0.0005 | 3.9627 | 0.0178 |
| **Beutenbergiaceae** | 0.0039 | 0.0011 | 0.0015 | 0.0006 | 3.2932 | 0.0428 | 0.0019 | 0.0021 | 0.0029 | 0.0023 | -0.6180 | 0.5703 | 0.0030 | 0.0028 | 0.0007 | 0.0003 | 1.4277 | 0.2877 |
| **Bifidobacteriaceae** | 0.2036 | 0.0229 | 0.3002 | 0.0900 | -1.8018 | 0.1988 | 0.2204 | 0.0314 | 1.1213 | 0.9059 | -1.7215 | 0.2270 | 0.2255 | 0.0426 | 0.8041 | 0.5844 | -1.7103 | 0.2280 |
| **Blattabacteriaceae** | 0.0023 | 0.0014 | 0.0022 | 0.0010 | 0.0694 | 0.9484 | 0.0032 | 0.0021 | 0.0015 | 0.0010 | 1.2522 | 0.3010 | 0.0041 | 0.0022 | 0.0010 | 0.0004 | 2.4309 | 0.1278 |
| **Brachyspiraceae** | 0.0870 | 0.0073 | 0.0812 | 0.0135 | 0.6541 | 0.5587 | 0.0950 | 0.0032 | 0.0679 | 0.0028 | 11.0879 | 0.0004 | 0.0859 | 0.0049 | 0.0685 | 0.0284 | 1.0423 | 0.4015 |
| **Bradyrhizobiaceae** | 0.0211 | 0.0021 | 0.0225 | 0.0029 | -0.6610 | 0.5476 | 0.0187 | 0.0031 | 0.0163 | 0.0002 | 1.2894 | 0.3252 | 0.0229 | 0.0065 | 0.0184 | 0.0043 | 0.9872 | 0.3873 |
| **Brevibacteriaceae** | 0.0004 | 0.0003 | 0.0005 | 0.0001 | -0.2120 | 0.8486 | 0.0006 | 0.0009 | 0.0007 | 0.0009 | -0.1065 | 0.9203 | 0.0007 | 0.0002 | 0.0003 | 0.0001 | 3.0974 | 0.0475 |
| **Brucellaceae** | 0.0181 | 0.0062 | 0.0224 | 0.0139 | -0.4835 | 0.6644 | 0.0182 | 0.0051 | 0.0231 | 0.0047 | -1.2367 | 0.2843 | 0.0281 | 0.0047 | 0.0215 | 0.0039 | 1.8540 | 0.1398 |
| **Burkholderiaceae** | 0.0393 | 0.0039 | 0.0375 | 0.0083 | 0.3277 | 0.7659 | 0.0315 | 0.0043 | 0.0251 | 0.0037 | 1.9838 | 0.1201 | 0.0341 | 0.0024 | 0.0191 | 0.0021 | 8.1328 | 0.0014 |
| **Campylobacteraceae** | 0.0359 | 0.0043 | 0.0414 | 0.0099 | -0.8784 | 0.4502 | 0.0373 | 0.0050 | 0.0320 | 0.0034 | 1.4902 | 0.2199 | 0.0497 | 0.0065 | 0.0314 | 0.0029 | 4.4524 | 0.0245 |
| **Cardiobacteriaceae** | 0.0014 | 0.0001 | 0.0021 | 0.0007 | -1.7965 | 0.2092 | 0.0029 | 0.0026 | 0.0011 | 0.0001 | 1.2358 | 0.3419 | 0.0026 | 0.0007 | 0.0014 | 0.0006 | 2.1373 | 0.1007 |
| **Carnobacteriaceae** | 0.0190 | 0.0037 | 0.0227 | 0.0038 | -1.2290 | 0.2864 | 0.0237 | 0.0056 | 0.0137 | 0.0014 | 2.9584 | 0.0855 | 0.0262 | 0.0006 | 0.0139 | 0.0052 | 4.0258 | 0.0543 |
| **Catenulisporaceae** | 0.0045 | 0.0064 | 0.0023 | 0.0005 | 0.5777 | 0.6211 | 0.0011 | 0.0005 | 0.0017 | 0.0011 | -0.8548 | 0.4594 | 0.0028 | 0.0023 | 0.0008 | 0.0004 | 1.4513 | 0.2762 |
| **Caulobacteraceae** | 0.0144 | 0.0043 | 0.0111 | 0.0005 | 1.3327 | 0.3110 | 0.0116 | 0.0037 | 0.0088 | 0.0033 | 0.9483 | 0.3974 | 0.0136 | 0.0007 | 0.0065 | 0.0030 | 3.9793 | 0.0497 |
| **Cellulomonadaceae** | 0.0042 | 0.0019 | 0.0020 | 0.0010 | 1.8098 | 0.1675 | 0.0028 | 0.0011 | 0.0017 | 0.0005 | 1.5488 | 0.2204 | 0.0042 | 0.0028 | 0.0024 | 0.0010 | 1.0831 | 0.3733 |
| **Chlamydiaceae** | 0.0023 | 0.0013 | 0.0020 | 0.0008 | 0.2798 | 0.7958 | 0.0019 | 0.0005 | 0.0058 | 0.0025 | -2.6271 | 0.1098 | 0.0014 | 0.0006 | 0.0092 | 0.0102 | -1.3049 | 0.3211 |
| **Chlorobiaceae** | 0.0929 | 0.0216 | 0.0645 | 0.0240 | 1.5242 | 0.2029 | 0.0588 | 0.0047 | 0.0619 | 0.0181 | -0.2846 | 0.7999 | 0.0914 | 0.0188 | 0.0591 | 0.0161 | 2.2574 | 0.0885 |
| **Chloroflexaceae** | 0.0296 | 0.0025 | 0.0327 | 0.0072 | -0.7018 | 0.5429 | 0.0252 | 0.0012 | 0.0243 | 0.0054 | 0.2784 | 0.8048 | 0.0253 | 0.0012 | 0.0211 | 0.0068 | 1.0450 | 0.4004 |
| **Chromatiaceae** | 0.0116 | 0.0014 | 0.0124 | 0.0004 | -0.8626 | 0.4693 | 0.0115 | 0.0021 | 0.0117 | 0.0042 | -0.0695 | 0.9491 | 0.0126 | 0.0034 | 0.0053 | 0.0021 | 3.1534 | 0.0444 |
| **Chrysiogenaceae** | 0.0029 | 0.0017 | 0.0033 | 0.0014 | -0.3529 | 0.7423 | 0.0019 | 0.0001 | 0.0025 | 0.0007 | -1.4973 | 0.2659 | 0.0047 | 0.0012 | 0.0024 | 0.0014 | 2.1958 | 0.0947 |
| **Clostridiaceae** | 19.2809 | 2.6412 | 21.9955 | 4.8145 | -0.8562 | 0.4529 | 23.4983 | 0.9972 | 14.4178 | 2.1545 | 6.6248 | 0.0085 | 20.7901 | 0.7362 | 12.6154 | 3.9390 | 3.5334 | 0.0649 |
| **Clostridiales.Family.XI..Incertae.Sedis** | 0.1059 | 0.0147 | 0.1316 | 0.0080 | -2.6600 | 0.0742 | 0.1396 | 0.0050 | 0.0938 | 0.0043 | 12.0103 | 0.0003 | 0.1436 | 0.0085 | 0.0747 | 0.0141 | 7.2598 | 0.0039 |
| **Clostridiales.Family.XIV..Incertae.Sedis** | 0.0000 | 0.0000 | 0.0000 | 0.0000 | NA | NA | 0.0000 | 0.0000 | 0.0000 | 0.0000 | NA | NA | 0.0001 | 0.0002 | 0.0000 | 0.0000 | 1.0000 | 0.4226 |
| **Clostridiales.Family.XVII..Incertae.Sedis** | 0.0071 | 0.0013 | 0.0100 | 0.0052 | -0.9512 | 0.4319 | 0.0071 | 0.0043 | 0.0056 | 0.0007 | 0.6105 | 0.6010 | 0.0079 | 0.0023 | 0.0030 | 0.0007 | 3.4614 | 0.0567 |
| **Colwelliaceae** | 0.0027 | 0.0030 | 0.0051 | 0.0037 | -0.8483 | 0.4460 | 0.0030 | 0.0013 | 0.0037 | 0.0012 | -0.6808 | 0.5337 | 0.0042 | 0.0045 | 0.0030 | 0.0006 | 0.4658 | 0.6857 |
| **Comamonadaceae** | 0.0231 | 0.0050 | 0.0206 | 0.0020 | 0.8135 | 0.4828 | 0.0269 | 0.0044 | 0.0210 | 0.0012 | 2.2239 | 0.1406 | 0.0268 | 0.0068 | 0.0158 | 0.0030 | 2.5520 | 0.0921 |
| **Conexibacteraceae** | 0.0018 | 0.0007 | 0.0023 | 0.0007 | -0.7497 | 0.4951 | 0.0030 | 0.0014 | 0.0018 | 0.0005 | 1.3315 | 0.2922 | 0.0020 | 0.0014 | 0.0012 | 0.0005 | 0.9292 | 0.4328 |
| **Coriobacteriaceae** | 0.5912 | 0.1677 | 0.7269 | 0.2274 | -0.8318 | 0.4561 | 0.6872 | 0.0827 | 0.6757 | 0.3157 | 0.0611 | 0.9562 | 0.6200 | 0.0431 | 0.5744 | 0.1995 | 0.3871 | 0.7332 |
| **Corynebacteriaceae** | 0.0109 | 0.0017 | 0.0128 | 0.0039 | -0.7823 | 0.4965 | 0.0106 | 0.0017 | 0.0066 | 0.0016 | 2.9287 | 0.0434 | 0.0148 | 0.0043 | 0.0069 | 0.0037 | 2.4035 | 0.0754 |
| **Coxiellaceae** | 0.0018 | 0.0005 | 0.0025 | 0.0006 | -1.3457 | 0.2562 | 0.0019 | 0.0015 | 0.0014 | 0.0005 | 0.5668 | 0.6196 | 0.0035 | 0.0008 | 0.0008 | 0.0007 | 4.2134 | 0.0140 |
| **Cyclobacteriaceae** | 0.0099 | 0.0017 | 0.0095 | 0.0067 | 0.1122 | 0.9199 | 0.0103 | 0.0046 | 0.0054 | 0.0023 | 1.6331 | 0.2017 | 0.0163 | 0.0115 | 0.0083 | 0.0031 | 1.1645 | 0.3511 |
| **Cystobacteraceae** | 0.0007 | 0.0001 | 0.0029 | 0.0007 | -5.4613 | 0.0291 | 0.0013 | 0.0008 | 0.0013 | 0.0001 | 0.0022 | 0.9984 | 0.0022 | 0.0012 | 0.0014 | 0.0015 | 0.7633 | 0.4896 |
| **Cytophagaceae** | 0.1902 | 0.0310 | 0.1319 | 0.0722 | 1.2848 | 0.2977 | 0.0899 | 0.0216 | 0.1179 | 0.0402 | -1.0615 | 0.3648 | 0.1721 | 0.0143 | 0.0986 | 0.0321 | 3.6306 | 0.0412 |
| **Deferribacteraceae** | 0.0137 | 0.0012 | 0.0085 | 0.0019 | 3.9640 | 0.0221 | 0.0106 | 0.0028 | 0.0075 | 0.0014 | 1.6825 | 0.1926 | 0.0137 | 0.0051 | 0.0058 | 0.0003 | 2.6773 | 0.1147 |
| **Deinococcaceae** | 0.0101 | 0.0047 | 0.0139 | 0.0035 | -1.0962 | 0.3394 | 0.0082 | 0.0012 | 0.0088 | 0.0018 | -0.4397 | 0.6858 | 0.0092 | 0.0013 | 0.0121 | 0.0055 | -0.8797 | 0.4637 |
| **Dermabacteraceae** | 0.0022 | 0.0006 | 0.0013 | 0.0005 | 1.8468 | 0.1400 | 0.0015 | 0.0007 | 0.0011 | 0.0005 | 0.8314 | 0.4557 | 0.0038 | 0.0015 | 0.0021 | 0.0005 | 1.7558 | 0.2002 |
| **Dermacoccaceae** | 0.0026 | 0.0022 | 0.0015 | 0.0004 | 0.8621 | 0.4755 | 0.0016 | 0.0006 | 0.0042 | 0.0015 | -2.8155 | 0.0805 | 0.0024 | 0.0017 | 0.0013 | 0.0012 | 0.8815 | 0.4324 |
| **Desulfarculaceae** | 0.0030 | 0.0028 | 0.0018 | 0.0006 | 0.7478 | 0.5261 | 0.0029 | 0.0010 | 0.0028 | 0.0013 | 0.0814 | 0.9392 | 0.0031 | 0.0015 | 0.0013 | 0.0009 | 1.7698 | 0.1664 |
| **Desulfobacteraceae** | 0.0153 | 0.0025 | 0.0136 | 0.0009 | 1.1387 | 0.3528 | 0.0136 | 0.0024 | 0.0093 | 0.0008 | 2.9074 | 0.0800 | 0.0188 | 0.0022 | 0.0083 | 0.0022 | 5.9072 | 0.0041 |
| **Desulfobulbaceae** | 0.0122 | 0.0053 | 0.0105 | 0.0039 | 0.4548 | 0.6749 | 0.0110 | 0.0017 | 0.0129 | 0.0032 | -0.8886 | 0.4383 | 0.0172 | 0.0055 | 0.0087 | 0.0076 | 1.5818 | 0.1953 |
| **Desulfohalobiaceae** | 0.0026 | 0.0010 | 0.0049 | 0.0005 | -3.6894 | 0.0349 | 0.0030 | 0.0005 | 0.0027 | 0.0012 | 0.4413 | 0.6925 | 0.0041 | 0.0016 | 0.0015 | 0.0005 | 2.6753 | 0.0984 |
| **Desulfomicrobiaceae** | 0.0102 | 0.0010 | 0.0154 | 0.0044 | -1.9987 | 0.1721 | 0.0097 | 0.0051 | 0.0120 | 0.0024 | -0.7216 | 0.5251 | 0.0083 | 0.0045 | 0.0057 | 0.0035 | 0.7909 | 0.4755 |
| **Desulfovibrionaceae** | 0.0575 | 0.0074 | 0.0623 | 0.0095 | -0.6893 | 0.5306 | 0.0564 | 0.0084 | 0.0463 | 0.0075 | 1.5592 | 0.1948 | 0.0547 | 0.0065 | 0.0378 | 0.0031 | 4.0434 | 0.0294 |
| **Desulfurobacteriaceae** | 0.0026 | 0.0007 | 0.0035 | 0.0017 | -0.8400 | 0.4699 | 0.0016 | 0.0009 | 0.0021 | 0.0008 | -0.7088 | 0.5189 | 0.0071 | 0.0012 | 0.0018 | 0.0013 | 5.0607 | 0.0072 |
| **Desulfuromonadaceae** | 0.0083 | 0.0007 | 0.0157 | 0.0037 | -3.3842 | 0.0696 | 0.0141 | 0.0032 | 0.0100 | 0.0062 | 1.0302 | 0.3792 | 0.0098 | 0.0039 | 0.0076 | 0.0030 | 0.7671 | 0.4882 |
| **Dictyoglomaceae** | 0.0136 | 0.0046 | 0.0093 | 0.0029 | 1.3844 | 0.2505 | 0.0100 | 0.0028 | 0.0058 | 0.0027 | 1.8800 | 0.1334 | 0.0097 | 0.0030 | 0.0041 | 0.0006 | 3.1220 | 0.0798 |
| **Dietziaceae** | 0.0002 | 0.0001 | 0.0005 | 0.0004 | -1.2815 | 0.3208 | 0.0003 | 0.0004 | 0.0001 | 0.0001 | 0.8161 | 0.4922 | 0.0007 | 0.0008 | 0.0001 | 0.0001 | 1.3738 | 0.3005 |

**Supplemental Table 3: *t-test* for Equality of Means of Bacterial Family Continued**

Dependent Variable: Diet

|  | **Day 0** | | | | | | **2 Days PD** | | | | | | **2 Weeks PD** | | | | | |
| --- | --- | --- | --- | --- | --- | --- | --- | --- | --- | --- | --- | --- | --- | --- | --- | --- | --- | --- |
|  | **Chow** | | **WD** | | **F** | **P value** | **Chow** | | **WD** | | **F** | **P value** | **Chow** | | **WD** | | **F** | **P value** |
|  | **Mean** | **SD** | **Mean** | **SD** |  |  | **Mean** | **SD** | **Mean** | **SD** |  |  | **Mean** | **SD** | **Mean** | **SD** |  |  |
| **Ectothiorhodospiraceae** | 0.0163 | 0.0025 | 0.0206 | 0.0038 | -1.6255 | 0.1901 | 0.0158 | 0.0072 | 0.0158 | 0.0037 | 0.0155 | 0.9886 | 0.0163 | 0.0006 | 0.0145 | 0.0029 | 1.0559 | 0.3940 |
| **Elusimicrobiaceae** | 0.0187 | 0.0059 | 0.0206 | 0.0046 | -0.4256 | 0.6935 | 0.0161 | 0.0031 | 0.0070 | 0.0017 | 4.4525 | 0.0196 | 0.0260 | 0.0083 | 0.0052 | 0.0026 | 4.1360 | 0.0393 |
| **Enterobacteriaceae** | 0.1886 | 0.0277 | 0.1666 | 0.0232 | 1.0557 | 0.3524 | 0.1692 | 0.0167 | 0.1293 | 0.0153 | 3.0457 | 0.0386 | 0.2336 | 0.0255 | 0.1198 | 0.0160 | 6.5585 | 0.0050 |
| **Enterococcaceae** | 0.8228 | 0.2616 | 1.2704 | 0.5463 | -1.2800 | 0.2942 | 1.2970 | 0.1122 | 0.7489 | 0.1640 | 4.7788 | 0.0119 | 0.9451 | 0.3640 | 0.7345 | 0.1147 | 0.9554 | 0.4256 |
| **Entomoplasmataceae** | 0.0029 | 0.0010 | 0.0054 | 0.0028 | -1.4085 | 0.2723 | 0.0046 | 0.0011 | 0.0048 | 0.0021 | -0.1020 | 0.9253 | 0.0038 | 0.0014 | 0.0036 | 0.0031 | 0.1193 | 0.9130 |
| **Erysipelotrichaceae** | 1.9170 | 0.3814 | 2.2195 | 0.2105 | -1.2030 | 0.3124 | 2.3229 | 0.2195 | 2.6881 | 1.1166 | -0.5559 | 0.6306 | 2.2906 | 0.1197 | 2.1707 | 0.7594 | 0.2701 | 0.8113 |
| **Erythrobacteraceae** | 0.0038 | 0.0009 | 0.0016 | 0.0006 | 3.7091 | 0.0272 | 0.0032 | 0.0013 | 0.0027 | 0.0010 | 0.5075 | 0.6404 | 0.0022 | 0.0007 | 0.0015 | 0.0004 | 1.4875 | 0.2271 |
| **Eubacteriaceae** | 8.3302 | 1.0495 | 9.1017 | 2.0583 | -0.5784 | 0.6039 | 10.2006 | 0.5624 | 5.9014 | 1.2561 | 5.4107 | 0.0152 | 8.5027 | 0.1872 | 5.4188 | 2.0783 | 2.5598 | 0.1227 |
| **Ferrimonadaceae** | 0.0053 | 0.0010 | 0.0033 | 0.0008 | 2.7836 | 0.0535 | 0.0039 | 0.0018 | 0.0038 | 0.0007 | 0.0889 | 0.9354 | 0.0042 | 0.0011 | 0.0031 | 0.0009 | 1.2723 | 0.2755 |
| **Fibrobacteraceae** | 0.1480 | 0.0285 | 0.1458 | 0.0034 | 0.1318 | 0.9069 | 0.1342 | 0.0008 | 0.1023 | 0.0188 | 2.9370 | 0.0986 | 0.1565 | 0.0216 | 0.0929 | 0.0254 | 3.3010 | 0.0311 |
| **Flammeovirgaceae** | 0.0264 | 0.0031 | 0.0235 | 0.0147 | 0.3278 | 0.7719 | 0.0171 | 0.0036 | 0.0231 | 0.0056 | -1.5578 | 0.2068 | 0.0246 | 0.0109 | 0.0186 | 0.0035 | 0.9186 | 0.4408 |
| **Flavobacteriaceae** | 0.4405 | 0.0565 | 0.3013 | 0.1617 | 1.4074 | 0.2715 | 0.2379 | 0.0231 | 0.2855 | 0.0874 | -0.9125 | 0.4475 | 0.4396 | 0.0212 | 0.2899 | 0.0680 | 3.6365 | 0.0519 |
| **Francisellaceae** | 0.0139 | 0.0017 | 0.0165 | 0.0011 | -2.2473 | 0.0963 | 0.0159 | 0.0082 | 0.0045 | 0.0009 | 2.4071 | 0.1351 | 0.0181 | 0.0139 | 0.0162 | 0.0168 | 0.1529 | 0.8861 |
| **Frankiaceae** | 0.0041 | 0.0006 | 0.0069 | 0.0023 | -2.0747 | 0.1558 | 0.0043 | 0.0004 | 0.0047 | 0.0010 | -0.5543 | 0.6249 | 0.0062 | 0.0021 | 0.0028 | 0.0007 | 2.6639 | 0.0927 |
| **Fusobacteriaceae** | 0.2480 | 0.0201 | 0.2431 | 0.0300 | 0.2380 | 0.8252 | 0.2670 | 0.0260 | 0.2111 | 0.0170 | 3.1163 | 0.0435 | 0.2711 | 0.0370 | 0.1650 | 0.0522 | 2.8690 | 0.0515 |
| **Gallionellaceae** | 0.0021 | 0.0005 | 0.0028 | 0.0003 | -1.9889 | 0.1275 | 0.0029 | 0.0014 | 0.0028 | 0.0035 | 0.0569 | 0.9586 | 0.0032 | 0.0013 | 0.0016 | 0.0007 | 1.7894 | 0.1666 |
| **Geobacteraceae** | 0.0396 | 0.0048 | 0.0362 | 0.0050 | 0.8657 | 0.4356 | 0.0358 | 0.0029 | 0.0354 | 0.0079 | 0.0804 | 0.9419 | 0.0424 | 0.0045 | 0.0349 | 0.0095 | 1.2191 | 0.3138 |
| **Geodermatophilaceae** | 0.0011 | 0.0006 | 0.0005 | 0.0003 | 1.5626 | 0.2270 | 0.0008 | 0.0004 | 0.0008 | 0.0002 | 0.1596 | 0.8824 | 0.0013 | 0.0006 | 0.0004 | 0.0002 | 2.4012 | 0.1146 |
| **Glycomycetaceae** | 0.0013 | 0.0003 | 0.0013 | 0.0005 | -0.0927 | 0.9314 | 0.0019 | 0.0007 | 0.0014 | 0.0011 | 0.6024 | 0.5847 | 0.0010 | 0.0005 | 0.0004 | 0.0001 | 1.9625 | 0.1791 |
| **Gordoniaceae** | 0.0008 | 0.0006 | 0.0007 | 0.0006 | 0.1954 | 0.8546 | 0.0016 | 0.0008 | 0.0004 | 0.0001 | 2.5034 | 0.1230 | 0.0009 | 0.0008 | 0.0004 | 0.0002 | 0.9540 | 0.4306 |
| **Hahellaceae** | 0.0027 | 0.0021 | 0.0035 | 0.0010 | -0.5755 | 0.6077 | 0.0032 | 0.0012 | 0.0031 | 0.0008 | 0.0306 | 0.9773 | 0.0031 | 0.0009 | 0.0022 | 0.0014 | 0.9473 | 0.4065 |
| **Halanaerobiaceae** | 0.0280 | 0.0031 | 0.0293 | 0.0069 | -0.2894 | 0.7924 | 0.0278 | 0.0053 | 0.0200 | 0.0058 | 1.7407 | 0.1574 | 0.0408 | 0.0085 | 0.0158 | 0.0042 | 4.5924 | 0.0207 |
| **Haliangiaceae** | 0.0049 | 0.0029 | 0.0036 | 0.0025 | 0.6089 | 0.5759 | 0.0043 | 0.0031 | 0.0046 | 0.0037 | -0.1042 | 0.9222 | 0.0026 | 0.0018 | 0.0021 | 0.0011 | 0.4657 | 0.6702 |
| **Halobacteroidaceae** | 0.0064 | 0.0019 | 0.0088 | 0.0034 | -1.0973 | 0.3488 | 0.0072 | 0.0021 | 0.0072 | 0.0013 | -0.0204 | 0.9848 | 0.0107 | 0.0015 | 0.0034 | 0.0001 | 8.1360 | 0.0142 |
| **Halomonadaceae** | 0.0033 | 0.0011 | 0.0021 | 0.0011 | 1.3457 | 0.2497 | 0.0023 | 0.0019 | 0.0025 | 0.0002 | -0.1744 | 0.8773 | 0.0027 | 0.0021 | 0.0040 | 0.0008 | -1.0563 | 0.3816 |
| **Halothiobacillaceae** | 0.0102 | 0.0044 | 0.0108 | 0.0035 | -0.1922 | 0.8573 | 0.0077 | 0.0015 | 0.0067 | 0.0028 | 0.5199 | 0.6383 | 0.0120 | 0.0059 | 0.0080 | 0.0024 | 1.1007 | 0.3614 |
| **Helicobacteraceae** | 0.0380 | 0.0001 | 0.0516 | 0.0060 | -3.9288 | 0.0591 | 0.0489 | 0.0151 | 0.0313 | 0.0027 | 1.9776 | 0.1788 | 0.0492 | 0.0164 | 0.0162 | 0.0074 | 3.1692 | 0.0562 |
| **Heliobacteriaceae** | 0.0798 | 0.0140 | 0.1082 | 0.0389 | -1.1858 | 0.3358 | 0.0887 | 0.0199 | 0.0617 | 0.0145 | 1.9044 | 0.1362 | 0.1011 | 0.0346 | 0.0737 | 0.0388 | 0.9116 | 0.4142 |
| **Herpetosiphonaceae** | 0.0100 | 0.0050 | 0.0050 | 0.0034 | 1.4071 | 0.2411 | 0.0068 | 0.0060 | 0.0045 | 0.0058 | 0.4831 | 0.6543 | 0.0142 | 0.0006 | 0.0056 | 0.0032 | 4.4864 | 0.0410 |
| **Hydrogenophilaceae** | 0.0051 | 0.0033 | 0.0031 | 0.0008 | 1.0244 | 0.4038 | 0.0048 | 0.0032 | 0.0061 | 0.0008 | -0.6909 | 0.5540 | 0.0070 | 0.0007 | 0.0045 | 0.0011 | 3.3328 | 0.0405 |
| **Hydrogenothermaceae** | 0.0059 | 0.0002 | 0.0056 | 0.0014 | 0.2885 | 0.7994 | 0.0050 | 0.0016 | 0.0054 | 0.0045 | -0.1704 | 0.8773 | 0.0091 | 0.0016 | 0.0044 | 0.0015 | 3.6191 | 0.0225 |
| **Hyphomicrobiaceae** | 0.0035 | 0.0029 | 0.0035 | 0.0012 | -0.0121 | 0.9912 | 0.0032 | 0.0016 | 0.0024 | 0.0012 | 0.7183 | 0.5149 | 0.0018 | 0.0005 | 0.0032 | 0.0004 | -3.7820 | 0.0197 |
| **Hyphomonadaceae** | 0.0071 | 0.0028 | 0.0069 | 0.0030 | 0.0615 | 0.9539 | 0.0085 | 0.0023 | 0.0073 | 0.0017 | 0.7175 | 0.5155 | 0.0089 | 0.0038 | 0.0058 | 0.0007 | 1.3628 | 0.2985 |
| **Idiomarinaceae** | 0.0049 | 0.0005 | 0.0076 | 0.0023 | -2.0340 | 0.1674 | 0.0050 | 0.0005 | 0.0022 | 0.0004 | 8.0488 | 0.0017 | 0.0061 | 0.0017 | 0.0033 | 0.0009 | 2.4369 | 0.0903 |
| **Intrasporangiaceae** | 0.0042 | 0.0047 | 0.0028 | 0.0018 | 0.4899 | 0.6632 | 0.0053 | 0.0005 | 0.0044 | 0.0013 | 1.0940 | 0.3689 | 0.0045 | 0.0054 | 0.0044 | 0.0021 | 0.0368 | 0.9733 |
| **Jonesiaceae** | 0.0021 | 0.0013 | 0.0037 | 0.0020 | -1.2130 | 0.3010 | 0.0031 | 0.0025 | 0.0027 | 0.0022 | 0.1865 | 0.8612 | 0.0024 | 0.0016 | 0.0008 | 0.0005 | 1.5743 | 0.2360 |
| **Kineosporiaceae** | 0.0017 | 0.0006 | 0.0019 | 0.0009 | -0.3335 | 0.7572 | 0.0017 | 0.0004 | 0.0015 | 0.0008 | 0.4567 | 0.6814 | 0.0021 | 0.0003 | 0.0014 | 0.0004 | 2.3961 | 0.0778 |
| **Ktedonobacteraceae** | 0.0026 | 0.0012 | 0.0041 | 0.0026 | -0.8675 | 0.4526 | 0.0030 | 0.0010 | 0.0014 | 0.0003 | 2.5927 | 0.1031 | 0.0028 | 0.0012 | 0.0008 | 0.0006 | 2.5467 | 0.0865 |
| **Lachnospiraceae** | 14.0439 | 1.9700 | 15.2176 | 3.1440 | -0.5479 | 0.6181 | 16.9959 | 0.6384 | 10.2421 | 1.9308 | 5.7524 | 0.0181 | 14.6498 | 0.2130 | 9.8551 | 3.9036 | 2.1243 | 0.1668 |
| **Lactobacillaceae** | 1.1418 | 0.5876 | 1.1632 | 0.4939 | -0.0482 | 0.9639 | 2.3991 | 0.2986 | 12.5788 | 8.7755 | -2.0080 | 0.1821 | 1.1354 | 0.5371 | 4.6283 | 2.2323 | -2.6350 | 0.1062 |
| **Legionellaceae** | 0.0023 | 0.0009 | 0.0021 | 0.0010 | 0.3243 | 0.7622 | 0.0030 | 0.0020 | 0.0026 | 0.0019 | 0.2076 | 0.8457 | 0.0020 | 0.0012 | 0.0031 | 0.0020 | -0.8325 | 0.4607 |
| **Lentisphaeraceae** | 0.0031 | 0.0010 | 0.0046 | 0.0025 | -0.9796 | 0.4084 | 0.0033 | 0.0011 | 0.0056 | 0.0025 | -1.4861 | 0.2409 | 0.0047 | 0.0017 | 0.0046 | 0.0026 | 0.0488 | 0.9638 |
| **Leptospiraceae** | 0.0114 | 0.0038 | 0.0128 | 0.0009 | -0.6234 | 0.5905 | 0.0122 | 0.0013 | 0.0128 | 0.0015 | -0.5612 | 0.6052 | 0.0104 | 0.0045 | 0.0142 | 0.0062 | -0.8646 | 0.4407 |
| **Leuconostocaceae** | 0.0274 | 0.0052 | 0.0242 | 0.0035 | 0.8606 | 0.4449 | 0.0264 | 0.0068 | 0.0570 | 0.0270 | -1.9045 | 0.1825 | 0.0347 | 0.0074 | 0.1431 | 0.0182 | -9.5495 | 0.0040 |
| **Listeriaceae** | 0.0651 | 0.0091 | 0.0720 | 0.0124 | -0.7707 | 0.4876 | 0.0763 | 0.0045 | 0.0962 | 0.0266 | -1.2803 | 0.3229 | 0.0736 | 0.0020 | 0.1769 | 0.0256 | -6.9698 | 0.0194 |
| **Mariprofundaceae** | 0.0017 | 0.0010 | 0.0009 | 0.0001 | 1.3066 | 0.3168 | 0.0014 | 0.0009 | 0.0007 | 0.0003 | 1.2084 | 0.3269 | 0.0026 | 0.0027 | 0.0010 | 0.0004 | 1.0838 | 0.3868 |
| **Methylacidiphilaceae** | 0.0043 | 0.0030 | 0.0010 | 0.0007 | 1.8428 | 0.1954 | 0.0013 | 0.0008 | 0.0039 | 0.0015 | -2.5981 | 0.0793 | 0.0011 | 0.0010 | 0.0020 | 0.0013 | -0.9609 | 0.3949 |
| **Methylobacteriaceae** | 0.0062 | 0.0020 | 0.0038 | 0.0015 | 1.6248 | 0.1845 | 0.0046 | 0.0013 | 0.0040 | 0.0013 | 0.5494 | 0.6120 | 0.0058 | 0.0031 | 0.0059 | 0.0030 | -0.0078 | 0.9941 |
| **Methylococcaceae** | 0.0052 | 0.0003 | 0.0071 | 0.0002 | -8.9534 | 0.0021 | 0.0069 | 0.0019 | 0.0074 | 0.0048 | -0.1510 | 0.8908 | 0.0054 | 0.0001 | 0.0037 | 0.0012 | 2.4714 | 0.1311 |
| **Methylocystaceae** | 0.0008 | 0.0004 | 0.0016 | 0.0003 | -3.0067 | 0.0475 | 0.0009 | 0.0003 | 0.0010 | 0.0008 | -0.3897 | 0.7271 | 0.0011 | 0.0002 | 0.0001 | 0.0001 | 9.1099 | 0.0025 |
| **Methylophilaceae** | 0.0089 | 0.0036 | 0.0090 | 0.0024 | -0.0479 | 0.9644 | 0.0075 | 0.0011 | 0.0070 | 0.0033 | 0.2307 | 0.8354 | 0.0074 | 0.0023 | 0.0053 | 0.0014 | 1.3639 | 0.2577 |
| **Microbacteriaceae** | 0.0014 | 0.0007 | 0.0020 | 0.0005 | -1.1193 | 0.3303 | 0.0013 | 0.0005 | 0.0010 | 0.0006 | 0.5831 | 0.5916 | 0.0021 | 0.0014 | 0.0005 | 0.0002 | 1.8767 | 0.1964 |
| **Microchaetaceae** | 0.0000 | 0.0000 | 0.0000 | 0.0000 | NA | NA | 0.0000 | 0.0000 | 0.0000 | 0.0000 | NA | NA | 0.0000 | 0.0000 | 0.0000 | 0.0000 | NA | NA |
| **Micrococcaceae** | 0.0296 | 0.0076 | 0.0241 | 0.0064 | 0.9559 | 0.3947 | 0.0336 | 0.0029 | 0.0189 | 0.0030 | 6.0250 | 0.0038 | 0.0387 | 0.0097 | 0.0178 | 0.0046 | 3.3730 | 0.0464 |
| **Micromonosporaceae** | 0.0063 | 0.0005 | 0.0036 | 0.0012 | 3.6050 | 0.0434 | 0.0094 | 0.0028 | 0.0062 | 0.0011 | 1.8867 | 0.1707 | 0.0068 | 0.0015 | 0.0042 | 0.0011 | 2.4330 | 0.0768 |
| **Moraxellaceae** | 0.0667 | 0.0233 | 0.0532 | 0.0158 | 0.8339 | 0.4571 | 0.0770 | 0.0110 | 0.0416 | 0.0082 | 4.4588 | 0.0134 | 0.0905 | 0.0145 | 0.0374 | 0.0062 | 5.8293 | 0.0133 |
| **Moritellaceae** | 0.0012 | 0.0007 | 0.0019 | 0.0014 | -0.7312 | 0.5178 | 0.0009 | 0.0004 | 0.0005 | 0.0002 | 1.3165 | 0.2793 | 0.0033 | 0.0044 | 0.0003 | 0.0000 | 1.1998 | 0.3531 |
| **Mycobacteriaceae** | 0.0083 | 0.0019 | 0.0076 | 0.0008 | 0.5253 | 0.6392 | 0.0107 | 0.0065 | 0.0087 | 0.0015 | 0.5225 | 0.6490 | 0.0093 | 0.0007 | 0.0067 | 0.0010 | 3.6586 | 0.0249 |
| **Mycoplasmataceae** | 0.0154 | 0.0015 | 0.0188 | 0.0016 | -2.6617 | 0.0566 | 0.0130 | 0.0029 | 0.0112 | 0.0040 | 0.6239 | 0.5699 | 0.0187 | 0.0031 | 0.0075 | 0.0022 | 5.1896 | 0.0086 |
| **Myxococcaceae** | 0.0183 | 0.0045 | 0.0131 | 0.0070 | 1.0860 | 0.3481 | 0.0182 | 0.0084 | 0.0174 | 0.0033 | 0.1512 | 0.8907 | 0.0157 | 0.0030 | 0.0242 | 0.0145 | -0.9908 | 0.4191 |
| **NAkamurellaceae** | 0.0025 | 0.0011 | 0.0017 | 0.0010 | 0.9838 | 0.3823 | 0.0024 | 0.0010 | 0.0012 | 0.0003 | 2.0555 | 0.1537 | 0.0038 | 0.0022 | 0.0007 | 0.0007 | 2.3962 | 0.1191 |
| **NAnnocystaceae** | 0.0013 | 0.0010 | 0.0003 | 0.0003 | 1.6511 | 0.2211 | 0.0003 | 0.0002 | 0.0005 | 0.0001 | -1.5114 | 0.2072 | 0.0003 | 0.0003 | 0.0005 | 0.0003 | -0.5491 | 0.6122 |
| **NAtranaerobiaceae** | 0.0123 | 0.0019 | 0.0143 | 0.0049 | -0.6458 | 0.5709 | 0.0156 | 0.0006 | 0.0107 | 0.0028 | 2.9724 | 0.0862 | 0.0182 | 0.0056 | 0.0051 | 0.0007 | 4.0347 | 0.0533 |
| **NAutiliaceae** | 0.0034 | 0.0023 | 0.0053 | 0.0030 | -0.8745 | 0.4337 | 0.0047 | 0.0014 | 0.0038 | 0.0022 | 0.5941 | 0.5892 | 0.0065 | 0.0010 | 0.0031 | 0.0031 | 1.8097 | 0.1909 |
| **Neisseriaceae** | 0.0509 | 0.0083 | 0.0509 | 0.0178 | 0.0008 | 0.9994 | 0.0349 | 0.0039 | 0.0449 | 0.0058 | -2.4584 | 0.0787 | 0.0562 | 0.0157 | 0.0318 | 0.0020 | 2.6733 | 0.1122 |
| **Nitrosomonadaceae** | 0.0108 | 0.0032 | 0.0183 | 0.0055 | -2.0302 | 0.1289 | 0.0144 | 0.0035 | 0.0149 | 0.0007 | -0.2410 | 0.8304 | 0.0101 | 0.0036 | 0.0095 | 0.0035 | 0.2154 | 0.8400 |
| **Nitrospiraceae** | 0.0068 | 0.0030 | 0.0037 | 0.0013 | 1.6560 | 0.2048 | 0.0083 | 0.0027 | 0.0026 | 0.0007 | 3.5291 | 0.0584 | 0.0080 | 0.0039 | 0.0075 | 0.0068 | 0.1053 | 0.9225 |
| **Nocardiaceae** | 0.0107 | 0.0054 | 0.0066 | 0.0016 | 1.2530 | 0.3205 | 0.0103 | 0.0034 | 0.0057 | 0.0011 | 2.2747 | 0.1282 | 0.0083 | 0.0013 | 0.0081 | 0.0025 | 0.1357 | 0.9008 |
| **Nocardioidaceae** | 0.0062 | 0.0020 | 0.0059 | 0.0025 | 0.1758 | 0.8693 | 0.0073 | 0.0019 | 0.0054 | 0.0009 | 1.6266 | 0.2033 | 0.0075 | 0.0025 | 0.0038 | 0.0010 | 2.3589 | 0.1134 |
| **Nocardiopsaceae** | 0.0041 | 0.0015 | 0.0044 | 0.0023 | -0.1982 | 0.8542 | 0.0043 | 0.0017 | 0.0031 | 0.0020 | 0.8267 | 0.4565 | 0.0032 | 0.0008 | 0.0040 | 0.0032 | -0.3882 | 0.7318 |
| **Nostocaceae** | 0.0136 | 0.0104 | 0.0116 | 0.0019 | 0.3258 | 0.7738 | 0.0098 | 0.0008 | 0.0075 | 0.0024 | 1.5702 | 0.2339 | 0.0130 | 0.0053 | 0.0078 | 0.0034 | 1.4556 | 0.2311 |

**Supplemental Table 3: *t-test* for Equality of Means of Bacterial Family Continued**

Dependent Variable: Diet

|  | **Day 0** | | | | | | **2 Days PD** | | | | | | **2 Weeks PD** | | | | | |
| --- | --- | --- | --- | --- | --- | --- | --- | --- | --- | --- | --- | --- | --- | --- | --- | --- | --- | --- |
|  | **Chow** | | **WD** | | **F** | **P value** | **Chow** | | **WD** | | **F** | **P value** | **Chow** | | **WD** | | **F** | **P value** |
|  | **Mean** | **SD** | **Mean** | **SD** |  |  | **Mean** | **SD** | **Mean** | **SD** |  |  | **Mean** | **SD** | **Mean** | **SD** |  |  |
| **Oceanospirillaceae** | 0.0042 | 0.0017 | 0.0045 | 0.0016 | -0.1688 | 0.8742 | 0.0044 | 0.0013 | 0.0048 | 0.0017 | -0.2649 | 0.8052 | 0.0062 | 0.0029 | 0.0049 | 0.0022 | 0.6296 | 0.5659 |
| **Opitutaceae** | 0.0100 | 0.0020 | 0.0145 | 0.0067 | -1.1135 | 0.3661 | 0.0101 | 0.0019 | 0.0122 | 0.0005 | -1.8861 | 0.1844 | 0.0085 | 0.0045 | 0.0127 | 0.0040 | -1.2199 | 0.2902 |
| **Oscillochloridaceae** | 0.0006 | 0.0002 | 0.0015 | 0.0002 | -5.5228 | 0.0061 | 0.0012 | 0.0003 | 0.0007 | 0.0002 | 2.5786 | 0.0809 | 0.0013 | 0.0004 | 0.0003 | 0.0003 | 3.2092 | 0.0389 |
| **Oxalobacteraceae** | 0.0173 | 0.0021 | 0.0178 | 0.0064 | -0.1121 | 0.9194 | 0.0205 | 0.0012 | 0.0160 | 0.0044 | 1.6836 | 0.2183 | 0.0291 | 0.0075 | 0.0134 | 0.0073 | 2.5988 | 0.0602 |
| **Paenibacillaceae** | 0.1008 | 0.0187 | 0.1242 | 0.0055 | -2.0809 | 0.1539 | 0.1193 | 0.0112 | 0.0860 | 0.0056 | 4.6025 | 0.0199 | 0.1209 | 0.0083 | 0.0790 | 0.0309 | 2.2667 | 0.1356 |
| **Parachlamydiaceae** | 0.0025 | 0.0026 | 0.0010 | 0.0012 | 0.8596 | 0.4584 | 0.0017 | 0.0004 | 0.0014 | 0.0010 | 0.4101 | 0.7106 | 0.0020 | 0.0024 | 0.0001 | 0.0001 | 1.3255 | 0.3157 |
| **Parvularculaceae** | 0.0006 | 0.0002 | 0.0014 | 0.0014 | -1.0081 | 0.4166 | 0.0020 | 0.0012 | 0.0008 | 0.0003 | 1.7278 | 0.2143 | 0.0015 | 0.0003 | 0.0018 | 0.0012 | -0.4738 | 0.6765 |
| **Pasteurellaceae** | 0.0663 | 0.0116 | 0.0580 | 0.0087 | 0.9960 | 0.3798 | 0.0712 | 0.0149 | 0.0562 | 0.0187 | 1.0880 | 0.3406 | 0.0746 | 0.0094 | 0.0545 | 0.0074 | 2.9194 | 0.0464 |
| **Pasteuriaceae** | 0.0000 | 0.0000 | 0.0000 | 0.0000 | NA | NA | 0.0000 | 0.0000 | 0.0000 | 0.0000 | NA | NA | 0.0000 | 0.0000 | 0.0000 | 0.0000 | NA | NA |
| **Pelobacteraceae** | 0.0260 | 0.0057 | 0.0199 | 0.0066 | 1.1997 | 0.2978 | 0.0203 | 0.0036 | 0.0163 | 0.0043 | 1.2431 | 0.2839 | 0.0235 | 0.0015 | 0.0192 | 0.0043 | 1.6289 | 0.2200 |
| **Peptococcaceae** | 0.2259 | 0.0394 | 0.2345 | 0.0280 | -0.3054 | 0.7768 | 0.2604 | 0.0297 | 0.1636 | 0.0139 | 5.1101 | 0.0167 | 0.2251 | 0.0291 | 0.1104 | 0.0118 | 6.3229 | 0.0116 |
| **Peptostreptococcaceae** | 0.0592 | 0.0075 | 0.0652 | 0.0049 | -1.1627 | 0.3188 | 0.0750 | 0.0106 | 0.0454 | 0.0060 | 4.2079 | 0.0221 | 0.0708 | 0.0141 | 0.0370 | 0.0024 | 4.0995 | 0.0496 |
| **Phyllobacteriaceae** | 0.0097 | 0.0042 | 0.0094 | 0.0021 | 0.1105 | 0.9191 | 0.0085 | 0.0023 | 0.0076 | 0.0032 | 0.3935 | 0.7158 | 0.0063 | 0.0009 | 0.0052 | 0.0021 | 0.7863 | 0.4939 |
| **Piscirickettsiaceae** | 0.0054 | 0.0032 | 0.0024 | 0.0021 | 1.3516 | 0.2582 | 0.0021 | 0.0005 | 0.0012 | 0.0001 | 2.7841 | 0.0950 | 0.0030 | 0.0013 | 0.0029 | 0.0014 | 0.1484 | 0.8893 |
| **Planctomycetaceae** | 0.0116 | 0.0058 | 0.0153 | 0.0027 | -0.9908 | 0.3991 | 0.0096 | 0.0023 | 0.0100 | 0.0032 | -0.1650 | 0.8777 | 0.0161 | 0.0066 | 0.0071 | 0.0045 | 1.9605 | 0.1310 |
| **Planococcaceae** | 0.0035 | 0.0023 | 0.0043 | 0.0019 | -0.4902 | 0.6502 | 0.0041 | 0.0025 | 0.0014 | 0.0002 | 1.9378 | 0.1911 | 0.0026 | 0.0006 | 0.0011 | 0.0006 | 3.0702 | 0.0373 |
| **Polyangiaceae** | 0.0000 | 0.0000 | 0.0000 | 0.0000 | NA | NA | 0.0000 | 0.0000 | 0.0000 | 0.0000 | NA | NA | 0.0000 | 0.0000 | 0.0000 | 0.0000 | NA | NA |
| **Porphyromonadaceae** | 4.1280 | 0.6227 | 3.1064 | 1.6669 | 0.9945 | 0.4049 | 2.3345 | 0.3571 | 3.0296 | 0.6395 | -1.6436 | 0.1948 | 4.6598 | 0.3982 | 3.0886 | 0.8200 | 2.9855 | 0.0611 |
| **Prevotellaceae** | 3.0185 | 0.5047 | 2.4260 | 1.2389 | 0.7671 | 0.5056 | 1.8946 | 0.2479 | 2.3259 | 0.5112 | -1.3148 | 0.2831 | 3.6192 | 0.2236 | 2.3329 | 0.7139 | 2.9783 | 0.0779 |
| **Prochlorococcaceae** | 0.0045 | 0.0011 | 0.0089 | 0.0046 | -1.6441 | 0.2291 | 0.0080 | 0.0024 | 0.0050 | 0.0008 | 2.0065 | 0.1594 | 0.0068 | 0.0015 | 0.0042 | 0.0015 | 2.1847 | 0.0942 |
| **Promicromonosporaceae** | 0.0027 | 0.0018 | 0.0059 | 0.0068 | -0.7673 | 0.5145 | 0.0018 | 0.0004 | 0.0020 | 0.0018 | -0.2056 | 0.8546 | 0.0021 | 0.0013 | 0.0007 | 0.0004 | 1.8011 | 0.1960 |
| **Propionibacteriaceae** | 0.0083 | 0.0045 | 0.0063 | 0.0039 | 0.5833 | 0.5915 | 0.0126 | 0.0047 | 0.0057 | 0.0006 | 2.5151 | 0.1247 | 0.0131 | 0.0066 | 0.0055 | 0.0001 | 2.0156 | 0.1813 |
| **Pseudoalteromonadaceae** | 0.0061 | 0.0019 | 0.0065 | 0.0031 | -0.2250 | 0.8350 | 0.0037 | 0.0011 | 0.0059 | 0.0017 | -1.8511 | 0.1491 | 0.0054 | 0.0008 | 0.0035 | 0.0017 | 1.7274 | 0.1842 |
| **Pseudomonadaceae** | 0.3660 | 0.1094 | 0.2772 | 0.0913 | 1.0789 | 0.3432 | 0.3882 | 0.0463 | 0.1767 | 0.0367 | 6.2039 | 0.0041 | 0.4447 | 0.0598 | 0.1576 | 0.0102 | 8.1992 | 0.0123 |
| **Pseudonocardiaceae** | 0.0030 | 0.0004 | 0.0049 | 0.0014 | -2.2306 | 0.1401 | 0.0066 | 0.0019 | 0.0057 | 0.0051 | 0.2766 | 0.8030 | 0.0031 | 0.0004 | 0.0038 | 0.0028 | -0.4556 | 0.6916 |
| **Psychromonadaceae** | 0.0024 | 0.0012 | 0.0030 | 0.0002 | -0.8593 | 0.4748 | 0.0028 | 0.0008 | 0.0037 | 0.0020 | -0.7234 | 0.5288 | 0.0032 | 0.0022 | 0.0027 | 0.0005 | 0.4016 | 0.7241 |
| **Puniceicoccaceae** | 0.0040 | 0.0031 | 0.0061 | 0.0040 | -0.7470 | 0.4990 | 0.0023 | 0.0007 | 0.0087 | 0.0015 | -6.7514 | 0.0073 | 0.0077 | 0.0028 | 0.0119 | 0.0097 | -0.7275 | 0.5332 |
| **Rhizobiaceae** | 0.0170 | 0.0054 | 0.0224 | 0.0095 | -0.8499 | 0.4546 | 0.0152 | 0.0019 | 0.0143 | 0.0032 | 0.3961 | 0.7166 | 0.0159 | 0.0014 | 0.0109 | 0.0015 | 4.0981 | 0.0150 |
| **Rhodobacteraceae** | 0.0399 | 0.0012 | 0.0319 | 0.0057 | 2.3570 | 0.1316 | 0.0349 | 0.0068 | 0.0271 | 0.0009 | 1.9720 | 0.1831 | 0.0393 | 0.0012 | 0.0241 | 0.0048 | 5.3471 | 0.0252 |
| **Rhodobiaceae** | 0.0000 | 0.0000 | 0.0000 | 0.0000 | NA | NA | 0.0000 | 0.0000 | 0.0001 | 0.0001 | -1.9858 | 0.1854 | 0.0000 | 0.0000 | 0.0000 | 0.0000 | NA | NA |
| **Rhodocyclaceae** | 0.0117 | 0.0045 | 0.0072 | 0.0016 | 1.6455 | 0.2172 | 0.0062 | 0.0017 | 0.0069 | 0.0006 | -0.6755 | 0.5573 | 0.0118 | 0.0016 | 0.0061 | 0.0013 | 4.7669 | 0.0097 |
| **Rhodospirillaceae** | 0.0093 | 0.0016 | 0.0069 | 0.0023 | 1.4549 | 0.2268 | 0.0108 | 0.0027 | 0.0066 | 0.0019 | 2.1567 | 0.1057 | 0.0072 | 0.0029 | 0.0046 | 0.0011 | 1.4688 | 0.2531 |
| **Rhodothermaceae** | 0.0167 | 0.0121 | 0.0091 | 0.0054 | 0.9910 | 0.4003 | 0.0115 | 0.0054 | 0.0120 | 0.0031 | -0.1412 | 0.8962 | 0.0126 | 0.0046 | 0.0078 | 0.0032 | 1.4617 | 0.2249 |
| **Rickettsiaceae** | 0.0049 | 0.0026 | 0.0069 | 0.0017 | -1.1217 | 0.3344 | 0.0061 | 0.0024 | 0.0045 | 0.0017 | 0.9433 | 0.4055 | 0.0063 | 0.0025 | 0.0036 | 0.0018 | 1.5628 | 0.2011 |
| **Rikenellaceae** | 8.1655 | 8.8954 | 4.0310 | 6.2726 | 0.6579 | 0.5503 | 1.2517 | 1.2639 | 5.1421 | 6.6034 | -1.0022 | 0.4156 | 1.0081 | 0.1092 | 1.0428 | 0.3470 | -0.1653 | 0.8816 |
| **Rubrobacteraceae** | 0.0046 | 0.0030 | 0.0049 | 0.0028 | -0.1177 | 0.9120 | 0.0049 | 0.0051 | 0.0051 | 0.0004 | -0.0464 | 0.9671 | 0.0049 | 0.0004 | 0.0026 | 0.0015 | 2.4866 | 0.1148 |
| **Ruminococcaceae** | 8.5980 | 1.4372 | 10.4467 | 2.2116 | -1.2140 | 0.3016 | 11.4740 | 0.6013 | 6.8634 | 1.1913 | 5.9843 | 0.0097 | 9.9516 | 0.1398 | 6.1887 | 1.5041 | 4.3145 | 0.0482 |
| **Sanguibacteraceae** | 0.0027 | 0.0015 | 0.0030 | 0.0023 | -0.1793 | 0.8680 | 0.0042 | 0.0018 | 0.0011 | 0.0003 | 2.8318 | 0.0979 | 0.0033 | 0.0011 | 0.0016 | 0.0006 | 2.4459 | 0.0922 |
| **Segniliparaceae** | 0.0003 | 0.0003 | 0.0005 | 0.0003 | -0.8482 | 0.4442 | 0.0005 | 0.0001 | 0.0003 | 0.0003 | 1.1608 | 0.3537 | 0.0008 | 0.0008 | 0.0003 | 0.0002 | 1.0725 | 0.3854 |
| **Shewanellaceae** | 0.0285 | 0.0024 | 0.0320 | 0.0034 | -1.4623 | 0.2256 | 0.0302 | 0.0107 | 0.0256 | 0.0063 | 0.6389 | 0.5653 | 0.0289 | 0.0078 | 0.0238 | 0.0068 | 0.8483 | 0.4449 |
| **Simkaniaceae** | 0.0000 | 0.0000 | 0.0000 | 0.0000 | NA | NA | 0.0000 | 0.0000 | 0.0000 | 0.0000 | NA | NA | 0.0000 | 0.0000 | 0.0000 | 0.0000 | NA | NA |
| **Solibacteraceae** | 0.0037 | 0.0010 | 0.0095 | 0.0028 | -3.3834 | 0.0558 | 0.0053 | 0.0008 | 0.0071 | 0.0028 | -1.0620 | 0.3860 | 0.0065 | 0.0008 | 0.0077 | 0.0070 | -0.2936 | 0.7960 |
| **Sphaerobacteraceae** | 0.0035 | 0.0013 | 0.0043 | 0.0016 | -0.6300 | 0.5651 | 0.0035 | 0.0007 | 0.0024 | 0.0010 | 1.5396 | 0.2058 | 0.0032 | 0.0004 | 0.0010 | 0.0003 | 7.1234 | 0.0034 |
| **Sphingobacteriaceae** | 0.1614 | 0.0057 | 0.1160 | 0.0644 | 1.2174 | 0.3460 | 0.0932 | 0.0146 | 0.1138 | 0.0470 | -0.7249 | 0.5331 | 0.1728 | 0.0124 | 0.0957 | 0.0188 | 5.9418 | 0.0063 |
| **Sphingomonadaceae** | 0.0093 | 0.0020 | 0.0068 | 0.0009 | 2.0444 | 0.1398 | 0.0109 | 0.0026 | 0.0077 | 0.0035 | 1.2772 | 0.2750 | 0.0102 | 0.0044 | 0.0083 | 0.0015 | 0.6980 | 0.5449 |
| **Spirochaetaceae** | 0.1097 | 0.0447 | 0.1206 | 0.0270 | -0.3607 | 0.7402 | 0.1124 | 0.0188 | 0.0981 | 0.0180 | 0.9536 | 0.3944 | 0.1303 | 0.0275 | 0.0633 | 0.0233 | 3.2230 | 0.0334 |
| **Spiroplasmataceae** | 0.0008 | 0.0007 | 0.0007 | 0.0005 | 0.3044 | 0.7783 | 0.0016 | 0.0007 | 0.0016 | 0.0012 | 0.0269 | 0.9801 | 0.0007 | 0.0003 | 0.0013 | 0.0011 | -0.8585 | 0.4699 |
| **Sporolactobacillaceae** | 0.0000 | 0.0000 | 0.0000 | 0.0000 | NA | NA | 0.0000 | 0.0000 | 0.0000 | 0.0000 | NA | NA | 0.0000 | 0.0000 | 0.0000 | 0.0000 | NA | NA |
| **Staphylococcaceae** | 0.0301 | 0.0038 | 0.0385 | 0.0019 | -3.4593 | 0.0412 | 0.0337 | 0.0040 | 0.0332 | 0.0070 | 0.1263 | 0.9071 | 0.0343 | 0.0088 | 0.0352 | 0.0135 | -0.0935 | 0.9307 |
| **Streptococcaceae** | 1.3136 | 0.2291 | 1.6920 | 0.5944 | -1.0288 | 0.3902 | 1.7527 | 0.1921 | 4.5598 | 2.1846 | -2.2170 | 0.1550 | 1.2595 | 0.3045 | 14.5521 | 1.9181 | -11.8547 | 0.0059 |
| **Streptomycetaceae** | 0.0092 | 0.0037 | 0.0089 | 0.0009 | 0.1279 | 0.9087 | 0.0121 | 0.0007 | 0.0069 | 0.0023 | 3.6961 | 0.0502 | 0.0107 | 0.0027 | 0.0047 | 0.0015 | 3.3329 | 0.0410 |
| **Streptosporangiaceae** | 0.0020 | 0.0010 | 0.0018 | 0.0010 | 0.2598 | 0.8078 | 0.0019 | 0.0003 | 0.0015 | 0.0011 | 0.6108 | 0.5979 | 0.0022 | 0.0017 | 0.0008 | 0.0003 | 1.4499 | 0.2790 |
| **Succinivibrionaceae** | 0.0256 | 0.0081 | 0.0312 | 0.0097 | -0.7602 | 0.4907 | 0.0319 | 0.0124 | 0.0295 | 0.0105 | 0.2534 | 0.8128 | 0.0356 | 0.0111 | 0.0180 | 0.0057 | 2.4310 | 0.0938 |
| **Synergistaceae** | 0.0700 | 0.0143 | 0.0977 | 0.0260 | -1.6187 | 0.2007 | 0.0907 | 0.0199 | 0.0631 | 0.0121 | 2.0488 | 0.1246 | 0.0987 | 0.0182 | 0.0500 | 0.0191 | 3.1951 | 0.0332 |
| **Syntrophaceae** | 0.0096 | 0.0021 | 0.0119 | 0.0015 | -1.6199 | 0.1878 | 0.0103 | 0.0028 | 0.0070 | 0.0021 | 1.6273 | 0.1834 | 0.0166 | 0.0054 | 0.0080 | 0.0036 | 2.3216 | 0.0908 |
| **Syntrophobacteraceae** | 0.0136 | 0.0040 | 0.0131 | 0.0048 | 0.1367 | 0.8981 | 0.0084 | 0.0082 | 0.0075 | 0.0007 | 0.2038 | 0.8571 | 0.0111 | 0.0037 | 0.0097 | 0.0034 | 0.4804 | 0.6562 |
| **Syntrophomonadaceae** | 0.0503 | 0.0183 | 0.0675 | 0.0205 | -1.0860 | 0.3393 | 0.0535 | 0.0014 | 0.0387 | 0.0075 | 3.3382 | 0.0722 | 0.0662 | 0.0113 | 0.0339 | 0.0118 | 3.4346 | 0.0265 |
| **Thermaceae** | 0.0086 | 0.0032 | 0.0126 | 0.0041 | -1.3221 | 0.2604 | 0.0093 | 0.0019 | 0.0070 | 0.0007 | 1.9590 | 0.1591 | 0.0123 | 0.0018 | 0.0050 | 0.0018 | 5.0762 | 0.0071 |
| **Thermoactinomycetaceae** | 0.0000 | 0.0000 | 0.0000 | 0.0000 | NA | NA | 0.0000 | 0.0000 | 0.0000 | 0.0000 | NA | NA | 0.0000 | 0.0000 | 0.0000 | 0.0000 | NA | NA |
| **Thermoanaerobacteraceae** | 0.2017 | 0.0235 | 0.2343 | 0.0232 | -1.7064 | 0.1631 | 0.2353 | 0.0104 | 0.1595 | 0.0176 | 6.4207 | 0.0061 | 0.2761 | 0.0193 | 0.1049 | 0.0250 | 9.3954 | 0.0010 |
| **Thermoanaerobacterales.Family.III.. Incertae.Sedis** | 0.1590 | 0.0303 | 0.1948 | 0.0295 | -1.4676 | 0.2162 | 0.2061 | 0.0338 | 0.1048 | 0.0182 | 4.5619 | 0.0188 | 0.2226 | 0.0458 | 0.0821 | 0.0308 | 4.4099 | 0.0156 |
| **Thermodesulfobiaceae** | 0.0017 | 0.0005 | 0.0013 | 0.0008 | 0.7980 | 0.4781 | 0.0032 | 0.0020 | 0.0009 | 0.0002 | 1.9964 | 0.1814 | 0.0024 | 0.0004 | 0.0010 | 0.0004 | 4.1565 | 0.0146 |
| **Thermomicrobiaceae** | 0.0017 | 0.0012 | 0.0015 | 0.0009 | 0.2104 | 0.8444 | 0.0017 | 0.0006 | 0.0014 | 0.0007 | 0.5843 | 0.5906 | 0.0032 | 0.0020 | 0.0008 | 0.0003 | 2.0815 | 0.1674 |
| **Thermomonosporaceae** | 0.0009 | 0.0002 | 0.0028 | 0.0028 | -1.1419 | 0.3712 | 0.0021 | 0.0010 | 0.0016 | 0.0012 | 0.5873 | 0.5897 | 0.0025 | 0.0015 | 0.0008 | 0.0007 | 1.7569 | 0.1790 |
| **Thermotogaceae** | 0.0342 | 0.0046 | 0.0324 | 0.0048 | 0.4494 | 0.6765 | 0.0347 | 0.0006 | 0.0240 | 0.0013 | 13.2843 | 0.0013 | 0.0411 | 0.0051 | 0.0194 | 0.0034 | 6.1413 | 0.0057 |
| **Thiotrichaceae** | 0.0014 | 0.0014 | 0.0006 | 0.0002 | 0.9781 | 0.4268 | 0.0013 | 0.0004 | 0.0008 | 0.0007 | 1.0557 | 0.3633 | 0.0007 | 0.0009 | 0.0017 | 0.0010 | -1.2642 | 0.2763 |
| **Trueperaceae** | 0.0032 | 0.0017 | 0.0023 | 0.0010 | 0.7702 | 0.4921 | 0.0024 | 0.0016 | 0.0016 | 0.0006 | 0.8189 | 0.4816 | 0.0018 | 0.0010 | 0.0008 | 0.0007 | 1.4807 | 0.2193 |
| **Tsukamurellaceae** | 0.0056 | 0.0029 | 0.0034 | 0.0032 | 0.9105 | 0.4147 | 0.0059 | 0.0029 | 0.0034 | 0.0029 | 1.0261 | 0.3628 | 0.0048 | 0.0022 | 0.0054 | 0.0029 | -0.2840 | 0.7915 |
| **Veillonellaceae** | 0.3036 | 0.0490 | 0.3614 | 0.0746 | -1.1216 | 0.3339 | 0.3696 | 0.0134 | 0.2214 | 0.0494 | 5.0186 | 0.0281 | 0.3391 | 0.0441 | 0.1729 | 0.0430 | 4.6725 | 0.0095 |
| **Verrucomicrobia.subdivision.3** | 0.0033 | 0.0020 | 0.0038 | 0.0006 | -0.3612 | 0.7484 | 0.0032 | 0.0014 | 0.0079 | 0.0042 | -1.8279 | 0.1841 | 0.0045 | 0.0037 | 0.0074 | 0.0060 | -0.7035 | 0.5279 |
| **Verrucomicrobiaceae** | 1.6850 | 1.8824 | 1.4288 | 2.3377 | 0.1479 | 0.8899 | 0.3760 | 0.5293 | 9.1322 | 4.5470 | -3.3130 | 0.0774 | 0.0945 | 0.0169 | 17.7877 | 17.0592 | -1.7964 | 0.2143 |

**Supplemental Table 3: *t-test* for Equality of Means of Bacterial Family Continued**

Dependent Variable: Diet

|  | **Day 0** | | | | | | **2 Days PD** | | | | | | **2 Weeks PD** | | | | | | |  |
| --- | --- | --- | --- | --- | --- | --- | --- | --- | --- | --- | --- | --- | --- | --- | --- | --- | --- | --- | --- | --- |
|  | **Chow** | | **WD** | | **F** | **P value** | **Chow** | | **WD** | | **F** | **P value** | | **Chow** | | **WD** | | **F** | **P value** | |
|  | **Mean** | **SD** | **Mean** | **SD** |  |  | **Mean** | **SD** | **Mean** | **SD** |  |  | | **Mean** | **SD** | **Mean** | **SD** |  |  |  |
| **Vibrionaceae** | 0.0319 | 0.0027 | 0.0328 | 0.0062 | -0.2197 | 0.8415 | 0.0309 | 0.0029 | 0.0244 | 0.0024 | 3.0141 | 0.0410 | | 0.0388 | 0.0018 | 0.0212 | 0.0015 | 12.7408 | 0.0003 |  |
| **Victivallaceae** | 0.0214 | 0.0073 | 0.0205 | 0.0053 | 0.1858 | 0.8625 | 0.0197 | 0.0053 | 0.0175 | 0.0043 | 0.5716 | 0.5995 | | 0.0318 | 0.0017 | 0.0180 | 0.0042 | 5.2272 | 0.0186 |  |
| **Waddliaceae** | 0.0018 | 0.0008 | 0.0021 | 0.0012 | -0.3119 | 0.7725 | 0.0014 | 0.0007 | 0.0013 | 0.0003 | 0.4123 | 0.7099 | | 0.0010 | 0.0002 | 0.0014 | 0.0013 | -0.5563 | 0.6312 |  |
| **Williamsiaceae** | 0.0000 | 0.0000 | 0.0000 | 0.0000 | NA | NA | 0.0000 | 0.0000 | 0.0000 | 0.0000 | NA | NA | | 0.0000 | 0.0000 | 0.0000 | 0.0000 | NA | NA |  |
| **Xanthobacteraceae** | 0.0031 | 0.0011 | 0.0046 | 0.0025 | -0.9305 | 0.4261 | 0.0050 | 0.0009 | 0.0035 | 0.0018 | 1.3349 | 0.2754 | | 0.0038 | 0.0012 | 0.0017 | 0.0005 | 2.8225 | 0.0755 |  |
| **Xanthomonadaceae** | 0.0211 | 0.0058 | 0.0199 | 0.0061 | 0.2447 | 0.8188 | 0.0162 | 0.0031 | 0.0133 | 0.0025 | 1.2997 | 0.2666 | | 0.0290 | 0.0048 | 0.0157 | 0.0055 | 3.1768 | 0.0346 |  |

|  | **8 Weeks PD** | | | | | | **12 Weeks PD** | | | | | |
| --- | --- | --- | --- | --- | --- | --- | --- | --- | --- | --- | --- | --- |
|  | **Chow** | | **WD** | | **F** | **P value** | **Chow** | | **WD** | | **F** | **P value** |
|  | **Mean** | **SD** | **Mean** | **SD** |  |  | **Mean** | **SD** | **Mean** | **SD** |  |  |
| **Acetobacteraceae** | 0.0086 | 0.0011 | 0.0131 | 0.0031 | -2.4018 | 0.1117 | 0.0094 | 0.0008 | 0.0046 | 0.0022 | 3.5365 | 0.0505 |
| **Acholeplasmataceae** | 0.0563 | 0.0331 | 0.0131 | 0.0064 | 2.2167 | 0.1480 | 0.0350 | 0.0367 | 0.0046 | 0.0015 | 1.4333 | 0.2878 |
| **Acidaminococcaceae** | 0.1870 | 0.0104 | 0.1651 | 0.0721 | 0.5207 | 0.6526 | 0.2011 | 0.0734 | 0.0715 | 0.0077 | 3.0443 | 0.0906 |
| **Acidimicrobiaceae** | 0.0006 | 0.0003 | 0.0004 | 0.0004 | 0.7799 | 0.4790 | 0.0007 | 0.0002 | 0.0002 | 0.0002 | 4.2514 | 0.0131 |
| **Acidithiobacillaceae** | 0.0011 | 0.0004 | 0.0025 | 0.0014 | -1.6926 | 0.2118 | 0.0038 | 0.0018 | 0.0014 | 0.0015 | 1.8422 | 0.1415 |
| **Acidobacteriaceae** | 0.0087 | 0.0027 | 0.0112 | 0.0071 | -0.5544 | 0.6239 | 0.0073 | 0.0021 | 0.0039 | 0.0001 | 2.7458 | 0.1102 |
| **Acidothermaceae** | 0.0027 | 0.0019 | 0.0015 | 0.0013 | 0.9262 | 0.4124 | 0.0020 | 0.0005 | 0.0011 | 0.0006 | 2.1747 | 0.0963 |
| **Actinomycetaceae** | 0.0671 | 0.0186 | 0.0491 | 0.0123 | 1.3883 | 0.2475 | 0.0589 | 0.0243 | 0.0219 | 0.0036 | 2.6046 | 0.1160 |
| **Actinosynnemataceae** | 0.0015 | 0.0008 | 0.0024 | 0.0019 | -0.6820 | 0.5496 | 0.0010 | 0.0004 | 0.0005 | 0.0002 | 1.5984 | 0.2074 |
| **Aerococcaceae** | 0.2851 | 0.0466 | 0.1740 | 0.0419 | 3.0691 | 0.0379 | 0.3009 | 0.1114 | 0.1247 | 0.0084 | 2.7321 | 0.1106 |
| **Aeromonadaceae** | 0.0139 | 0.0023 | 0.0074 | 0.0016 | 4.0320 | 0.0195 | 0.0108 | 0.0018 | 0.0048 | 0.0006 | 5.6137 | 0.0190 |
| **Alcaligenaceae** | 0.0200 | 0.0030 | 0.0284 | 0.0126 | -1.1283 | 0.3658 | 0.0135 | 0.0027 | 0.0060 | 0.0029 | 3.2583 | 0.0313 |
| **Alcanivoracaceae** | 0.0021 | 0.0009 | 0.0017 | 0.0014 | 0.4276 | 0.6938 | 0.0027 | 0.0006 | 0.0005 | 0.0001 | 5.7987 | 0.0233 |
| **Alicyclobacillaceae** | 0.0277 | 0.0049 | 0.0229 | 0.0119 | 0.6352 | 0.5758 | 0.0255 | 0.0063 | 0.0114 | 0.0010 | 3.8265 | 0.0570 |
| **Alteromonadaceae** | 0.0122 | 0.0009 | 0.0226 | 0.0080 | -2.2476 | 0.1503 | 0.0097 | 0.0008 | 0.0051 | 0.0013 | 5.2819 | 0.0109 |
| **Anaeroplasmataceae** | 0.0058 | 0.0040 | 0.0006 | 0.0009 | 2.2466 | 0.1427 | 0.0021 | 0.0030 | 0.0000 | 0.0000 | 1.2443 | 0.3394 |
| **Anaplasmataceae** | 0.0046 | 0.0020 | 0.0026 | 0.0002 | 1.7428 | 0.2198 | 0.0035 | 0.0006 | 0.0015 | 0.0003 | 5.0115 | 0.0204 |
| **Aquificaceae** | 0.0069 | 0.0018 | 0.0067 | 0.0037 | 0.0983 | 0.9282 | 0.0082 | 0.0012 | 0.0034 | 0.0012 | 4.8416 | 0.0084 |
| **Aurantimonadaceae** | 0.0014 | 0.0008 | 0.0014 | 0.0010 | 0.1083 | 0.9192 | 0.0037 | 0.0002 | 0.0006 | 0.0002 | 18.4673 | 0.0002 |
| **Bacillaceae** | 1.3897 | 0.4255 | 1.3150 | 0.6280 | 0.1705 | 0.8740 | 2.6520 | 0.8653 | 4.3943 | 0.0897 | -3.4686 | 0.0718 |
| **Bacteriovoracaceae** | 0.0004 | 0.0004 | 0.0001 | 0.0001 | 1.3064 | 0.2988 | 0.0004 | 0.0004 | 0.0003 | 0.0003 | 0.2446 | 0.8192 |
| **Bacteroidaceae** | 15.7996 | 1.2619 | 20.5114 | 7.0160 | -1.1448 | 0.3646 | 12.1226 | 1.2892 | 5.7424 | 0.6423 | 7.6726 | 0.0050 |
| **Bartonellaceae** | 0.0014 | 0.0007 | 0.0013 | 0.0015 | 0.1377 | 0.8998 | 0.0015 | 0.0002 | 0.0005 | 0.0001 | 7.4950 | 0.0042 |
| **Bdellovibrionaceae** | 0.0001 | 0.0001 | 0.0000 | 0.0001 | 0.3102 | 0.7746 | 0.0000 | 0.0001 | 0.0000 | 0.0000 | 0.5547 | 0.6227 |
| **Beijerinckiaceae** | 0.0046 | 0.0013 | 0.0016 | 0.0007 | 3.5487 | 0.0365 | 0.0032 | 0.0006 | 0.0011 | 0.0001 | 6.2696 | 0.0197 |
| **Beutenbergiaceae** | 0.0015 | 0.0006 | 0.0012 | 0.0003 | 0.7125 | 0.5263 | 0.0035 | 0.0027 | 0.0007 | 0.0002 | 1.7955 | 0.2132 |
| **Bifidobacteriaceae** | 0.6624 | 0.7675 | 0.5214 | 0.5459 | 0.2592 | 0.8096 | 0.3089 | 0.1826 | 0.0947 | 0.0299 | 2.0053 | 0.1762 |
| **Blattabacteriaceae** | 0.0021 | 0.0020 | 0.0035 | 0.0029 | -0.6741 | 0.5411 | 0.0042 | 0.0024 | 0.0008 | 0.0004 | 2.3921 | 0.1331 |
| **Brachyspiraceae** | 0.0714 | 0.0081 | 0.0523 | 0.0078 | 2.9244 | 0.0431 | 0.0800 | 0.0275 | 0.0232 | 0.0038 | 3.5484 | 0.0673 |
| **Bradyrhizobiaceae** | 0.0212 | 0.0048 | 0.0287 | 0.0012 | -2.5923 | 0.1077 | 0.0171 | 0.0034 | 0.0100 | 0.0015 | 3.3188 | 0.0524 |
| **Brevibacteriaceae** | 0.0004 | 0.0003 | 0.0002 | 0.0001 | 1.0654 | 0.3889 | 0.0004 | 0.0002 | 0.0002 | 0.0002 | 1.8451 | 0.1390 |
| **Brucellaceae** | 0.0203 | 0.0116 | 0.0247 | 0.0018 | -0.6572 | 0.5757 | 0.0156 | 0.0029 | 0.0072 | 0.0029 | 3.5243 | 0.0244 |
| **Burkholderiaceae** | 0.0226 | 0.0035 | 0.0225 | 0.0041 | 0.0376 | 0.9719 | 0.0272 | 0.0004 | 0.0159 | 0.0041 | 4.7912 | 0.0393 |
| **Campylobacteraceae** | 0.0396 | 0.0011 | 0.0365 | 0.0017 | 2.6447 | 0.0657 | 0.0385 | 0.0043 | 0.0188 | 0.0022 | 7.0530 | 0.0062 |
| **Cardiobacteriaceae** | 0.0019 | 0.0007 | 0.0011 | 0.0007 | 1.4439 | 0.2230 | 0.0023 | 0.0004 | 0.0010 | 0.0003 | 5.0595 | 0.0098 |
| **Carnobacteriaceae** | 0.0242 | 0.0029 | 0.0114 | 0.0021 | 6.2641 | 0.0045 | 0.0255 | 0.0055 | 0.0196 | 0.0028 | 1.6729 | 0.1934 |
| **Catenulisporaceae** | 0.0014 | 0.0002 | 0.0008 | 0.0004 | 1.9938 | 0.1445 | 0.0014 | 0.0002 | 0.0021 | 0.0010 | -1.0962 | 0.3785 |
| **Caulobacteraceae** | 0.0096 | 0.0012 | 0.0072 | 0.0005 | 3.2338 | 0.0572 | 0.0091 | 0.0014 | 0.0045 | 0.0023 | 2.9350 | 0.0537 |
| **Cellulomonadaceae** | 0.0058 | 0.0068 | 0.0029 | 0.0009 | 0.7432 | 0.5327 | 0.0017 | 0.0008 | 0.0011 | 0.0010 | 0.8493 | 0.4448 |
| **Chlamydiaceae** | 0.0039 | 0.0030 | 0.0084 | 0.0117 | -0.6407 | 0.5805 | 0.0031 | 0.0022 | 0.0025 | 0.0015 | 0.3774 | 0.7274 |
| **Chlorobiaceae** | 0.0808 | 0.0081 | 0.0894 | 0.0096 | -1.1882 | 0.3022 | 0.0700 | 0.0066 | 0.0324 | 0.0048 | 7.9632 | 0.0019 |
| **Chloroflexaceae** | 0.0229 | 0.0032 | 0.0171 | 0.0047 | 1.7620 | 0.1626 | 0.0228 | 0.0032 | 0.0119 | 0.0039 | 3.7579 | 0.0212 |
| **Chromatiaceae** | 0.0162 | 0.0012 | 0.0068 | 0.0046 | 3.4354 | 0.0627 | 0.0112 | 0.0015 | 0.0051 | 0.0025 | 3.6119 | 0.0313 |
| **Chrysiogenaceae** | 0.0049 | 0.0028 | 0.0038 | 0.0025 | 0.5423 | 0.6169 | 0.0041 | 0.0012 | 0.0014 | 0.0005 | 3.6275 | 0.0398 |
| **Clostridiaceae** | 18.1951 | 2.8978 | 12.4679 | 2.7755 | 2.4721 | 0.0689 | 18.0047 | 7.3308 | 6.5751 | 0.7858 | 2.6851 | 0.1125 |
| **Clostridiales.Family.XI..Incertae.Sedis** | 0.1256 | 0.0228 | 0.1003 | 0.0323 | 1.1117 | 0.3350 | 0.1223 | 0.0394 | 0.0416 | 0.0065 | 3.4970 | 0.0676 |
| **Clostridiales.Family.XIV..Incertae.Sedis** | 0.0000 | 0.0000 | 0.0000 | 0.0000 | NA | NA | 0.0000 | 0.0000 | 0.0000 | 0.0000 | NA | NA |
| **Clostridiales.Family.XVII..Incertae.Sedis** | 0.0053 | 0.0016 | 0.0062 | 0.0033 | -0.4108 | 0.7098 | 0.0061 | 0.0009 | 0.0029 | 0.0007 | 4.8706 | 0.0099 |
| **Colwelliaceae** | 0.0026 | 0.0021 | 0.0064 | 0.0026 | -1.9785 | 0.1224 | 0.0036 | 0.0015 | 0.0011 | 0.0008 | 2.5839 | 0.0806 |
| **Comamonadaceae** | 0.0193 | 0.0053 | 0.0216 | 0.0034 | -0.6421 | 0.5618 | 0.0206 | 0.0036 | 0.0103 | 0.0002 | 4.9814 | 0.0375 |
| **Conexibacteraceae** | 0.0021 | 0.0010 | 0.0013 | 0.0007 | 1.0565 | 0.3570 | 0.0024 | 0.0004 | 0.0015 | 0.0005 | 2.2490 | 0.0973 |
| **Coriobacteriaceae** | 1.3998 | 1.0780 | 1.2742 | 0.9011 | 0.1548 | 0.8847 | 0.5729 | 0.1601 | 0.6120 | 0.6514 | -0.1011 | 0.9278 |
| **Corynebacteriaceae** | 0.0113 | 0.0004 | 0.0107 | 0.0018 | 0.5449 | 0.6364 | 0.0120 | 0.0007 | 0.0042 | 0.0006 | 14.4563 | 0.0002 |
| **Coxiellaceae** | 0.0021 | 0.0012 | 0.0012 | 0.0005 | 1.2173 | 0.3215 | 0.0021 | 0.0012 | 0.0010 | 0.0002 | 1.6376 | 0.2388 |
| **Cyclobacteriaceae** | 0.0090 | 0.0031 | 0.0122 | 0.0067 | -0.7363 | 0.5181 | 0.0107 | 0.0022 | 0.0043 | 0.0017 | 4.0220 | 0.0172 |
| **Cystobacteraceae** | 0.0020 | 0.0004 | 0.0033 | 0.0046 | -0.4873 | 0.6737 | 0.0014 | 0.0008 | 0.0007 | 0.0003 | 1.4505 | 0.2613 |
| **Cytophagaceae** | 0.1551 | 0.0271 | 0.1793 | 0.0516 | -0.7206 | 0.5228 | 0.1254 | 0.0027 | 0.0589 | 0.0080 | 13.5538 | 0.0023 |
| **Deferribacteraceae** | 0.0100 | 0.0011 | 0.0083 | 0.0026 | 0.9954 | 0.3990 | 0.0095 | 0.0027 | 0.0056 | 0.0016 | 2.1513 | 0.1120 |
| **Deinococcaceae** | 0.0099 | 0.0011 | 0.0123 | 0.0036 | -1.0696 | 0.3803 | 0.0108 | 0.0018 | 0.0066 | 0.0007 | 3.8254 | 0.0401 |
| **Dermabacteraceae** | 0.0025 | 0.0009 | 0.0019 | 0.0017 | 0.5389 | 0.6276 | 0.0013 | 0.0007 | 0.0008 | 0.0001 | 1.2436 | 0.3380 |

**Supplemental Table 3: *t-test* for Equality of Means of Bacterial Family Continued**

Dependent Variable: Diet

|  | **8 Weeks PD** | | | | | | | | **12 Weeks PD** | | | | | | | | |
| --- | --- | --- | --- | --- | --- | --- | --- | --- | --- | --- | --- | --- | --- | --- | --- | --- | --- |
|  | **Chow** | | **WD** | | **F** | | **P value** | | **Chow** | | **WD** | | **F** | | **P value** | | |
|  | **Mean** | **SD** | **Mean** | **SD** | |  | |  | **Mean** | **SD** | **Mean** | **SD** |  | | |  | |
| **Dermacoccaceae** | 0.0014 | 0.0010 | 0.0014 | 0.0009 | | 0.0763 | | 0.9429 | 0.0010 | 0.0005 | 0.0008 | 0.0005 | | 0.6613 | | | 0.5446 |
| **Desulfarculaceae** | 0.0031 | 0.0021 | 0.0029 | 0.0007 | | 0.1387 | | 0.9001 | 0.0021 | 0.0009 | 0.0009 | 0.0003 | | 2.2662 | | | 0.1328 |
| **Desulfobacteraceae** | 0.0143 | 0.0011 | 0.0074 | 0.0006 | | 9.7331 | | 0.0017 | 0.0134 | 0.0018 | 0.0050 | 0.0021 | | 5.3977 | | | 0.0060 |
| **Desulfobulbaceae** | 0.0084 | 0.0030 | 0.0149 | 0.0037 | | -2.3136 | | 0.0846 | 0.0103 | 0.0035 | 0.0043 | 0.0001 | | 3.0023 | | | 0.0951 |
| **Desulfohalobiaceae** | 0.0035 | 0.0013 | 0.0012 | 0.0001 | | 3.0914 | | 0.0887 | 0.0046 | 0.0013 | 0.0015 | 0.0002 | | 4.0564 | | | 0.0497 |
| **Desulfomicrobiaceae** | 0.0083 | 0.0010 | 0.0109 | 0.0046 | | -0.9612 | | 0.4300 | 0.0083 | 0.0029 | 0.0045 | 0.0022 | | 1.7988 | | | 0.1520 |
| **Desulfovibrionaceae** | 0.0511 | 0.0065 | 0.0490 | 0.0092 | | 0.3327 | | 0.7578 | 0.0530 | 0.0137 | 0.0245 | 0.0021 | | 3.5707 | | | 0.0657 |
| **Desulfurobacteriaceae** | 0.0045 | 0.0022 | 0.0039 | 0.0028 | | 0.2929 | | 0.7850 | 0.0033 | 0.0018 | 0.0014 | 0.0011 | | 1.6450 | | | 0.1905 |
| **Desulfuromonadaceae** | 0.0089 | 0.0010 | 0.0035 | 0.0015 | | 5.0824 | | 0.0099 | 0.0110 | 0.0043 | 0.0057 | 0.0028 | | 1.7801 | | | 0.1612 |
| **Dictyoglomaceae** | 0.0086 | 0.0007 | 0.0057 | 0.0032 | | 1.5548 | | 0.2491 | 0.0076 | 0.0011 | 0.0029 | 0.0002 | | 7.2448 | | | 0.0151 |
| **Dietziaceae** | 0.0001 | 0.0001 | 0.0002 | 0.0002 | | -1.0710 | | 0.3530 | 0.0004 | 0.0001 | 0.0001 | 0.0001 | | 2.4455 | | | 0.0714 |
| **Ectothiorhodospiraceae** | 0.0129 | 0.0019 | 0.0191 | 0.0084 | | -1.2311 | | 0.3333 | 0.0131 | 0.0029 | 0.0058 | 0.0011 | | 4.0224 | | | 0.0373 |
| **Elusimicrobiaceae** | 0.0148 | 0.0014 | 0.0039 | 0.0019 | | 8.0589 | | 0.0017 | 0.0096 | 0.0018 | 0.0026 | 0.0003 | | 6.7384 | | | 0.0177 |
| **Enterobacteriaceae** | 0.1461 | 0.0236 | 0.1485 | 0.0136 | | -0.1504 | | 0.8895 | 0.1170 | 0.0023 | 0.0972 | 0.0091 | | 3.6378 | | | 0.0567 |
| **Enterococcaceae** | 2.2192 | 0.5942 | 2.2898 | 1.0185 | | -0.1037 | | 0.9235 | 13.1972 | 16.8607 | 52.7090 | 4.8452 | | -3.9011 | | | 0.0466 |
| **Entomoplasmataceae** | 0.0057 | 0.0005 | 0.0044 | 0.0016 | | 1.3835 | | 0.2833 | 0.0048 | 0.0015 | 0.0015 | 0.0006 | | 3.5592 | | | 0.0481 |
| **Erysipelotrichaceae** | 2.6134 | 0.3463 | 2.0048 | 0.4775 | | 1.7873 | | 0.1553 | 1.9712 | 0.5337 | 0.8522 | 0.2085 | | 3.3828 | | | 0.0533 |
| **Erythrobacteraceae** | 0.0036 | 0.0015 | 0.0041 | 0.0008 | | -0.4865 | | 0.6585 | 0.0042 | 0.0005 | 0.0021 | 0.0005 | | 4.9780 | | | 0.0076 |
| **Eubacteriaceae** | 8.0328 | 1.2023 | 5.3334 | 1.2425 | | 2.7041 | | 0.0539 | 7.6673 | 3.3104 | 2.5886 | 0.5717 | | 2.6185 | | | 0.1132 |
| **Ferrimonadaceae** | 0.0043 | 0.0028 | 0.0059 | 0.0017 | | -0.8563 | | 0.4495 | 0.0030 | 0.0016 | 0.0026 | 0.0002 | | 0.4449 | | | 0.6987 |
| **Fibrobacteraceae** | 0.1209 | 0.0103 | 0.1097 | 0.0410 | | 0.4587 | | 0.6870 | 0.1070 | 0.0347 | 0.0426 | 0.0089 | | 3.1104 | | | 0.0768 |
| **Flammeovirgaceae** | 0.0269 | 0.0066 | 0.0303 | 0.0125 | | -0.4120 | | 0.7077 | 0.0186 | 0.0044 | 0.0088 | 0.0018 | | 3.5861 | | | 0.0459 |
| **Flavobacteriaceae** | 0.3710 | 0.0678 | 0.5362 | 0.2215 | | -1.2359 | | 0.3248 | 0.3110 | 0.0335 | 0.1525 | 0.0122 | | 7.6929 | | | 0.0082 |
| **Francisellaceae** | 0.0113 | 0.0033 | 0.0060 | 0.0046 | | 1.6139 | | 0.1893 | 0.0114 | 0.0071 | 0.0079 | 0.0043 | | 0.7238 | | | 0.5172 |
| **Frankiaceae** | 0.0085 | 0.0027 | 0.0058 | 0.0012 | | 1.5588 | | 0.2237 | 0.0048 | 0.0015 | 0.0028 | 0.0004 | | 2.3105 | | | 0.1296 |
| **Fusobacteriaceae** | 0.2279 | 0.0356 | 0.2037 | 0.0511 | | 0.6724 | | 0.5424 | 0.2284 | 0.0685 | 0.0979 | 0.0128 | | 3.2440 | | | 0.0761 |
| **Gallionellaceae** | 0.0038 | 0.0011 | 0.0067 | 0.0041 | | -1.1812 | | 0.3450 | 0.0018 | 0.0010 | 0.0017 | 0.0015 | | 0.1216 | | | 0.9100 |
| **Geobacteraceae** | 0.0424 | 0.0064 | 0.0391 | 0.0155 | | 0.3454 | | 0.7552 | 0.0373 | 0.0033 | 0.0178 | 0.0030 | | 7.6005 | | | 0.0017 |
| **Geodermatophilaceae** | 0.0012 | 0.0009 | 0.0010 | 0.0011 | | 0.2490 | | 0.8161 | 0.0012 | 0.0007 | 0.0006 | 0.0003 | | 1.4303 | | | 0.2513 |
| **Glycomycetaceae** | 0.0024 | 0.0010 | 0.0012 | 0.0004 | | 1.9788 | | 0.1533 | 0.0017 | 0.0014 | 0.0018 | 0.0012 | | -0.1596 | | | 0.8810 |
| **Gordoniaceae** | 0.0006 | 0.0003 | 0.0002 | 0.0003 | | 1.3891 | | 0.2381 | 0.0011 | 0.0008 | 0.0004 | 0.0003 | | 1.4257 | | | 0.2669 |
| **Hahellaceae** | 0.0035 | 0.0022 | 0.0030 | 0.0030 | | 0.2499 | | 0.8161 | 0.0032 | 0.0004 | 0.0014 | 0.0004 | | 5.3963 | | | 0.0058 |
| **Halanaerobiaceae** | 0.0304 | 0.0061 | 0.0255 | 0.0153 | | 0.5102 | | 0.6498 | 0.0306 | 0.0134 | 0.0109 | 0.0011 | | 2.5325 | | | 0.1252 |
| **Haliangiaceae** | 0.0036 | 0.0009 | 0.0040 | 0.0037 | | -0.1695 | | 0.8796 | 0.0042 | 0.0027 | 0.0014 | 0.0004 | | 1.8025 | | | 0.2073 |
| **Halobacteroidaceae** | 0.0082 | 0.0007 | 0.0095 | 0.0055 | | -0.3983 | | 0.7279 | 0.0095 | 0.0037 | 0.0041 | 0.0015 | | 2.2890 | | | 0.1174 |
| **Halomonadaceae** | 0.0030 | 0.0019 | 0.0051 | 0.0003 | | -1.8900 | | 0.1931 | 0.0028 | 0.0020 | 0.0012 | 0.0010 | | 1.2298 | | | 0.3086 |
| **Halothiobacillaceae** | 0.0099 | 0.0057 | 0.0140 | 0.0038 | | -1.0403 | | 0.3647 | 0.0043 | 0.0005 | 0.0016 | 0.0011 | | 3.7776 | | | 0.0359 |
| **Helicobacteraceae** | 0.0321 | 0.0041 | 0.0154 | 0.0042 | | 4.9214 | | 0.0079 | 0.0378 | 0.0088 | 0.0249 | 0.0100 | | 1.6776 | | | 0.1697 |
| **Heliobacteriaceae** | 0.0787 | 0.0070 | 0.0790 | 0.0421 | | -0.0130 | | 0.9908 | 0.0680 | 0.0237 | 0.0298 | 0.0092 | | 2.6022 | | | 0.0932 |
| **Herpetosiphonaceae** | 0.0092 | 0.0035 | 0.0027 | 0.0009 | | 3.1358 | | 0.0749 | 0.0048 | 0.0033 | 0.0017 | 0.0007 | | 1.5955 | | | 0.2409 |
| **Hydrogenophilaceae** | 0.0050 | 0.0037 | 0.0083 | 0.0062 | | -0.8050 | | 0.4753 | 0.0033 | 0.0020 | 0.0016 | 0.0009 | | 1.3404 | | | 0.2781 |
| **Hydrogenothermaceae** | 0.0056 | 0.0005 | 0.0078 | 0.0023 | | -1.6468 | | 0.2306 | 0.0084 | 0.0040 | 0.0045 | 0.0018 | | 1.5328 | | | 0.2294 |
| **Hyphomicrobiaceae** | 0.0023 | 0.0003 | 0.0009 | 0.0006 | | 3.6121 | | 0.0420 | 0.0028 | 0.0005 | 0.0008 | 0.0001 | | 7.2554 | | | 0.0163 |
| **Hyphomonadaceae** | 0.0038 | 0.0017 | 0.0112 | 0.0037 | | -3.1200 | | 0.0581 | 0.0056 | 0.0014 | 0.0031 | 0.0010 | | 2.5962 | | | 0.0659 |
| **Idiomarinaceae** | 0.0067 | 0.0036 | 0.0027 | 0.0014 | | 1.7961 | | 0.1860 | 0.0053 | 0.0035 | 0.0025 | 0.0016 | | 1.2875 | | | 0.2943 |
| **Intrasporangiaceae** | 0.0023 | 0.0001 | 0.0041 | 0.0028 | | -1.0721 | | 0.3958 | 0.0022 | 0.0004 | 0.0016 | 0.0005 | | 1.6778 | | | 0.1709 |
| **Jonesiaceae** | 0.0026 | 0.0003 | 0.0005 | 0.0005 | | 6.5638 | | 0.0035 | 0.0030 | 0.0013 | 0.0008 | 0.0002 | | 2.8597 | | | 0.0997 |
| **Kineosporiaceae** | 0.0013 | 0.0008 | 0.0019 | 0.0004 | | -0.9751 | | 0.4022 | 0.0029 | 0.0013 | 0.0010 | 0.0001 | | 2.5419 | | | 0.1237 |
| **Ktedonobacteraceae** | 0.0019 | 0.0006 | 0.0008 | 0.0006 | | 2.3441 | | 0.0791 | 0.0021 | 0.0005 | 0.0008 | 0.0000 | | 4.9209 | | | 0.0376 |
| **Lachnospiraceae** | 13.3740 | 2.1888 | 9.1069 | 1.9558 | | 2.5179 | | 0.0663 | 13.0858 | 6.0083 | 4.6901 | 0.8094 | | 2.3986 | | | 0.1342 |
| **Lactobacillaceae** | 2.6587 | 1.4437 | 3.3846 | 1.4719 | | -0.6098 | | 0.5750 | 0.7866 | 0.1907 | 2.1981 | 0.9875 | | -2.4309 | | | 0.1269 |
| **Legionellaceae** | 0.0031 | 0.0005 | 0.0027 | 0.0016 | | 0.3307 | | 0.7679 | 0.0033 | 0.0009 | 0.0010 | 0.0001 | | 4.2976 | | | 0.0469 |
| **Lentisphaeraceae** | 0.0037 | 0.0008 | 0.0073 | 0.0033 | | -1.7951 | | 0.2025 | 0.0020 | 0.0011 | 0.0016 | 0.0009 | | 0.4021 | | | 0.7090 |
| **Leptospiraceae** | 0.0185 | 0.0055 | 0.0106 | 0.0010 | | 2.4689 | | 0.1247 | 0.0133 | 0.0065 | 0.0045 | 0.0010 | | 2.3396 | | | 0.1386 |
| **Leuconostocaceae** | 0.0653 | 0.0244 | 0.1437 | 0.0482 | | -2.5154 | | 0.0876 | 0.1039 | 0.0303 | 0.1561 | 0.0038 | | -2.9633 | | | 0.0940 |
| **Listeriaceae** | 0.0763 | 0.0093 | 0.1267 | 0.0212 | | -3.7762 | | 0.0381 | 0.1171 | 0.0556 | 0.2132 | 0.0198 | | -2.8169 | | | 0.0826 |
| **Mariprofundaceae** | 0.0012 | 0.0006 | 0.0006 | 0.0004 | | 1.4228 | | 0.2308 | 0.0018 | 0.0009 | 0.0003 | 0.0001 | | 2.8045 | | | 0.1019 |
| **Methylacidiphilaceae** | 0.0055 | 0.0060 | 0.0020 | 0.0022 | | 0.9643 | | 0.4181 | 0.0053 | 0.0053 | 0.0023 | 0.0014 | | 0.9355 | | | 0.4374 |
| **Methylobacteriaceae** | 0.0063 | 0.0020 | 0.0060 | 0.0005 | | 0.3022 | | 0.7883 | 0.0053 | 0.0016 | 0.0044 | 0.0017 | | 0.6626 | | | 0.5440 |
| **Methylococcaceae** | 0.0064 | 0.0035 | 0.0058 | 0.0032 | | 0.2006 | | 0.8509 | 0.0054 | 0.0021 | 0.0031 | 0.0007 | | 1.7417 | | | 0.2010 |
| **Methylocystaceae** | 0.0013 | 0.0015 | 0.0005 | 0.0005 | | 0.8329 | | 0.4763 | 0.0009 | 0.0005 | 0.0011 | 0.0002 | | -0.6117 | | | 0.5890 |
| **Methylophilaceae** | 0.0076 | 0.0004 | 0.0076 | 0.0022 | | 0.0453 | | 0.9678 | 0.0068 | 0.0003 | 0.0037 | 0.0015 | | 3.6255 | | | 0.0585 |
| **Microbacteriaceae** | 0.0015 | 0.0003 | 0.0023 | 0.0019 | | -0.7255 | | 0.5396 | 0.0014 | 0.0007 | 0.0008 | 0.0001 | | 1.2700 | | | 0.3276 |
| **Microchaetaceae** | 0.0001 | 0.0001 | 0.0000 | 0.0000 | | 1.0000 | | 0.4226 | 0.0000 | 0.0000 | 0.0000 | 0.0000 | | NA | | | NA |
| **Micrococcaceae** | 0.0149 | 0.0059 | 0.0098 | 0.0042 | | 1.2274 | | 0.2937 | 0.0128 | 0.0045 | 0.0182 | 0.0017 | | -1.9384 | | | 0.1622 |
| **Micromonosporaceae** | 0.0122 | 0.0065 | 0.0046 | 0.0017 | | 1.9672 | | 0.1721 | 0.0083 | 0.0051 | 0.0033 | 0.0001 | | 1.7069 | | | 0.2298 |
| **Moraxellaceae** | 0.0225 | 0.0101 | 0.0190 | 0.0064 | | 0.5005 | | 0.6474 | 0.0237 | 0.0190 | 0.0503 | 0.0118 | | -2.0539 | | | 0.1227 |
| **Moritellaceae** | 0.0007 | 0.0005 | 0.0005 | 0.0004 | | 0.7074 | | 0.5188 | 0.0009 | 0.0002 | 0.0004 | 0.0001 | | 4.2597 | | | 0.0316 |
| **Mycobacteriaceae** | 0.0099 | 0.0034 | 0.0088 | 0.0035 | | 0.3824 | | 0.7216 | 0.0091 | 0.0011 | 0.0049 | 0.0011 | | 4.6897 | | | 0.0094 |
| **Mycoplasmataceae** | 0.0197 | 0.0051 | 0.0089 | 0.0027 | | 3.2491 | | 0.0466 | 0.0149 | 0.0059 | 0.0056 | 0.0027 | | 2.4783 | | | 0.0951 |
| **Myxococcaceae** | 0.0165 | 0.0031 | 0.0181 | 0.0059 | | -0.4033 | | 0.7135 | 0.0186 | 0.0032 | 0.0096 | 0.0021 | | 4.0928 | | | 0.0205 |

**Supplemental Table 3: *t-test* for Equality of Means of Bacterial Family Continued**

Dependent Variable: Diet

|  | **8 Weeks PD** | | | | | | **12 Weeks PD** | | | | | | |  |
| --- | --- | --- | --- | --- | --- | --- | --- | --- | --- | --- | --- | --- | --- | --- |
|  | **Chow** | | **WD** | | **F** | **P value** | | **Chow** | | **WD** | | **F** | **P value** | |
|  | **Mean** | **SD** | **Mean** | **SD** |  |  | | **Mean** | **SD** | **Mean** | **SD** |  |  | |
| **NAkamurellaceae** | 0.0017 | 0.0015 | 0.0013 | 0.0005 | 0.4033 | 0.7204 | | 0.0017 | 0.0002 | 0.0007 | 0.0004 | 3.8089 | 0.0345 | |
| **NAnnocystaceae** | 0.0004 | 0.0005 | 0.0004 | 0.0003 | -0.0315 | 0.9769 | | 0.0008 | 0.0002 | 0.0002 | 0.0001 | 4.4007 | 0.0140 | |
| **NAtranaerobiaceae** | 0.0117 | 0.0010 | 0.0067 | 0.0035 | 2.3896 | 0.1226 | | 0.0093 | 0.0028 | 0.0031 | 0.0014 | 3.4922 | 0.0417 | |
| **NAutiliaceae** | 0.0047 | 0.0018 | 0.0059 | 0.0034 | -0.5410 | 0.6263 | | 0.0040 | 0.0024 | 0.0010 | 0.0006 | 2.0328 | 0.1631 | |
| **Neisseriaceae** | 0.0440 | 0.0157 | 0.0636 | 0.0302 | -0.9963 | 0.3923 | | 0.0378 | 0.0055 | 0.0178 | 0.0039 | 5.1630 | 0.0086 | |
| **Nitrosomonadaceae** | 0.0121 | 0.0025 | 0.0126 | 0.0059 | -0.1464 | 0.8940 | | 0.0075 | 0.0011 | 0.0060 | 0.0006 | 2.0028 | 0.1351 | |
| **Nitrospiraceae** | 0.0070 | 0.0007 | 0.0033 | 0.0018 | 3.2807 | 0.0550 | | 0.0059 | 0.0040 | 0.0027 | 0.0017 | 1.2674 | 0.3025 | |
| **Nocardiaceae** | 0.0085 | 0.0021 | 0.0108 | 0.0043 | -0.8559 | 0.4567 | | 0.0042 | 0.0003 | 0.0026 | 0.0006 | 4.5116 | 0.0209 | |
| **Nocardioidaceae** | 0.0079 | 0.0043 | 0.0039 | 0.0014 | 1.5244 | 0.2457 | | 0.0063 | 0.0006 | 0.0025 | 0.0005 | 8.9441 | 0.0011 | |
| **Nocardiopsaceae** | 0.0042 | 0.0008 | 0.0029 | 0.0009 | 1.8893 | 0.1321 | | 0.0043 | 0.0012 | 0.0017 | 0.0003 | 3.7323 | 0.0527 | |
| **Nostocaceae** | 0.0109 | 0.0021 | 0.0094 | 0.0030 | 0.6976 | 0.5282 | | 0.0115 | 0.0076 | 0.0048 | 0.0009 | 1.5074 | 0.2672 | |
| **Oceanospirillaceae** | 0.0065 | 0.0018 | 0.0063 | 0.0018 | 0.1024 | 0.9234 | | 0.0049 | 0.0008 | 0.0030 | 0.0009 | 2.6483 | 0.0573 | |
| **Opitutaceae** | 0.0131 | 0.0087 | 0.0194 | 0.0086 | -0.9003 | 0.4189 | | 0.0128 | 0.0035 | 0.0051 | 0.0023 | 3.1575 | 0.0411 | |
| **Oscillochloridaceae** | 0.0010 | 0.0005 | 0.0004 | 0.0002 | 1.8077 | 0.1791 | | 0.0010 | 0.0007 | 0.0004 | 0.0002 | 1.5251 | 0.2535 | |
| **Oxalobacteraceae** | 0.0164 | 0.0020 | 0.0174 | 0.0074 | -0.2158 | 0.8469 | | 0.0118 | 0.0015 | 0.0061 | 0.0022 | 3.6189 | 0.0279 | |
| **Paenibacillaceae** | 0.1323 | 0.0258 | 0.0924 | 0.0290 | 1.7763 | 0.1513 | | 0.2537 | 0.1894 | 0.6075 | 0.0586 | -3.0912 | 0.0727 | |
| **Parachlamydiaceae** | 0.0050 | 0.0036 | 0.0031 | 0.0037 | 0.6336 | 0.5608 | | 0.0012 | 0.0003 | 0.0005 | 0.0001 | 3.7292 | 0.0397 | |
| **Parvularculaceae** | 0.0009 | 0.0004 | 0.0025 | 0.0015 | -1.8410 | 0.1886 | | 0.0018 | 0.0003 | 0.0006 | 0.0003 | 5.1350 | 0.0073 | |
| **Pasteurellaceae** | 0.0586 | 0.0095 | 0.0467 | 0.0074 | 1.7140 | 0.1659 | | 0.0608 | 0.0101 | 0.0378 | 0.0021 | 3.8538 | 0.0539 | |
| **Pasteuriaceae** | 0.0000 | 0.0000 | 0.0000 | 0.0001 | -1.0000 | 0.4226 | | 0.0000 | 0.0001 | 0.0000 | 0.0000 | 1.0000 | 0.4226 | |
| **Pelobacteraceae** | 0.0211 | 0.0035 | 0.0181 | 0.0115 | 0.4397 | 0.6970 | | 0.0217 | 0.0055 | 0.0093 | 0.0029 | 3.4736 | 0.0395 | |
| **Peptococcaceae** | 0.2190 | 0.0344 | 0.1715 | 0.0741 | 1.0082 | 0.3917 | | 0.2136 | 0.0903 | 0.0907 | 0.0056 | 2.3520 | 0.1420 | |
| **Peptostreptococcaceae** | 0.0678 | 0.0126 | 0.0490 | 0.0199 | 1.3808 | 0.2517 | | 0.0643 | 0.0309 | 0.0220 | 0.0090 | 2.2768 | 0.1323 | |
| **Phyllobacteriaceae** | 0.0102 | 0.0012 | 0.0132 | 0.0026 | -1.8501 | 0.1689 | | 0.0083 | 0.0017 | 0.0056 | 0.0005 | 2.6558 | 0.0983 | |
| **Piscirickettsiaceae** | 0.0053 | 0.0011 | 0.0037 | 0.0029 | 0.9146 | 0.4370 | | 0.0025 | 0.0016 | 0.0016 | 0.0007 | 0.8779 | 0.4495 | |
| **Planctomycetaceae** | 0.0100 | 0.0022 | 0.0104 | 0.0027 | -0.1691 | 0.8742 | | 0.0115 | 0.0020 | 0.0070 | 0.0004 | 3.8256 | 0.0538 | |
| **Planococcaceae** | 0.0040 | 0.0027 | 0.0013 | 0.0002 | 1.7110 | 0.2272 | | 0.0031 | 0.0008 | 0.0032 | 0.0011 | -0.1293 | 0.9039 | |
| **Polyangiaceae** | 0.0000 | 0.0000 | 0.0000 | 0.0000 | NA | NA | | 0.0000 | 0.0000 | 0.0000 | 0.0000 | -1.0000 | 0.4226 | |
| **Porphyromonadaceae** | 4.0061 | 0.3710 | 5.9083 | 2.2003 | -1.4765 | 0.2714 | | 2.7317 | 0.3540 | 1.4530 | 0.1983 | 5.4588 | 0.0107 | |
| **Prevotellaceae** | 3.0429 | 0.1847 | 4.4246 | 1.6704 | -1.4241 | 0.2877 | | 2.2578 | 0.2589 | 1.1928 | 0.0866 | 6.7558 | 0.0123 | |
| **Prochlorococcaceae** | 0.0058 | 0.0019 | 0.0047 | 0.0017 | 0.6921 | 0.5275 | | 0.0054 | 0.0004 | 0.0028 | 0.0006 | 6.0220 | 0.0050 | |
| **Promicromonosporaceae** | 0.0020 | 0.0014 | 0.0031 | 0.0013 | -1.0112 | 0.3694 | | 0.0044 | 0.0032 | 0.0014 | 0.0005 | 1.6139 | 0.2416 | |
| **Propionibacteriaceae** | 0.0088 | 0.0004 | 0.0112 | 0.0073 | -0.5550 | 0.6343 | | 0.0123 | 0.0094 | 0.0049 | 0.0019 | 1.3344 | 0.3051 | |
| **Pseudoalteromonadaceae** | 0.0057 | 0.0028 | 0.0056 | 0.0005 | 0.0653 | 0.9536 | | 0.0057 | 0.0020 | 0.0029 | 0.0015 | 1.9029 | 0.1368 | |
| **Pseudomonadaceae** | 0.0703 | 0.0604 | 0.0529 | 0.0424 | 0.4103 | 0.7049 | | 0.0672 | 0.0610 | 0.1647 | 0.0258 | -2.5459 | 0.0938 | |
| **Pseudonocardiaceae** | 0.0042 | 0.0004 | 0.0034 | 0.0023 | 0.5650 | 0.6256 | | 0.0045 | 0.0007 | 0.0021 | 0.0013 | 2.8420 | 0.0610 | |
| **Psychromonadaceae** | 0.0036 | 0.0021 | 0.0043 | 0.0021 | -0.3878 | 0.7179 | | 0.0063 | 0.0007 | 0.0029 | 0.0016 | 3.3049 | 0.0503 | |
| **Puniceicoccaceae** | 0.0038 | 0.0014 | 0.0058 | 0.0028 | -1.0738 | 0.3625 | | 0.0053 | 0.0023 | 0.0039 | 0.0012 | 0.9054 | 0.4302 | |
| **Rhizobiaceae** | 0.0138 | 0.0005 | 0.0148 | 0.0052 | -0.3142 | 0.7826 | | 0.0182 | 0.0065 | 0.0086 | 0.0013 | 2.4792 | 0.1217 | |
| **Rhodobacteraceae** | 0.0308 | 0.0033 | 0.0323 | 0.0151 | -0.1681 | 0.8808 | | 0.0316 | 0.0043 | 0.0116 | 0.0019 | 7.3398 | 0.0073 | |
| **Rhodobiaceae** | 0.0000 | 0.0000 | 0.0000 | 0.0000 | NA | NA | | 0.0000 | 0.0000 | 0.0000 | 0.0000 | NA | NA | |
| **Rhodocyclaceae** | 0.0099 | 0.0014 | 0.0116 | 0.0077 | -0.3817 | 0.7374 | | 0.0048 | 0.0008 | 0.0034 | 0.0006 | 2.4712 | 0.0746 | |
| **Rhodospirillaceae** | 0.0055 | 0.0038 | 0.0101 | 0.0051 | -1.2540 | 0.2835 | | 0.0097 | 0.0032 | 0.0044 | 0.0022 | 2.3866 | 0.0842 | |
| **Rhodothermaceae** | 0.0112 | 0.0008 | 0.0116 | 0.0016 | -0.3868 | 0.7245 | | 0.0119 | 0.0045 | 0.0052 | 0.0018 | 2.4261 | 0.1057 | |
| **Rickettsiaceae** | 0.0047 | 0.0010 | 0.0033 | 0.0029 | 0.7959 | 0.4955 | | 0.0044 | 0.0011 | 0.0023 | 0.0007 | 2.8100 | 0.0631 | |
| **Rikenellaceae** | 3.4056 | 4.5383 | 1.4354 | 0.3978 | 0.7491 | 0.5309 | | 2.9607 | 2.3937 | 0.5166 | 0.2174 | 1.7613 | 0.2182 | |
| **Rubrobacteraceae** | 0.0058 | 0.0016 | 0.0043 | 0.0017 | 1.1137 | 0.3278 | | 0.0041 | 0.0009 | 0.0016 | 0.0003 | 4.4180 | 0.0295 | |
| **Ruminococcaceae** | 8.5891 | 1.5831 | 6.0831 | 1.7762 | 1.8243 | 0.1431 | | 8.0036 | 3.1847 | 3.8093 | 1.4626 | 2.0730 | 0.1361 | |
| **Sanguibacteraceae** | 0.0018 | 0.0010 | 0.0010 | 0.0010 | 0.9590 | 0.3921 | | 0.0020 | 0.0011 | 0.0008 | 0.0003 | 1.8729 | 0.1844 | |
| **Segniliparaceae** | 0.0011 | 0.0005 | 0.0003 | 0.0003 | 2.4065 | 0.0931 | | 0.0008 | 0.0004 | 0.0002 | 0.0002 | 2.4969 | 0.0976 | |
| **Shewanellaceae** | 0.0313 | 0.0079 | 0.0267 | 0.0123 | 0.5491 | 0.6170 | | 0.0288 | 0.0033 | 0.0121 | 0.0022 | 7.3079 | 0.0032 | |
| **Simkaniaceae** | 0.0000 | 0.0000 | 0.0000 | 0.0000 | NA | NA | | 0.0000 | 0.0000 | 0.0000 | 0.0000 | 1.0000 | 0.4226 | |
| **Solibacteraceae** | 0.0094 | 0.0016 | 0.0087 | 0.0023 | 0.4206 | 0.6981 | | 0.0056 | 0.0004 | 0.0050 | 0.0007 | 1.2937 | 0.2809 | |
| **Sphaerobacteraceae** | 0.0035 | 0.0017 | 0.0019 | 0.0013 | 1.3447 | 0.2531 | | 0.0036 | 0.0006 | 0.0019 | 0.0012 | 2.2844 | 0.1096 | |
| **Sphingobacteriaceae** | 0.1576 | 0.0128 | 0.1696 | 0.0590 | -0.3444 | 0.7608 | | 0.1274 | 0.0172 | 0.0525 | 0.0024 | 7.4514 | 0.0158 | |
| **Sphingomonadaceae** | 0.0122 | 0.0037 | 0.0105 | 0.0013 | 0.7345 | 0.5261 | | 0.0128 | 0.0032 | 0.0045 | 0.0018 | 3.9272 | 0.0278 | |
| **Spirochaetaceae** | 0.0918 | 0.0201 | 0.0758 | 0.0217 | 0.9372 | 0.4020 | | 0.1009 | 0.0316 | 0.0387 | 0.0090 | 3.2826 | 0.0667 | |
| **Spiroplasmataceae** | 0.0022 | 0.0014 | 0.0012 | 0.0006 | 1.1425 | 0.3451 | | 0.0005 | 0.0004 | 0.0003 | 0.0001 | 0.8985 | 0.4586 | |
| **Sporolactobacillaceae** | 0.0000 | 0.0000 | 0.0000 | 0.0000 | NA | NA | | 0.0000 | 0.0000 | 0.0000 | 0.0000 | NA | NA | |
| **Staphylococcaceae** | 0.0554 | 0.0111 | 0.0314 | 0.0034 | 3.5728 | 0.0547 | | 0.0621 | 0.0219 | 0.0861 | 0.0071 | -1.8109 | 0.1896 | |
| **Streptococcaceae** | 1.3117 | 0.6465 | 4.5001 | 0.9261 | -4.8894 | 0.0107 | | 1.7859 | 0.5067 | 3.2933 | 0.2085 | -4.7651 | 0.0230 | |
| **Streptomycetaceae** | 0.0083 | 0.0039 | 0.0100 | 0.0067 | -0.3913 | 0.7203 | | 0.0093 | 0.0017 | 0.0054 | 0.0025 | 2.2849 | 0.0934 | |
| **Streptosporangiaceae** | 0.0019 | 0.0002 | 0.0013 | 0.0016 | 0.5429 | 0.6405 | | 0.0019 | 0.0005 | 0.0006 | 0.0003 | 3.6864 | 0.0348 | |
| **Succinivibrionaceae** | 0.0298 | 0.0068 | 0.0241 | 0.0076 | 0.9720 | 0.3868 | | 0.0297 | 0.0116 | 0.0120 | 0.0020 | 2.5924 | 0.1151 | |
| **Synergistaceae** | 0.0733 | 0.0139 | 0.0582 | 0.0154 | 1.2650 | 0.2752 | | 0.0607 | 0.0220 | 0.0316 | 0.0021 | 2.2811 | 0.1478 | |
| **Syntrophaceae** | 0.0078 | 0.0053 | 0.0107 | 0.0032 | -0.8129 | 0.4706 | | 0.0081 | 0.0013 | 0.0039 | 0.0009 | 4.6730 | 0.0118 | |
| **Syntrophobacteraceae** | 0.0135 | 0.0040 | 0.0098 | 0.0085 | 0.6800 | 0.5476 | | 0.0102 | 0.0033 | 0.0052 | 0.0004 | 2.5710 | 0.1198 | |
| **Syntrophomonadaceae** | 0.0526 | 0.0155 | 0.0444 | 0.0184 | 0.5908 | 0.5873 | | 0.0524 | 0.0239 | 0.0152 | 0.0015 | 2.6814 | 0.1145 | |
| **Thermaceae** | 0.0122 | 0.0042 | 0.0057 | 0.0020 | 2.4234 | 0.0990 | | 0.0089 | 0.0008 | 0.0037 | 0.0006 | 9.1205 | 0.0012 | |
| **Thermoactinomycetaceae** | 0.0000 | 0.0000 | 0.0000 | 0.0000 | NA | NA | | 0.0000 | 0.0000 | 0.0000 | 0.0000 | -0.3502 | 0.7477 | |
| **Thermoanaerobacteraceae** | 0.2140 | 0.0253 | 0.1589 | 0.0735 | 1.2280 | 0.3236 | | 0.1984 | 0.0717 | 0.0703 | 0.0028 | 3.0914 | 0.0903 | |

**Supplemental Table 3: *t-test* for Equality of Means of Bacterial Family Continued**

Dependent Variable: Diet

|  | **8 Weeks PD** | | | | | | **12 Weeks PD** | | | | | | | |  |
| --- | --- | --- | --- | --- | --- | --- | --- | --- | --- | --- | --- | --- | --- | --- | --- |
|  | **Chow** | | **WD** | | **F** | **P value** | | **Chow** | | **WD** | | | **F** | **P value** | |
|  | **Mean** | **SD** | **Mean** | **SD** |  |  | | **Mean** | **SD** | **Mean** | **SD** | |  |  | |
| **Thermoanaerobacterales.Family.III.. Incertae.Sedis** | 0.1782 | 0.0227 | 0.1191 | 0.0607 | 1.5799 | 0.2279 | | 0.1426 | 0.0321 | 0.0493 | 0.0030 | 5.0126 | | 0.0362 |  |
| **Thermodesulfobiaceae** | 0.0016 | 0.0007 | 0.0023 | 0.0014 | -0.7431 | 0.5126 | | 0.0019 | 0.0006 | 0.0006 | 0.0000 | 3.7436 | | 0.0639 |  |
| **Thermomicrobiaceae** | 0.0024 | 0.0005 | 0.0018 | 0.0004 | 1.5779 | 0.1927 | | 0.0018 | 0.0008 | 0.0010 | 0.0001 | 1.5846 | | 0.2525 |  |
| **Thermomonosporaceae** | 0.0013 | 0.0006 | 0.0015 | 0.0006 | -0.2509 | 0.8142 | | 0.0012 | 0.0002 | 0.0005 | 0.0003 | 3.0294 | | 0.0471 |  |
| **Thermotogaceae** | 0.0316 | 0.0008 | 0.0248 | 0.0067 | 1.7554 | 0.2175 | | 0.0314 | 0.0046 | 0.0132 | 0.0008 | 6.7209 | | 0.0181 |  |
| **Thiotrichaceae** | 0.0005 | 0.0005 | 0.0005 | 0.0005 | 0.0028 | 0.9979 | | 0.0006 | 0.0001 | 0.0005 | 0.0004 | 0.2025 | | 0.8572 |  |
| **Trueperaceae** | 0.0022 | 0.0011 | 0.0022 | 0.0018 | -0.0179 | 0.9867 | | 0.0019 | 0.0004 | 0.0014 | 0.0007 | 1.0754 | | 0.3540 |  |
| **Tsukamurellaceae** | 0.0057 | 0.0038 | 0.0068 | 0.0028 | -0.4180 | 0.6991 | | 0.0023 | 0.0018 | 0.0009 | 0.0003 | 1.3128 | | 0.3151 |  |
| **Veillonellaceae** | 0.2734 | 0.0269 | 0.2155 | 0.0649 | 1.4266 | 0.2595 | | 0.2878 | 0.0894 | 0.1152 | 0.0021 | 3.3432 | | 0.0789 |  |
| **Verrucomicrobia.subdivision.3** | 0.0042 | 0.0046 | 0.0095 | 0.0121 | -0.7235 | 0.5298 | | 0.0060 | 0.0031 | 0.0035 | 0.0014 | 1.2595 | | 0.3013 |  |
| **Verrucomicrobiaceae** | 4.5474 | 7.7277 | 11.6350 | 19.8391 | -0.5766 | 0.6104 | | 3.4750 | 4.8288 | 4.6965 | 4.9070 | -0.3073 | | 0.7739 |  |
| **Vibrionaceae** | 0.0267 | 0.0023 | 0.0285 | 0.0045 | -0.6225 | 0.5781 | | 0.0306 | 0.0013 | 0.0142 | 0.0010 | 17.5828 | | 0.0001 |  |
| **Victivallaceae** | 0.0278 | 0.0059 | 0.0315 | 0.0033 | -0.9642 | 0.4026 | | 0.0190 | 0.0056 | 0.0106 | 0.0027 | 2.3220 | | 0.1070 |  |
| **Waddliaceae** | 0.0010 | 0.0007 | 0.0014 | 0.0005 | -0.7202 | 0.5154 | | 0.0020 | 0.0004 | 0.0015 | 0.0017 | 0.4958 | | 0.6655 |  |
| **Williamsiaceae** | 0.0000 | 0.0000 | 0.0000 | 0.0000 | NA | NA | | 0.0000 | 0.0000 | 0.0000 | 0.0000 | 1.0000 | | 0.4226 |  |
| **Xanthobacteraceae** | 0.0040 | 0.0011 | 0.0021 | 0.0004 | 2.8924 | 0.0728 | | 0.0041 | 0.0003 | 0.0016 | 0.0003 | 11.0937 | | 0.0004 |  |
| **Xanthomonadaceae** | 0.0186 | 0.0017 | 0.0159 | 0.0085 | 0.5403 | 0.6398 | | 0.0166 | 0.0066 | 0.0078 | 0.0025 | 2.1651 | | 0.1346 |  |
