# Supplementary material for: Integrative Longitudinal Analysis of Metabolic Phenotype and Microbiota Changes During the Development of Obesity
Source: Front Cell Infect Microbiol. 2021 Aug 3;11:671926. doi: 10.3389/fcimb.2021.671926 (PMC8370388; doi:10.3389/fcimb.2021.671926)
Supplement: Supplementary file 5 [file Table_4.docx]

**Supplemental Table 4: *t-test* for Equality of Means of Viral Families within the Order Caudovirales**

Dependent Variable: Diet

|  | **Day 0** | | | | | | | | | | | | **2 Days PD** | | | | | | | | **2 Weeks PD** | | | | | |
| --- | --- | --- | --- | --- | --- | --- | --- | --- | --- | --- | --- | --- | --- | --- | --- | --- | --- | --- | --- | --- | --- | --- | --- | --- | --- | --- |
|  | **Chow** | | | | **WD** | | | | | **F** | | **P value** | **Chow** | | | **WD** | | **F** | | **P value** | **Chow** | | **WD** | | **F** | **P value** |
|  | **Mean** | | **SD** | | **Mean** | | | **SD** | |  | |  | **Mean** | | **SD** | **Mean** | **SD** |  | |  | **Mean** | **SD** | **Mean** | **SD** |  |  |
| **Myoviridae** | 30.6549 | | 4.2343 | | 27.0929 | | | 6.7158 | | 0.7771 | | 0.4880 | 27.9932 | | 7.0451 | 17.0704 | 6.7424 | 1.9401 | | 0.1245 | 29.5282 | 2.9154 | 7.8997 | 1.7988 | 10.9355 | 0.0010 |
| **Podoviridae** | 9.1439 | | 4.2490 | | 8.0928 | | | 3.7275 | | 0.3221 | | 0.7638 | 4.0031 | | 2.3127 | 2.7993 | 1.6089 | 0.7401 | | 0.5050 | 11.6960 | 5.2014 | 4.7771 | 1.4925 | 2.2146 | 0.1391 |
| **Siphoviridae** | 52.4628 | | 2.6628 | | 59.2296 | | | 7.4079 | | -1.4889 | | 0.2501 | 61.6397 | | 4.4497 | 75.8496 | 9.3111 | -2.3850 | | 0.1012 | 53.8677 | 2.1008 | 86.3300 | 2.2691 | -18.1829 | 0.0001 |
| **unclassified** | 7.7385 | | 5.0157 | | 5.5847 | | | 3.0073 | | 0.6379 | | 0.5654 | 6.3640 | | 3.2631 | 4.2807 | 2.3272 | 0.9003 | | 0.4239 | 4.9081 | 1.4313 | 0.9932 | 0.2034 | 4.6902 | 0.0394 |
|  | **8 Weeks PD** | | | | | | | | | | | | **12 Weeks PD** | | | | | | | |  |  |  |  |  |  |
|  | **Chow** | | | **WD** | | | | | **F** | | **P value** | | **Chow** | | | **WD** | | | **F** | **P value** |  |  |  |  |  |  |
|  | **Mean** | **SD** | | **Mean** | | **SD** | | |  | |  | | **Mean** | **SD** | | **Mean** | **SD** | |  |  |  |  |  |  |  |  |
| **Myoviridae** | 29.5172 | 7.7274 | | 14.2586 | | | 4.9482 | | 2.8802 | | 0.0546 | | 18.5103 | 11.9874 | | 2.2970 | 0.5485 | | 2.3402 | 0.1436 |  |  |  |  |  |  |
| **Podoviridae** | 4.8513 | 5.4856 | | 13.0946 | | | 3.0326 | | -2.2779 | | 0.1038 | | 4.6126 | 2.2992 | | 1.6727 | 0.7634 | | 2.1020 | 0.1471 |  |  |  |  |  |  |
| **Siphoviridae** | 62.3797 | 9.3653 | | 68.3475 | | | 3.5388 | | -1.0325 | | 0.3894 | | 68.8058 | 20.6140 | | 95.7761 | 1.3667 | | -2.2612 | 0.1511 |  |  |  |  |  |  |
| **unclassified** | 3.2518 | 0.8794 | | 4.2993 | | | 2.4128 | | -0.7065 | | 0.5395 | | 8.0712 | 6.4626 | | 0.2543 | 0.1172 | | 2.0947 | 0.1711 |  |  |  |  |  |  |
