# Supplementary material for: Integrative Longitudinal Analysis of Metabolic Phenotype and Microbiota Changes During the Development of Obesity
Source: Front Cell Infect Microbiol. 2021 Aug 3;11:671926. doi: 10.3389/fcimb.2021.671926 (PMC8370388; doi:10.3389/fcimb.2021.671926)
Supplement: Supplementary file 6 [file Table_5.docx]

**Supplemental Table 5: *t-test* for Equality of Means of Viral Genera within the Order Caudovirales**

Dependent Variable: Diet

|  |  |  |  |  | **Day 0** | |  |  |  |  | **2 Days PD** | |  |  |  |  | **2 Weeks PD** | |  |  |
| --- | --- | --- | --- | --- | --- | --- | --- | --- | --- | --- | --- | --- | --- | --- | --- | --- | --- | --- | --- | --- |
|  |  |  | **Chow** | | **WD** | | **F** | **P value** | **Chow** | | **WD** | | **F** | **P value** | **Chow** | | **WD** | | **F** | **P value** |
| **Family** | **Subfamily** | **Genera** | **Mean** | **SD** | **Mean** | **SD** |  |  | **Mean** | **SD** | **Mean** | **SD** |  |  | **Mean** | **SD** | **Mean** | **SD** |  |  |
| **Myoviridae** | **Peduovirinae** | **Hp1-like viruses** | 0.0000 | 0.0000 | 0.0000 | 0.0000 | NA | NA | 0.0647 | 0.1121 | 0.0604 | 0.1046 | 0.0490 | 0.9633 | 0.0000 | 0.0000 | 0.0000 | 0.0000 | NA | NA |
| **Myoviridae** | **Peduovirinae** | **P2-like viruses** | 0.2226 | 0.0399 | 0.9021 | 0.6690 | -1.7561 | 0.2203 | 0.9259 | 0.4020 | 0.4820 | 0.4175 | 1.3265 | 0.2554 | 0.3537 | 0.6126 | 0.3446 | 0.2976 | 0.0230 | 0.9832 |
| **Myoviridae** | **Spounavirinae** | **K-like viruses** | 0.0000 | 0.0000 | 0.0000 | 0.0000 | NA | NA | 0.0000 | 0.0000 | 0.0000 | 0.0000 | NA | NA | 0.0000 | 0.0000 | 0.0000 | 0.0000 | NA | NA |
| **Myoviridae** | **Spounavirinae** | **P100-like viruses** | 0.7513 | 0.9305 | 0.0000 | 0.0000 | 1.3986 | 0.2968 | 0.3361 | 0.3132 | 0.1566 | 0.1459 | 0.8997 | 0.4382 | 0.3092 | 0.3333 | 1.8295 | 1.6681 | -1.5481 | 0.2527 |
| **Myoviridae** | **Spounavirinae** | **SPO1-like viruses** | 3.9870 | 3.3957 | 2.9771 | 2.2665 | 0.4285 | 0.6934 | 1.5963 | 0.8942 | 1.0352 | 1.2205 | 0.6424 | 0.5586 | 2.9306 | 2.4982 | 0.2647 | 0.3351 | 1.8319 | 0.2040 |
| **Myoviridae** | **Spounavirinae** | **Twort-like viruses** | 0.4446 | 0.6214 | 0.6383 | 0.4155 | -0.4488 | 0.6800 | 0.5617 | 0.2757 | 0.2341 | 0.2795 | 1.4457 | 0.2218 | 1.1106 | 0.1939 | 0.0426 | 0.0389 | 9.3545 | 0.0087 |
| **Myoviridae** | **Spounavirinae** | **Unclassified** | 0.1629 | 0.1417 | 0.0536 | 0.0928 | 1.1176 | 0.3355 | 0.1377 | 0.2386 | 0.0481 | 0.0833 | 0.6144 | 0.5906 | 0.3259 | 0.3312 | 0.0000 | 0.0000 | 1.7041 | 0.2305 |
| **Myoviridae** | **Tevenvirinae** | **JS98-like viruses** | 0.0000 | 0.0000 | 0.0000 | 0.0000 | NA | NA | 0.0000 | 0.0000 | 0.0000 | 0.0000 | NA | NA | 0.0000 | 0.0000 | 0.0000 | 0.0000 | NA | NA |
| **Myoviridae** | **Tevenvirinae** | **RB49-like** | 0.0597 | 0.1035 | 0.0000 | 0.0000 | 1.0000 | 0.4226 | 0.0000 | 0.0000 | 0.0000 | 0.0000 | NA | NA | 0.0000 | 0.0000 | 0.0000 | 0.0000 | NA | NA |
| **Myoviridae** | **Tevenvirinae** | **Schizot4-like viruses** | 0.0000 | 0.0000 | 0.0536 | 0.0928 | -1.0000 | 0.4226 | 0.0000 | 0.0000 | 0.0000 | 0.0000 | NA | NA | 0.0000 | 0.0000 | 0.0000 | 0.0000 | NA | NA |
| **Myoviridae** | **Tevenvirinae** | **SP18-like viruses** | 1.5073 | 1.6277 | 0.7091 | 0.4866 | 0.8138 | 0.4898 | 0.0921 | 0.1595 | 0.4559 | 0.3992 | -1.4658 | 0.2513 | 2.8302 | 0.8280 | 0.1662 | 0.0892 | 5.5406 | 0.0295 |
| **Myoviridae** | **Tevenvirinae** | **T4-like viruses** | 8.4211 | 1.3570 | 8.0922 | 2.4021 | 0.2065 | 0.8490 | 6.8890 | 1.4929 | 4.7825 | 2.6135 | 1.2122 | 0.3077 | 8.2123 | 0.2707 | 1.1619 | 0.6098 | 18.3026 | 0.0006 |
| **Myoviridae** |  | **0305phi8-36-like viruses** | 0.1540 | 0.2667 | 0.0000 | 0.0000 | 1.0000 | 0.4226 | 0.0647 | 0.1121 | 0.0000 | 0.0000 | 1.0000 | 0.4226 | 0.0000 | 0.0000 | 0.0000 | 0.0000 | NA | NA |
| **Myoviridae** |  | **Bcep781-like viruses** | 1.9670 | 3.2090 | 1.3934 | 2.4134 | 0.2475 | 0.8176 | 1.2618 | 2.0196 | 0.1058 | 0.1833 | 0.9873 | 0.4261 | 0.8412 | 1.4570 | 0.0000 | 0.0000 | 1.0000 | 0.4226 |
| **Myoviridae** |  | **BcepMu-like viruses** | 0.0597 | 0.1035 | 0.0000 | 0.0000 | 1.0000 | 0.4226 | 0.0000 | 0.0000 | 0.0000 | 0.0000 | NA | NA | 0.0000 | 0.0000 | 0.0000 | 0.0000 | NA | NA |
| **Myoviridae** |  | **FelixO1-like viruses** | 0.0000 | 0.0000 | 0.0000 | 0.0000 | NA | NA | 0.0000 | 0.0000 | 0.0962 | 0.1666 | -1.0000 | 0.4226 | 0.1104 | 0.1912 | 0.0171 | 0.0296 | 0.8351 | 0.4880 |
| **Myoviridae** |  | **HAP1-like viruses** | 0.5065 | 0.6871 | 0.1921 | 0.1841 | 0.7657 | 0.5150 | 0.3966 | 0.3886 | 0.1133 | 0.0988 | 1.2240 | 0.3333 | 0.4363 | 0.5076 | 0.0597 | 0.0533 | 1.2781 | 0.3271 |
| **Myoviridae** |  | **P1-like viruses** | 0.2488 | 0.2582 | 0.6373 | 0.6423 | -0.9722 | 0.4117 | 0.4098 | 0.3628 | 0.1662 | 0.1593 | 1.0650 | 0.3715 | 0.6298 | 0.3641 | 0.0255 | 0.0441 | 2.8545 | 0.1006 |
| **Myoviridae** |  | **PAKP1-like viruses** | 0.0000 | 0.0000 | 0.0000 | 0.0000 | NA | NA | 0.0000 | 0.0000 | 0.0000 | 0.0000 | NA | NA | 0.0000 | 0.0000 | 0.0000 | 0.0000 | NA | NA |
| **Myoviridae** |  | **PB1-like viruses** | 0.0000 | 0.0000 | 0.0000 | 0.0000 | NA | NA | 0.0647 | 0.1121 | 0.0000 | 0.0000 | 1.0000 | 0.4226 | 0.0000 | 0.0000 | 0.0000 | 0.0000 | NA | NA |
| **Myoviridae** |  | **phiCD119-like viruses** | 12.8368 | 7.4496 | 12.0364 | 3.4769 | 0.1686 | 0.8774 | 15.0898 | 7.7731 | 9.3721 | 4.0904 | 1.1275 | 0.3409 | 11.4223 | 0.8767 | 3.7659 | 1.5875 | 7.3126 | 0.0046 |
| **Myoviridae** |  | **phiKZ-like viruses** | 0.5376 | 0.9312 | 0.0000 | 0.0000 | 1.0000 | 0.4226 | 0.1568 | 0.1419 | 0.1010 | 0.0878 | 0.5793 | 0.5992 | 0.0000 | 0.0000 | 0.0599 | 0.0646 | -1.6058 | 0.2495 |
| **Myoviridae** |  | **phiPLPE-like viruses** | 0.0000 | 0.0000 | 0.1309 | 0.1141 | -1.9873 | 0.1852 | 0.0921 | 0.1595 | 0.3736 | 0.3240 | -1.3502 | 0.2722 | 0.0000 | 0.0000 | 0.0896 | 0.0969 | -1.6018 | 0.2504 |
| **Myoviridae** |  | **rV5-like viruses** | 0.1367 | 0.1212 | 0.0000 | 0.0000 | 1.9539 | 0.1899 | 0.0647 | 0.1121 | 0.0000 | 0.0000 | 1.0000 | 0.4226 | 0.0000 | 0.0000 | 0.0556 | 0.0518 | -1.8572 | 0.2044 |
| **Myoviridae** |  | **Secunda5-like viruses** | 0.0000 | 0.0000 | 0.0000 | 0.0000 | NA | NA | 0.0647 | 0.1121 | 0.0481 | 0.0833 | 0.2062 | 0.8475 | 0.0000 | 0.0000 | 0.0000 | 0.0000 | NA | NA |
| **Myoviridae** |  | **SfV-like viruses** | 0.0000 | 0.0000 | 0.0000 | 0.0000 | NA | NA | 0.0000 | 0.0000 | 0.0000 | 0.0000 | NA | NA | 0.0000 | 0.0000 | 0.0000 | 0.0000 | NA | NA |
| **Myoviridae** |  | **unclassified** | 0.0770 | 0.1333 | 0.0000 | 0.0000 | 1.0000 | 0.4226 | 0.0647 | 0.1121 | 0.0000 | 0.0000 | 1.0000 | 0.4226 | 0.3259 | 0.3312 | 0.0599 | 0.0646 | 1.3654 | 0.2973 |
| **Myoviridae** |  | **VHML-like viruses** | 0.0000 | 0.0000 | 0.3487 | 0.6039 | -1.0000 | 0.4226 | 0.2066 | 0.3579 | 0.0000 | 0.0000 | 1.0000 | 0.4226 | 0.4421 | 0.7657 | 0.0000 | 0.0000 | 1.0000 | 0.4226 |
| **Myoviridae** |  | **Vi1-like viruses** | 0.0000 | 0.0000 | 0.0000 | 0.0000 | NA | NA | 0.0000 | 0.0000 | 0.0000 | 0.0000 | NA | NA | 0.0000 | 0.0000 | 0.0000 | 0.0000 | NA | NA |
| **Podoviridae** | **Autogrphivirinae** | **KP34-like viruses** | 0.0770 | 0.1333 | 0.0000 | 0.0000 | 1.0000 | 0.4226 | 0.2630 | 0.2953 | 0.0000 | 0.0000 | 1.5427 | 0.2629 | 0.0000 | 0.0000 | 0.0000 | 0.0000 | NA | NA |
| **Podoviridae** | **Autogrphivirinae** | **phiKMV-like viruses** | 0.1629 | 0.1417 | 0.0000 | 0.0000 | 1.9910 | 0.1847 | 0.0000 | 0.0000 | 0.0000 | 0.0000 | NA | NA | 0.0000 | 0.0000 | 0.0426 | 0.0389 | -1.8934 | 0.1988 |
| **Podoviridae** | **Autogrphivirinae** | **SP6-like viruses** | 0.0000 | 0.0000 | 0.1072 | 0.1856 | -1.0000 | 0.4226 | 0.2257 | 0.0442 | 0.1539 | 0.1589 | 0.7533 | 0.5206 | 0.1052 | 0.1821 | 0.0171 | 0.0296 | 0.8265 | 0.4916 |
| **Podoviridae** | **Autogrphivirinae** | **T7-like viruses** | 1.2000 | 0.8890 | 0.8118 | 0.5220 | 0.6521 | 0.5577 | 1.5026 | 0.5929 | 0.4388 | 0.2830 | 2.8047 | 0.0712 | 0.9783 | 0.3332 | 0.1615 | 0.1977 | 3.6518 | 0.0310 |
| **Podoviridae** | **Autogrphivirinae** | **unclassified** | 0.0000 | 0.0000 | 0.0000 | 0.0000 | NA | NA | 0.1942 | 0.3363 | 0.0000 | 0.0000 | 1.0000 | 0.4226 | 0.0000 | 0.0000 | 0.0214 | 0.0370 | -1.0000 | 0.4226 |
| **Podoviridae** | **Picovirinae** | **P68-like viruses** | 0.0000 | 0.0000 | 0.0000 | 0.0000 | NA | NA | 0.0000 | 0.0000 | 0.0000 | 0.0000 | NA | NA | 0.0000 | 0.0000 | 0.0000 | 0.0000 | NA | NA |
| **Podoviridae** | **Picovirinae** | **Phi29-like viruses** | 0.9927 | 0.2828 | 0.1223 | 0.2119 | 4.2669 | 0.0153 | 0.0000 | 0.0000 | 0.3078 | 0.3179 | -1.6773 | 0.2355 | 0.0000 | 0.0000 | 0.0509 | 0.0882 | -1.0000 | 0.4226 |
| **Podoviridae** | **Picovirinae** | **unclassified** | 0.0000 | 0.0000 | 0.0000 | 0.0000 | NA | NA | 0.0000 | 0.0000 | 0.0529 | 0.0916 | -1.0000 | 0.4226 | 0.0000 | 0.0000 | 0.0000 | 0.0000 | NA | NA |
| **Podoviridae** |  | **Bcep22-like viruses** | 0.5469 | 0.4848 | 0.0000 | 0.0000 | 1.9539 | 0.1899 | 0.1294 | 0.2242 | 0.1491 | 0.1445 | -0.1277 | 0.9056 | 0.0000 | 0.0000 | 0.0000 | 0.0000 | NA | NA |
| **Podoviridae** |  | **Bpp-1-like viruses** | 5.7940 | 5.2984 | 6.6203 | 4.2027 | -0.2116 | 0.8432 | 0.1568 | 0.1419 | 0.8454 | 1.4643 | -0.8107 | 0.5013 | 8.1025 | 7.0521 | 4.2111 | 1.0598 | 0.9452 | 0.4406 |
| **Podoviridae** |  | **Epsilon15-like viruses** | 0.0000 | 0.0000 | 0.0000 | 0.0000 | NA | NA | 0.0000 | 0.0000 | 0.0000 | 0.0000 | NA | NA | 0.0000 | 0.0000 | 0.0000 | 0.0000 | NA | NA |
| **Podoviridae** |  | **F116-like viruses** | 0.0000 | 0.0000 | 0.0000 | 0.0000 | NA | NA | 0.0000 | 0.0000 | 0.0000 | 0.0000 | NA | NA | 0.0000 | 0.0000 | 0.0639 | 0.0125 | -8.8299 | 0.0126 |
| **Podoviridae** |  | **LUZ24-like viruses** | 0.0000 | 0.0000 | 0.0000 | 0.0000 | NA | NA | 0.0000 | 0.0000 | 0.0000 | 0.0000 | NA | NA | 0.0884 | 0.1531 | 0.0171 | 0.0296 | 0.7919 | 0.5064 |
| **Podoviridae** |  | **N4-like viruses** | 0.0000 | 0.0000 | 0.0000 | 0.0000 | NA | NA | 0.0000 | 0.0000 | 0.0000 | 0.0000 | NA | NA | 0.1052 | 0.1821 | 0.0171 | 0.0296 | 0.8265 | 0.4916 |
| **Podoviridae** |  | **P22-like viruses** | 0.0859 | 0.1488 | 0.0536 | 0.0928 | 0.3192 | 0.7685 | 0.0647 | 0.1121 | 0.1587 | 0.2749 | -0.5484 | 0.6262 | 0.1104 | 0.1912 | 0.0428 | 0.0741 | 0.5712 | 0.6137 |
| **Podoviridae** |  | **unclassified** | 0.8502 | 1.2778 | 0.7678 | 0.3121 | 0.1084 | 0.9226 | 2.1396 | 2.5577 | 0.9577 | 1.0957 | 0.7357 | 0.5204 | 2.6204 | 1.8105 | 0.1913 | 0.2314 | 2.3051 | 0.1436 |
| **Siphoviridae** | **Guernseyvirinae** | **Jersey-like viruses** | 0.1195 | 0.2069 | 0.0000 | 0.0000 | 1.0000 | 0.4226 | 0.0000 | 0.0000 | 0.0000 | 0.0000 | NA | NA | 0.2103 | 0.3643 | 0.0000 | 0.0000 | 1.0000 | 0.4226 |
| **Siphoviridae** | **Tunavirinae** | **TLS-like viruses** | 0.0000 | 0.0000 | 0.0000 | 0.0000 | NA | NA | 0.0000 | 0.0000 | 0.0000 | 0.0000 | NA | NA | 0.0000 | 0.0000 | 0.0000 | 0.0000 | NA | NA |
| **Siphoviridae** |  | **1706-like viruses** | 6.4523 | 2.6415 | 10.6756 | 5.1347 | -1.2668 | 0.2949 | 15.2329 | 6.8744 | 5.5667 | 6.0723 | 1.8253 | 0.1431 | 6.3376 | 7.2468 | 0.6702 | 0.4909 | 1.3515 | 0.3081 |
| **Siphoviridae** |  | **3a-like viruses** | 0.0000 | 0.0000 | 0.1309 | 0.1141 | -1.9873 | 0.1852 | 0.1842 | 0.3190 | 0.0481 | 0.0833 | 0.7148 | 0.5410 | 0.0000 | 0.0000 | 0.0171 | 0.0296 | -1.0000 | 0.4226 |
| **Siphoviridae** |  | **77-like viruses** | 0.0000 | 0.0000 | 0.0000 | 0.0000 | NA | NA | 0.0000 | 0.0000 | 0.0604 | 0.1046 | -1.0000 | 0.4226 | 0.0000 | 0.0000 | 0.0000 | 0.0000 | NA | NA |
| **Siphoviridae** |  | **936-like viruses** | 0.4117 | 0.3890 | 0.2381 | 0.0734 | 0.7596 | 0.5222 | 0.1294 | 0.2242 | 2.0196 | 0.8150 | -3.8730 | 0.0482 | 0.7350 | 0.4075 | 1.3870 | 0.3393 | -2.1298 | 0.1025 |
| **Siphoviridae** |  | **c2-like viruses** | 0.8659 | 0.6126 | 0.6005 | 0.5239 | 0.5703 | 0.5997 | 0.2298 | 0.2105 | 0.0000 | 0.0000 | 1.8912 | 0.1992 | 1.2603 | 0.5973 | 0.1493 | 0.0450 | 3.2128 | 0.0835 |
| **Siphoviridae** |  | **Che8-like viruses** | 0.5139 | 0.2719 | 7.5284 | 8.7381 | -1.3897 | 0.2989 | 0.6587 | 0.6323 | 0.3474 | 0.3632 | 0.7396 | 0.5102 | 0.5027 | 0.1751 | 0.1193 | 0.1066 | 3.2398 | 0.0417 |
| **Siphoviridae** |  | **CJW1-like viruses** | 0.0770 | 0.1333 | 0.0000 | 0.0000 | 1.0000 | 0.4226 | 0.0000 | 0.0000 | 0.0529 | 0.0916 | -1.0000 | 0.4226 | 0.0884 | 0.1531 | 0.0000 | 0.0000 | 1.0000 | 0.4226 |
| **Siphoviridae** |  | **D3112-like viruses** | 0.0000 | 0.0000 | 0.0536 | 0.0928 | -1.0000 | 0.4226 | 0.0000 | 0.0000 | 0.0000 | 0.0000 | NA | NA | 0.0000 | 0.0000 | 0.0000 | 0.0000 | NA | NA |
| **Siphoviridae** |  | **D3-like viruses** | 0.0597 | 0.1035 | 0.0000 | 0.0000 | 1.0000 | 0.4226 | 0.0000 | 0.0000 | 0.0481 | 0.0833 | -1.0000 | 0.4226 | 0.1104 | 0.1912 | 0.0000 | 0.0000 | 1.0000 | 0.4226 |
| **Siphoviridae** |  | **HK578-like viruses** | 0.0000 | 0.0000 | 0.0000 | 0.0000 | NA | NA | 0.0647 | 0.1121 | 0.0000 | 0.0000 | 1.0000 | 0.4226 | 0.0000 | 0.0000 | 0.0000 | 0.0000 | NA | NA |
| **Siphoviridae** |  | **IEBH-like viruses** | 0.4028 | 0.3599 | 0.6061 | 0.2715 | -0.7810 | 0.4815 | 0.1942 | 0.3363 | 0.0604 | 0.1046 | 0.6579 | 0.5685 | 0.6623 | 1.1471 | 0.0000 | 0.0000 | 1.0000 | 0.4226 |
| **Siphoviridae** |  | **L5-like viruses** | 0.2987 | 0.5173 | 0.5277 | 0.5519 | -0.5245 | 0.6278 | 0.2987 | 0.3105 | 0.3832 | 0.1396 | -0.4298 | 0.6985 | 0.2872 | 0.2680 | 0.0811 | 0.0197 | 1.3290 | 0.3140 |
| **Siphoviridae** |  | **Lambda-like viruses** | 2.9789 | 1.3898 | 1.3291 | 1.5677 | 1.3639 | 0.2453 | 2.9196 | 2.5288 | 1.0010 | 0.9964 | 1.2227 | 0.3204 | 1.9549 | 1.5383 | 0.2179 | 0.3136 | 1.9164 | 0.1855 |
| **Siphoviridae** |  | **Lebron-like viruses** | 0.0000 | 0.0000 | 0.0000 | 0.0000 | NA | NA | 0.0000 | 0.0000 | 0.0000 | 0.0000 | NA | NA | 0.2155 | 0.1868 | 0.0000 | 0.0000 | 1.9982 | 0.1837 |
| **Siphoviridae** |  | **Omega-like viruses** | 0.1540 | 0.2667 | 0.0536 | 0.0928 | 0.6157 | 0.5900 | 0.0689 | 0.1193 | 0.0529 | 0.0916 | 0.1838 | 0.8637 | 0.0000 | 0.0000 | 0.0000 | 0.0000 | NA | NA |
| **Siphoviridae** |  | **P23-45-like viruses** | 0.0000 | 0.0000 | 0.0000 | 0.0000 | NA | NA | 0.0000 | 0.0000 | 0.0481 | 0.0833 | -1.0000 | 0.4226 | 0.0000 | 0.0000 | 0.0000 | 0.0000 | NA | NA |
| **Siphoviridae** |  | **P335-like viruses** | 3.4500 | 0.3520 | 3.4204 | 0.3255 | 0.1070 | 0.9200 | 4.6098 | 1.0816 | 20.7495 | 9.1758 | -3.0256 | 0.0910 | 4.4060 | 1.5133 | 43.8690 | 1.9631 | -27.5759 | 1.78E-05 |
| **Siphoviridae** |  | **PhiC31-like viruses** | 0.0000 | 0.0000 | 0.0000 | 0.0000 | NA | NA | 0.0000 | 0.0000 | 0.0000 | 0.0000 | NA | NA | 0.0000 | 0.0000 | 0.0000 | 0.0000 | NA | NA |
| **Siphoviridae** |  | **phiE125-like viruses** | 0.1367 | 0.1212 | 2.6053 | 2.6540 | -1.6094 | 0.2483 | 0.3593 | 0.0730 | 0.4708 | 0.6943 | -0.2767 | 0.8075 | 0.8292 | 0.9441 | 0.2523 | 0.2296 | 1.0284 | 0.4020 |
| **Siphoviridae** |  | **phiETA-like viruses** | 2.3381 | 1.8098 | 1.7181 | 0.5832 | 0.5647 | 0.6204 | 1.6901 | 0.1556 | 1.3157 | 0.4746 | 1.2983 | 0.3039 | 1.8226 | 1.1750 | 0.4779 | 0.1637 | 1.9632 | 0.1838 |
| **Siphoviridae** |  | **phiFL-like viruses** | 2.7101 | 1.5134 | 0.8820 | 1.0329 | 1.7281 | 0.1685 | 1.9288 | 2.6444 | 2.0757 | 1.2761 | -0.0867 | 0.9366 | 4.3354 | 3.4680 | 1.2152 | 0.1455 | 1.5570 | 0.2593 |
| **Siphoviridae** |  | **phiLJ1-like viruses** | 0.0770 | 0.1333 | 0.3604 | 0.1739 | -2.2401 | 0.0931 | 0.0689 | 0.1193 | 0.2405 | 0.4166 | -0.6861 | 0.5547 | 0.0000 | 0.0000 | 0.0000 | 0.0000 | NA | NA |
| **Siphoviridae** |  | **PsiM1-like viruses** | 0.0000 | 0.0000 | 0.0000 | 0.0000 | NA | NA | 0.0000 | 0.0000 | 0.0000 | 0.0000 | NA | NA | 0.0000 | 0.0000 | 0.0000 | 0.0000 | NA | NA |
| **Siphoviridae** |  | **Sfi11-like viruses** | 0.1195 | 0.2069 | 0.1931 | 0.2111 | -0.4312 | 0.6886 | 0.2066 | 0.3579 | 0.1566 | 0.1459 | 0.2242 | 0.8388 | 0.1052 | 0.1821 | 0.1580 | 0.0710 | -0.4684 | 0.6760 |

**Supplemental Table 5: *t-test* for Equality of Means of Viral Genera within the Order Caudovirales Continued**

Dependent Variable: Diet

|  |  |  |  | |  | | **Day 0** | | | |  | |  | |  | |  | | **2 Days PD** | | | |  | |  |  | |  | | **2 Weeks PD** | |  |  |
| --- | --- | --- | --- | --- | --- | --- | --- | --- | --- | --- | --- | --- | --- | --- | --- | --- | --- | --- | --- | --- | --- | --- | --- | --- | --- | --- | --- | --- | --- | --- | --- | --- | --- |
|  |  |  | **Chow** | | | | **WD** | | | | **F** | | **P value** | | **Chow** | | | | **WD** | | | | **F** | | **P value** | **Chow** | | | | **WD** | | **F** | **P value** |
| **Family** | **Subfamily** | **Genera** | **Mean** | | **SD** | | **Mean** | | **SD** | |  | |  | | **Mean** | | **SD** | | **Mean** | | **SD** | |  | |  | **Mean** | | **SD** | | **Mean** | **SD** |  |  |
| **Siphoviridae** |  | **Sfi21-like viruses** | 1.0968 | | 0.6445 | | 0.6692 | | 0.2623 | | 1.0644 | | 0.3745 | | 1.2363 | | 0.9571 | | 0.7615 | | 0.3073 | | 0.8181 | | 0.4863 | 0.5090 | | 0.6653 | | 0.7598 | 0.4545 | -0.5391 | 0.6220 |
| **Siphoviridae** |  | **SPbeta-like viruses** | 1.2154 | | 0.2627 | | 1.5528 | | 0.7656 | | -0.7220 | | 0.5327 | | 2.5945 | | 1.4866 | | 2.7880 | | 2.3691 | | -0.1199 | | 0.9114 | 1.3440 | | 1.2965 | | 0.5583 | 0.3071 | 1.0214 | 0.4051 |
| **Siphoviridae** |  | **T5-like viruses** | 0.0859 | | 0.1488 | | 0.2092 | | 0.3624 | | -0.5452 | | 0.6280 | | 0.0647 | | 0.1121 | | 0.0604 | | 0.1046 | | 0.0490 | | 0.9633 | 0.5090 | | 0.6653 | | 0.0000 | 0.0000 | 1.3252 | 0.3162 |
| **Siphoviridae** |  | **TM4-like viruses** | 0.0000 | | 0.0000 | | 0.0000 | | 0.0000 | | NA | | NA | | 0.0000 | | 0.0000 | | 0.0604 | | 0.1046 | | -1.0000 | | 0.4226 | 0.0884 | | 0.1531 | | 0.0000 | 0.0000 | 1.0000 | 0.4226 |
| **Siphoviridae** |  | **TP21-like viruses** | 0.0859 | | 0.1488 | | 0.0000 | | 0.0000 | | 1.0000 | | 0.4226 | | 0.0000 | | 0.0000 | | 0.0000 | | 0.0000 | | NA | | NA | 0.0000 | | 0.0000 | | 0.0000 | 0.0000 | NA | NA |
| **Siphoviridae** |  | **unclassified** | 28.5816 | | 0.7282 | | 25.6749 | | 6.0119 | | 0.8314 | | 0.4911 | | 28.7013 | | 6.3108 | | 37.4213 | | 3.1602 | | -2.1399 | | 0.1236 | 27.4438 | | 5.7454 | | 36.3721 | 2.9275 | -2.3982 | 0.0968 |
| **Siphoviridae** |  | **Wbeta-like viruses** | 0.0000 | | 0.0000 | | 0.0000 | | 0.0000 | | NA | | NA | | 0.0000 | | 0.0000 | | 0.0000 | | 0.0000 | | NA | | NA | 0.0000 | | 0.0000 | | 0.0000 | 0.0000 | NA | NA |
| **Siphoviridae** |  | **Xp10-like viruses** | 0.0000 | | 0.0000 | | 0.0000 | | 0.0000 | | NA | | NA | | 0.0689 | | 0.1193 | | 0.0604 | | 0.1046 | | 0.0926 | | 0.9307 | 0.0000 | | 0.0000 | | 0.0255 | 0.0441 | -1.0000 | 0.4226 |
| **Siphoviridae** |  | **Yua-like viruses** | 0.0859 | | 0.1488 | | 0.1395 | | 0.2416 | | -0.3270 | | 0.7632 | | 0.0647 | | 0.1121 | | 0.0000 | | 0.0000 | | 1.0000 | | 0.4226 | 0.0000 | | 0.0000 | | 0.0000 | 0.0000 | NA | NA |
|  |  |  | | **8 Weeks PD** | | | | | | | | | | | | **12 Weeks PD** | | | | | | | | | | | | |  |  |  |  |  |
|  |  |  | | **Chow** | | | | **WD** | | | | **F** | | **P value** | | **Chow** | | | | **WD** | | | | **F** | | | **P value** | |  |  |  |  |  |
| **Family** | **Subfamily** | **Genera** | | **Mean** | | **SD** | | **Mean** | | **SD** | |  | |  | | **Mean** | | **SD** | | **Mean** | | **SD** | |  | | |  | |  |  |  |  |  |
| **Myoviridae** | **Peduovirinae** | **Hp1-like viruses** | | 0.0000 | | 0.0000 | | 0.0452 | | 0.0782 | | -1.0000 | | 0.4226 | | 0.0326 | | 0.0564 | | 0.0000 | | 0.0000 | | 1.0000 | | | 0.4226 | |  |  |  |  |  |
| **Myoviridae** | **Peduovirinae** | **P2-like viruses** | | 0.5912 | | 0.3320 | | 0.2967 | | 0.1519 | | 1.3968 | | 0.2628 | | 0.5912 | | 0.6629 | | 0.0404 | | 0.0371 | | 1.4371 | | | 0.2865 | |  |  |  |  |  |
| **Myoviridae** | **Spounavirinae** | **K-like viruses** | | 0.0000 | | 0.0000 | | 0.0000 | | 0.0000 | | NA | | NA | | 0.0000 | | 0.0000 | | 0.0033 | | 0.0058 | | -1.0000 | | | 0.4226 | |  |  |  |  |  |
| **Myoviridae** | **Spounavirinae** | **P100-like viruses** | | 0.4009 | | 0.2084 | | 0.0601 | | 0.1040 | | 2.5343 | | 0.0869 | | 0.0138 | | 0.0239 | | 0.0067 | | 0.0116 | | 0.4656 | | | 0.6744 | |  |  |  |  |  |
| **Myoviridae** | **Spounavirinae** | **SPO1-like viruses** | | 2.2869 | | 1.5078 | | 1.5049 | | 2.0149 | | 0.5382 | | 0.6211 | | 0.7719 | | 0.6575 | | 0.8423 | | 0.5227 | | -0.1451 | | | 0.8920 | |  |  |  |  |  |
| **Myoviridae** | **Spounavirinae** | **Twort-like viruses** | | 1.0123 | | 0.9090 | | 0.2588 | | 0.2249 | | 1.3936 | | 0.2854 | | 0.8148 | | 1.1258 | | 0.0177 | | 0.0071 | | 1.2263 | | | 0.3449 | |  |  |  |  |  |
| **Myoviridae** | **Spounavirinae** | **Unclassified** | | 0.0838 | | 0.1451 | | 0.0000 | | 0.0000 | | 1.0000 | | 0.4226 | | 0.0591 | | 0.0851 | | 0.0263 | | 0.0246 | | 0.6417 | | | 0.5784 | |  |  |  |  |  |
| **Myoviridae** | **Tevenvirinae** | **JS98-like viruses** | | 0.0000 | | 0.0000 | | 0.0000 | | 0.0000 | | NA | | NA | | 0.0000 | | 0.0000 | | 0.0078 | | 0.0135 | | -1.0000 | | | 0.4226 | |  |  |  |  |  |
| **Myoviridae** | **Tevenvirinae** | **RB49-like** | | 0.0000 | | 0.0000 | | 0.0000 | | 0.0000 | | NA | | NA | | 0.0587 | | 0.0517 | | 0.0000 | | 0.0000 | | 1.9647 | | | 0.1884 | |  |  |  |  |  |
| **Myoviridae** | **Tevenvirinae** | **Schizot4-like viruses** | | 0.0000 | | 0.0000 | | 0.0000 | | 0.0000 | | NA | | NA | | 0.0000 | | 0.0000 | | 0.0033 | | 0.0058 | | -1.0000 | | | 0.4226 | |  |  |  |  |  |
| **Myoviridae** | **Tevenvirinae** | **SP18-like viruses** | | 0.4392 | | 0.5143 | | 0.6607 | | 1.1443 | | -0.3058 | | 0.7812 | | 0.2868 | | 0.2515 | | 0.3770 | | 0.1649 | | -0.5194 | | | 0.6350 | |  |  |  |  |  |
| **Myoviridae** | **Tevenvirinae** | **T4-like viruses** | | 8.2118 | | 2.4985 | | 3.8290 | | 0.4657 | | 2.9869 | | 0.0887 | | 4.8000 | | 3.0090 | | 0.4226 | | 0.2444 | | 2.5114 | | | 0.1270 | |  |  |  |  |  |
| **Myoviridae** |  | **0305phi8-36-like viruses** | | 0.0000 | | 0.0000 | | 0.0000 | | 0.0000 | | NA | | NA | | 0.0330 | | 0.0406 | | 0.0033 | | 0.0056 | | 1.2583 | | | 0.3313 | |  |  |  |  |  |
| **Myoviridae** |  | **Bcep781-like viruses** | | 1.1260 | | 1.5357 | | 0.0000 | | 0.0000 | | 1.2699 | | 0.3319 | | 0.8353 | | 1.4468 | | 0.0228 | | 0.0395 | | 0.9723 | | | 0.4333 | |  |  |  |  |  |
| **Myoviridae** |  | **BcepMu-like viruses** | | 0.1065 | | 0.1845 | | 0.0000 | | 0.0000 | | 1.0000 | | 0.4226 | | 0.0035 | | 0.0060 | | 0.0000 | | 0.0000 | | 1.0000 | | | 0.4226 | |  |  |  |  |  |
| **Myoviridae** |  | **FelixO1-like viruses** | | 0.0000 | | 0.0000 | | 0.0000 | | 0.0000 | | NA | | NA | | 0.1374 | | 0.2203 | | 0.0000 | | 0.0000 | | 1.0804 | | | 0.3929 | |  |  |  |  |  |
| **Myoviridae** |  | **HAP1-like viruses** | | 0.1042 | | 0.1804 | | 0.0452 | | 0.0782 | | 0.5196 | | 0.6425 | | 0.0104 | | 0.0180 | | 0.0244 | | 0.0152 | | -1.0338 | | | 0.3612 | |  |  |  |  |  |
| **Myoviridae** |  | **P1-like viruses** | | 0.1065 | | 0.1845 | | 0.0601 | | 0.1040 | | 0.3798 | | 0.7282 | | 0.0296 | | 0.0425 | | 0.0000 | | 0.0000 | | 1.2037 | | | 0.3519 | |  |  |  |  |  |
| **Myoviridae** |  | **PAKP1-like viruses** | | 0.0000 | | 0.0000 | | 0.0000 | | 0.0000 | | NA | | NA | | 0.0000 | | 0.0000 | | 0.0033 | | 0.0058 | | -1.0000 | | | 0.4226 | |  |  |  |  |  |
| **Myoviridae** |  | **PB1-like viruses** | | 0.0000 | | 0.0000 | | 0.0000 | | 0.0000 | | NA | | NA | | 0.0000 | | 0.0000 | | 0.0000 | | 0.0000 | | NA | | | NA | |  |  |  |  |  |
| **Myoviridae** |  | **phiCD119-like viruses** | | 15.5147 | | 7.6764 | | 7.6023 | | 5.2795 | | 1.4710 | | 0.2240 | | 10.5220 | | 7.4833 | | 0.4676 | | 0.2978 | | 2.3253 | | | 0.1452 | |  |  |  |  |  |
| **Myoviridae** |  | **phiKZ-like viruses** | | 0.0000 | | 0.0000 | | 0.2705 | | 0.2703 | | -1.7336 | | 0.2251 | | 0.1340 | | 0.2231 | | 0.0137 | | 0.0051 | | 0.9330 | | | 0.4492 | |  |  |  |  |  |
| **Myoviridae** |  | **phiPLPE-like viruses** | | 0.2083 | | 0.3608 | | 0.0601 | | 0.1040 | | 0.6839 | | 0.5558 | | 0.1055 | | 0.0477 | | 0.0000 | | 0.0000 | | 3.8318 | | | 0.0619 | |  |  |  |  |  |
| **Myoviridae** |  | **rV5-like viruses** | | 0.0000 | | 0.0000 | | 0.0000 | | 0.0000 | | NA | | NA | | 0.0326 | | 0.0564 | | 0.0066 | | 0.0057 | | 0.7933 | | | 0.5093 | |  |  |  |  |  |
| **Myoviridae** |  | **Secunda5-like viruses** | | 0.0000 | | 0.0000 | | 0.0000 | | 0.0000 | | NA | | NA | | 0.0720 | | 0.1073 | | 0.0000 | | 0.0000 | | 1.1627 | | | 0.3649 | |  |  |  |  |  |
| **Myoviridae** |  | **SfV-like viruses** | | 0.0000 | | 0.0000 | | 0.0000 | | 0.0000 | | NA | | NA | | 0.0000 | | 0.0000 | | 0.0000 | | 0.0000 | | NA | | | NA | |  |  |  |  |  |
| **Myoviridae** |  | **unclassified** | | 0.1065 | | 0.1845 | | 0.0411 | | 0.0712 | | 0.5729 | | 0.6127 | | 0.0138 | | 0.0239 | | 0.0172 | | 0.0112 | | -0.2190 | | | 0.8414 | |  |  |  |  |  |
| **Myoviridae** |  | **VHML-like viruses** | | 0.0000 | | 0.0000 | | 0.2258 | | 0.3912 | | -1.0000 | | 0.4226 | | 0.0069 | | 0.0120 | | 0.0000 | | 0.0000 | | 1.0000 | | | 0.4226 | |  |  |  |  |  |
| **Myoviridae** |  | **Vi1-like viruses** | | 0.0000 | | 0.0000 | | 0.0000 | | 0.0000 | | NA | | NA | | 0.0000 | | 0.0000 | | 0.0000 | | 0.0000 | | NA | | | NA | |  |  |  |  |  |
| **Podoviridae** | **Autogrphivirinae** | **KP34-like viruses** | | 0.1042 | | 0.1804 | | 0.0411 | | 0.0712 | | 0.5632 | | 0.6181 | | 0.0395 | | 0.0515 | | 0.0066 | | 0.0057 | | 1.0997 | | | 0.3838 | |  |  |  |  |  |
| **Podoviridae** | **Autogrphivirinae** | **phiKMV-like viruses** | | 0.0000 | | 0.0000 | | 0.0000 | | 0.0000 | | NA | | NA | | 0.0000 | | 0.0000 | | 0.0000 | | 0.0000 | | NA | | | NA | |  |  |  |  |  |
| **Podoviridae** | **Autogrphivirinae** | **SP6-like viruses** | | 0.0000 | | 0.0000 | | 0.0000 | | 0.0000 | | NA | | NA | | 0.0104 | | 0.0180 | | 0.0137 | | 0.0148 | | -0.2458 | | | 0.8184 | |  |  |  |  |  |
| **Podoviridae** | **Autogrphivirinae** | **T7-like viruses** | | 0.3125 | | 0.5413 | | 0.0452 | | 0.0782 | | 0.8467 | | 0.4832 | | 0.0484 | | 0.0838 | | 0.0744 | | 0.0346 | | -0.4967 | | | 0.6575 | |  |  |  |  |  |
| **Podoviridae** | **Autogrphivirinae** | **unclassified** | | 0.0000 | | 0.0000 | | 0.0000 | | 0.0000 | | NA | | NA | | 0.0035 | | 0.0060 | | 0.0000 | | 0.0000 | | 1.0000 | | | 0.4226 | |  |  |  |  |  |
| **Podoviridae** | **Picovirinae** | **P68-like viruses** | | 0.0000 | | 0.0000 | | 0.0601 | | 0.1040 | | -1.0000 | | 0.4226 | | 0.2043 | | 0.2318 | | 0.7580 | | 0.0912 | | -3.8499 | | | 0.0396 | |  |  |  |  |  |
| **Podoviridae** | **Picovirinae** | **Phi29-like viruses** | | 0.5004 | | 0.6612 | | 0.1355 | | 0.2347 | | 0.9009 | | 0.4460 | | 0.4277 | | 0.5866 | | 0.0773 | | 0.1240 | | 1.0121 | | | 0.4105 | |  |  |  |  |  |
| **Podoviridae** | **Picovirinae** | **unclassified** | | 0.0000 | | 0.0000 | | 0.0000 | | 0.0000 | | NA | | NA | | 0.0000 | | 0.0000 | | 0.0000 | | 0.0000 | | NA | | | NA | |  |  |  |  |  |
| **Podoviridae** |  | **Bcep22-like viruses** | | 0.0838 | | 0.1451 | | 0.1766 | | 0.2084 | | -0.6333 | | 0.5648 | | 0.6732 | | 0.8565 | | 0.0455 | | 0.0617 | | 1.2659 | | | 0.3319 | |  |  |  |  |  |
| **Podoviridae** |  | **Bpp-1-like viruses** | | 3.0988 | | 5.3673 | | 12.1150 | | 3.3297 | | -2.4724 | | 0.0812 | | 3.0920 | | 1.9101 | | 0.6337 | | 0.5812 | | 2.1325 | | | 0.1465 | |  |  |  |  |  |
| **Podoviridae** |  | **Epsilon15-like viruses** | | 0.0000 | | 0.0000 | | 0.0000 | | 0.0000 | | NA | | NA | | 0.0000 | | 0.0000 | | 0.0000 | | 0.0000 | | NA | | | NA | |  |  |  |  |  |
| **Podoviridae** |  | **F116-like viruses** | | 0.1042 | | 0.1804 | | 0.0411 | | 0.0712 | | 0.5632 | | 0.6181 | | 0.0000 | | 0.0000 | | 0.0065 | | 0.0113 | | -1.0000 | | | 0.4226 | |  |  |  |  |  |
| **Podoviridae** |  | **LUZ24-like viruses** | | 0.0000 | | 0.0000 | | 0.0000 | | 0.0000 | | NA | | NA | | 0.0104 | | 0.0180 | | 0.0065 | | 0.0113 | | 0.3146 | | | 0.7716 | |  |  |  |  |  |
| **Podoviridae** |  | **N4-like viruses** | | 0.0000 | | 0.0000 | | 0.0000 | | 0.0000 | | NA | | NA | | 0.0000 | | 0.0000 | | 0.0000 | | 0.0000 | | NA | | | NA | |  |  |  |  |  |
| **Podoviridae** |  | **P22-like viruses** | | 0.2083 | | 0.3608 | | 0.2105 | | 0.1877 | | -0.0090 | | 0.9934 | | 0.0035 | | 0.0060 | | 0.0039 | | 0.0067 | | -0.0841 | | | 0.9371 | |  |  |  |  |  |
| **Podoviridae** |  | **unclassified** | | 0.4392 | | 0.5143 | | 0.2696 | | 0.1949 | | 0.5339 | | 0.6362 | | 0.1208 | | 0.0975 | | 0.0742 | | 0.0671 | | 0.6818 | | | 0.5373 | |  |  |  |  |  |
| **Siphoviridae** | **Guernseyvirinae** | **Jersey-like viruses** | | 0.0000 | | 0.0000 | | 0.0903 | | 0.1565 | | -1.0000 | | 0.4226 | | 0.0326 | | 0.0564 | | 0.0000 | | 0.0000 | | 1.0000 | | | 0.4226 | |  |  |  |  |  |
| **Siphoviridae** | **Tunavirinae** | **TLS-like viruses** | | 0.0838 | | 0.1451 | | 0.0000 | | 0.0000 | | 1.0000 | | 0.4226 | | 0.0000 | | 0.0000 | | 0.0000 | | 0.0000 | | NA | | | NA | |  |  |  |  |  |
| **Siphoviridae** |  | **1706-like viruses** | | 6.6067 | | 3.7087 | | 2.4496 | | 1.4882 | | 1.8018 | | 0.1822 | | 6.6811 | | 4.9249 | | 0.7829 | | 0.4416 | | 2.0661 | | | 0.1728 | |  |  |  |  |  |
| **Siphoviridae** |  | **3a-like viruses** | | 0.5025 | | 0.8704 | | 0.0903 | | 0.1565 | | 0.8073 | | 0.4998 | | 0.1305 | | 0.2261 | | 0.0165 | | 0.0152 | | 0.8714 | | | 0.4747 | |  |  |  |  |  |
| **Siphoviridae** |  | **77-like viruses** | | 0.0000 | | 0.0000 | | 0.0000 | | 0.0000 | | NA | | NA | | 0.0587 | | 0.0517 | | 0.0000 | | 0.0000 | | 1.9647 | | | 0.1884 | |  |  |  |  |  |
| **Siphoviridae** |  | **936-like viruses** | | 0.3782 | | 0.1077 | | 1.7605 | | 1.2044 | | -1.9799 | | 0.1843 | | 0.2275 | | 0.1295 | | 0.4176 | | 0.2288 | | -1.2526 | | | 0.2950 | |  |  |  |  |  |
| **Siphoviridae** |  | **c2-like viruses** | | 0.5480 | | 0.5087 | | 0.1423 | | 0.1276 | | 1.3401 | | 0.2994 | | 0.0695 | | 0.0798 | | 0.0402 | | 0.0253 | | 0.6056 | | | 0.5971 | |  |  |  |  |  |
| **Siphoviridae** |  | **Che8-like viruses** | | 1.1934 | | 0.8628 | | 0.2402 | | 0.4161 | | 1.7235 | | 0.1870 | | 0.3048 | | 0.1802 | | 0.0169 | | 0.0201 | | 2.7504 | | | 0.1078 | |  |  |  |  |  |
| **Siphoviridae** |  | **CJW1-like viruses** | | 0.0838 | | 0.1451 | | 0.0000 | | 0.0000 | | 1.0000 | | 0.4226 | | 0.0000 | | 0.0000 | | 0.0000 | | 0.0000 | | NA | | | NA | |  |  |  |  |  |
| **Siphoviridae** |  | **D3112-like viruses** | | 0.0000 | | 0.0000 | | 0.0000 | | 0.0000 | | NA | | NA | | 0.0000 | | 0.0000 | | 0.0039 | | 0.0067 | | -1.0000 | | | 0.4226 | |  |  |  |  |  |
| **Siphoviridae** |  | **D3-like viruses** | | 0.1065 | | 0.1845 | | 0.0000 | | 0.0000 | | 1.0000 | | 0.4226 | | 0.0035 | | 0.0060 | | 0.0000 | | 0.0000 | | 1.0000 | | | 0.4226 | |  |  |  |  |  |
| **Siphoviridae** |  | **HK578-like viruses** | | 0.0000 | | 0.0000 | | 0.0000 | | 0.0000 | | NA | | NA | | 0.0000 | | 0.0000 | | 0.0000 | | 0.0000 | | NA | | | NA | |  |  |  |  |  |
| **Siphoviridae** |  | **IEBH-like viruses** | | 2.2737 | | 0.9158 | | 1.7514 | | 0.8865 | | 0.7097 | | 0.5171 | | 2.6351 | | 1.6542 | | 1.0094 | | 0.1482 | | 1.6954 | | | 0.2301 | |  |  |  |  |  |
| **Siphoviridae** |  | **L5-like viruses** | | 0.6092 | | 0.3441 | | 0.1274 | | 0.1235 | | 2.2830 | | 0.1239 | | 0.2316 | | 0.1979 | | 0.0928 | | 0.1524 | | 0.9627 | | | 0.3935 | |  |  |  |  |  |

**Supplemental Table 5: *t-test* for Equality of Means of Viral Genera within the Order Caudovirales Continued**

Dependent Variable: Diet

|  |  |  | **8 Weeks PD** | | | | | | **12 Weeks PD** | | | | | |
| --- | --- | --- | --- | --- | --- | --- | --- | --- | --- | --- | --- | --- | --- | --- |
|  |  |  | **Chow** | | **WD** | | **F** | **P value** | **Chow** | | **WD** | | **F** | **P value** |
| **Family** | **Subfamily** | **Genera** | **Mean** | **SD** | **Mean** | **SD** |  |  | **Mean** | **SD** | **Mean** | **SD** |  |  |
| **Siphoviridae** |  | **Lambda-like viruses** | 1.2157 | 1.5583 | 2.5347 | 1.8916 | -0.9322 | 0.4058 | 1.2354 | 1.3038 | 0.2067 | 0.1944 | 1.3517 | 0.3042 |
| **Siphoviridae** |  | **Lebron-like viruses** | 0.0000 | 0.0000 | 0.0000 | 0.0000 | NA | NA | 0.0000 | 0.0000 | 0.0000 | 0.0000 | NA | NA |
| **Siphoviridae** |  | **Omega-like viruses** | 0.0000 | 0.0000 | 0.0411 | 0.0712 | -1.0000 | 0.4226 | 0.0977 | 0.1691 | 0.0033 | 0.0058 | 0.9652 | 0.4361 |
| **Siphoviridae** |  | **P23-45-like viruses** | 0.0000 | 0.0000 | 0.0000 | 0.0000 | NA | NA | 0.0000 | 0.0000 | 0.0000 | 0.0000 | NA | NA |
| **Siphoviridae** |  | **P335-like viruses** | 4.3599 | 1.2686 | 22.0336 | 0.8216 | -20.2533 | 0.0001 | 11.4235 | 13.9236 | 25.1686 | 5.1619 | -1.6032 | 0.2233 |
| **Siphoviridae** |  | **PhiC31-like viruses** | 0.0000 | 0.0000 | 0.0000 | 0.0000 | NA | NA | 0.0000 | 0.0000 | 0.0000 | 0.0000 | NA | NA |
| **Siphoviridae** |  | **phiE125-like viruses** | 0.7088 | 1.0178 | 0.3265 | 0.3415 | 0.6167 | 0.5900 | 1.1938 | 0.8536 | 0.1477 | 0.0509 | 2.1187 | 0.1674 |
| **Siphoviridae** |  | **phiETA-like viruses** | 2.6903 | 0.2826 | 0.8296 | 0.5517 | 5.1990 | 0.0140 | 1.8906 | 0.3433 | 3.3322 | 1.2104 | -1.9845 | 0.1678 |
| **Siphoviridae** |  | **phiFL-like viruses** | 3.6961 | 1.5337 | 2.7654 | 1.2694 | 0.8097 | 0.4650 | 8.3100 | 6.2314 | 16.3929 | 1.6192 | -2.1745 | 0.1464 |
| **Siphoviridae** |  | **phiLJ1-like viruses** | 0.1879 | 0.1656 | 0.0000 | 0.0000 | 1.9655 | 0.1883 | 0.1272 | 0.1475 | 0.0261 | 0.0280 | 1.1668 | 0.3566 |
| **Siphoviridae** |  | **PsiM1-like viruses** | 0.1042 | 0.1804 | 0.0000 | 0.0000 | 1.0000 | 0.4226 | 0.0000 | 0.0000 | 0.0000 | 0.0000 | NA | NA |
| **Siphoviridae** |  | **Sfi11-like viruses** | 0.2083 | 0.3608 | 0.7384 | 0.4192 | -1.6599 | 0.1738 | 0.0138 | 0.0239 | 0.0763 | 0.0214 | -3.3664 | 0.0287 |
| **Siphoviridae** |  | **Sfi21-like viruses** | 2.5383 | 0.9606 | 0.4051 | 0.1963 | 3.7683 | 0.0562 | 0.3598 | 0.2834 | 0.0908 | 0.0407 | 1.6269 | 0.2404 |
| **Siphoviridae** |  | **SPbeta-like viruses** | 2.4476 | 1.5687 | 0.6007 | 0.3085 | 2.0010 | 0.1742 | 1.5220 | 1.0869 | 0.3220 | 0.0394 | 1.9109 | 0.1959 |
| **Siphoviridae** |  | **T5-like viruses** | 0.0000 | 0.0000 | 0.0601 | 0.1040 | -1.0000 | 0.4226 | 0.0104 | 0.0180 | 0.0000 | 0.0000 | 1.0000 | 0.4226 |
| **Siphoviridae** |  | **TM4-like viruses** | 0.0000 | 0.0000 | 0.0000 | 0.0000 | NA | NA | 0.0000 | 0.0000 | 0.0033 | 0.0058 | -1.0000 | 0.4226 |
| **Siphoviridae** |  | **TP21-like viruses** | 0.4237 | 0.4888 | 0.0822 | 0.1424 | 1.1616 | 0.3502 | 0.8637 | 0.8872 | 1.6709 | 0.6208 | -1.2912 | 0.2737 |
| **Siphoviridae** |  | **unclassified** | 31.3296 | 9.1759 | 31.2181 | 1.6010 | 0.0207 | 0.9852 | 31.2250 | 9.8317 | 45.9063 | 2.4416 | -2.5102 | 0.1151 |
| **Siphoviridae** |  | **Wbeta-like viruses** | 0.0000 | 0.0000 | 0.0601 | 0.1040 | -1.0000 | 0.4226 | 0.0346 | 0.0599 | 0.0486 | 0.0017 | -0.4048 | 0.7248 |
| **Siphoviridae** |  | **Xp10-like viruses** | 0.0000 | 0.0000 | 0.0000 | 0.0000 | NA | NA | 0.0000 | 0.0000 | 0.0000 | 0.0000 | NA | NA |
| **Siphoviridae** |  | **Yua-like viruses** | 0.0000 | 0.0000 | 0.0000 | 0.0000 | NA | NA | 0.0000 | 0.0000 | 0.0000 | 0.0000 | NA | NA |
