# Supplementary material for: Integrative Longitudinal Analysis of Metabolic Phenotype and Microbiota Changes During the Development of Obesity
Source: Front Cell Infect Microbiol. 2021 Aug 3;11:671926. doi: 10.3389/fcimb.2021.671926 (PMC8370388; doi:10.3389/fcimb.2021.671926)
Supplement: Supplementary file 7 [file Table_6.docx]

**Supplemental Table 6: *t-test* for Equality of Means of Diversity and Evenness**

Dependent Variable: Diet

|  | **Day 0** | | | | | | **2 Days PD** | | | | | | **2 Weeks PD** | | | | | |  |
| --- | --- | --- | --- | --- | --- | --- | --- | --- | --- | --- | --- | --- | --- | --- | --- | --- | --- | --- | --- |
|  | **Chow** | | **WD** | | **F** | **P value** | **Chow** | | **WD** | | **F** | **P value** | **Chow** | | **WD** | | **F** | **P value** |  |
|  | **Mean** | **SD** | **Mean** | **SD** |  |  | **Mean** | **SD** | **Mean** | **SD** |  |  | **Mean** | **SD** | **Mean** | **SD** |  |  | |
| **Bacterial OTUs Shannon Diversity** | 3.1375 | 0.0597 | 3.1548 | 0.0482 | -0.3901 | 0.7172 | 3.1640 | 0.0158 | 3.1402 | 0.0638 | 0.6280 | 0.5879 | 3.1784 | 0.0032 | 2.9484 | 0.2165 | 1.8393 | 0.2072 | |
| **Bacterial OTUs Evenness** | 0.4954 | 0.0104 | 0.4985 | 0.0072 | -0.4296 | 0.6922 | 0.4994 | 0.0033 | 0.4971 | 0.0091 | 0.4160 | 0.7103 | 0.5027 | 0.0010 | 0.4670 | 0.0353 | 1.7495 | 0.2221 | |
| **Bacteriophage OTUs Shannon Diversity** | 2.5325 | 0.1777 | 2.5182 | 0.0705 | 0.1296 | 0.9062 | 2.3772 | 0.1729 | 2.1004 | 0.2683 | 1.5018 | 0.2193 | 2.5640 | 0.0839 | 1.5507 | 0.0590 | 17.1128 | 0.0001 | |
| **Bacteriophage OTUs Evenness** | 0.7277 | 0.0421 | 0.7364 | 0.0301 | -0.2917 | 0.7864 | 0.6878 | 0.0796 | 0.6099 | 0.0743 | 1.2404 | 0.2829 | 0.7515 | 0.0263 | 0.4509 | 0.0269 | 13.8481 | 0.0002 | |
|  | **8 Weeks PD** | | | | | | **12 Weeks PD** | | | | | |  |  |  |  |  |  |  |
|  | **Chow** | | **WD** | | **F** | **P value** | **Chow** | | **WD** | | **F** | **P value** |  |  |  |  |  |  |  |
|  | **Mean** | **SD** | **Mean** | **SD** |  |  | **Mean** | **SD** | **Mean** | **SD** |  |  |  |  |  |  |  |  |  |
| **Bacterial OTUs Shannon Diversity** | 3.2370 | 0.0366 | 3.0810 | 0.2365 | 1.1294 | 0.3714 | 3.0898 | 0.1958 | 2.2475 | 0.1097 | 6.5003 | 0.0064 |  |  |  |  |  |  |  |
| **Bacterial OTUs Evenness** | 0.5105 | 0.0061 | 0.4893 | 0.0374 | 0.9687 | 0.4304 | 0.4840 | 0.0326 | 0.3534 | 0.0176 | 6.1082 | 0.0082 |  |  |  |  |  |  |  |
| **Bacteriophage OTUs Shannon Diversity** | 2.4466 | 0.2471 | 2.1885 | 0.0351 | 1.7913 | 0.2102 | 2.2650 | 0.4908 | 1.5759 | 0.0616 | 2.4128 | 0.1334 |  |  |  |  |  |  |  |
| **Bacteriophage OTUs Evenness** | 0.7194 | 0.0514 | 0.6481 | 0.0097 | 2.3588 | 0.1340 | 0.6189 | 0.1540 | 0.4263 | 0.0140 | 2.1576 | 0.1616 |  |  |  |  |  |  |  |
