# Supplementary material for: Integrative Longitudinal Analysis of Metabolic Phenotype and Microbiota Changes During the Development of Obesity
Source: Front Cell Infect Microbiol. 2021 Aug 3;11:671926. doi: 10.3389/fcimb.2021.671926 (PMC8370388; doi:10.3389/fcimb.2021.671926)
Supplement: Supplementary file 8 [file Table_7.docx]

**Supplemental Table 7: Diversity and Evenness Trend Analysis**

Mann-Kendall Trend Test

|  |  | **N** | **Z** | **S** | **VarS** | **Tau** | **P value** |
| --- | --- | --- | --- | --- | --- | --- | --- |
| **Bacterial OTUs Shannon Diversity** | Chow | 18 | 1.288 | 35 | 697 | 0.228758 | 0.901 |
|  | WD | 18 | -2.197 | -59 | 697 | -0.38562 | 0.014 |
| **Bacterial OTUs Evenness** | Chow | 18 | 0.833 | 23 | 697 | 0.150327 | 0.798 |
|  | WD | 18 | -2.045 | -55 | 697 | -0.35948 | 0.020 |
| **Bacteriophage OTUs Shannon Diversity** | Chow | 18 | -0.303 | -9 | 697 | -0.05882 | 0.381 |
|  | WD | 18 | -2.424 | -65 | 697 | -0.42484 | 0.008 |
| **Bacteriophage OTUs Evenness** | Chow | 18 | -0.985 | -27 | 697 | -0.17647 | 0.162 |
|  | WD | 18 | -2.803 | -75 | 697 | -0.4902 | 0.003 |
