# Supplementary material for: Integrative Longitudinal Analysis of Metabolic Phenotype and Microbiota Changes During the Development of Obesity
Source: Front Cell Infect Microbiol. 2021 Aug 3;11:671926. doi: 10.3389/fcimb.2021.671926 (PMC8370388; doi:10.3389/fcimb.2021.671926)
Supplement: Supplementary file 9 [file Table_8.docx]

**Supplemental Table 8: Bacteriophage Genera Characteristics**

Information is based on annotated species within the order Caudovirales

| **Family** | **Subfamily** | **Genera** | **Lifestyle Present Within Sample** | **Host Family** | **Virus/Host Correlation Coefficient at 12 wk PD** |
| --- | --- | --- | --- | --- | --- |
| Myoviridae |  | 0305phi8-36-like viruses | Virulent | Bacillaceae | -0.231 |
| Siphoviridae |  | 1706-like viruses | Virulent | Streptococcaceae | -0.8433 |
| Siphoviridae |  | 3a-like viruses | Virulent and Temperate | Staphylococcaceae | 0.0595 |
| Siphoviridae |  | 77-like viruses | Temperate | Staphylococcaceae | -0.7993 |
| Siphoviridae |  | 936-like viruses | Virulent | Streptococcaceae | 0.4059 |
| Podoviridae |  | Bcep22-like viruses | Virulent | Burkholderiaceae | 0.4717 |
| Myoviridae |  | Bcep781-like viruses | Virulent | Burkholderiaceae | 0.4188 |
| Myoviridae |  | BcepMu-like viruses | Temperate | Burkholderiaceae | 0.4214 |
| Podoviridae |  | Bpp-1-like viruses | Temperate | Alcaligenaceae, Burkholderiaceae | NA / 0.7532 |
| Siphoviridae |  | c2-like viruses | Virulent | Streptococcaceae | -0.1031 |
| Siphoviridae |  | Che8-like viruses | Mostly Temperate (1 Unknown) | Enterobacteriaceae | 0.7344 |
| Siphoviridae |  | CJW1-like viruses | Virulent | Mycobacteriaceae | NA |
| unclassified |  | Cvm10-like viruses | Virulent | Enterobacteriaceae | NA |
| Siphoviridae |  | D3112-like viruses | Unknown | Pseudomonadaceae | 0.5432 |
| Siphoviridae |  | D3-like viruses | Temperate | Pseudomonadaceae | 0.1572 |
| Podoviridae |  | Epsilon15-like viruses | Temperate | Enterobacteriaceae | NA |
| Podoviridae |  | F116-like viruses | Temperate | Pseudomonadaceae | 0.3408 |
| Myoviridae |  | FelixO1-like viruses | Virulent | Enterobacteriaceae | 0.5198 |
| Myoviridae |  | HAP1-like viruses | Temperate | Halomonadaceae | NA |
| Siphoviridae |  | HK578-like viruses | Virulent | Enterobacteriaceae | NA |
| Myoviridae | Peduovirinae | HP1-like viruses | Temperate | Pasteurellaceae | 0.5005 |
| Siphoviridae |  | IEBH-like viruses | Temperate | Bacillaceae | -0.7951 |
| Siphoviridae | Guernseyvirinae | Jersey-like viruses | Virulent | Enterobacteriaceae | 0.336 |
| Myoviridae | Tevenvirinae | JS98-like viruses | Unknown | Enterobacteriaceae | -0.7763 |
| Myoviridae | Spounavirinae | K-like viruses | Virulent | Staphylococcaceae | 0.2431 |
| Podoviridae | Autogrphivirinae | KP34-like viruses | Virulent | Enterobacteriaceae | 0.3934 |
| Siphoviridae |  | L5-like viruses | Virulent and Temperate | Mycobacteriaceae | 0.1947 |
| Siphoviridae |  | Lambda-like viruses | Temperate | Multiple | NA |
| Siphoviridae |  | Lebron-like viruses | Temperate | Mycobacteriaceae | NA |
| Podoviridae |  | LUZ24-like viruses | Temperate | Pseudomonadaceae | 0.472 |
| Podoviridae |  | N4-like viruses | Virulent | Enterobacteriaceae | NA |
| Siphoviridae |  | Omega-like viruses | Temperate | Mycobacteriaceae | 0.6081 |
| Myoviridae | Spounavirinae | P100-like viruses | Virulent | Listeriaceae | 0.2422 |
| Myoviridae |  | P1-like viruses | Temperate | Enterobacteriaceae | 0.5521 |
| Podoviridae |  | P22-like viruses | Temperate | Enterobacteriaceae | -0.3943 |
| Siphoviridae |  | P23-45-like viruses | Virulent | Thermaceae | NA |
| Myoviridae | Peduovirinae | P2-like viruses | Temperate | Burkholderiaceae, Enterobacteraceae | 0.5564 / 0.5813 |
| Siphoviridae |  | P335-like viruses | Virulent and Temperate | Streptococcaceae | 0.8013 |
| Podoviridae | Picovirinae | P68-like viruses | Virulent | Staphylococcaceae, Streptococcaceae | 0.8323 / 0.9489 |
| Myoviridae |  | PAKP1-like viruses | Unknown | Vibrionaceae | -0.3893 |
| Myoviridae |  | PB1-like viruses | Virulent | Burkholderiaceae | NA |
| Podoviridae | Picovirinae | Phi29-like viruses | Virulent | Bacillaceae, Streptococcaceae | -0.2447 / -0.3306 |
| Siphoviridae |  | PhiC31-like viruses | Temperate | Streptomycetaceae | NA |
| Myoviridae |  | phiCD119-like viruses | Temperate | Clostridaceae | 0.9812 |
| Siphoviridae |  | phiE125-like viruses | Temperate | Burkholderiaceae | 0.661 |
| Siphoviridae |  | phiETA-like viruses | Temperate | Staphylococcaceae | 0.6388 |
| Siphoviridae |  | phiFL-like viruses | Temperate | Enterococcaceae | 0.9296 |
| Podoviridae | Autogrphivirinae | phiKMV-like viruses | Virulent | Pseudomonadaceae | NA |
| Myoviridae |  | phiKZ-like viruses | Virulent | Pseudomonadaceae | -0.5719 |
| Siphoviridae |  | phiLJ1-like viruses | Virulent | Lactobacillaceae | -0.327 |
| Myoviridae |  | phiPLPE-like viruses | Virulent and Temperate | Pateurellaceae | 0.9791 |
| Siphoviridae |  | PsiM1-like viruses | Unknown | Methanobacteriaceae | NA |
| Myoviridae | Tevenvirinae | RB49-like viruses | Virulent | Enterobacteriaceae | 0.6387 |
| Myoviridae |  | rV5-like viruses | Virulent | Enterobacteriaceae | 0.306 |
| Myoviridae | Tevenvirinae | Schizot4-like viruses | Virulent | Vibrionaceae | -0.3893 |
| Myoviridae |  | Secunda5-like viruses | Virulent | Aeromonadaceae | 0.3895 |
| Siphoviridae |  | Sfi11-like viruses | Virulent and Temperate | Streptococcaceae | 0.8539 |
| Siphoviridae |  | Sfi21-like viruses | Virulent and Temperate | Streptococcaceae | -0.7135 |
| Myoviridae |  | SfV-like viruses | Temperate | Enterobacteriaceae | NA |
| Myoviridae | Tevenvirinae | SP18-like viruses | Virulent | Enterobacteriaceae | -0.411 |
| Podoviridae | Autogrphivirinae | SP6-like viruses | Virulent | Enterobacteriaceae | -0.2322 |
| Siphoviridae |  | SPbeta-like viruses | Temperate | Bacillaceae | -0.6233 |
| Myoviridae | Spounavirinae | SPO1-like viruses | Virulent | Bacillaceae | 0.2549 |
| Myoviridae | Tevenvirinae | T4-like viruses | Virulent | Multiple | NA |
| Siphoviridae |  | T5-like viruses | Virulent | Enterobacteriaceae | 0.3428 |
| Podoviridae | Autogrphivirinae | T7-like viruses | Virulent | Enterobacteriaceae | -0.0917 |
| Siphoviridae |  | TM4-like viruses | Virulent | Mycobacteriaceae | -0.3544 |
| Siphoviridae |  | TP21-like viruses | Temperate | Bacillaceae | 0.7467 |
| Myoviridae | Spounavirinae | Twort-like viruses | Virulent | Staphylococcaceae | -0.0661 |
| Myoviridae |  | VHML-like viruses | Temperate | Vibrionaceae | 0.3769 |
| Myoviridae |  | Vi1-like viruses | Virulent | Comamonadeceae | NA |
| Siphoviridae |  | Wbeta-like viruses | Temperate | Bacillaceae | 0.5459 |
| Siphoviridae |  | Xp10-like viruses | Virulent | Xanthomonadaceae | NA |
| Siphoviridae |  | Yua-like viruses | Temperate | Alphaproteobacteria | NA |
