# Supplementary material for: Integrative Longitudinal Analysis of Metabolic Phenotype and Microbiota Changes During the Development of Obesity
Source: Front Cell Infect Microbiol. 2021 Aug 3;11:671926. doi: 10.3389/fcimb.2021.671926 (PMC8370388; doi:10.3389/fcimb.2021.671926)
Supplement: Supplementary file 10 [file Table_9.docx]

**Supplemental Table 9: Person’s Correlation Coefficient Results for Bacterial Family and Bacteriophage Genera**

|  | Acholeplasmataceae | Acidaminococcaceae | Actinomycetaceae | Aerococcaceae | Bacillaceae | Bacteroidaceae | Bifidobacteriaceae | Brachyspiraceae | Burkholderiaceae | Campylobacteraceae | Carnobacteriaceae | Chlorobiaceae | Clostridiaceae |
| --- | --- | --- | --- | --- | --- | --- | --- | --- | --- | --- | --- | --- | --- |
| 0305phi8-36-like viruses | 0.9732 | 0.5478 | 0.4922 | 0.7112 | -0.2831 | 0.6493 | 0.2557 | 0.6443 | 0.4751 | 0.6229 | 0.9016 | 0.3420 | 0.5510 |
| 1706-like viruses | 0.7367 | 0.9759 | 0.9768 | 0.9732 | -0.8684 | 0.8339 | 0.0228 | 0.9594 | 0.6779 | 0.8528 | 0.6964 | 0.6319 | 0.9815 |
| 3a-like viruses | 0.9750 | 0.5723 | 0.5206 | 0.7297 | -0.2639 | 0.5753 | -0.0154 | 0.6326 | 0.3928 | 0.5574 | 0.8542 | 0.2133 | 0.5842 |
| 77-like viruses | 0.5948 | 0.9705 | 0.9850 | 0.9305 | -0.9163 | 0.7952 | 0.0050 | 0.9349 | 0.6349 | 0.8264 | 0.5527 | 0.6460 | 0.9800 |
| 936-like viruses | -0.6653 | -0.5225 | -0.5982 | -0.6269 | 0.4578 | -0.5172 | -0.2461 | -0.6445 | -0.3434 | -0.5057 | -0.8312 | -0.3337 | -0.5406 |
| Bcep22-like viruses | 0.0034 | 0.7126 | 0.7635 | 0.5563 | -0.8684 | 0.5176 | 0.0326 | 0.6306 | 0.4717 | 0.5638 | 0.0474 | 0.6034 | 0.7126 |
| Bcep781-like viruses | 0.9856 | 0.5964 | 0.5524 | 0.7528 | -0.3003 | 0.6020 | 0.0171 | 0.6635 | 0.4188 | 0.5833 | 0.8880 | 0.2454 | 0.6077 |
| BcepMu-like viruses | -0.0979 | -0.1144 | -0.1791 | -0.1376 | -0.0472 | 0.2626 | 0.9396 | -0.0388 | 0.4214 | 0.2231 | 0.0483 | 0.5072 | -0.1724 |
| Bpp-1-like viruses | 0.8600 | 0.9318 | 0.8907 | 0.9639 | -0.7633 | 0.8692 | 0.0955 | 0.9273 | 0.7532 | 0.8698 | 0.7964 | 0.6326 | 0.9237 |
| c2-like viruses | 0.8702 | 0.3340 | 0.2425 | 0.5099 | -0.0170 | 0.4455 | 0.1184 | 0.4075 | 0.2814 | 0.4197 | 0.6943 | 0.1262 | 0.3405 |
| Che8-like viruses | 0.6745 | 0.4804 | 0.4048 | 0.5573 | -0.4187 | 0.7711 | 0.8258 | 0.5872 | 0.7427 | 0.7327 | 0.7311 | 0.7187 | 0.4428 |
| D3112-like viruses | -0.2635 | -0.4029 | -0.3119 | -0.3917 | 0.3414 | -0.4860 | -0.3802 | -0.3895 | -0.2845 | -0.5191 | 0.0066 | -0.4675 | -0.4146 |
| D3-like viruses | -0.0979 | -0.1144 | -0.1791 | -0.1376 | -0.0472 | 0.2626 | 0.9396 | -0.0388 | 0.4214 | 0.2231 | 0.0483 | 0.5072 | -0.1724 |
| F116-like viruses | -0.2340 | -0.3958 | -0.3173 | -0.3240 | 0.4126 | -0.4801 | -0.2156 | -0.3376 | -0.7522 | -0.4416 | -0.3387 | -0.5241 | -0.3216 |
| FelixO1-like viruses | 0.9909 | 0.6037 | 0.5539 | 0.7575 | -0.3154 | 0.6317 | 0.0734 | 0.6735 | 0.4638 | 0.6098 | 0.9036 | 0.2880 | 0.6099 |
| HAP1-like viruses | -0.5577 | -0.7259 | -0.8143 | -0.7634 | 0.6561 | -0.5168 | 0.0988 | -0.7635 | -0.2185 | -0.5544 | -0.5569 | -0.3090 | -0.7758 |
| Hp1-like viruses | -0.1075 | 0.6315 | 0.6849 | 0.4623 | -0.8051 | 0.4237 | -0.0126 | 0.5383 | 0.3795 | 0.4753 | -0.0803 | 0.5355 | 0.6350 |
| IEBH-like viruses | 0.7854 | 0.9428 | 0.9434 | 0.9632 | -0.7951 | 0.7862 | -0.0632 | 0.9303 | 0.6162 | 0.8029 | 0.7279 | 0.5403 | 0.9534 |
| Jersey-like viruses | -0.1075 | 0.6315 | 0.6849 | 0.4623 | -0.8051 | 0.4237 | -0.0126 | 0.5383 | 0.3795 | 0.4753 | -0.0803 | 0.5355 | 0.6350 |
| JS98-like viruses | -0.2635 | -0.4029 | -0.3119 | -0.3917 | 0.3414 | -0.4860 | -0.3802 | -0.3895 | -0.2845 | -0.5191 | 0.0066 | -0.4675 | -0.4146 |
| K-like viruses | -0.2839 | -0.3225 | -0.4349 | -0.3669 | 0.4083 | -0.3322 | -0.3541 | -0.4417 | -0.2014 | -0.3301 | -0.5287 | -0.3096 | -0.3398 |
| KP34-like viruses | -0.1929 | 0.5704 | 0.6088 | 0.3874 | -0.7810 | 0.4239 | 0.1377 | 0.4812 | 0.3981 | 0.4744 | -0.1687 | 0.5965 | 0.5670 |
| L5-like viruses | 0.6540 | 0.7179 | 0.7697 | 0.7507 | -0.6101 | 0.5384 | -0.2078 | 0.7253 | 0.4726 | 0.5350 | 0.7569 | 0.3088 | 0.7217 |
| Lambda-like viruses | 0.9886 | 0.6878 | 0.6216 | 0.8164 | -0.4127 | 0.7202 | 0.1209 | 0.7385 | 0.5654 | 0.7019 | 0.8792 | 0.3992 | 0.6873 |
| LUZ24-like viruses | -0.2291 | -0.3396 | -0.3540 | -0.3191 | 0.1983 | -0.0366 | 0.7520 | -0.2347 | -0.0479 | -0.0509 | -0.1538 | 0.1663 | -0.3502 |
| Omega-like viruses | -0.1179 | 0.6244 | 0.6743 | 0.4526 | -0.7961 | 0.4149 | -0.0249 | 0.5265 | 0.3750 | 0.4669 | -0.0990 | 0.5282 | 0.6274 |
| P100-like viruses | -0.2305 | -0.2649 | -0.3816 | -0.3087 | 0.1471 | 0.1002 | 0.7536 | -0.2473 | 0.3178 | 0.0624 | -0.2030 | 0.3508 | -0.3300 |
| P1-like viruses | 0.9917 | 0.5997 | 0.5443 | 0.7531 | -0.3220 | 0.6585 | 0.1500 | 0.6762 | 0.5019 | 0.6333 | 0.9155 | 0.3316 | 0.6013 |
| P22-like viruses | -0.2928 | -0.4215 | -0.3933 | -0.4292 | 0.2502 | -0.2113 | 0.3791 | -0.3542 | 0.0748 | -0.2683 | 0.0414 | -0.0145 | -0.4743 |
| P2-like viruses | 0.9755 | 0.7874 | 0.7598 | 0.8978 | -0.5413 | 0.7396 | 0.0263 | 0.8315 | 0.5564 | 0.7320 | 0.9040 | 0.4129 | 0.7966 |
| P335-like viruses | -0.6815 | -0.9340 | -0.9062 | -0.9140 | 0.8039 | -0.7772 | 0.0868 | -0.8804 | -0.6640 | -0.7965 | -0.5838 | -0.5766 | -0.9329 |
| P68-like viruses | -0.5801 | -0.9550 | -0.9717 | -0.9132 | 0.9740 | -0.8992 | -0.3473 | -0.9628 | -0.7800 | -0.9162 | -0.6379 | -0.8315 | -0.9481 |
| PAKP1-like viruses | -0.2839 | -0.3225 | -0.4349 | -0.3669 | 0.4083 | -0.3322 | -0.3541 | -0.4417 | -0.2014 | -0.3301 | -0.5287 | -0.3096 | -0.3398 |
| Phi29-like viruses | 0.9588 | 0.5430 | 0.4603 | 0.6914 | -0.2447 | 0.6159 | 0.1134 | 0.6001 | 0.4902 | 0.5882 | 0.8371 | 0.2924 | 0.5385 |
| phiCD119-like viruses | 0.5598 | 0.9790 | 0.9872 | 0.9237 | -0.9538 | 0.8324 | 0.0953 | 0.9421 | 0.6957 | 0.8620 | 0.5329 | 0.7208 | 0.9812 |
| phiE125-like viruses | 0.7640 | 0.9740 | 0.9775 | 0.9831 | -0.8588 | 0.8399 | 0.0453 | 0.9686 | 0.6611 | 0.8591 | 0.7204 | 0.6271 | 0.9834 |
| phiETA-like viruses | -0.5234 | -0.6844 | -0.6523 | -0.6517 | 0.6803 | -0.7310 | -0.3029 | -0.6774 | -0.8743 | -0.6992 | -0.6805 | -0.6896 | -0.6289 |
| phiFL-like viruses | -0.5870 | -0.9618 | -0.9845 | -0.9291 | 0.9227 | -0.8064 | -0.0920 | -0.9450 | -0.6027 | -0.8421 | -0.5436 | -0.6695 | -0.9784 |
| phiKZ-like viruses | 0.9827 | 0.5758 | 0.5308 | 0.7358 | -0.2771 | 0.5870 | 0.0171 | 0.6450 | 0.4075 | 0.5668 | 0.8864 | 0.2298 | 0.5867 |
| phiLJ1-like viruses | 0.0901 | 0.7446 | 0.8052 | 0.6077 | -0.8636 | 0.5138 | -0.0743 | 0.6656 | 0.4597 | 0.5570 | 0.1436 | 0.5453 | 0.7483 |
| phiPLPE-like viruses | 0.8642 | 0.9344 | 0.8968 | 0.9726 | -0.8149 | 0.9618 | 0.3809 | 0.9707 | 0.8252 | 0.9588 | 0.8455 | 0.7788 | 0.9229 |
| RB49-like viruses | 0.5948 | 0.9705 | 0.9850 | 0.9305 | -0.9163 | 0.7952 | 0.0050 | 0.9349 | 0.6349 | 0.8264 | 0.5527 | 0.6460 | 0.9800 |
| rV5-like viruses | -0.1655 | 0.5778 | 0.6292 | 0.4056 | -0.7465 | 0.3531 | -0.0729 | 0.4748 | 0.2932 | 0.4107 | -0.1741 | 0.4666 | 0.5873 |
| Schizot4-like viruses | -0.2839 | -0.3225 | -0.4349 | -0.3669 | 0.4083 | -0.3322 | -0.3541 | -0.4417 | -0.2014 | -0.3301 | -0.5287 | -0.3096 | -0.3398 |
| Secunda5-like viruses | -0.1198 | 0.6293 | 0.6766 | 0.4549 | -0.8231 | 0.4588 | 0.0886 | 0.5427 | 0.4311 | 0.5069 | -0.0763 | 0.5988 | 0.6266 |
| Sfi11-like viruses | -0.6636 | -0.9078 | -0.9381 | -0.9120 | 0.8930 | -0.8677 | -0.3671 | -0.9534 | -0.6847 | -0.8830 | -0.7216 | -0.7537 | -0.9140 |
| Sfi21-like viruses | 0.8695 | 0.9078 | 0.8830 | 0.9560 | -0.7108 | 0.7925 | -0.0425 | 0.9038 | 0.6275 | 0.8003 | 0.7803 | 0.5126 | 0.9145 |
| SP18-like viruses | 0.2697 | 0.1975 | 0.3075 | 0.2567 | -0.0696 | -0.1259 | -0.7456 | 0.1900 | -0.2471 | -0.1110 | 0.3172 | -0.3819 | 0.2440 |
| SP6-like viruses | -0.3579 | -0.5477 | -0.5104 | -0.5059 | 0.4029 | -0.3193 | 0.5089 | -0.4315 | -0.3381 | -0.3328 | -0.2302 | -0.1392 | -0.5412 |
| SPbeta-like viruses | 0.9707 | 0.8409 | 0.8039 | 0.9345 | -0.6233 | 0.8268 | 0.1500 | 0.8846 | 0.6597 | 0.8185 | 0.9102 | 0.5365 | 0.8421 |
| SPO1-like viruses | 0.5377 | 0.0354 | -0.0844 | 0.1529 | 0.2549 | 0.1131 | -0.1843 | 0.0290 | 0.1712 | 0.0721 | 0.3780 | -0.1313 | 0.0129 |
| T4-like viruses | 0.6435 | 0.9895 | 0.9969 | 0.9597 | -0.9377 | 0.8605 | 0.1291 | 0.9719 | 0.6934 | 0.8874 | 0.6111 | 0.7179 | 0.9956 |
| T5-like viruses | -0.0979 | -0.1144 | -0.1791 | -0.1376 | -0.0472 | 0.2626 | 0.9396 | -0.0388 | 0.4214 | 0.2231 | 0.0483 | 0.5072 | -0.1724 |
| T7-like viruses | -0.5007 | -0.6760 | -0.7489 | -0.6836 | 0.5686 | -0.3759 | 0.4742 | -0.6440 | -0.2427 | -0.3988 | -0.5216 | -0.1372 | -0.7039 |
| TM4-like viruses | -0.2839 | -0.3225 | -0.4349 | -0.3669 | 0.4083 | -0.3322 | -0.3541 | -0.4417 | -0.2014 | -0.3301 | -0.5287 | -0.3096 | -0.3398 |
| TP21-like viruses | -0.6095 | -0.8148 | -0.8573 | -0.8088 | 0.7467 | -0.6356 | 0.1328 | -0.8011 | -0.5813 | -0.6394 | -0.7014 | -0.4530 | -0.8124 |
| Twort-like viruses | 0.9963 | 0.6675 | 0.6212 | 0.8086 | -0.3905 | 0.6791 | 0.0756 | 0.7301 | 0.5070 | 0.6610 | 0.9114 | 0.3408 | 0.6735 |
| VHML-like viruses | -0.0979 | -0.1144 | -0.1791 | -0.1376 | -0.0472 | 0.2626 | 0.9396 | -0.0388 | 0.4214 | 0.2231 | 0.0483 | 0.5072 | -0.1724 |
| Wbeta-like viruses | -0.5084 | -0.6992 | -0.7440 | -0.7054 | 0.5459 | -0.3759 | 0.5429 | -0.6427 | -0.1737 | -0.4145 | -0.3983 | -0.1095 | -0.7393 |

**Supplemental Table 9: Person’s Correlation Coefficient Results for Bacterial Family and Bacteriophage Genera Continued**

|  | Clostridiales Family XI | Coriobacteriaceae | Cytophagaceae | Desulfovibrionaceae | Enterobacteriaceae | Enterococcaceae | Erysipelotrichaceae | Eubacteriaceae | Fibrobacteraceae | Flavobacteriaceae | Fusobacteriaceae | Geobacteraceae | Halanaerobiaceae |
| --- | --- | --- | --- | --- | --- | --- | --- | --- | --- | --- | --- | --- | --- |
| 0305phi8-36-like viruses | 0.6543 | 0.2071 | 0.4923 | 0.6340 | 0.5692 | -0.5532 | 0.7928 | 0.5629 | 0.7774 | 0.6705 | 0.5781 | 0.6287 | 0.7803 |
| 1706-like viruses | 0.9560 | 0.0663 | 0.6874 | 0.9630 | 0.6572 | -0.9396 | 0.8875 | 0.9810 | 0.9194 | 0.6593 | 0.9663 | 0.8079 | 0.9545 |
| 3a-like viruses | 0.6487 | 0.1668 | 0.3617 | 0.6261 | 0.4861 | -0.5507 | 0.7447 | 0.5868 | 0.7755 | 0.5302 | 0.5943 | 0.5533 | 0.8020 |
| 77-like viruses | 0.9313 | 0.1073 | 0.6631 | 0.9343 | 0.6387 | -0.9327 | 0.8316 | 0.9763 | 0.8530 | 0.5947 | 0.9557 | 0.7637 | 0.8830 |
| 936-like viruses | -0.5859 | -0.3158 | -0.4950 | -0.6482 | -0.2235 | 0.4772 | -0.6970 | -0.6289 | -0.5520 | -0.5237 | -0.5099 | -0.4819 | -0.6419 |
| Bcep22-like viruses | 0.6143 | -0.0202 | 0.5221 | 0.6364 | 0.3954 | -0.6864 | 0.4331 | 0.7066 | 0.4392 | 0.3254 | 0.6804 | 0.4997 | 0.4544 |
| Bcep781-like viruses | 0.6737 | 0.1709 | 0.4002 | 0.6586 | 0.4900 | -0.5733 | 0.7724 | 0.6172 | 0.7916 | 0.5610 | 0.6162 | 0.5794 | 0.8234 |
| BcepMu-like viruses | -0.0409 | -0.2200 | 0.4828 | -0.0357 | 0.3428 | -0.0060 | 0.0452 | -0.1796 | -0.0107 | 0.5013 | -0.0805 | 0.3021 | -0.1269 |
| Bpp-1-like viruses | 0.9363 | -0.0583 | 0.7141 | 0.9357 | 0.7215 | -0.9164 | 0.8991 | 0.9097 | 0.9696 | 0.7437 | 0.9361 | 0.8590 | 0.9842 |
| c2-like viruses | 0.4451 | 0.2079 | 0.2462 | 0.3879 | 0.4897 | -0.3506 | 0.5880 | 0.3231 | 0.6157 | 0.4711 | 0.3766 | 0.4281 | 0.5902 |
| Che8-like viruses | 0.5894 | -0.0138 | 0.8025 | 0.5842 | 0.7344 | -0.5596 | 0.7143 | 0.4460 | 0.6701 | 0.9098 | 0.5192 | 0.7801 | 0.5990 |
| D3112-like viruses | -0.4584 | -0.4674 | -0.3521 | -0.3296 | -0.7763 | 0.4727 | -0.4745 | -0.3434 | -0.4553 | -0.4269 | -0.4489 | -0.4416 | -0.3184 |
| D3-like viruses | -0.0409 | -0.2200 | 0.4828 | -0.0357 | 0.3428 | -0.0060 | 0.0452 | -0.1796 | -0.0107 | 0.5013 | -0.0805 | 0.3021 | -0.1269 |
| F116-like viruses | -0.3385 | 0.8875 | -0.5608 | -0.3980 | -0.3417 | 0.4336 | -0.2195 | -0.2821 | -0.4141 | -0.5022 | -0.3926 | -0.5591 | -0.3949 |
| FelixO1-like viruses | 0.6836 | 0.1354 | 0.4431 | 0.6704 | 0.5198 | -0.5880 | 0.7841 | 0.6179 | 0.8057 | 0.6040 | 0.6253 | 0.6134 | 0.8309 |
| HAP1-like viruses | -0.7323 | -0.5660 | -0.3729 | -0.7427 | -0.3201 | 0.6492 | -0.7388 | -0.8299 | -0.6194 | -0.3436 | -0.7042 | -0.4458 | -0.7037 |
| Hp1-like viruses | 0.5260 | 0.0065 | 0.4310 | 0.5401 | 0.3360 | -0.6057 | 0.3353 | 0.6241 | 0.3403 | 0.2253 | 0.5987 | 0.4037 | 0.3498 |
| IEBH-like viruses | 0.9282 | 0.0858 | 0.6135 | 0.9338 | 0.6051 | -0.8970 | 0.8727 | 0.9552 | 0.9106 | 0.6079 | 0.9336 | 0.7583 | 0.9571 |
| Jersey-like viruses | 0.5260 | 0.0065 | 0.4310 | 0.5401 | 0.3360 | -0.6057 | 0.3353 | 0.6241 | 0.3403 | 0.2253 | 0.5987 | 0.4037 | 0.3498 |
| JS98-like viruses | -0.4584 | -0.4674 | -0.3521 | -0.3296 | -0.7763 | 0.4727 | -0.4745 | -0.3434 | -0.4553 | -0.4269 | -0.4489 | -0.4416 | -0.3184 |
| K-like viruses | -0.3677 | -0.3523 | -0.4144 | -0.4430 | -0.0576 | 0.2875 | -0.4609 | -0.4407 | -0.2591 | -0.3693 | -0.3006 | -0.2968 | -0.3396 |
| KP34-like viruses | 0.4756 | 0.0133 | 0.4641 | 0.4773 | 0.3934 | -0.5698 | 0.2941 | 0.5469 | 0.2881 | 0.2599 | 0.5458 | 0.4070 | 0.2652 |
| L5-like viruses | 0.6829 | -0.1402 | 0.4497 | 0.7598 | 0.1970 | -0.6386 | 0.6342 | 0.7671 | 0.6661 | 0.4087 | 0.6856 | 0.5333 | 0.7843 |
| Lambda-like viruses | 0.7577 | 0.0825 | 0.5300 | 0.7351 | 0.6346 | -0.6837 | 0.8305 | 0.6795 | 0.8752 | 0.6770 | 0.7121 | 0.7055 | 0.8805 |
| LUZ24-like viruses | -0.2371 | 0.3158 | 0.1219 | -0.2673 | 0.1198 | 0.2492 | -0.0868 | -0.3337 | -0.2533 | 0.1736 | -0.3059 | -0.0461 | -0.3507 |
| Omega-like viruses | 0.5167 | -0.0056 | 0.4195 | 0.5283 | 0.3362 | -0.5996 | 0.3215 | 0.6129 | 0.3336 | 0.2140 | 0.5922 | 0.3960 | 0.3404 |
| P100-like viruses | -0.2142 | -0.3826 | 0.2772 | -0.2448 | 0.3089 | 0.1303 | -0.1740 | -0.3848 | -0.1332 | 0.3166 | -0.2213 | 0.1556 | -0.2853 |
| P1-like viruses | 0.6863 | 0.1188 | 0.4861 | 0.6734 | 0.5521 | -0.5936 | 0.7947 | 0.6088 | 0.8119 | 0.6499 | 0.6243 | 0.6432 | 0.8280 |
| P22-like viruses | -0.4133 | -0.5537 | 0.0639 | -0.3019 | -0.3943 | 0.3905 | -0.3629 | -0.4201 | -0.3883 | 0.0151 | -0.4347 | -0.1449 | -0.3601 |
| P2-like viruses | 0.8344 | 0.1338 | 0.5483 | 0.8313 | 0.5814 | -0.7562 | 0.8799 | 0.8064 | 0.9032 | 0.6490 | 0.7966 | 0.7150 | 0.9427 |
| P335-like viruses | -0.8948 | 0.0678 | -0.6081 | -0.8876 | -0.6538 | 0.9035 | -0.7897 | -0.9057 | -0.8851 | -0.5829 | -0.9276 | -0.7630 | -0.9042 |
| P68-like viruses | -0.9434 | -0.0732 | -0.8604 | -0.9659 | -0.7140 | 0.9476 | -0.8851 | -0.9615 | -0.8556 | -0.7853 | -0.9452 | -0.8767 | -0.8667 |
| PAKP1-like viruses | -0.3677 | -0.3523 | -0.4144 | -0.4430 | -0.0576 | 0.2875 | -0.4609 | -0.4407 | -0.2591 | -0.3693 | -0.3006 | -0.2968 | -0.3396 |
| Phi29-like viruses | 0.6253 | 0.0343 | 0.4304 | 0.5977 | 0.5638 | -0.5481 | 0.7209 | 0.5255 | 0.7795 | 0.6092 | 0.5739 | 0.6096 | 0.7759 |
| phiCD119-like viruses | 0.9389 | 0.0626 | 0.7263 | 0.9429 | 0.6845 | -0.9537 | 0.8333 | 0.9737 | 0.8555 | 0.6466 | 0.9659 | 0.8061 | 0.8720 |
| phiE125-like viruses | 0.9647 | 0.1312 | 0.6875 | 0.9681 | 0.6671 | -0.9378 | 0.9129 | 0.9871 | 0.9280 | 0.6708 | 0.9665 | 0.8084 | 0.9628 |
| phiETA-like viruses | -0.6530 | 0.6151 | -0.7818 | -0.7302 | -0.4737 | 0.6949 | -0.5780 | -0.6305 | -0.6846 | -0.7249 | -0.6737 | -0.7798 | -0.7064 |
| phiFL-like viruses | -0.9400 | -0.2548 | -0.6787 | -0.9335 | -0.6715 | 0.9296 | -0.8664 | -0.9816 | -0.8443 | -0.6174 | -0.9507 | -0.7626 | -0.8656 |
| phiKZ-like viruses | 0.6552 | 0.1656 | 0.3870 | 0.6405 | 0.4764 | -0.5534 | 0.7582 | 0.5966 | 0.7776 | 0.5520 | 0.5962 | 0.5655 | 0.8094 |
| phiLJ1-like viruses | 0.6429 | -0.0413 | 0.4956 | 0.6775 | 0.3403 | -0.6995 | 0.4636 | 0.7517 | 0.4787 | 0.3061 | 0.7068 | 0.4958 | 0.5210 |
| phiPLPE-like viruses | 0.9724 | 0.0613 | 0.8573 | 0.9699 | 0.8265 | -0.9439 | 0.9824 | 0.9216 | 0.9876 | 0.8941 | 0.9468 | 0.9451 | 0.9787 |
| RB49-like viruses | 0.9313 | 0.1073 | 0.6631 | 0.9343 | 0.6387 | -0.9327 | 0.8316 | 0.9763 | 0.8530 | 0.5947 | 0.9557 | 0.7637 | 0.8830 |
| rV5-like viruses | 0.4699 | 0.0612 | 0.3437 | 0.4703 | 0.3060 | -0.5509 | 0.2750 | 0.5693 | 0.2816 | 0.1418 | 0.5465 | 0.3281 | 0.2848 |
| Schizot4-like viruses | -0.3677 | -0.3523 | -0.4144 | -0.4430 | -0.0576 | 0.2875 | -0.4609 | -0.4407 | -0.2591 | -0.3693 | -0.3006 | -0.2968 | -0.3396 |
| Secunda5-like viruses | 0.5300 | -0.0172 | 0.4900 | 0.5449 | 0.3784 | -0.6160 | 0.3455 | 0.6147 | 0.3446 | 0.2830 | 0.5996 | 0.4427 | 0.3417 |
| Sfi11-like viruses | -0.9283 | -0.2578 | -0.8108 | -0.9466 | -0.6770 | 0.8935 | -0.9274 | -0.9458 | -0.8471 | -0.7752 | -0.9019 | -0.8302 | -0.8693 |
| Sfi21-like viruses | 0.9118 | 0.0595 | 0.6002 | 0.9063 | 0.6420 | -0.8726 | 0.8813 | 0.9074 | 0.9378 | 0.6383 | 0.9084 | 0.7695 | 0.9715 |
| SP18-like viruses | 0.1445 | 0.1199 | -0.2593 | 0.2108 | -0.4110 | -0.0632 | 0.1109 | 0.3079 | 0.1068 | -0.2923 | 0.1502 | -0.1544 | 0.2787 |
| SP6-like viruses | -0.4561 | 0.3781 | -0.1527 | -0.4584 | -0.2322 | 0.4974 | -0.2915 | -0.4924 | -0.4904 | -0.1135 | -0.5307 | -0.3350 | -0.5304 |
| SPbeta-like viruses | 0.8892 | 0.0999 | 0.6584 | 0.8845 | 0.6792 | -0.8252 | 0.9283 | 0.8464 | 0.9509 | 0.7482 | 0.8533 | 0.8061 | 0.9723 |
| SPO1-like viruses | 0.0751 | -0.3565 | -0.0346 | 0.0454 | 0.1637 | -0.0502 | 0.1197 | -0.0370 | 0.2955 | 0.1432 | 0.0661 | 0.1492 | 0.2774 |
| T4-like viruses | 0.9682 | 0.1369 | 0.7406 | 0.9683 | 0.7111 | -0.9644 | 0.8921 | 0.9941 | 0.8961 | 0.6869 | 0.9805 | 0.8283 | 0.9137 |
| T5-like viruses | -0.0409 | -0.2200 | 0.4828 | -0.0357 | 0.3428 | -0.0060 | 0.0452 | -0.1796 | -0.0107 | 0.5013 | -0.0805 | 0.3021 | -0.1269 |
| T7-like viruses | -0.6121 | -0.0383 | -0.2286 | -0.6640 | -0.0917 | 0.5709 | -0.5219 | -0.7396 | -0.5519 | -0.1614 | -0.6360 | -0.3491 | -0.6794 |
| TM4-like viruses | -0.3677 | -0.3523 | -0.4144 | -0.4430 | -0.0576 | 0.2875 | -0.4609 | -0.4407 | -0.2591 | -0.3693 | -0.3006 | -0.2968 | -0.3396 |
| TP21-like viruses | -0.7651 | 0.1752 | -0.5583 | -0.8341 | -0.3194 | 0.7482 | -0.6810 | -0.8436 | -0.7251 | -0.4865 | -0.7832 | -0.6320 | -0.8216 |
| Twort-like viruses | 0.7393 | 0.1323 | 0.4895 | 0.7276 | 0.5570 | -0.6496 | 0.8244 | 0.6807 | 0.8476 | 0.6347 | 0.6866 | 0.6593 | 0.8739 |
| VHML-like viruses | -0.0409 | -0.2200 | 0.4828 | -0.0357 | 0.3428 | -0.0060 | 0.0452 | -0.1796 | -0.0107 | 0.5013 | -0.0805 | 0.3021 | -0.1269 |
| Wbeta-like viruses | -0.6403 | -0.2000 | -0.1537 | -0.6417 | -0.2146 | 0.6027 | -0.5419 | -0.7450 | -0.5873 | -0.1163 | -0.6714 | -0.3328 | -0.6797 |

**Supplemental Table 9: Person’s Correlation Coefficient Results for Bacterial Family and Bacteriophage Genera Continued**

|  | Helicobacteraceae | Heliobacteriaceae | Lachnospiraceae | Lactobacillaceae | Leuconostocaceae | Listeriaceae | Micrococcaceae | Moraxellaceae | Neisseriaceae | Paenibacillaceae | Pasteurellaceae | Peptococcaceae | Peptostreptococcaceae |
| --- | --- | --- | --- | --- | --- | --- | --- | --- | --- | --- | --- | --- | --- |
| 0305phi8-36-like viruses | 0.3143 | 0.6014 | 0.5337 | -0.4532 | -0.0570 | -0.5163 | -0.4175 | -0.4545 | 0.5115 | -0.5416 | 0.6539 | 0.6446 | 0.8360 |
| 1706-like viruses | 0.8118 | 0.9494 | 0.9835 | -0.6017 | -0.7022 | -0.9472 | -0.9676 | -0.9282 | 0.8473 | -0.9563 | 0.9513 | 0.9909 | 0.9039 |
| 3a-like viruses | 0.4177 | 0.6151 | 0.5727 | -0.4322 | -0.0047 | -0.5401 | -0.5109 | -0.5440 | 0.4313 | -0.5654 | 0.6441 | 0.6889 | 0.8367 |
| 77-like viruses | 0.8132 | 0.9475 | 0.9865 | -0.5488 | -0.7864 | -0.9591 | -0.9641 | -0.9206 | 0.8612 | -0.9545 | 0.9116 | 0.9639 | 0.8345 |
| 936-like viruses | -0.0343 | -0.5628 | -0.5842 | 0.0531 | 0.3168 | 0.4465 | 0.4204 | 0.2533 | -0.6184 | 0.4468 | -0.6107 | -0.5985 | -0.6610 |
| Bcep22-like viruses | 0.6449 | 0.6502 | 0.7267 | -0.3434 | -0.9027 | -0.7179 | -0.7506 | -0.6767 | 0.6863 | -0.6984 | 0.6003 | 0.6210 | 0.3690 |
| Bcep781-like viruses | 0.4109 | 0.6367 | 0.5995 | -0.4303 | -0.0435 | -0.5578 | -0.5303 | -0.5478 | 0.4705 | -0.5834 | 0.6737 | 0.7110 | 0.8546 |
| BcepMu-like viruses | -0.1575 | -0.1522 | -0.2236 | -0.3159 | -0.1970 | 0.1204 | 0.2823 | 0.2084 | 0.1667 | 0.0883 | 0.0072 | -0.2281 | -0.1210 |
| Bpp-1-like viruses | 0.8350 | 0.8910 | 0.9055 | -0.7355 | -0.5731 | -0.8923 | -0.9193 | -0.9264 | 0.7769 | -0.9242 | 0.9504 | 0.9555 | 0.9287 |
| c2-like viruses | 0.2428 | 0.4085 | 0.3085 | -0.3911 | 0.2080 | -0.3359 | -0.2159 | -0.3296 | 0.2496 | -0.3575 | 0.4216 | 0.4429 | 0.6752 |
| Che8-like viruses | 0.2665 | 0.4787 | 0.3980 | -0.6302 | -0.3542 | -0.4504 | -0.2821 | -0.3429 | 0.6314 | -0.4894 | 0.6221 | 0.4591 | 0.6233 |
| D3112-like viruses | -0.3792 | -0.5203 | -0.3666 | 0.4022 | 0.3131 | 0.5375 | 0.1846 | 0.3962 | -0.4849 | 0.4892 | -0.3312 | -0.3661 | -0.4620 |
| D3-like viruses | -0.1575 | -0.1522 | -0.2236 | -0.3159 | -0.1970 | 0.1204 | 0.2823 | 0.2084 | 0.1667 | 0.0883 | 0.0072 | -0.2281 | -0.1210 |
| F116-like viruses | -0.6781 | -0.1787 | -0.2688 | 0.7976 | 0.4189 | 0.2945 | 0.5442 | 0.5607 | -0.2306 | 0.3946 | -0.4724 | -0.3063 | -0.1819 |
| FelixO1-like viruses | 0.4228 | 0.6362 | 0.5974 | -0.4709 | -0.0657 | -0.5618 | -0.5324 | -0.5544 | 0.4877 | -0.5920 | 0.6899 | 0.7102 | 0.8567 |
| HAP1-like viruses | -0.3067 | -0.8110 | -0.8286 | 0.0197 | 0.5002 | 0.7239 | 0.6494 | 0.5218 | -0.7281 | 0.6769 | -0.6743 | -0.7964 | -0.7542 |
| Hp1-like viruses | 0.5925 | 0.5776 | 0.6502 | -0.2703 | -0.8604 | -0.6514 | -0.6737 | -0.6093 | 0.6100 | -0.6235 | 0.5005 | 0.5344 | 0.2738 |
| IEBH-like viruses | 0.7818 | 0.9249 | 0.9585 | -0.5601 | -0.6021 | -0.9105 | -0.9455 | -0.9050 | 0.7856 | -0.9208 | 0.9226 | 0.9810 | 0.9127 |
| Jersey-like viruses | 0.5925 | 0.5776 | 0.6502 | -0.2703 | -0.8604 | -0.6514 | -0.6737 | -0.6093 | 0.6100 | -0.6235 | 0.5005 | 0.5344 | 0.2738 |
| JS98-like viruses | -0.3792 | -0.5203 | -0.3666 | 0.4022 | 0.3131 | 0.5375 | 0.1846 | 0.3962 | -0.4849 | 0.4892 | -0.3312 | -0.3661 | -0.4620 |
| K-like viruses | 0.1950 | -0.3647 | -0.3950 | -0.1638 | 0.3800 | 0.2620 | 0.2049 | 0.0044 | -0.5356 | 0.2426 | -0.3873 | -0.3496 | -0.3642 |
| KP34-like viruses | 0.5454 | 0.5228 | 0.5712 | -0.2928 | -0.8764 | -0.6076 | -0.5743 | -0.5426 | 0.6054 | -0.5766 | 0.4428 | 0.4476 | 0.2054 |
| L5-like viruses | 0.5374 | 0.6429 | 0.7533 | -0.3324 | -0.4362 | -0.6119 | -0.8195 | -0.6610 | 0.5441 | -0.6473 | 0.7466 | 0.7757 | 0.6705 |
| Lambda-like viruses | 0.5539 | 0.7060 | 0.6640 | -0.5954 | -0.1743 | -0.6553 | -0.6163 | -0.6651 | 0.5608 | -0.6880 | 0.7609 | 0.7710 | 0.8949 |
| LUZ24-like viruses | -0.5458 | -0.2474 | -0.3670 | 0.1733 | 0.0620 | 0.2857 | 0.5838 | 0.5244 | 0.0204 | 0.3145 | -0.2708 | -0.3933 | -0.2200 |
| Omega-like viruses | 0.6029 | 0.5687 | 0.6408 | -0.2777 | -0.8527 | -0.6465 | -0.6709 | -0.6130 | 0.5954 | -0.6191 | 0.4903 | 0.5257 | 0.2630 |
| P100-like viruses | -0.0621 | -0.3220 | -0.4063 | -0.3873 | -0.0132 | 0.2422 | 0.3739 | 0.2064 | -0.0901 | 0.2015 | -0.1764 | -0.3892 | -0.2911 |
| P1-like viruses | 0.4138 | 0.6295 | 0.5846 | -0.5006 | -0.0822 | -0.5570 | -0.5142 | -0.5424 | 0.5055 | -0.5901 | 0.6965 | 0.6980 | 0.8544 |
| P22-like viruses | -0.4336 | -0.5475 | -0.4720 | 0.1018 | 0.1155 | 0.5383 | 0.3636 | 0.4855 | -0.2815 | 0.4742 | -0.2713 | -0.4750 | -0.4756 |
| P2-like viruses | 0.5868 | 0.8023 | 0.7930 | -0.5260 | -0.2980 | -0.7441 | -0.7402 | -0.7298 | 0.6527 | -0.7675 | 0.8373 | 0.8743 | 0.9414 |
| P335-like viruses | -0.9150 | -0.8839 | -0.9205 | 0.6817 | 0.6408 | 0.9140 | 0.9712 | 0.9775 | -0.7322 | 0.9325 | -0.8917 | -0.9394 | -0.8325 |
| P68-like viruses | -0.7033 | -0.9217 | -0.9495 | 0.5955 | 0.8932 | 0.9269 | 0.8855 | 0.8239 | -0.9700 | 0.9309 | -0.9476 | -0.9163 | -0.8182 |
| PAKP1-like viruses | 0.1950 | -0.3647 | -0.3950 | -0.1638 | 0.3800 | 0.2620 | 0.2049 | 0.0044 | -0.5356 | 0.2426 | -0.3873 | -0.3496 | -0.3642 |
| Phi29-like viruses | 0.4588 | 0.5620 | 0.5081 | -0.5606 | -0.0090 | -0.5099 | -0.4752 | -0.5474 | 0.4107 | -0.5500 | 0.6351 | 0.6348 | 0.7967 |
| phiCD119-like viruses | 0.8324 | 0.9448 | 0.9817 | -0.6007 | -0.8469 | -0.9682 | -0.9634 | -0.9277 | 0.8929 | -0.9669 | 0.9245 | 0.9522 | 0.8160 |
| phiE125-like viruses | 0.7747 | 0.9634 | 0.9876 | -0.5743 | -0.6857 | -0.9495 | -0.9455 | -0.9059 | 0.8635 | -0.9539 | 0.9527 | 0.9967 | 0.9287 |
| phiETA-like viruses | -0.7037 | -0.5107 | -0.6026 | 0.7760 | 0.6211 | 0.5673 | 0.7665 | 0.7051 | -0.5772 | 0.6507 | -0.7740 | -0.6309 | -0.5245 |
| phiFL-like viruses | -0.7296 | -0.9727 | -0.9890 | 0.4842 | 0.8009 | 0.9671 | 0.9043 | 0.8623 | -0.9135 | 0.9476 | -0.9004 | -0.9564 | -0.8537 |
| phiKZ-like viruses | 0.3930 | 0.6163 | 0.5782 | -0.4213 | -0.0204 | -0.5361 | -0.5101 | -0.5284 | 0.4506 | -0.5628 | 0.6567 | 0.6922 | 0.8413 |
| phiLJ1-like viruses | 0.6631 | 0.6752 | 0.7704 | -0.3270 | -0.8622 | -0.7307 | -0.8123 | -0.7113 | 0.6745 | -0.7174 | 0.6400 | 0.6772 | 0.4215 |
| phiPLPE-like viruses | 0.7201 | 0.9202 | 0.9029 | -0.7297 | -0.6567 | -0.9038 | -0.8341 | -0.8411 | 0.9034 | -0.9269 | 0.9791 | 0.9401 | 0.9592 |
| RB49-like viruses | 0.8132 | 0.9475 | 0.9865 | -0.5488 | -0.7864 | -0.9591 | -0.9641 | -0.9206 | 0.8612 | -0.9545 | 0.9116 | 0.9639 | 0.8345 |
| rV5-like viruses | 0.5631 | 0.5400 | 0.6026 | -0.2144 | -0.8059 | -0.6153 | -0.6185 | -0.5715 | 0.5500 | -0.5781 | 0.4275 | 0.4838 | 0.2256 |
| Schizot4-like viruses | 0.1950 | -0.3647 | -0.3950 | -0.1638 | 0.3800 | 0.2620 | 0.2049 | 0.0044 | -0.5356 | 0.2426 | -0.3873 | -0.3496 | -0.3642 |
| Secunda5-like viruses | 0.5849 | 0.5704 | 0.6365 | -0.3087 | -0.8954 | -0.6488 | -0.6540 | -0.5966 | 0.6377 | -0.6239 | 0.5093 | 0.5183 | 0.2651 |
| Sfi11-like viruses | -0.5471 | -0.9210 | -0.9262 | 0.4663 | 0.7837 | 0.8839 | 0.7907 | 0.7152 | -0.9666 | 0.8759 | -0.9171 | -0.9037 | -0.8671 |
| Sfi21-like viruses | 0.7803 | 0.8941 | 0.9086 | -0.6153 | -0.4956 | -0.8761 | -0.9005 | -0.8939 | 0.7337 | -0.8952 | 0.9085 | 0.9589 | 0.9369 |
| SP18-like viruses | 0.0452 | 0.1971 | 0.3160 | 0.3246 | 0.0943 | -0.1239 | -0.3628 | -0.1662 | -0.0135 | -0.1175 | 0.1630 | 0.3245 | 0.2266 |
| SP6-like viruses | -0.8051 | -0.4424 | -0.5269 | 0.5032 | 0.2705 | 0.5142 | 0.7413 | 0.7593 | -0.1995 | 0.5521 | -0.4821 | -0.5609 | -0.3994 |
| SPbeta-like viruses | 0.6417 | 0.8458 | 0.8304 | -0.6165 | -0.4003 | -0.8006 | -0.7745 | -0.7766 | 0.7343 | -0.8267 | 0.8949 | 0.9043 | 0.9655 |
| SPO1-like viruses | 0.2974 | -0.0089 | -0.0365 | -0.4250 | 0.4197 | 0.0003 | -0.1064 | -0.2356 | -0.2115 | -0.0660 | 0.1181 | 0.1103 | 0.2428 |
| T4-like viruses | 0.7942 | 0.9748 | 0.9975 | -0.5874 | -0.8096 | -0.9794 | -0.9468 | -0.9122 | 0.9196 | -0.9757 | 0.9482 | 0.9789 | 0.8784 |
| T5-like viruses | -0.1575 | -0.1522 | -0.2236 | -0.3159 | -0.1970 | 0.1204 | 0.2823 | 0.2084 | 0.1667 | 0.0883 | 0.0072 | -0.2281 | -0.1210 |
| T7-like viruses | -0.5269 | -0.6396 | -0.7497 | 0.1520 | 0.3958 | 0.6112 | 0.8004 | 0.6487 | -0.4609 | 0.6147 | -0.6280 | -0.7455 | -0.5957 |
| TM4-like viruses | 0.1950 | -0.3647 | -0.3950 | -0.1638 | 0.3800 | 0.2620 | 0.2049 | 0.0044 | -0.5356 | 0.2426 | -0.3873 | -0.3496 | -0.3642 |
| TP21-like viruses | -0.6664 | -0.7262 | -0.8352 | 0.4455 | 0.5996 | 0.7220 | 0.9101 | 0.7716 | -0.6494 | 0.7553 | -0.8226 | -0.8390 | -0.6968 |
| Twort-like viruses | 0.4821 | 0.6945 | 0.6620 | -0.5047 | -0.1423 | -0.6260 | -0.5996 | -0.6163 | 0.5475 | -0.6549 | 0.7444 | 0.7666 | 0.8922 |
| VHML-like viruses | -0.1575 | -0.1522 | -0.2236 | -0.3159 | -0.1970 | 0.1204 | 0.2823 | 0.2084 | 0.1667 | 0.0883 | 0.0072 | -0.2281 | -0.1210 |
| Wbeta-like viruses | -0.6066 | -0.7088 | -0.7736 | 0.1777 | 0.3561 | 0.6879 | 0.7898 | 0.7156 | -0.4591 | 0.6703 | -0.6043 | -0.7737 | -0.6471 |

**Supplemental Table 9: Person’s Correlation Coefficient Results for Bacterial Family and Bacteriophage Genera Continued**

|  | Porphyromonadaceae | Prevotellaceae | Pseudomonadaceae | Rikenellaceae | Ruminococcaceae | Sphingobacteriaceae | Spirochaetaceae | Staphylococcaceae | Streptococcaceae | Succinivibrionaceae | Synergistaceae |
| --- | --- | --- | --- | --- | --- | --- | --- | --- | --- | --- | --- |
| 0305phi8-36-like viruses | 0.6524 | 0.6817 | -0.5378 | -0.2581 | 0.5392 | 0.5921 | 0.5193 | 0.0847 | -0.3461 | 0.6197 | 0.5067 |
| 1706-like viruses | 0.5764 | 0.6263 | -0.9494 | 0.4397 | 0.9524 | 0.5629 | 0.9455 | -0.7027 | -0.8433 | 0.9795 | 0.9926 |
| 3a-like viruses | 0.5026 | 0.5316 | -0.5818 | -0.3263 | 0.6206 | 0.4114 | 0.5266 | 0.0595 | -0.3092 | 0.6450 | 0.5620 |
| 77-like viruses | 0.5075 | 0.5616 | -0.9510 | 0.5690 | 0.9406 | 0.5121 | 0.9548 | -0.7993 | -0.8680 | 0.9649 | 0.9915 |
| 936-like viruses | -0.4250 | -0.5023 | 0.3912 | -0.0916 | -0.3287 | -0.5357 | -0.4792 | 0.1927 | 0.4059 | -0.6072 | -0.5681 |
| Bcep22-like viruses | 0.2457 | 0.2853 | -0.6800 | 0.8849 | 0.6422 | 0.3146 | 0.7247 | -0.9617 | -0.7861 | 0.6519 | 0.7422 |
| Bcep781-like viruses | 0.5267 | 0.5598 | -0.5927 | -0.2905 | 0.6245 | 0.4492 | 0.5487 | 0.0284 | -0.3412 | 0.6700 | 0.5891 |
| BcepMu-like viruses | 0.5696 | 0.5279 | 0.1572 | 0.2877 | -0.2415 | 0.6159 | -0.0622 | 0.0157 | -0.1688 | -0.1807 | -0.2494 |
| Bpp-1-like viruses | 0.6886 | 0.7171 | -0.9173 | 0.2782 | 0.9432 | 0.6293 | 0.8926 | -0.5556 | -0.7930 | 0.9285 | 0.9199 |
| c2-like viruses | 0.4928 | 0.4998 | -0.3866 | -0.4886 | 0.4168 | 0.3715 | 0.3185 | 0.3243 | -0.1031 | 0.4070 | 0.2756 |
| Che8-like viruses | 0.9257 | 0.9267 | -0.4355 | 0.1719 | 0.3751 | 0.9239 | 0.4937 | -0.1196 | -0.5334 | 0.4765 | 0.3658 |
| D3112-like viruses | -0.4728 | -0.4838 | 0.5432 | -0.2473 | -0.4785 | -0.4010 | -0.5083 | 0.1587 | 0.3721 | -0.4115 | -0.2798 |
| D3-like viruses | 0.5696 | 0.5279 | 0.1572 | 0.2877 | -0.2415 | 0.6159 | -0.0622 | 0.0157 | -0.1688 | -0.1807 | -0.2494 |
| F116-like viruses | -0.5154 | -0.4523 | 0.3408 | -0.3516 | -0.4224 | -0.4429 | -0.3306 | 0.4987 | 0.5411 | -0.2608 | -0.3585 |
| FelixO1-like viruses | 0.5735 | 0.6028 | -0.5962 | -0.2666 | 0.6259 | 0.4963 | 0.5568 | 0.0156 | -0.3666 | 0.6703 | 0.5881 |
| HAP1-like viruses | -0.2255 | -0.3253 | 0.6772 | -0.2877 | -0.6198 | -0.3083 | -0.7155 | 0.4588 | 0.5294 | -0.8148 | -0.7962 |
| Hp1-like viruses | 0.1521 | 0.1892 | -0.6132 | 0.8784 | 0.5741 | 0.2208 | 0.6530 | -0.9309 | -0.7140 | 0.5693 | 0.6623 |
| IEBH-like viruses | 0.5245 | 0.5754 | -0.9210 | 0.3196 | 0.9371 | 0.4980 | 0.9033 | -0.6143 | -0.7691 | 0.9602 | 0.9693 |
| Jersey-like viruses | 0.1521 | 0.1892 | -0.6132 | 0.8784 | 0.5741 | 0.2208 | 0.6530 | -0.9309 | -0.7140 | 0.5693 | 0.6623 |
| JS98-like viruses | -0.4728 | -0.4838 | 0.5432 | -0.2473 | -0.4785 | -0.4010 | -0.5083 | 0.1587 | 0.3721 | -0.4115 | -0.2798 |
| K-like viruses | -0.2716 | -0.3503 | 0.1710 | -0.2878 | -0.0647 | -0.4518 | -0.3068 | 0.2431 | 0.3239 | -0.3899 | -0.3705 |
| KP34-like viruses | 0.2066 | 0.2349 | -0.5636 | 0.9334 | 0.5057 | 0.2784 | 0.6137 | -0.9098 | -0.7069 | 0.4960 | 0.5713 |
| L5-like viruses | 0.3006 | 0.3480 | -0.6131 | 0.1887 | 0.6540 | 0.3231 | 0.6245 | -0.5108 | -0.5692 | 0.7350 | 0.8060 |
| Lambda-like viruses | 0.6531 | 0.6757 | -0.6944 | -0.1548 | 0.7252 | 0.5618 | 0.6495 | -0.0956 | -0.4755 | 0.7338 | 0.6559 |
| LUZ24-like viruses | 0.2297 | 0.2279 | 0.3472 | 0.0624 | -0.4740 | 0.3157 | -0.2524 | 0.3078 | 0.1601 | -0.3223 | -0.4439 |
| Omega-like viruses | 0.1437 | 0.1784 | -0.6112 | 0.8740 | 0.5755 | 0.2066 | 0.6465 | -0.9284 | -0.7074 | 0.5595 | 0.6537 |
| P100-like viruses | 0.4299 | 0.3517 | 0.2351 | 0.1458 | -0.2674 | 0.3900 | -0.2063 | 0.1305 | -0.0121 | -0.3619 | -0.4200 |
| P1-like viruses | 0.6247 | 0.6508 | -0.5887 | -0.2457 | 0.6118 | 0.5504 | 0.5567 | 0.0170 | -0.3835 | 0.6616 | 0.5731 |
| P22-like viruses | 0.0274 | -0.0127 | 0.5704 | 0.0067 | -0.5788 | 0.1217 | -0.4707 | 0.1442 | 0.1856 | -0.4778 | -0.4187 |
| P2-like viruses | 0.5912 | 0.6339 | -0.7680 | -0.0374 | 0.7912 | 0.5361 | 0.7393 | -0.2474 | -0.5604 | 0.8406 | 0.7911 |
| P335-like viruses | -0.5209 | -0.5498 | 0.9420 | -0.3943 | -0.9787 | -0.4587 | -0.9015 | 0.6893 | 0.8013 | -0.9141 | -0.9426 |
| P68-like viruses | -0.7029 | -0.7554 | 0.8913 | -0.6990 | -0.8387 | -0.7562 | -0.9500 | 0.8230 | 0.9489 | -0.9379 | -0.9458 |
| PAKP1-like viruses | -0.2716 | -0.3503 | 0.1710 | -0.2878 | -0.0647 | -0.4518 | -0.3068 | 0.2431 | 0.3239 | -0.3899 | -0.3705 |
| Phi29-like viruses | 0.6061 | 0.6147 | -0.5604 | -0.3064 | 0.6042 | 0.4938 | 0.5027 | 0.0715 | -0.3306 | 0.5900 | 0.5013 |
| phiCD119-like viruses | 0.5652 | 0.6139 | -0.9562 | 0.6464 | 0.9380 | 0.5750 | 0.9698 | -0.8470 | -0.9159 | 0.9591 | 0.9867 |
| phiE125-like viruses | 0.5867 | 0.6419 | -0.9478 | 0.4152 | 0.9425 | 0.5786 | 0.9475 | -0.6685 | -0.8300 | 0.9887 | 0.9889 |
| phiETA-like viruses | -0.6837 | -0.6689 | 0.5799 | -0.4524 | -0.6181 | -0.6733 | -0.6122 | 0.6388 | 0.7600 | -0.5991 | -0.6727 |
| phiFL-like viruses | -0.5289 | -0.5942 | 0.9441 | -0.5903 | -0.9057 | -0.5552 | -0.9639 | 0.7710 | 0.8617 | -0.9726 | -0.9741 |
| phiKZ-like viruses | 0.5197 | 0.5514 | -0.5719 | -0.3121 | 0.6050 | 0.4410 | 0.5273 | 0.0523 | -0.3205 | 0.6503 | 0.5681 |
| phiLJ1-like viruses | 0.2125 | 0.2574 | -0.6986 | 0.8083 | 0.6778 | 0.2775 | 0.7357 | -0.9463 | -0.7739 | 0.6950 | 0.7967 |
| phiPLPE-like viruses | 0.8428 | 0.8799 | -0.9014 | 0.3753 | 0.8779 | 0.8254 | 0.9195 | -0.5489 | -0.8500 | 0.9386 | 0.8925 |
| RB49-like viruses | 0.5075 | 0.5616 | -0.9510 | 0.5690 | 0.9406 | 0.5121 | 0.9548 | -0.7993 | -0.8680 | 0.9649 | 0.9915 |
| rV5-like viruses | 0.0751 | 0.1117 | -0.5806 | 0.8413 | 0.5431 | 0.1345 | 0.6084 | -0.8851 | -0.6479 | 0.5203 | 0.6084 |
| Schizot4-like viruses | -0.2716 | -0.3503 | 0.1710 | -0.2878 | -0.0647 | -0.4518 | -0.3068 | 0.2431 | 0.3239 | -0.3899 | -0.3705 |
| Secunda5-like viruses | 0.2160 | 0.2492 | -0.6060 | 0.9234 | 0.5572 | 0.2908 | 0.6567 | -0.9441 | -0.7437 | 0.5589 | 0.6460 |
| Sfi11-like viruses | -0.6866 | -0.7552 | 0.8381 | -0.5625 | -0.7673 | -0.7569 | -0.9060 | 0.6705 | 0.8539 | -0.9302 | -0.9035 |
| Sfi21-like viruses | 0.5723 | 0.6130 | -0.9013 | 0.1893 | 0.9304 | 0.5145 | 0.8662 | -0.4943 | -0.7135 | 0.9291 | 0.9175 |
| SP18-like viruses | -0.4021 | -0.3427 | -0.1283 | -0.2379 | 0.1909 | -0.3697 | 0.1029 | -0.0741 | 0.0540 | 0.2757 | 0.3610 |
| SP6-like viruses | -0.0804 | -0.0687 | 0.5850 | -0.1171 | -0.7024 | 0.0410 | -0.4841 | 0.4676 | 0.4123 | -0.4982 | -0.5936 |
| SPbeta-like viruses | 0.6955 | 0.7343 | -0.8194 | 0.0730 | 0.8307 | 0.6452 | 0.8022 | -0.3277 | -0.6548 | 0.8786 | 0.8259 |
| SPO1-like viruses | 0.2001 | 0.1474 | -0.0984 | -0.5980 | 0.2206 | 0.0130 | -0.0210 | 0.3741 | 0.1221 | 0.0326 | -0.0021 |
| T4-like viruses | 0.6044 | 0.6593 | -0.9654 | 0.5830 | 0.9403 | 0.6154 | 0.9811 | -0.7833 | -0.9012 | 0.9865 | 0.9936 |
| T5-like viruses | 0.5696 | 0.5279 | 0.1572 | 0.2877 | -0.2415 | 0.6159 | -0.0622 | 0.0157 | -0.1688 | -0.1807 | -0.2494 |
| T7-like viruses | -0.0444 | -0.1050 | 0.6146 | -0.1841 | -0.6595 | -0.0612 | -0.5987 | 0.5255 | 0.4738 | -0.7092 | -0.7921 |
| TM4-like viruses | -0.2716 | -0.3503 | 0.1710 | -0.2878 | -0.0647 | -0.4518 | -0.3068 | 0.2431 | 0.3239 | -0.3899 | -0.3705 |
| TP21-like viruses | -0.3812 | -0.4251 | 0.7203 | -0.3700 | -0.7522 | -0.4033 | -0.7358 | 0.6726 | 0.7090 | -0.8078 | -0.8867 |
| Twort-like viruses | 0.5976 | 0.6301 | -0.6577 | -0.1933 | 0.6848 | 0.5249 | 0.6214 | -0.0661 | -0.4354 | 0.7289 | 0.6541 |
| VHML-like viruses | 0.5696 | 0.5279 | 0.1572 | 0.2877 | -0.2415 | 0.6159 | -0.0622 | 0.0157 | -0.1688 | -0.1807 | -0.2494 |
| Wbeta-like viruses | -0.0192 | -0.0794 | 0.7067 | -0.1407 | -0.7530 | 0.0090 | -0.6520 | 0.4816 | 0.4495 | -0.7420 | -0.7924 |

**Supplemental Table 9: Person’s Correlation Coefficient Results for Bacterial Family and Bacteriophage Genera Continued**

|  | Syntrophomonadaceae | Thermoanaerobacteraceae | Thermoanaerobacterales Family III | Veillonellaceae | Verrucomicrobiaceae |
| --- | --- | --- | --- | --- | --- |
| 0305phi8-36-like viruses | 0.8285 | 0.7268 | 0.7367 | 0.7379 | -0.3665 |
| 1706-like viruses | 0.9204 | 0.9619 | 0.8844 | 0.9494 | -0.5690 |
| 3a-like viruses | 0.8299 | 0.7322 | 0.6830 | 0.7312 | -0.4227 |
| 77-like viruses | 0.8398 | 0.9097 | 0.8283 | 0.8955 | -0.5778 |
| 936-like viruses | -0.6350 | -0.6305 | -0.6128 | -0.6329 | 0.7217 |
| Bcep22-like viruses | 0.3856 | 0.5337 | 0.4785 | 0.5186 | -0.3485 |
| Bcep781-like viruses | 0.8491 | 0.7570 | 0.7107 | 0.7564 | -0.4544 |
| BcepMu-like viruses | -0.0596 | -0.0710 | 0.1513 | -0.0296 | 0.5477 |
| Bpp-1-like viruses | 0.9710 | 0.9708 | 0.9200 | 0.9641 | -0.4026 |
| c2-like viruses | 0.6555 | 0.5192 | 0.5262 | 0.5302 | -0.1769 |
| Che8-like viruses | 0.6657 | 0.6106 | 0.7553 | 0.6434 | 0.0268 |
| D3112-like viruses | -0.3834 | -0.3648 | -0.4329 | -0.3847 | 0.0164 |
| D3-like viruses | -0.0596 | -0.0710 | 0.1513 | -0.0296 | 0.5477 |
| F116-like viruses | -0.3667 | -0.3941 | -0.4289 | -0.3926 | -0.4502 |
| FelixO1-like viruses | 0.8595 | 0.7672 | 0.7336 | 0.7688 | -0.4146 |
| HAP1-like viruses | -0.6691 | -0.7149 | -0.6097 | -0.7008 | 0.9406 |
| Hp1-like viruses | 0.2803 | 0.4336 | 0.3760 | 0.4177 | -0.3117 |
| IEBH-like viruses | 0.9239 | 0.9490 | 0.8550 | 0.9342 | -0.6017 |
| Jersey-like viruses | 0.2803 | 0.4336 | 0.3760 | 0.4177 | -0.3117 |
| JS98-like viruses | -0.3834 | -0.3648 | -0.4329 | -0.3847 | 0.0164 |
| K-like viruses | -0.3254 | -0.3676 | -0.3843 | -0.3741 | 0.6376 |
| KP34-like viruses | 0.2108 | 0.3642 | 0.3477 | 0.3561 | -0.1868 |
| L5-like viruses | 0.7210 | 0.7534 | 0.6351 | 0.7307 | -0.6005 |
| Lambda-like viruses | 0.9089 | 0.8281 | 0.8028 | 0.8305 | -0.3525 |
| LUZ24-like viruses | -0.2712 | -0.2980 | -0.1106 | -0.2585 | 0.2477 |
| Omega-like viruses | 0.2708 | 0.4237 | 0.3652 | 0.4075 | -0.2917 |
| P100-like viruses | -0.2125 | -0.2437 | -0.0337 | -0.2062 | 0.8390 |
| P1-like viruses | 0.8623 | 0.7682 | 0.7523 | 0.7732 | -0.3740 |
| P22-like viruses | -0.3645 | -0.3574 | -0.2494 | -0.3433 | 0.4198 |
| P2-like viruses | 0.9466 | 0.8981 | 0.8368 | 0.8930 | -0.5403 |
| P335-like viruses | -0.8680 | -0.9039 | -0.8152 | -0.8880 | 0.4104 |
| P68-like viruses | -0.8399 | -0.9149 | -0.9055 | -0.9142 | 0.4968 |
| PAKP1-like viruses | -0.3254 | -0.3676 | -0.3843 | -0.3741 | 0.6376 |
| Phi29-like viruses | 0.8164 | 0.7099 | 0.6980 | 0.7151 | -0.2298 |
| phiCD119-like viruses | 0.8314 | 0.9091 | 0.8482 | 0.8981 | -0.5114 |
| phiE125-like viruses | 0.9340 | 0.9694 | 0.8955 | 0.9586 | -0.6084 |
| phiETA-like viruses | -0.6725 | -0.7100 | -0.7251 | -0.7082 | -0.0186 |
| phiFL-like viruses | -0.8328 | -0.9023 | -0.8373 | -0.8927 | 0.6406 |
| phiKZ-like viruses | 0.8365 | 0.7410 | 0.6964 | 0.7408 | -0.4423 |
| phiLJ1-like viruses | 0.4426 | 0.5840 | 0.5002 | 0.5636 | -0.4350 |
| phiPLPE-like viruses | 0.9867 | 0.9869 | 0.9941 | 0.9927 | -0.4086 |
| RB49-like viruses | 0.8398 | 0.9097 | 0.8283 | 0.8955 | -0.5778 |
| rV5-like viruses | 0.2173 | 0.3685 | 0.3037 | 0.3516 | -0.3013 |
| Schizot4-like viruses | -0.3254 | -0.3676 | -0.3843 | -0.3741 | 0.6376 |
| Secunda5-like viruses | 0.2783 | 0.4328 | 0.3984 | 0.4212 | -0.2576 |
| Sfi11-like viruses | -0.8558 | -0.9071 | -0.8997 | -0.9096 | 0.6290 |
| Sfi21-like viruses | 0.9534 | 0.9484 | 0.8660 | 0.9369 | -0.5258 |
| SP18-like viruses | 0.2000 | 0.2170 | 0.0148 | 0.1790 | -0.7098 |
| SP6-like viruses | -0.4708 | -0.4967 | -0.3563 | -0.4665 | 0.1110 |
| SPbeta-like viruses | 0.9806 | 0.9413 | 0.9046 | 0.9405 | -0.4850 |
| SPO1-like viruses | 0.3117 | 0.1843 | 0.1639 | 0.1828 | 0.2861 |
| T4-like viruses | 0.8823 | 0.9446 | 0.8884 | 0.9361 | -0.5660 |
| T5-like viruses | -0.0596 | -0.0710 | 0.1513 | -0.0296 | 0.5477 |
| T7-like viruses | -0.6006 | -0.6534 | -0.4797 | -0.6203 | 0.7209 |
| TM4-like viruses | -0.3254 | -0.3676 | -0.3843 | -0.3741 | 0.6376 |
| TP21-like viruses | -0.7553 | -0.8116 | -0.7055 | -0.7902 | 0.5508 |
| Twort-like viruses | 0.8969 | 0.8165 | 0.7779 | 0.8168 | -0.4439 |
| VHML-like viruses | -0.0596 | -0.0710 | 0.1513 | -0.0296 | 0.5477 |
| Wbeta-like viruses | -0.6160 | -0.6557 | -0.4727 | -0.6229 | 0.7129 |
