# Supplementary material for: Integrative Longitudinal Analysis of Metabolic Phenotype and Microbiota Changes During the Development of Obesity
Source: Front Cell Infect Microbiol. 2021 Aug 3;11:671926. doi: 10.3389/fcimb.2021.671926 (PMC8370388; doi:10.3389/fcimb.2021.671926)
Supplement: Supplementary file 11 [file Table_10.docx]

**Supplemental Table 10: Person’s Correlation Coefficient Results for Bacterial Family and Metabolic Parameters**

|  | Day EE | Night RER | HOMA IR | Percent Weight Change | Visceral Fat |
| --- | --- | --- | --- | --- | --- |
| Actinomycetaceae | -0.8111 | -0.6705 | -0.5951 | -0.8234 | -0.7408 |
| Aerococcaceae | -0.7992 | -0.6187 | -0.6225 | -0.8464 | -0.8187 |
| Bacillaceae | 0.7341 | 0.5615 | 0.3769 | 0.7713 | 0.7757 |
| Bacteroidaceae | -0.9122 | -0.7727 | -0.8026 | -0.9762 | -0.9332 |
| Bifidobacteriaceae | 0.2048 | 0.3964 | 0.5303 | 0.0310 | -0.2831 |
| Brachyspiraceae | -0.8315 | -0.6400 | -0.6040 | -0.8781 | -0.8420 |
| Burkholderiaceae | -0.8237 | -0.5252 | -0.5717 | -0.8741 | -0.9352 |
| Campylobacteraceae | -0.9362 | -0.7676 | -0.7619 | -0.9798 | -0.9287 |
| Carnobacteriaceae | -0.5648 | -0.3023 | -0.5250 | -0.6654 | -0.6795 |
| Chlorobiaceae | -0.8910 | -0.7609 | -0.7018 | -0.9636 | -0.9493 |
| Clostridiaceae | -0.8413 | -0.7081 | -0.6524 | -0.8586 | -0.7886 |
| Clostridiales Family XI | -0.8573 | -0.6856 | -0.6570 | -0.8982 | -0.8563 |
| Cytophagaceae | -0.8508 | -0.7247 | -0.7420 | -0.9605 | -0.9622 |
| Desulfovibrionaceae | -0.8438 | -0.6818 | -0.6791 | -0.8984 | -0.8539 |
| Enterobacteriaceae | -0.8465 | -0.8143 | -0.8260 | -0.9034 | -0.8503 |
| Enterococcaceae | 0.8554 | 0.6564 | 0.5898 | 0.8905 | 0.8843 |
| Erysipelotrichaceae | -0.5509 | -0.2811 | -0.2207 | -0.6668 | -0.7856 |
| Eubacteriaceae | -0.8143 | -0.6829 | -0.6302 | -0.8411 | -0.7627 |
| Fibrobacteraceae | -0.8348 | -0.6176 | -0.6678 | -0.8820 | -0.8767 |
| Flavobacteriaceae | -0.8538 | -0.6784 | -0.7465 | -0.9579 | -0.9648 |
| Fusobacteriaceae | -0.8706 | -0.7136 | -0.6747 | -0.8954 | -0.8459 |
| Geobacteraceae | -0.9000 | -0.6649 | -0.6876 | -0.9607 | -0.9696 |
| Halanaerobiaceae | -0.7622 | -0.5543 | -0.6175 | -0.8246 | -0.8278 |
| Helicobacteraceae | -0.7111 | -0.6121 | -0.6153 | -0.7404 | -0.7868 |
| Heliobacteriaceae | -0.8592 | -0.7834 | -0.7347 | -0.8773 | -0.7646 |
| Lachnospiraceae | -0.8125 | -0.6949 | -0.6311 | -0.8285 | -0.7434 |
| Lactobacillaceae | 0.7495 | 0.7408 | 0.8846 | 0.6273 | 0.3630 |
| Leuconostocaceae | 0.8563 | 0.8019 | 0.6569 | 0.8056 | 0.6289 |
| Listeriaceae | 0.9014 | 0.8316 | 0.7817 | 0.9014 | 0.7834 |
| Micrococcaceae | 0.6679 | 0.4680 | 0.4253 | 0.6904 | 0.7270 |
| Moraxellaceae | 0.8178 | 0.6272 | 0.6089 | 0.7925 | 0.7762 |
| Neisseriaceae | -0.8812 | -0.8049 | -0.7228 | -0.9470 | -0.8591 |
| Paenibacillaceae | 0.8335 | 0.6614 | 0.5894 | 0.8621 | 0.8496 |
| Pasteurellaceae | -0.8748 | -0.7149 | -0.7484 | -0.9222 | -0.8697 |
| Peptococcaceae | -0.7943 | -0.6609 | -0.6534 | -0.8305 | -0.7772 |
| Peptostreptococcaceae | -0.7661 | -0.5712 | -0.6063 | -0.8087 | -0.7687 |
| Porphyromonadaceae | -0.8595 | -0.7202 | -0.8117 | -0.9485 | -0.9313 |
| Prevotellaceae | -0.8540 | -0.7328 | -0.8081 | -0.9526 | -0.9290 |
| Pseudomonadaceae | 0.8340 | 0.6737 | 0.5953 | 0.8381 | 0.8050 |
| Rikenellaceae | -0.6181 | -0.6397 | -0.3719 | -0.6410 | -0.5888 |
| Ruminococcaceae | -0.8371 | -0.7049 | -0.6916 | -0.8282 | -0.7760 |
| Spirochaetaceae | -0.8883 | -0.7430 | -0.6579 | -0.8961 | -0.8239 |
| Staphylococcaceae | 0.7309 | 0.7180 | 0.5544 | 0.7303 | 0.6532 |
| Streptococcaceae | 0.8290 | 0.8730 | 0.9439 | 0.7488 | 0.4722 |
| Succinivibrionaceae | -0.8574 | -0.7211 | -0.6915 | -0.8562 | -0.7436 |
| Synergistaceae | -0.8180 | -0.7217 | -0.6980 | -0.8335 | -0.7399 |
| Syntrophomonadaceae | -0.8007 | -0.5737 | -0.6497 | -0.8481 | -0.8317 |
| Thermoanaerobacteraceae | -0.8332 | -0.6480 | -0.6828 | -0.8816 | -0.8475 |
| Thermoanaerobacterales Family III | -0.8741 | -0.6776 | -0.7146 | -0.9420 | -0.9223 |
| Bacterial Diversity | -0.8662 | 0.7021 | -0.9175 | -0.9334 | -0.8651 |
| Bacterial Evenness | -0.8578 | 0.6998 | -0.9103 | -0.9267 | -0.8560 |
