# Supplementary material for: Integrative Longitudinal Analysis of Metabolic Phenotype and Microbiota Changes During the Development of Obesity
Source: Front Cell Infect Microbiol. 2021 Aug 3;11:671926. doi: 10.3389/fcimb.2021.671926 (PMC8370388; doi:10.3389/fcimb.2021.671926)
Supplement: Supplementary file 12 [file Table_11.docx]

**Supplemental Table 11: Pearson's Correlation Coefficient Results for Bacteriophage Genera and Metabolic Parameters**

|  | Day EE | Night RQ | Visceral Fat | Percent Weight Change | HOMA.IR |
| --- | --- | --- | --- | --- | --- |
| 0305phi8-36-like viruses | -0.4745 | 0.6407 | -0.5746 | -0.5330 | -0.5649 |
| 1706-like viruses | -0.7789 | 0.4452 | -0.6973 | -0.7412 | -0.7379 |
| 3a-like viruses | -0.3666 | 0.5197 | -0.4517 | -0.4012 | -0.4447 |
| 77-like viruses | -0.7689 | 0.3926 | -0.6455 | -0.7228 | -0.6928 |
| 936-like viruses | 0.7170 | -0.1628 | 0.5476 | 0.6170 | 0.7028 |
| Bcep22-like viruses | -0.6353 | 0.0905 | -0.4411 | -0.5588 | -0.4983 |
| Bcep781-like viruses | -0.4209 | 0.5145 | -0.4903 | -0.4460 | -0.4953 |
| BcepMu-like viruses | -0.2857 | 0.4657 | -0.4591 | -0.4097 | -0.3714 |
| Bpp-1-like viruses | -0.7265 | 0.5740 | -0.7525 | -0.7331 | -0.7459 |
| c2-like viruses | -0.1479 | 0.6341 | -0.3290 | -0.2567 | -0.2614 |
| Che8-like viruses | -0.6792 | 0.8086 | -0.8350 | -0.7895 | -0.7802 |
| D3112-like viruses | -0.2857 | 0.4657 | -0.4591 | -0.4097 | -0.3714 |
| D3-like viruses | 0.1793 | -0.8144 | 0.2723 | 0.3592 | 0.1486 |
| F116-like viruses | 0.4117 | -0.2234 | 0.6071 | 0.4293 | 0.5167 |
| FelixO1-like viruses | -0.4493 | 0.5477 | -0.5336 | -0.4816 | -0.5315 |
| HAP1-like viruses | 0.6324 | -0.1464 | 0.3638 | 0.5264 | 0.5155 |
| Hp1-like viruses | -0.5393 | 0.0361 | -0.3378 | -0.4659 | -0.3901 |
| IEBH-like viruses | -0.7138 | 0.4109 | -0.6379 | -0.6716 | -0.6821 |
| Jersey-like viruses | -0.5393 | 0.0361 | -0.3378 | -0.4659 | -0.3901 |
| JS98-like viruses | 0.1793 | -0.8144 | 0.2723 | 0.3592 | 0.1486 |
| K-like viruses | 0.6640 | 0.0180 | 0.4219 | 0.5426 | 0.6031 |
| KP34-like viruses | -0.5236 | 0.1222 | -0.3535 | -0.4848 | -0.3793 |
| L5-like viruses | -0.6454 | -0.0065 | -0.5173 | -0.5086 | -0.6327 |
| Lambda-like viruses | -0.5003 | 0.6397 | -0.6074 | -0.5526 | -0.5800 |
| LUZ24-like viruses | -0.0253 | 0.3042 | -0.0726 | -0.1308 | -0.0437 |
| Omega-like viruses | -0.5199 | 0.0370 | -0.3255 | -0.4502 | -0.3718 |
| P100-like viruses | -0.4763 | 0.5902 | -0.5754 | -0.5189 | -0.5662 |
| P1-like viruses | 0.0343 | 0.4652 | -0.2504 | -0.1448 | -0.0786 |
| P22-like viruses | -0.6036 | 0.5171 | -0.6181 | -0.6017 | -0.6420 |
| P23-45-like viruses | -0.0621 | -0.3350 | -0.1130 | -0.0037 | -0.1512 |
| P335-like viruses | 0.6390 | -0.4434 | 0.6185 | 0.6257 | 0.6137 |
| P68-like viruses | 0.9505 | -0.4943 | 0.8383 | 0.9165 | 0.8922 |
| PAKP1-like viruses | 0.6640 | 0.0180 | 0.4219 | 0.5426 | 0.6031 |
| Phi29-like viruses | -0.3615 | 0.6238 | -0.5211 | -0.4342 | -0.4712 |
| phiCD119-like viruses | -0.8105 | 0.4352 | -0.7006 | -0.7752 | -0.7370 |
| phiE125-like viruses | -0.7863 | 0.4679 | -0.6976 | -0.7514 | -0.7438 |
| phiETA-like viruses | 0.7599 | -0.3079 | 0.8347 | 0.7271 | 0.8280 |
| phiFL-like viruses | 0.7968 | -0.4431 | 0.6478 | 0.7590 | 0.7056 |
| phiKZ-like viruses | -0.4050 | 0.5084 | -0.4795 | -0.4313 | -0.4835 |
| phiLJ1-like viruses | -0.6442 | 0.0238 | -0.4351 | -0.5414 | -0.5127 |
| phiPLPE-like viruses | -0.8630 | 0.7117 | -0.8803 | -0.8914 | -0.8806 |
| RB49-like | -0.7689 | 0.3926 | -0.6455 | -0.7228 | -0.6928 |
| rV5-like viruses | -0.4446 | 0.0161 | -0.2417 | -0.3798 | -0.2859 |
| Schizot4-like viruses | 0.6640 | 0.0180 | 0.4219 | 0.5426 | 0.6031 |
| Secunda5-like viruses | -0.5788 | 0.0869 | -0.3928 | -0.5176 | -0.4364 |
| Sfi11-like viruses | 0.9372 | -0.5033 | 0.7991 | 0.8996 | 0.8793 |
| Sfi21-like viruses | -0.6550 | 0.4948 | -0.6409 | -0.6429 | -0.6566 |
| SP18-like viruses | -0.0395 | -0.5536 | 0.1934 | 0.1579 | 0.0224 |
| SP6-like viruses | 0.1455 | -0.0435 | 0.1847 | 0.1113 | 0.1470 |
| SPbeta-like viruses | -0.6877 | 0.6067 | -0.7176 | -0.7021 | -0.7276 |
| SPO1-like viruses | 0.2176 | 0.2923 | -0.0710 | 0.1225 | 0.0556 |
| T4-like viruses | -0.8308 | 0.4858 | -0.7228 | -0.8014 | -0.7646 |
| T5-like viruses | -0.2857 | 0.4657 | -0.4591 | -0.4097 | -0.3714 |
| T7-like viruses | 0.4665 | 0.1466 | 0.2664 | 0.3124 | 0.3949 |
| TM4-like viruses | 0.6640 | 0.0180 | 0.4219 | 0.5426 | 0.6031 |
| TP21-like viruses | 0.7271 | -0.0786 | 0.6023 | 0.6057 | 0.6987 |
| Twort-like viruses | -0.5042 | 0.5593 | -0.5736 | -0.5307 | -0.5755 |
| VHML-like viruses | -0.2857 | 0.4657 | -0.4591 | -0.4097 | -0.3714 |
| Wbeta-like viruses | 0.3394 | 0.0023 | 0.1681 | 0.2372 | 0.2524 |
| Bacteriophage Diversity | -0.7731 | 0.3597 | -0.6967 | -0.7719 | -0.6998 |
| Bacteriophage Evenness | -0.7186 | 0.3511 | -0.6583 | -0.7289 | -0.6495 |
