# Supplementary figures and images for: Integrative Longitudinal Analysis of Metabolic Phenotype and Microbiota Changes During the Development of Obesity
Source: Front Cell Infect Microbiol. 2021 Aug 3;11:671926. doi: 10.3389/fcimb.2021.671926 (PMC8370388; doi:10.3389/fcimb.2021.671926)

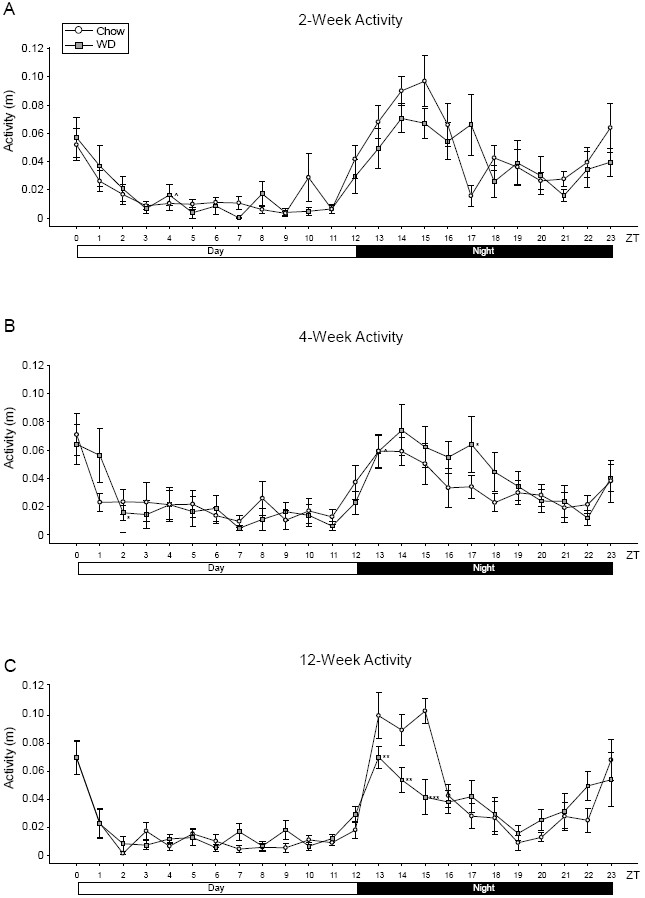

Supplement: Supplementary file 13 [file Image_1.jpeg]

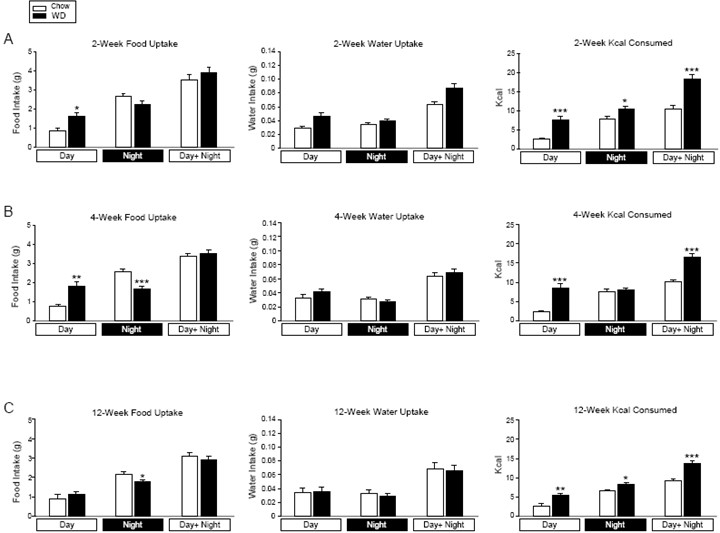

Supplement: Supplementary file 14 [file Image_2.jpeg]

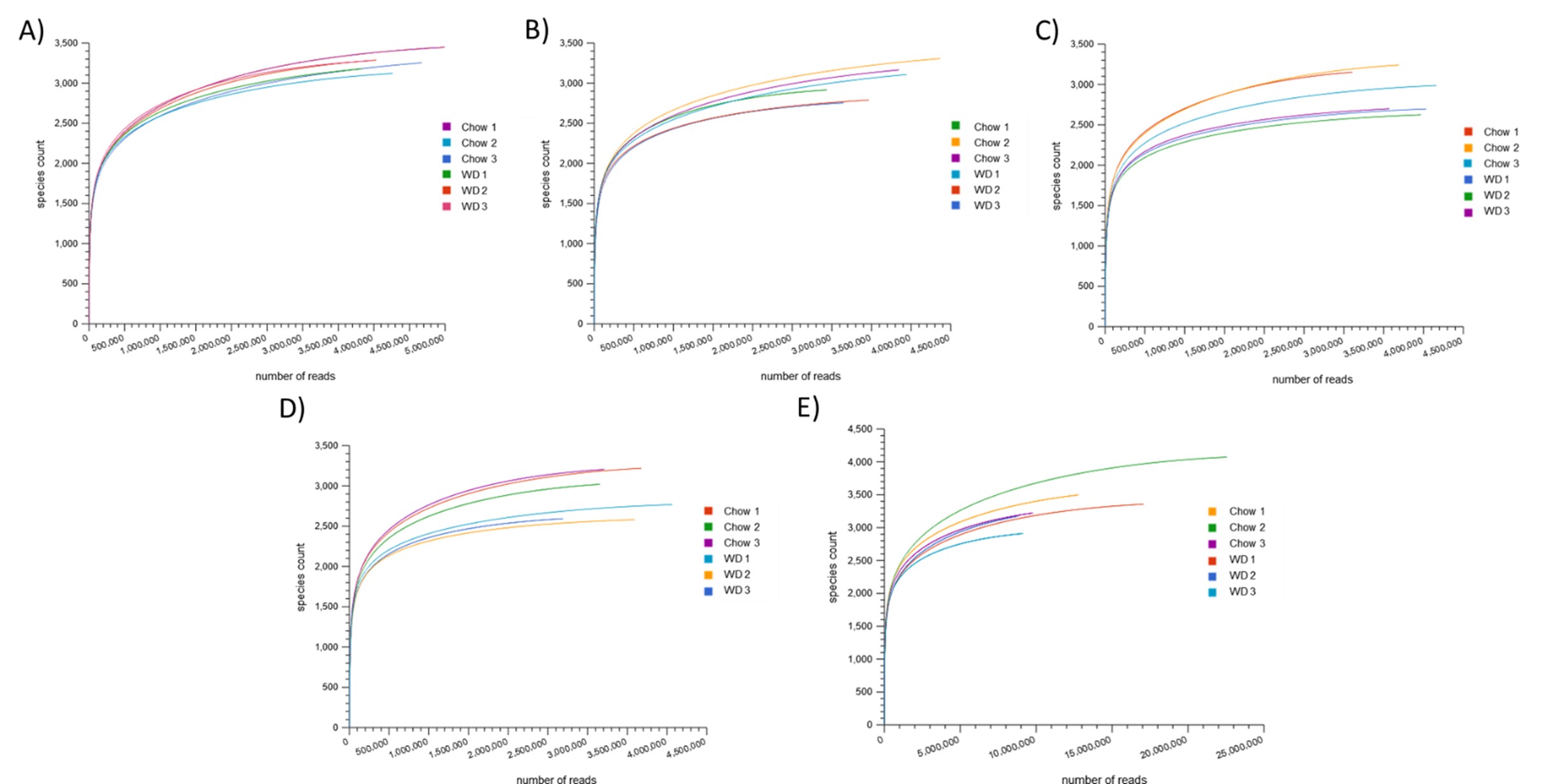

Supplement: Supplementary file 15 [file Image_3.jpeg]

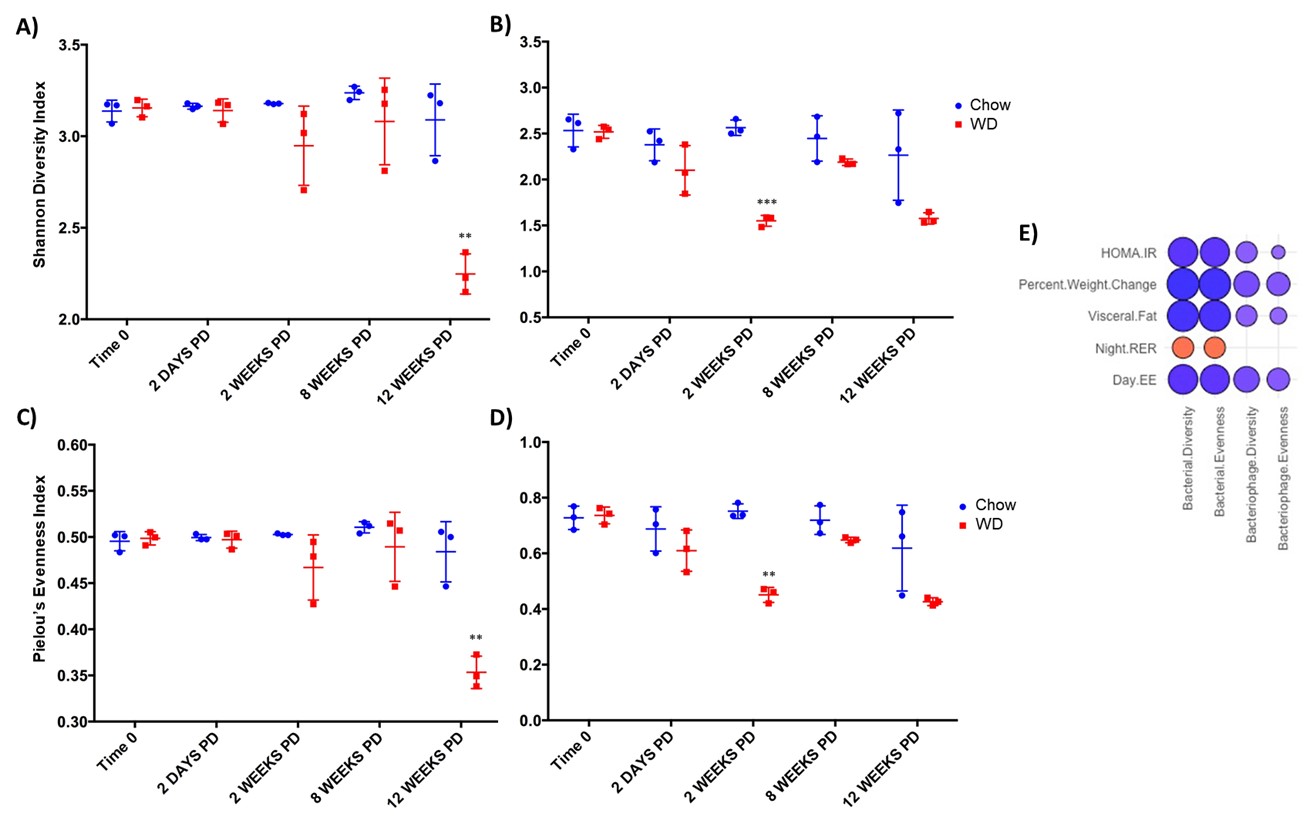

Supplement: Supplementary file 16 [file Image_4.jpeg]

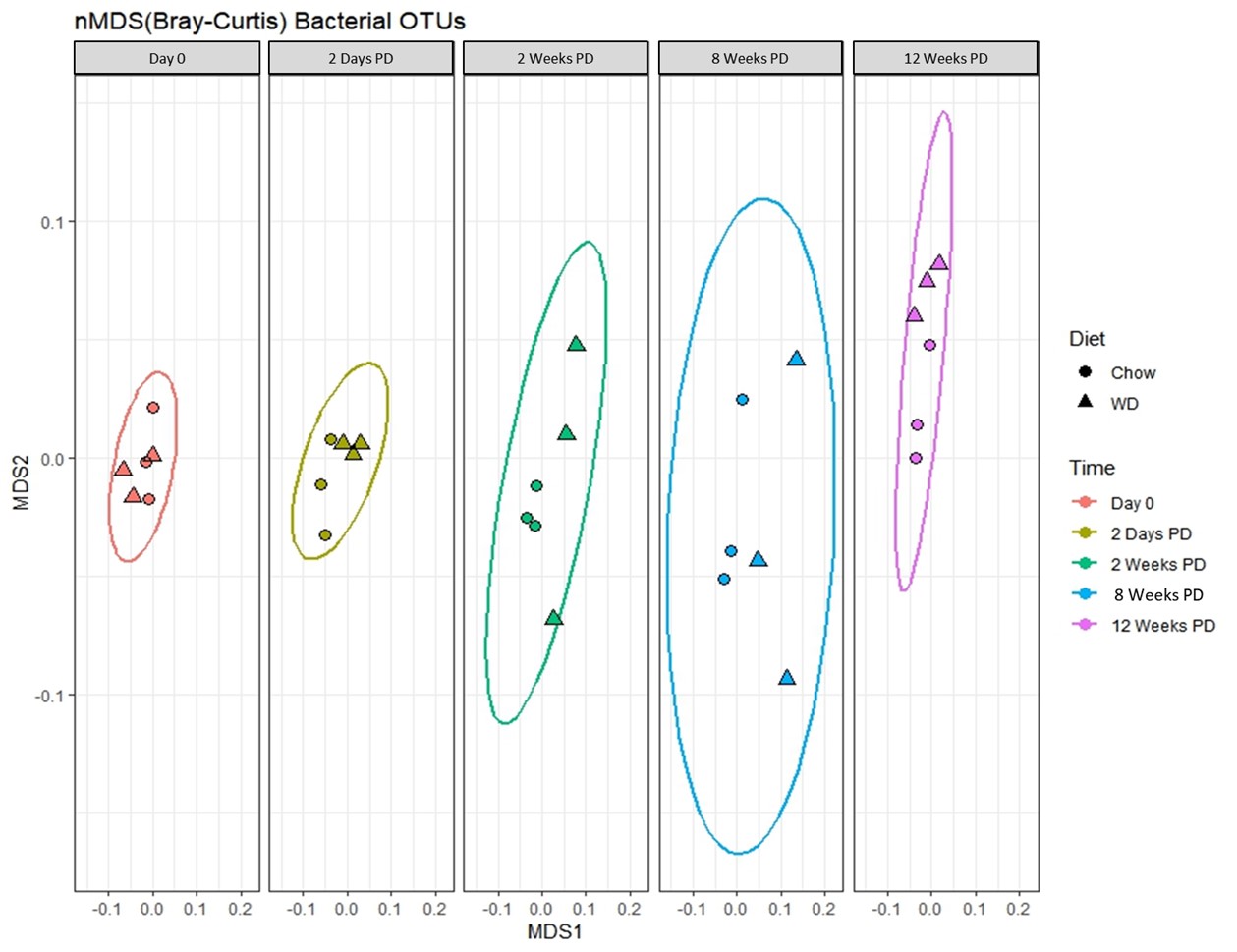

Supplement: Supplementary file 17 [file Image_5.jpeg]

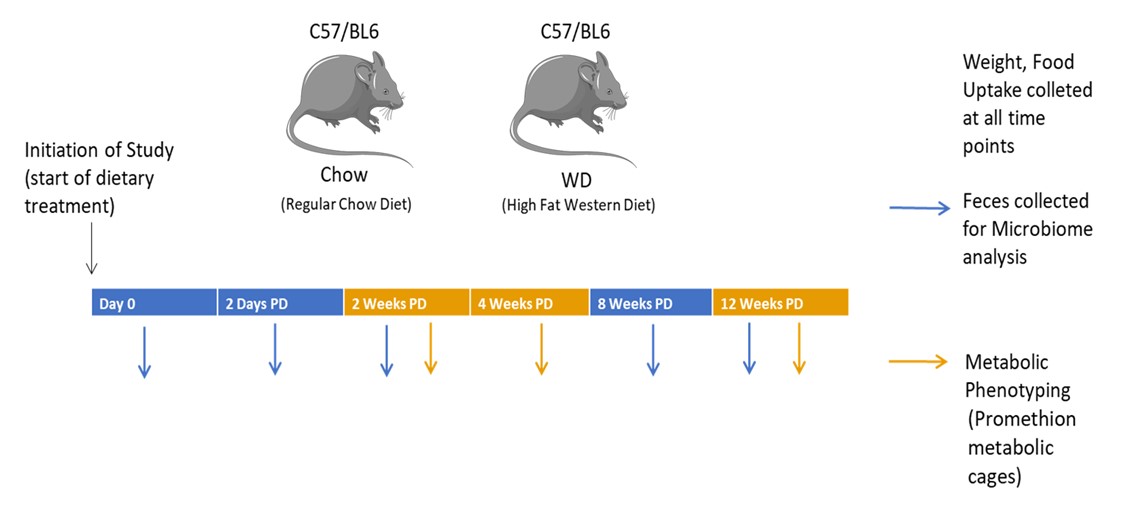

Supplement: Supplementary file 18 [file Image_6.jpeg]
